# Synthesis of Spiro-Bridged 3,4-Dihydrocoumarins Through an Organocatalytic Cascade Sequence Diels–Alder/Nucleophilic Ring Closing

**DOI:** 10.3390/molecules31162827

**Published:** 2026-08-13

**Authors:** Alberto Medina-Ortíz, José Luis Olivares-Romero, Alfonso Reyes-Luna, David Cruz Cruz, Clarisa Villegas Gómez

**Affiliations:** 1 Departamento de Química, División de Ciencias Naturales y Exactas, Universidad de Guanajuato, Noria Alta S/N, Guanajuato 36050, Mexico; a.medinaortiz@ugto.mx (A.M.-O.); a.reyes.luna@ugto.mx (A.R.-L.); 2 Red de Estudios Moleculares Avanzados, Instituto de Ecología A.C., Carretera Antigua a Coatepec 351, el Haya, Xalapa 91073, Mexico; jose.olivares@inecol.mx

**Keywords:** 3,4-dihydrocoumarins, spiro compounds, trienamine catalysis, cascade reactions, organocatalysis

## Abstract

3,4-dihydrocoumarins and spiro compounds constitute an important class of privileged frameworks present in a wide variety of natural and synthetic molecules with diverse applications. In this study, we developed an organocatalytic methodology for the construction of complex and diverse chiral spiro-bridged 3,4-dihydrocoumarins through a Diels–Alder/Nucleophilic ring-closing cascade via trienamine activation. The reaction proceeds efficiently with different trienamine precursors and a range of coumarin-3-carboxamides, which act as both dienophile and intramolecular nucleophile species. This process affords the desired products in good yields and with a high degree of stereocontrol. Furthermore, several transformations on the newly formed spiro-piperidone ring demonstrate the synthetic utility of the obtained compounds.

## 1. Introduction

During the last decades, the design and implementation of synthetic strategies for the construction of complex and diverse molecular architectures containing privileged frameworks has been one of the most challenging tasks in contemporary organic chemistry. In this regard, methodologies based on reactions capable of assembling such compounds through the formation of multiple chemical bonds in a single or sequential operation, such as one-pot, domino or multicomponent reactions, along with the generation of multiple chiral centers in a stereocontrolled fashion, have become highly valuable and attractive for these purposes due to their ability to provide a high atom-economy and to avoid the time-consuming and costly purification process [1,2,3,4,5,6,7,8,9,10,11]. In this context, an appropriate choice of the starting substrates constitutes a determining element in the rational design of a sequential synthetic methodology.

In terms of privileged structures, coumarins and their derivatives belong to an important class of relevant frameworks with a broad range of applications [12,13,14,15,16,17,18,19,20,21,22,23,24,25,26]. Particularly, the 3,4-dihydrocoumarin core constitutes an important motif present in a wide variety of natural and synthetic bioactive compounds [14,27,28,29,30,31,32,33,34,35,36,37,38]. Such activities include anticancer [39,40,41], cytotoxic [42], antimicrobial [42,43], antiviral [44], antioxidant [45,46,47], immunomodulatory [46], aldose reductase inhibition [48], antimitotic [49], estrogenic [50], and monoamine oxidase-B inhibition [51], among others. On the other hand, in recent years, spiro compounds have experienced significant interest in modern drug discovery due to their remarkable biological properties, which are associated with their three-dimensional structure, which provides important advantages over the more planar molecular entities [52,53,54,55,56,57,58,59]. For biological applications, the introduction of a spiro unit into a screened compound increases the probability of identifying a promising lead candidate.

Owing to the highly structural attractiveness of both 3,4-dihydrocoumarins and spiro compounds, we aimed to develop a strategy to incorporate both frameworks into a new complex molecular architecture through an organocatalytic sequence based on trienamine catalysis.

**Scheme 1:**
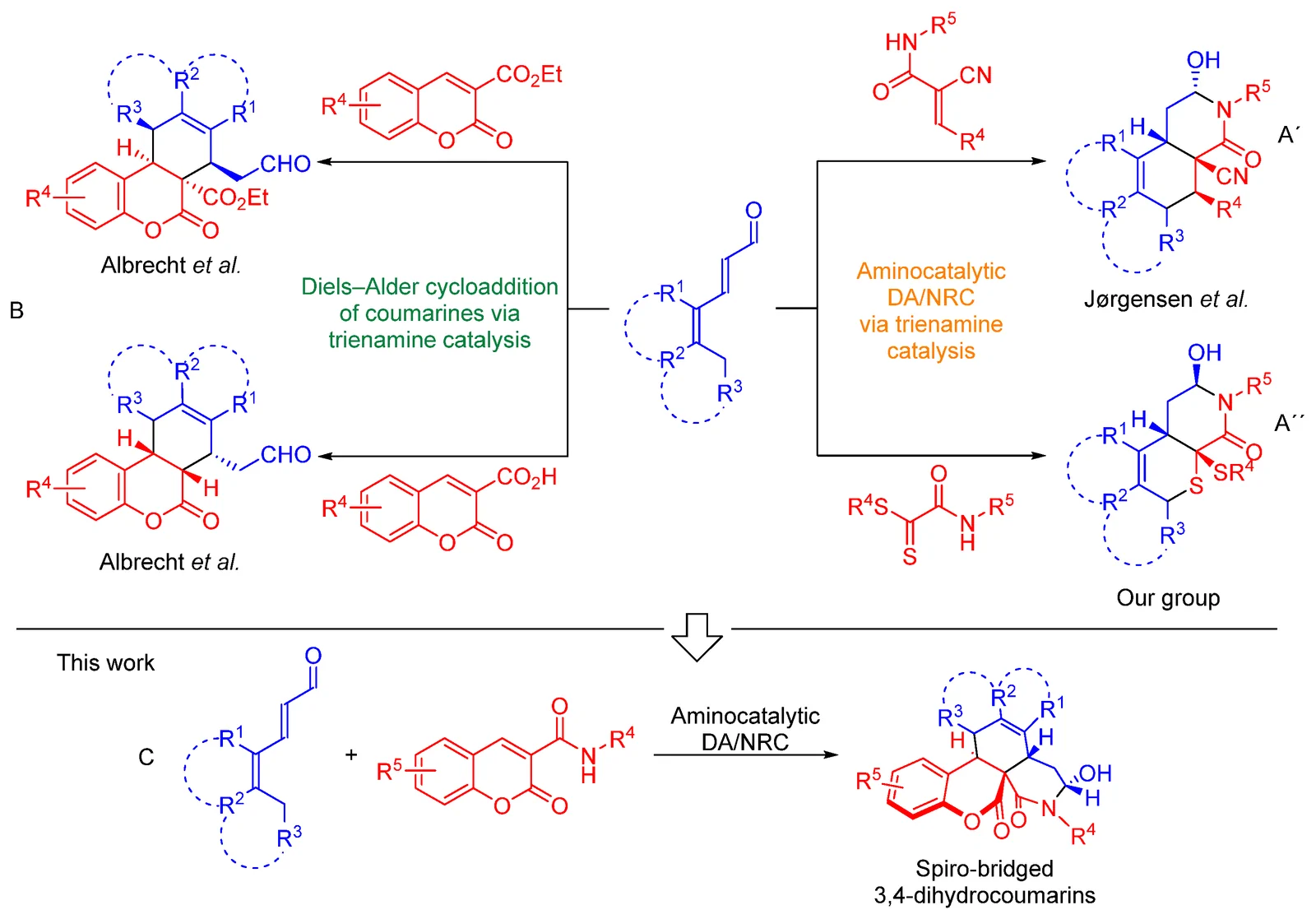
Trienamine catalysis in DA/NRC cascade reactions with (**A’**) cyanoacrylamides [60] and (**A”**) with dithioamides [62], (**B**) Diels–Alder reactions with coumarins [63,64,65,66] and (**C**) present work.

In previous work, Jørgensen and co-workers established an organocatalytic cascade methodology through a Diels–Alder cycloaddition and further nucleophilic ring-closing (DA/NRC) sequence employing cyanoacrylamides as activated dienophiles (Scheme 1A’) [60,61]. Later, our group expanded this strategy by using dithioamides as hetero-dienophiles (Scheme 1A”) [62], demonstrating that the carboxamide moiety can act as both an activating group and internal nucleophile. These results prompted us to evaluate other types of activated dienophiles. In this sense, it is well known that coumarins bearing electron-withdrawing groups at the C3 position can easily participate in Diels–Alder cycloadditions. In particular, coumarin-3-carboxylic acids and coumarin-3-carboxylates have been employed as dienophiles in organocatalytic Diels–Alder reactions mediated by dienamine and trienamine catalysis (Scheme 1B) [63,64,65,66]. To the best of our knowledge, there are no reports on this type of cycloadditions involving coumarin-3-carboxamides, despite their ready accessibility. Based on this, we envisioned a trienamine-mediated DA/NRC sequence using coumarin-3-carboxamides and 2,4-dienals to access chiral and structurally complex spiro-bridged 3,4-dihydrocoumarins.

## 2. Results and Discussion

In order to assess the viability of our hypothesis, we initially examined the reaction between 5-methyl-2,4-hexadienal **1A** and the *N*-benzylcoumarin-3-carboxamide **2a**, which was efficiently obtained from salicylaldehyde through a two-step synthesis [67]. Gratifyingly, when the reaction was carried out in the presence of 20 mol% of the Jørgensen–Hayashi catalyst **3a**, with benzoic acid as additive in CHCl_3_, the desired spiro-bridged 3,4-dihydrocoumarin **4Aa** was directly obtained without any traces of the simple Diels–Alder cycloadduct in 94% yield after 36 h at room temperature. Under these conditions, the diastereoselectivity was almost complete; however, no enantioinduction was observed (Table 1, entry 1). With these results, different catalysts were tested (**3b–d**). Promisingly, when the more sterically demanding catalyst **3b** was evaluated, the enantioselectivity was notably improved up to 76% ee along with the same yield (entry 2). Disappointingly, the catalysts α,α-bis [3,5-bis (trifluoromethyl)phenyl]-2-pyrrolidinemethanol trimethylsilyl ether **3c** and the bifunctional pyrrolidine–squaramide derivative **3d** resulted in low reactivity (entries 3 and 4). Thereafter, the reaction was screened in different solvents in the presence of catalyst **3b**. When the reaction was carried out in DCM, no conversion was observed after 36 h at room temperature (entry 5). The use of THF or CH_3_CN led to the product with moderate yields and low enantioselectivity (entries 6 and 7). A better yield and excellent enantioselectivity were obtained when dioxane was used; however, low diastereoselectivity was observed (entry 8). Finally, when the reaction was performed in toluene, the product was afforded in excellent yield (95%) and with a high degree of stereocontrol (96.5% ee, 94:6 dr) (entry 9).

**Scheme 2:**
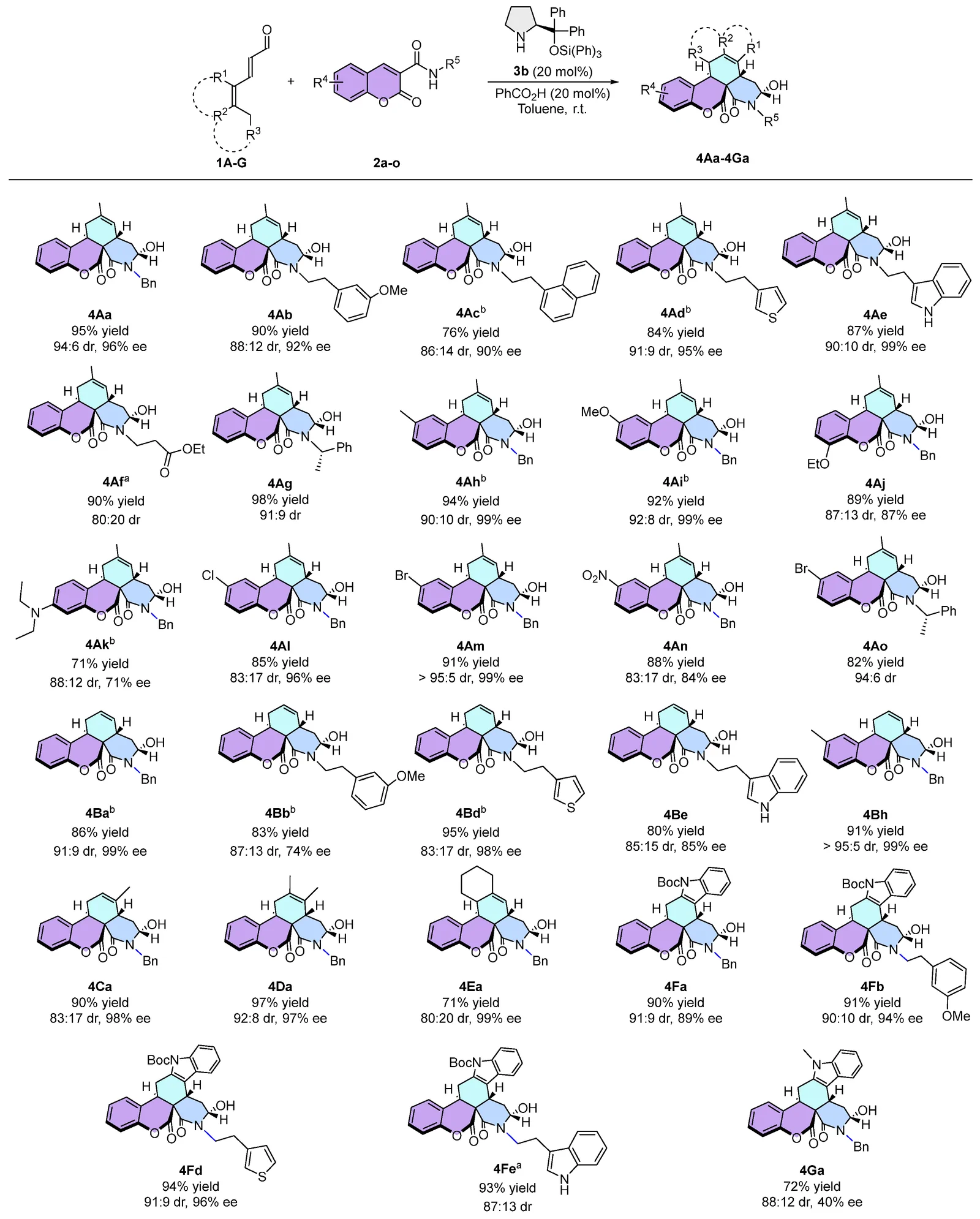
Scope of the DA/NRC process for the formation of spiro-bridged 3,4-dihydrocoumarins. The reactions were performed with 0.1 mmol of **1**, 0.066 mmol of **2**, 20 mol% of catalyst **3b** and benzoic acid as additive in 0.7 mL of toluene at room temperature. The yield is for both diastereoisomers of **4** after chromatographic purification on silica gel. The dr was determined by ^1^H NMR of the crude product. The ee was measured by chiral HPLC. ^[a]^ All the attempts to separate the enantiomeric mixture by chiral HPLC were unsuccessful. ^[b]^ Traces of starting material remain after purification.

Once we established the optimal conditions, we examined the scope and limitations of the DA/NRC sequence (Scheme 2). Initially, we evaluated the behavior of the 2,4-dienal **1A** with a series of coumarin-3-carboxamides **2**. In general, a wide range of substitution patterns at different positions of **2** was well-tolerated. Aromatic and hetero-aromatic substituents on the amide moiety (**2a–e**) resulted in good yields (76–95%) and with excellent stereoselectivities (86:14–94:6 dr, 90–99% ee). Similar results were obtained with the non-aromatic ester substrate **2f**, which provided the corresponding ester derivative **4Af**. Additionally, the phenylethyl group (**2g**) was also incorporated, affording product **4Ag** in very high yield (98%) without affecting the stereoselectivity (91:9 dr). On the other hand, both electron-donating and electron-withdrawing substituents on the aromatic ring of the coumarin core exhibited a similar behavior on the reaction (**4Ah–k** and **4Al–n**, respectively), although the enantioselectivity observed for the nitro-derivative (**4An**) was slightly lower (84% ee).

Subsequently, we investigated the reaction with different trienamine precursors **1A**–**G**, alkyl groups at γ- and δ-positions of the 2,4-dienal substrates (**1A**–**D**) afforded to the corresponding products **4Aa**–**Da** in good-to-excellent yields (86–97%) and with high stereoselectivities (83:17–92:8 dr, 96–99% ee). An additional fused cyclohexane ring was also successfully incorporated using the 2,4-dienal **1E**, providing the corresponding product **4Ea** with high enantiocontrol. Furthermore, the in situ-generated *ortho*-quinodimethanes from the 2-methylindole acrylaldehydes **1F,G** furnished the corresponding spiro-methyleneindole-bridged derivatives **4Fa,Ga** in moderate-to-good yields and stereoselectivities.

Finally, the combination of different 2,4-dienals **1** and coumarin-3-carboxamides **2** demonstrated the same tendency toward the formation of the desired products (**4Bb,d,e,h** and **4Fb,d,e**), thereby confirming the broad scope of the methodology.

**Figure 1:**
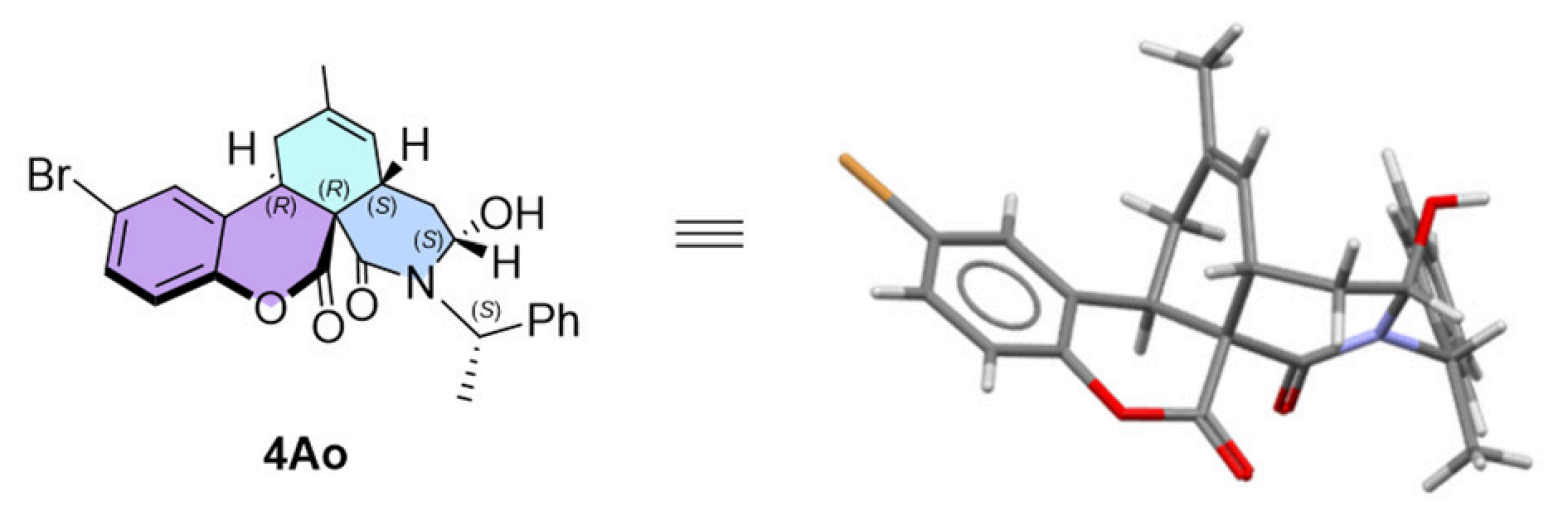
X-ray structure of compound **4Ao**.

The absolute configuration of the newly formed stereocenters in compound **4Ao** was unambiguously established by single-crystal X-ray diffraction as (3a*S*,5*S*,7a*R*,13b*R*) (Figure 1). The absolute configuration of the remaining products was assigned by analogy, based on their formation under same catalytic conditions with catalyst **3b**, their comparable stereochemical outcomes and the assumption that the DA/NRC sequence proceeds through the same favored trienamine transition state.

**Scheme 3:**
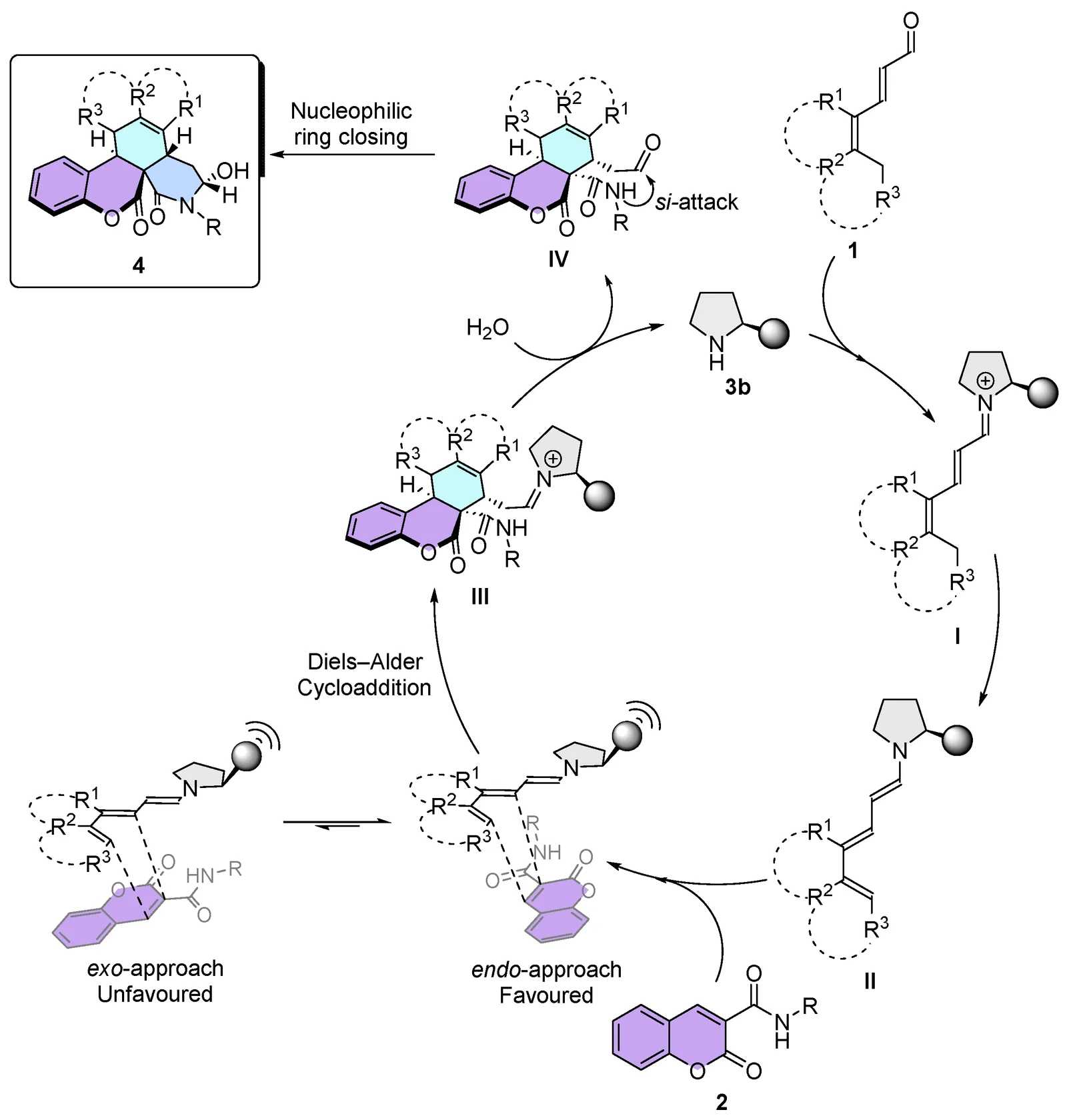
Catalytic cycle and proposed mechanism for the DA/NRC process.

According to the observed absolute configuration, a plausible mechanism for the DA/NRC sequence was proposed (Scheme 3). Initially, the condensation of the catalyst with the 2,4-dienal **1** leads to the formation of the trienamine species **II**, which undergoes a Diels–Alder cycloaddition in the presence of coumarin-3-carboxamide **2**. The approach of the coumarin to the trienamine double bonds occurs from the opposite face to the bulky -C(Ph)_2_OSi(Ph)_3_ substituent of the catalyst, and in an *endo* fashion with respect to the amide moiety. Subsequently, the resulting iminium ion **III** undergoes hydrolysis, regenerating the catalyst and furnishing the corresponding aldehyde **IV**. Finally, an intramolecular nucleophilic addition of the amide nitrogen to the *si*-face of the aldehyde carbonyl group affords to the desired product **4**.

**Scheme 4:**
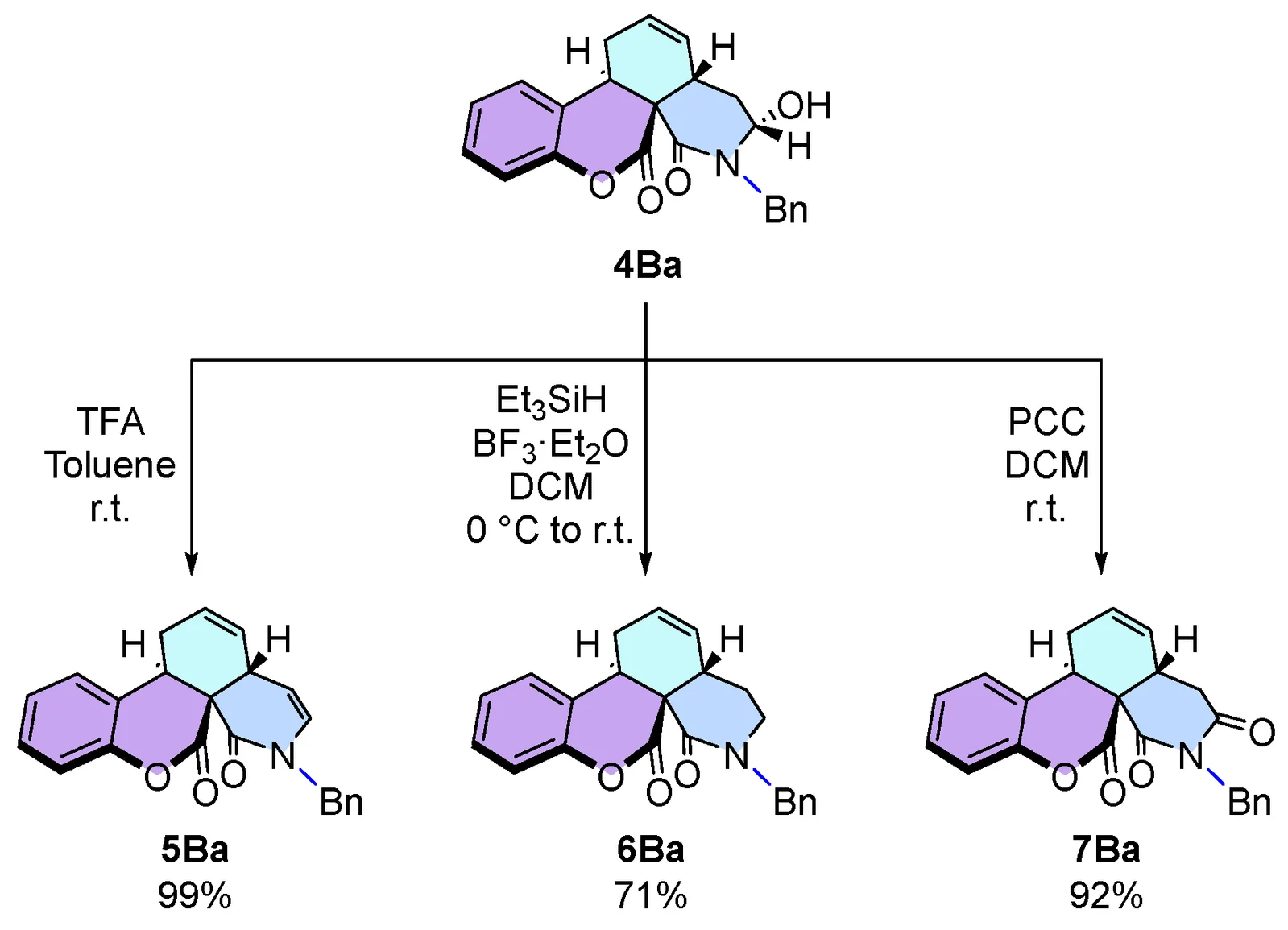
Synthetic transformations of **4Ba**.

In order to demonstrate the synthetic utility of the new spiro-bridged 3,4-dihydrocoumarins, the compound **4Ba** was transformed into the corresponding derivatives **5Ba**, **6Ba** and **7Ba**, through elimination, selective reduction [60] and oxidation reactions [60], respectively (Scheme 4). These transformations provide access to new spiro-bridged 3,4-dihydrocoumarin-dihydropyridinone, -piperidone and -glutarimide privileged scaffolds.

## 3. Materials and Methods

### 3.1. General Information

General procedures and spectroscopic data of catalyst **3b**, aldehydes **1** [68,69], and coumarin-3-carboxamides **2** are described in the Supporting Information.

Starting materials and reagents used in the project are commercially available unless synthesis is described. The solvents were distilled and dried according to standard procedures before use. Unless stated otherwise, all reactions were performed without an inert atmosphere in oven- and/or flame-dried glassware. Reaction progress was monitored by TLC (silica gel plates with F-254 indicator, Darmstadt, Germany), and the spots were visualized under UV light (254 or 365 nm). Flash column chromatography was performed using silica gel Kieselgel 60 (70–230 mesh, Düren, Germany) as the stationary phase and a mixture of ethyl acetate and hexanes as eluent.

Spectral data of the known compounds were in accordance with the literature data. ^1^H and ^13^C NMR spectra were acquired on spectrometers Bruker Ascend ^TM^ (500 or 400 MHz for ^1^H and 125 or 101 MHz for ^13^C, Bremen, Germany) in deuterated CDCl_3_ or DMSO-*d*_6_. Data is reported in the following order: chemical shift (δ) in ppm; multiplicities *s* (singlet), *d* (doublet), *t* (triplet), *q* (quartet), *quint* (quintet), *dd* (doublet of doublets), *dt* (doublet of triplets), *dq* (doublet of quartets), *td* (triplet of doublets), *ddd* (doublet of doublets of doublets) *br* (broad signal), *br d* (broad doublet signal), *m* (multiplet); coupling constants *J* (Hz) and integration.

Melting points were determined using an RY-2 melting point tester Hynotek (Ningbo, China).

For HRMS, samples were analyzed in positive mode by ESI-QTOF-MS using a maXis Impact from Bruker Daltonics (Billerica, MA, USA). HPLC chromatography was performed on a Shimadzu i-series HPLC System (Shimadzu Corporation, Kyoto, Japan) using *n*-hexane/isopropanol as the mobile phase. Enantiomeric purities of the reaction products were determined with Daicel Chiralcel OD-H, AD-H, and OJ-H (particle size of 5 μm and column size of 250 × 4.6 mm). The column temperature was 25 °C and UV absorption was measured at 210 nm. Optical rotations were measured on a Bellingham Stanley polarimeter with ${\left[\alpha \right]}_{D}^{22}$ values reported in degrees with concentrations reported in g/100 mL.

SC-XRD data for **4Ao** were performed in a SuperNova, single-source at offset/far, EosS2 diffractometer (Yarnton, Oxford Shire, UK) using Mo Kα radiation (λ = 0.71073). The crystals were kept at 293 (2) K during data collection. Using Olex2 v1.5 [70], the structures were solved with the SHELXT (2018-2) [71] structure solution program using Intrinsic Phasing and refined with the SHELXL (2019-1) [67] (refinement package using Least Squares minimization.

### 3.2. Experimental Procedure for Compounds ***4***

To a stirring solution of aminocatalyst **3b** (6.2 mg, 0.01212 mmol) and benzoic acid (1.5 mg, 0.01212 mmol, 0.2 equiv.) in dry toluene (0.7 mL), the 2,4-dienal **1** (10 mg, 0.0909 mmol, 1.5 equiv.) and dienophile **2** (0.0606 mmol, 1 equiv.) were subsequently added. The resulting solution was stirred at ambient temperature until the completion of reaction, as monitored by TLC. The reaction mixture was concentrated in vacuo to give a residue. The crude product was purified by flash column chromatography to afford the corresponding product **4**.

### 3.3. Characterization Data of Products ***4***

**(3a*S*,5*S*,7a*R*,13b*R*)-6-benzyl-5-hydroxy-2-methyl-1,3a,4,5,6,13b-hexahydro-7*H*,8*H*-chromeno [4,3-*i*]isoquinoline-7,8-dione (4Aa)**: The reaction was carried out following the general procedure to furnish; after 36 h, the crude product was a 94:6 mixture of diastereoisomers; dr was determined by the integration of ^1^H-NMR signal: *δ_major_* 5.17 ppm (*m*), *δ_minor_* 5.27 ppm (*m*). The crude product was purified by flash column chromatography with hexane-EtOAc (80:20) to afford product **4Aa** (22.1 mg, 95% yield, 96.5% ee) as a white crystalline solid. The ee value was determined by HPLC analysis on a Daicel Chiralpak OD-H column: 90:10 hexane/*i*-PrOH, flow rate 0.5 mL/min, λ = 210 nm: *τ_major_* = 26.2 min, *τ_minor_* = 32.4 min. [α]_22_^D^ = −142.7 (*c* = 0.5, acetone). **^1^H NMR** (500 MHz, CDCl_3_) δ 7.44 (*d*, *J* = 7.6 Hz, 2H), 7.36 (*t*, *J* = 7.7 Hz, 2H), 7.29 (*m*, 2H), 7.16 (*m*, 2H), 7.07 (*d*, *J* = 8.3 Hz, 1H), 5.42 (*m*, 2H), 4.82 (*dq*, *J* = 12.1, 1.6 Hz, 1H), 4.32 (*m*, 2H), 2.58 (*m*, 3H), 2.45 (*m*, 1H), 2.32 (*dt*, *J* = 14.8, 4.4 Hz, 1H), 2.04 (*m*, 1H), 1.85 (*s*, 3H). **^13^C NMR** (126 MHz, CDCl_3_) δ 167.76, 166.41, 150.90, 136.76, 135.96, 128.72, 128.55, 127.94, 127.42, 125.68, 125.22, 123.71, 122.41, 116.57, 79.10, 51.54, 47.72, 34.93, 31.20, 30.34, 26.93, 24.07 **HRMS** (ESI+) *m*/*z* calcd. for C_24_H_23_NO_4_ [M + H]^+^ 390.1700 found 390.1710; **m.p.** 90–93 °C (170–180 °C decomp.).

**(3a*S*,5*S*,7a*R*,13b*R*)-5-hydroxy-6-(3-methoxyphenethyl)-2-methyl-1,3a,4,5,6,13b-hexahydro-7*H*,8*H*-chromeno [4,3-*i*]isoquinoline-7,8-dione (4Ab)**: The reaction was carried out following the general procedure to furnish; after 36 h, the crude product was a 88:12 mixture of diastereoisomers; dr was determined by the integration of ^1^H-NMR signal: *δ_major_* 3.57 ppm (*m*), *δ_minor_* 4.16 ppm (*m*). The crude product was purified by flash column chromatography with 20% EtOAc-hexane to afford product **4Ab** (23.3 mg, 90% yield, 92% ee) as a white crystalline solid. The ee value was determined by **HPLC** analysis on a Daicel Chiralpak OD-H column: 90:10 hexane/*i*-PrOH, flow rate 0.5 mL/min, λ = 210 nm: *τ_major_* = 30.0 min, *τ_minor_* = 25.1 min. [α]_22_^D^ = −109.3 (*c* = 0.5, acetone). **^1^H NMR** (500 MHz, CDCl_3_) δ 7.28 (*m*, 2H), 7.17 (*m*, 2H), 7.04 (*m*, 2H), 6.76 (*dd*, *J* = 8.6, 2.8 Hz, 1H), 6.69 (*d*, *J* = 2.7 Hz, 1H), 5.21 (*br*, 1H), 4.84 (*ddd*, *J* = 12.6, 4.7, 3.6 Hz, 1H), 4.51 (*dd*, *J* = 11.8, 4.4 Hz, 1H), 4.26 (*m*, 1H), 3.80 (*s*, 3H), 3.10 (*ddd*, *J* = 15.7, 11.0, 4.7 Hz, 1H), 2.93 (*m*, 1H), 2.85 (*m*, 1H), 2.74 (*dt*, *J* = 15.7, 3.5 Hz, 1H), 2.58 (*br d*, *J* = 18.3 Hz, 1H), 2.42 (*m*, 2H), 2.24 (*dt*, *J* = 13.8, 4.1 Hz, 1H), 2.06 (*m*, 1H), 1.82 (s, 3H). **^13^C NMR** (101 MHz, CDCl_3_) δ 168.26, 165.89, 158.33, 151.10, 136.80, 135.40, 128.97, 128.39, 125.87, 125.67, 125.08, 124.44, 120.63, 116.55, 113.49, 113.06, 55.45, 53.01, 51.35, 40.58, 34.90, 31.90, 30.61, 29.49, 26.89, 24.11 **HRMS** (ESI+) *m*/*z* calcd. for C_26_H_27_NO_5_ [M + H]^+^ 434.1962 found 434.1987; **m.p.** 78–80 °C (160–170 °C decomp.).

**(3a*S*,5*S*,7a*R*,13b*R*)-5-hydroxy-2-methyl-6-(2-(naphthalen-1-yl)ethyl)-1,3a,4,5,6,13b-hexahydro-7*H*,8*H*-chromeno [4,3-*i*]isoquinoline-7,8-dione (4Ac)**: The reaction was carried out following the general procedure to furnish; after 36 h, the crude product was a 86:14 mixture of diastereoisomers; dr was determined by the integration of ^1^H-NMR signal: *δ_major_* 1.70 ppm (*m*), *δ_minor_* 1.68 ppm (*m*). The crude product was purified by flash column chromatography with 20% EtOAc-hexane to afford product **4Ac** (20.7 mg, 76% yield, 90.1% ee) as a pale crystalline solid. The ee value was determined by **HPLC** analysis on a Daicel Chiralpak OD-H column: 90:10 hexane/*i*-PrOH, flow rate 0.5 mL/min, λ = 210 nm: *τ_major_* = 60.3 min, *τ_minor_* = 68.6 min. [α]_22_^D^ = −231.4 (*c* = 0.5, acetone). **^1^H NMR** (400 MHz, CDCl_3_) δ 8.31 (*d*, *J* = 8.4 Hz, 1H), 7.85 (*m*, 1H), 7.74 (*dd*, *J* = 8.8, 4.1 Hz, 1H), 7.56 (*m*, 1H), 7.52 (*m*, 1H), 7.40 (*m*, 2H), 7.29 (*m*, 1H), 7.17 (*m* 2H), 7.08 (*dd*, *J* = 7.8, 1.1 Hz, 1H), 5.36 (*q*, *J* = 2.0 Hz, 1H), 5.1 (*br*, 1H), 4.63 (*m*, 1H), 4.37 (*dd*, *J* = 4.8, 2.3 Hz, 1H), 3.99 (*ddd*, *J* = 13.7, 9.3, 4.9 Hz, 1H), 3.86 (*m*, 1H), 3.74 (*ddd*, *J* = 13.2, 8.9, 7.3 Hz, 1H), 3.54 (*ddd*, *J* = 13.6, 8.8, 4.8 Hz, 1H), 3.43 (*m*, 1H), 2.61 (*m*, 1H), 2.50 (*m*, 1H), 2.32 (*dt*, *J* = 14.6, 4.3 Hz, 1H), 1.96 (*m*, 1H), 1.83 (*s*, 3H). **^13^C NMR** (101 MHz, CDCl_3_) δ 167.81, 166.14, 150.99, 136.29, 135.33, 134.01, 132.15, 128.83, 128.64, 127.52, 127.41, 126.46, 125.78, 125.72, 125.59, 125.28, 124.16, 122.49, 116.67, 81.47, 51.48, 48.25, 35.04, 31.64, 30.42, 26.96, 24.17 **HRMS** (ESI+) *m*/*z* calcd. for C_29_H_27_NO_4_ [M + H]^+^ 454.2013 found 454.1999; **m.p.** 88–90 °C (190 °C decomp.).

**(3a*S*,5*S*,7a*R*,13b*R*)-5-hydroxy-2-methyl-6-(2-(thiophen-3-yl)ethyl)-1,3a,4,5,6,13b-hexahydro-7*H*,8*H*-chromeno [4,3-*i*]isoquinoline-7,8-dione (4Ad)**: The reaction was carried out following the general procedure to furnish; after 36 h, the crude product was a 91:9 mixture of diastereoisomers; dr was determined by the integration of ^1^H-NMR signal: *δ_major_* 4.63 ppm (*m*), *δ_minor_* 4.78 ppm (m). The crude product was purified by flash column chromatography with 20% EtOAc-hexane to afford product **4Ad** (20.6 mg, 84% yield, 95.2% ee) as a colorless crystalline solid. The ee value was determined by **HPLC** analysis on a Daicel Chiralpak OD-H column: 90:10 hexane/*i*-PrOH, flow rate 0.5 mL/min, λ = 210 nm: *τ_major_* = 16.7 min, *τ_minor_* = 14.4 min. [α]_22_^D^ = −208.26 (*c* = 0.5, acetone). **^1^H NMR** (500 MHz, CDCl_3_) 7.28 (*td*, *J* = 7.8, 2.6 Hz, 1H), 7.15 (*m*, 3H), 7.06 (*d*, *J* = 6.7 Hz, 1H), 7.00 (*d*, *J* = 3.4 Hz, 1H), 6.93 (*dd*, *J* = 5.1, 3.4 Hz, 1H), 5.35 (*q*, *J* = 2.0 Hz, 1H), 4.60 (*br*, 1H), 4.28 (*dd, J* = 5.0, 2.3 Hz, 1H), 3.93 (*ddd*, *J* = 13.0, 7.9, 4.8 Hz, 1H), 3.65 (*dt*, *J* = 13.3, 7.8 Hz, 1H), 3.30 (*dt*, *J* = 15.4, 7.8 Hz, 1H), 3.19 (*ddd*, *J* = 13.6, 7.4, 4.3 Hz, 1H), 2.56 (*m*, 1H), 2.50 (*br*, 1H), 2.39 (*dq*, *J* = 18.4, 4.2 Hz, 1H), 2.30 (*dt*, *J* = 14.6, 4.4 Hz, 1H), 1.96 (*dt*, *J* = 14.5, 2.6 Hz, 1H), 1.82 (*s*, 3H). **^13^C NMR** (101 MHz, CDCl_3_) δ 166.98, 165.41, 151.23, 140.68, 131.92, 130.28, 128.62, 127.12, 126.11, 125.96, 125.08, 124.88, 124.71, 124.07, 119.28, 118.27, 116.63, 85.13, 49.54, 33.21, 32.63, 28.74, 23.59 **HRMS** (ESI+) *m*/*z* calcd. for C_23_H_23_NO_4_S [M + H]^+^ 410.1421 found 410.1447; **m.p.** 90–96 °C (150 °C decomp.).

**(3a*S*,5*S*,7a*R*,13b*R*)-6-(2-(1*H*-indol-3-yl)ethyl)-5-hydroxy-2-methyl-1,3a,4,5,6,13b-hexahydro-7*H*,8*H*-chromeno [4,3-*i*]isoquinoline-7,8-dione (4Ae)**: The reaction was carried out following the general procedure to furnish; after 36 h, the crude product was a 90:10 mixture of diastereoisomers; dr was determined by the integration of ^1^H-NMR signal: *δ_major_* 4.63 ppm (*m*), *δ_minor_* 4.73 ppm (*m*). The crude product was purified by flash column chromatography with 20% EtOAc-hexane to afford product **4Ae** (23.1 mg, 87% yield, >99% ee) as a pale crystalline solid. The ee value was determined by **HPLC** analysis on a Daicel Chiralpak OD-H column: 80:20 hexane/*i*-PrOH, flow rate 0.5 mL/min, λ = 210 nm: *τ_major_* = 28.5 min, *τ_minor_* = 25.4 min. [α]_22_^D^ = −10.06 (*c* = 0.5, acetone). **^1^H NMR** (400 MHz, CDCl_3_) δ 7.99 (*s*, 1H), 7.74 (*d*, *J* = 7.7 Hz, 1H), 7.37 (*d*, *J* = 8.0 Hz, 1H), 7.27 (*m*, 2H), 7.16 (*m*, 4H), 7.07 (*d*, *J* = 8.0 Hz, 1H), 5.34 (*s*, 1H), 7.59 (*m*, 1H), 4.32 (*d*, *J* = 3.1 Hz, 1H), 4.01 (*ddd*, *J* = 13.0, 8.1, 4.7 Hz, 1H), 3.67 (*dt*, *J* = 13.2, 8.0 Hz, 1H), 3.25 (*m*, 1H), 3.13 (*ddd*, *J* = 13.6, 7.7, 4.6 Hz, 1H), 2.58 (*br d*, *J* = 18.3 Hz, 1H), 2.45 (*m*, 3H), 2.26 (*dt*, *J* = 14.6, 4.4 Hz, 1H), 1.92 (*dt*, *J* = 14.6, 2.2 Hz, 1H), 1.80 (*s*, 3H). **^13^C NMR** (101 MHz, CDCl_3_) δ 165.90, 159.64, 151.00, 136.43, 136.13, 128.60, 125.75, 125.26, 123.81, 123.00, 122.57, 122.14, 119.63, 119.11, 116.64, 113.20, 111.22, 81.33, 51.43, 47.46, 35.00, 31.21, 30.43, 26.93, 24.13, 23.67 **HRMS** (ESI+) *m*/*z* calcd. for C_27_H_26_N_2_O_4_ [M + H]^+^ 443.1965 found 443.1988; **m.p.** 107–115 °C (210 °C decomp.).

**Ethyl-3-((3a*R*,5*S*,7a*S*,13b*S*)-5-hydroxy-2-methyl-7,8-dioxo-1,3a,4,13b-tetrahydro-5*H*,8*H*-chromeno [4,3-*i*]isoquinolin-6(7*H*)-yl)propanoate (4Af)**: The reaction was carried out following the general procedure to furnish; after 36 h, the crude product was a 80:20 mixture of diastereoisomers; dr was determined by the integration of ^1^H-NMR signal: *δ_major_* 4.21 ppm (*d*), *δ_minor_* 4.03 ppm (*d*). The crude product was purified by flash column chromatography with 20% EtOAc-hexane to afford product **4Af** (21.5 mg, 90% yield, 69% ee) as a white crystalline solid. The ee value was determined by **HPLC** analysis on a Daicel Chiralpak OD-H column: 90:10 hexane/*i*-PrOH, flow rate 0.5 mL/min, λ = 210 nm: *τ_major_* = 21.2 min, *τ_minor_* = 26.6 min. [α]_22_^D^ = −224.04 (*c* = 0.5, acetone). **^1^H NMR** (400 MHz, CDCl_3_) δ 7.37 (*m*, 1H), 7.14 (*m*, 2H), 7.04 (*dd*, *J* = 7.8, 1.0 Hz, 1H), 5.35 (*s*, 1H), 5.05 (*dd*, *J* = 4.3, 1.9 Hz, 1H), 4.21 (*m*, 1H), 4.14 (*qd*, *J* = 7.2, 1.4 Hz, 2H), 3.89 (*dt*, *J* = 13.7, 6.9 Hz, 1H), 3.67 (*dt*, *J* = 13.5, 6.5 Hz, 1H), 3.48 (*br*, 1H), 2.78 (*t*, *J* = 6.7 Hz, 2H), 2.52 (*m*, 2H), 2.39 (*m*, 1H), 2.32 (*dt*, *J* = 14.6, 4.4 Hz, 1H), 2.01 (*m*, 1H), 1.80 (*s*, 3H), 1.26 (*t*, *J* = 7.2 Hz, 3H). **^13^C NMR** (101 MHz, CDCl_3_) δ 172.79, 167.76, 166.43, 150.86, 135.24, 128.54, 128.27, 125.72, 125.20, 123.82, 122.38, 116.54, 81.69, 60.88, 51.35, 43.46, 34.77, 32.85, 31.44, 30.16, 24.04, 14.28 **HRMS** (ESI+) *m*/*z* calcd. for C_22_H_25_NO_6_ [M + H]^+^ 400.1755 found 400.1745; **m.p.** 110–119 °C (200 °C decomp.).

**(3a*S*,5*S*,7a*R*,13b*R*)-5-hydroxy-2-methyl-6-((*S*)-1-phenylethyl)-1,3a,4,5,6,13b-hexahydro-7*H*,8*H*-chromeno [4,3-*i*]isoquinoline-7,8-dione (4Ag)**: The reaction was carried out following the general procedure to furnish; after 36 h, the crude product was a 91:9 mixture of diastereoisomers; dr was determined by the integration of ^1^H-NMR signal: *δ_major_* 4.71 ppm (*s*), *δ_minor_* 5.18 ppm (*s*). The crude product was purified by flash column chromatography with 20% EtOAc-hexane to afford product **4Ag** (23.7 mg, 98% yield) as a pale crystalline solid. [α]_22_^D^ = −1688.96 (*c* =1, acetone). **^1^H NMR** (400 MHz, CDCl_3_) δ 7.51 (*m*, 2H), 7.38 (*t*, *J* = 7.6 Hz, 2H), 7.30 (*m*, 2H), 7.15 (*m*, 2H), 7.06 (*d*, *J* = 7.5 Hz, 1H), 5.97 (*q*, *J* = 7.1 Hz, 1H), 5.39 (*q*, *J* = 2.1 Hz, 1H), 4.65 (*br*, 1H), 4.35 (*dd*, *J* = 4.7, 2.3 Hz, 1H), 2.72 (*br d*, *J* = 11.5 Hz, 1H), 2.60 (*br d*, *J* = 18.4, 1.9 Hz, 1H), 2.50 (*br*, 1H), 2.41 (*m*, 1H), 2.17 (*dt*, *J* = 14.6, 4.3 Hz, 1H), 1.91 (*dt*, *J* = 14.5, 2.7 Hz, 1H), 1.85 (*s*, 3H), 1.73 (*d*, *J* = 7.1 Hz, 3H). **^13^C NMR** (101 MHz, CDCl_3_) δ 167.79, 166.20, 151.00, 139.83, 136.21, 128.66, 128.57, 128.18, 127.67, 125.68, 125.22, 123.72, 123.05, 116.65, 78.00, 53.69, 51.87, 35.34, 31.77, 30.44, 26.91, 24.19, 18.13 **HRMS** (ESI+) *m*/*z* calcd. for C_25_H_25_NO_4_ [M + H]^+^ 404.1856 found 404.1875; **m.p.** 131–133 °C (220 °C decomp.).

**(3a*S*,5*S*,7a*R*,13b*R*)-6-benzyl-5-hydroxy-2,12-dimethyl-1,3a,4,5,6,13b-hexahydro-7*H*,8*H*-chromeno [4,3-*i*]isoquinoline-7,8-dione (4Ah)**: The reaction was carried out following the general procedure to furnish; after 36 h, the crude product was a 90:10 mixture of diastereoisomers; dr was determined by the integration of ^1^H-NMR signal: *δ_major_* 1.87 ppm (*s*), *δ_minor_* 1.73 ppm (*s*). The crude product was purified by flash column chromatography with 20% EtOAc-hexane to afford product **4Ah** (22.7 mg, 94% yield, 99.4% ee) as a white crystalline solid. The ee value was determined by **HPLC** analysis on a Daicel Chiralpak OD-H column: 90:10 hexane/*i*-PrOH, flow rate 1 mL/min, λ = 210 nm: *τ_major_* = 24.6 min, *τ_minor_* = 28.6 min. [α]_22_^D^ = −191.62 (*c* = 0.5, acetone). **^1^H NMR** (500 MHz, CDCl_3_) δ 7.44 (*d*, *J* = 7.2 Hz, 2H), 7.36 (*t*, *J* = 7.6 Hz, 2H), 7.28 (*d*, *J* = 7.2 Hz, 1H), 7.07 (*m*, 1H), 6.95 (*d*, *J* = 8.2 Hz, 1H), 6.92 (*s*, 1H), 5.41 (*m*, 2H), 4.82 (*dd*, *J* = 4.4, 1.8 Hz, 1H), 4.31 (*m*, 2H), 2.58 (*m*, 2H), 2.42 (*m*, 1H), 2.34 (*m*, 4H), 2.31 (*m*, 1H), 2.03 (*m*, 1H), 1.85 (*s*, 3H). **^13^C NMR** (126 MHz, CDCl_3_) δ 167.88, 166.49, 148.90, 136.85, 136.33, 134.81, 133.73, 130.30, 129.07, 128.79, 128.60, 128.06, 127.49, 126.08, 123.31, 122.52, 116.39, 79.27, 51.65, 47.78, 34.98, 31.26, 30.42, 26.94, 24.20, 21.30 **HRMS** (ESI+) *m*/*z* calcd. for C_25_H_25_NO_4_ [M + H]^+^ 404.1856 found 404.1884; **m.p.** 93–95 °C (180 °C decomp.).

**(3a*S*,5*S*,7a*R*,13b*R*)-6-benzyl-5-hydroxy-12-methoxy-2-methyl-1,3a,4,5,6,13b-hexahydro-7*H*,8*H*-chromeno [4,3-*i*]isoquinoline-7,8-dione (4Ai)**: The reaction was carried out following the general procedure to furnish; after 36 h, the crude product was a 92:8 mixture of diastereoisomers; dr was determined by the integration of ^1^H-NMR signal: *δ_major_* 1.87 ppm (*s*), *δ_minor_* 1.74 ppm (*s*). The crude product was purified by flash column chromatography with 20% EtOAc-hexane to afford product **4Ai** (23.1 mg, 92% yield, >99% ee) as a white crystalline solid. The ee value was determined by **HPLC** analysis on a Daicel Chiralpak OD-H column: 90:10 hexane/*i*-PrOH, flow rate 1 mL/min, λ = 210 nm: *τ_major_* = 40.2 min, *τ_minor_* = 36.9 min. [α]_22_^D^ = −218.5 (*c* = 0.5, acetone). **^1^H NMR** (500 MHz, CDCl_3_) 7.44 (*d*, *J* = 7.1 Hz, 2H), 7.36 (*t*, *J* = 7.7 Hz, 2H), 7.28 (*d*, *J* = 7.2 Hz, 1H), 7.00 (*d*, *J* = 8.8 Hz, 1H), 6.79 (*ddd*, J = 8.9, 2.9, 1.0 Hz, 1H), 6.67 (*dd*, *J* = 2.9, 1.3 Hz, 1H), 5.40 (*m*, 2H), 4.82 (*m*, 1H), 4.32 (*d*, *J* = 15.1 Hz, 2H), 3.79 (*s*, 3H), 2.53 (*m*, 3H), 2.44 (*m*, 1H), 2.33 (*dt*, *J* = 14.7, 4.4 Hz, 1H), 2.03 (*m*, 1H), 1.85 (*s*, 3H). **^13^C NMR** (126 MHz, CDCl_3_) δ 167.83, 166.44, 156.92, 144.85, 136.82, 136.28, 130.32, 128.81, 128.61, 128.07, 127.51, 124.98, 122.56, 117.31, 112.49, 111.98, 79.27, 55.77, 51.54, 47.79, 35.20, 31.21, 30.41, 27.05, 24.21 **HRMS** (ESI+) *m*/*z* calcd. for C_25_H_25_NO_5_ [M + H]^+^ 420.1805 found 420.1850; **m.p.** 122–124 °C (205 °C decomp.).

**(3a*S*,5*S*,7a*R*,13b*R*)-6-benzyl-10-ethoxy-5-hydroxy-2-methyl-1,3a,4,5,6,13b-hexahydro-7*H*,8*H*-chromeno [4,3-*i*]isoquinoline-7,8-dione (4Aj)**: The reaction was carried out following the general procedure to furnish; after 36 h, the crude product was a 87:13 mixture of diastereoisomers; dr was determined by the integration of ^1^H-NMR signal: *δ_major_* 4.81 ppm (*s*), *δ_minor_* 5.01 ppm (*s*). The crude product was purified by flash column chromatography with 20% EtOAc-hexane to afford product **4Aj** (23.1 mg, 89% yield, 87% ee) as a brown crystalline solid. The ee value was determined by **HPLC** analysis on a Daicel Chiralpak OD-H column: 90:10 hexane/*i*-PrOH, flow rate 1 mL/min, λ = 210 nm: *τ_major_* = 44.5 min, *τ_minor_* = 34.7 min. [α]_22_^D^ = -267.2 (*c* = 0.5, acetone): **^1^H NMR** (500 MHz, CDCl_3_) δ 7.45 (*d*, *J* = 7.6 Hz, 2H), 7.35 (*t*, *J* = 7.6 Hz, 2H), 7.28 (*d*, *J* = 7.2 Hz, 1H), 7.07 (*t*, *J* = 8.0 Hz, 1H), 6.88 (*d*, *J* = 8.2 Hz, 1H), 6.72 (*d*, *J* = 7.8 Hz, 1H), 5.40 (*m*, 2H), 4.82 (*s*, 1H), 4.33 (*m*, 2H), 4.09 *(qd*, *J* = 7.0, 1.8 Hz, 2H), 2.60 (*m*, 2H), 2.55 (*s*, 1H), 2.42 (*m*, 1H), 2.34 (*dt*, *J* = 14.7, 4.4 Hz, 1H), 2.03 (*dt*, *J* = 14.6, 2.6 Hz, 1H), 1.83 (*s*, 3H), 1.45 (*t*, *J* = 7.0 Hz, 3H). **^13^C NMR** (126 MHz, CDCl_3_) δ 167.25, 166.47, 146.63, 140.32, 136.85, 136.57, 128.78, 128.02, 127.46, 125.06, 124.89, 122.44, 116.91, 112.88, 79.34, 65.00, 47.83, 35.22, 31.27, 30.33, 27.16, 24.20, 14.92 **HRMS** (ESI+) *m*/*z* calcd. for C_26_H_27_NO_5_ [M + H]^+^ 434.1962 found 434.2008; **m.p.** 115–118 °C (200 °C decomp.).

**(3a*S*,5*S*,7a*R*,13b*R*)-6-benzyl-11-(diethylamino)-5-hydroxy-2-methyl-1,3a,4,5,6,13b-hexahydro-7*H*,8*H*-chromeno [4,3-*i*]isoquinoline-7,8-dione (4Ak)**: The reaction was carried out following the general procedure to furnish; after 36 h, the crude product was a 88:12 mixture of diastereoisomers; dr was determined by the integration of ^1^H-NMR signal: *δ_major_* 4.84 ppm (*m*), *δ_minor_* 4.98 ppm (*m*). The crude product was purified by flash column chromatography with 20% EtOAc-hexane to afford product **4Ak** (19.6 mg, 71% yield, 71% ee) as a pale yellow crystalline solid. The ee value was determined by **HPLC** analysis on a Daicel Chiralpak OD-H column: 90:10 hexane/*i*-PrOH, flow rate 0.5 mL/min, λ = 210 nm: *τ_major_* = 41.1 min, *τ_minor_* = 27.1 min. [α]_22_^D^ = −321.78 (*c* = 0.5, acetone). **^1^H NMR** (500 MHz, CDCl_3_) δ 8.10 (*m*, 1H), 7.40 (*m*, 5H), 6.90 (*d*, *J* = 8.6 Hz, 1H), 6.43 (*dd*, *J* = 8.7, 2.7 Hz, 1H), 6.35 (*d*, *J* = 2.6 Hz, 1H), 5.41 (*m*, 2H), 4.81 (*d*, *J* = 3.9 Hz, 1H), 4.31 (*d*, *J* = 15.0 Hz, 1H), 4.23 (*m*, 1H), 3.32 (*qd*, *J* = 7.2, 2.3 Hz, 4H), 2.54 (*m*, 2H), 2.39 (*m*, 1H), 2.32 (*dt*, *J* = 14.7, 4.4 Hz, 1H), 2.03 (*m*, 1H), 1.83 (*s*, 3H), 1.15 (*t*, *J* = 7.0 Hz, 6H). **^13^C NMR** (101 MHz, CDCl_3_) δ 170.98, 166.69, 152.08, 148.29, 136.98, 130.30, 128.78, 128.61, 128.08, 127.44, 126.06, 122.33, 108.29, 99.50, 79.35, 52.14, 47.74, 44.57, 34.17, 31.28, 30.53, 27.04, 24.19, 12.65 **HRMS** (ESI+) *m*/*z* calcd. for C_28_H_32_N_2_O_4_ [M + H]^+^ 461.2435 found 461.2466; **m.p.** 103–110 °C (190 °C decomp.).

**(3a*S*,5*S*,7a*R*,13b*R*)-6-benzyl-12-chloro-5-hydroxy-2-methyl-1,3a,4,5,6,13b-hexahydro-7*H*,8*H*-chromeno [4,3-*i*]isoquinoline-7,8-dione (4Al)**: The reaction was carried out following the general procedure to furnish; after 36 h, the crude product was a 83:17 mixture of diastereoisomers; dr was determined by the integration of ^1^H-NMR signal: *δ_major_* 1.86 ppm (*s*), *δ_minor_* 1.70 ppm (*s*). The crude product was purified by flash column chromatography with 20% EtOAc-hexane to afford product **4Al** (21.5 mg, 85% yield, 96.1% ee) as a white crystalline solid. The ee value was determined by **HPLC** analysis on a Daicel Chiralpak OD-H column: 90:10 hexane/*i*-PrOH, flow rate 1 mL/min, λ = 210 nm: *τ_major_* = 32 min, *τ_minor_* = 29.6 min. [α]_22_^D^ = −174.2 (*c* = 0.5, acetone). **^1^H NMR** (500 MHz, CDCl_3_) δ 7.42 (*d*, *J* = 7.1 Hz, 2H), 7.36 (*t*, *J* = 7.6 Hz, 2H), 7.28 (*m*, 1H), 7.23 (*m*, 1H), 7.09 (*dd*, *J* = 2.5, 1.3 Hz, 1H), 7.01 (*d*, *J* = 8.6 Hz, 1H), 5.43 (*m*, 1H), 5.39 (*d*, *J* = 15.0 Hz, 1H), 4.83 (*m*, 1H), 4.32 (*m*, 2H), 2.50 (*m*, 3H), 2.31 (*dt*, *J* = 14.7, 4.4 Hz, 1H), 2.05 (*m*, 1H), 1.85 (*s*, 3H), 1.69 (*m*, 1H). **^13^C NMR** (126 MHz, CDCl_3_) δ 167.13, 166.00, 149.49, 136.69, 136.04, 130.56, 128.84, 128.68, 128.07, 127.59, 125.97, 125.74, 122.56, 118.03, 79.15, 51.27, 47.82, 35.11, 31.23, 30.41, 26.98, 24.19 **HRMS** (ESI+) *m*/*z* calcd. for C_24_H_22_ClNO_4_ [M + H]^+^ 424.1310 found 424.1324; **m.p.** 113–120 °C (210 °C decomp.).

**(3a*S*,5*S*,7a*R*,13b*R*)-6-benzyl-12-bromo-5-hydroxy-2-methyl-1,3a,4,5,6,13b-hexahydro-7*H*,8*H*-chromeno [4,3-*i*]isoquinoline-7,8-dione (4Am)**: The reaction was carried out following the general procedure to furnish; after 36 h, the crude product was a >95:5 mixture of diastereoisomers; dr was determined by the integration of ^1^H-NMR signal: *δ_major_* 4.91 ppm (*m*), *δ_minor_* 4.85 ppm (*m*). The crude product was purified by flash column chromatography with 20% EtOAc-hexane to afford product **4Am** (25.5 mg, 91% yield, >99% ee) as a white crystalline solid. The ee value was determined by **HPLC** analysis on a Daicel Chiralpak OD-H column: 90:10 hexane/*i*-PrOH, flow rate 1 mL/min, λ = 210 nm: *τ_major_* = 25.3 min, *τ_minor_* = 22.3 min. [α]_22_^D^ = −156.82 (*c* = 0.5, acetone). **^1^H NMR** (400 MHz, CDCl_3_) δ 7.41 (*m*, 3H), 7.35 (*t*, 2H), 7.28 (*d*, 1H), 7.24 (*m*, 1H), 6.96 (*d*, *J* = 8.6 Hz, 1H), 5.43 (*m*, 1H), 5.38 (*d*, *J* = 15.0 Hz, 1H), 4.83 (*m*, 1H), 4.32 (*m*, 2H), 2.50 (*m*, 4H), 2.31 (*dt*, *J* = 14.8, 4.4 Hz, 1H), 2.05 (*dt*, *J* = 14.7, 2.8 Hz, 1H), 1.86 (*s*, 3H). **^13^C NMR** (101 MHz, CDCl_3_) δ 167.06, 165.97, 150.04, 136.70, 135.92, 131.65, 128.88, 128.82, 128.06, 127.58, 126.18, 122.58, 118.39, 118.06, 79.13, 51.28, 47.81, 35.08, 31.26, 30.42, 27.01, 24.16 **HRMS** (ESI+) *m*/*z* calcd. for C_24_H_22_BrNO_4_ [M + H]^+^ 468.0805 found 468.0769; **m.p.** 117–122 °C (210 °C decomp.).

**(3a*S*,5*S*,7a*R*,13b*R*)-6-benzyl-5-hydroxy-2-methyl-12-nitro-1,3a,4,5,6,13b-hexahydro-7*H*,8*H*-chromeno [4,3-*i*]isoquinoline-7,8-dione (4An)**: The reaction was carried out following the general procedure to furnish; after 36 h, the crude product was a 83:17 mixture of diastereoisomers; dr was determined by the integration of ^1^H-NMR signal: *δ_major_* 5.43 ppm (*s*), *δ_minor_* 5.29 ppm (*s*). The crude product was purified by flash column chromatography with 20% EtOAc-hexane to afford product **4An** (22.9 mg, 88% yield, 84% ee) as a yellow semi-solid, which gradually crystallized upon standing at ambient temperature. The ee value was determined by **HPLC** analysis on a Daicel Chiralpak OD-H column: 90:10 hexane/*i*-PrOH, flow rate 1 mL/min, λ = 210 nm: *τ_major_* = 62.3 min, *τ_minor_* = 44.6 min. [α]_22_^D^= −182.14 (*c* = 0.5, acetone). **^1^H NMR** (400 MHz, CDCl_3_) δ 8.21 (*ddd*, *J* = 8.9, 2.6, 0.9 Hz, 1H), 8.07 (*m*, 1H), 7.40 (*m*, 2H), 7.35 (*t*, *J* = 7.4 Hz, 2H), 7.29 (*m*, 1H), 7.22 (*d*, *J* = 8.9 Hz, 1H), 5.43 (*m*, 1H), 5.38 (*d*, *J* = 15.0 Hz, 1H), 4.84 (*dd*, *J* = 4.4, 2.0 Hz, 1H), 4.34 (*m*, 1H), 4.31 (*d*, 1H), 2.57 (*m*, 4H), 2.29 (*dt*, *J* = 14.8, 4.4 Hz, 1H), 2.06 (*m*, 1H), 1.88 (*s*, 3H). **^13^C NMR** (101 MHz, CDCl_3_) δ 166.20, 165.53, 155.29, 144.96, 136.43, 130.25, 128.85, 128.58, 128.00, 127.67, 125.62, 124.72, 122.40, 122.18, 117.59, 78.89, 50.94, 47.72, 35.11, 31.28, 30.46, 24.15 **HRMS** (ESI+) *m*/*z* calcd. for C_24_H_22_N_2_O_6_ [M + H]^+^ 435.1551 found 435.1554 *m*/*z*.

**(3aS,5S,7aR,13bR)-12-bromo-5-hydroxy-2-methyl-6-((S)-1-phenylethyl)-1,3a,4,5,6,13b-hexahydro-7H,8H-chromeno [4,3-i]isoquinoline-7,8-dione (4Ao)**: The reaction was carried out following the general procedure to furnish; after 36 h, the crude product was a 94:6 mixture of diastereoisomers; dr was determined by the integration of ^1^H-NMR signal: *δ_major_* 5.44 ppm (*m*), *δ_minor_* 5.28 ppm (*m*). The crude product was purified by flash column chromatography with 20% EtOAc-hexane to afford product **4Ao** in 82% yield (23.6 mg) as a colorless crystalline solid. [α]_22_^D^ = −1607.2 (*c* = 1, acetone). **^1^H NMR** (400 MHz, CDCl_3_) δ 7.48 (*m*, 2H), 7.39 (*m*, 3H), 7.30 (*m*, 1H), 7.22 (*m*, 1H), 6.94 (*d*, *J* = 8.6 Hz, 1H), 5.93 (*q*, *J* = 7.1 Hz, 1H), 5.42 (*m*, 1H), 4.62 (*m*, 1H), 4.32 (*m*, 1H), 2.68 (*d*, *J* = 11.8 Hz, 1H), 2.51 (*m*, 2H), 2.42 (*m*, 1H), 2.15 (*dt*, *J* = 14.6, 4.3 Hz, 1H), 1.92 (*dt*, *J* = 14.7, 2.6 Hz, 1H), 1.87 (*s*, 3H), 1.72 (*d*, *J* = 7.2 Hz, 3H). **^13^C NMR** (101 MHz, CDCl_3_) δ 167.10, 165.80, 150.04, 139.69, 135.86, 131.60, 128.85, 128.68, 128.14, 127.72, 126.15, 123.13, 118.36, 118.01, 77.91, 53.80, 51.55, 35.39, 31.75, 30.39, 26.92, 24.19, 18.09 **HRMS** (ESI+) *m*/*z* calcd. for C_25_H_24_BrNO_4_ [M + H]^+^ 482.0961 found 482.0978; **m.p.** 146–150 °C (250 °C decomp.).

**(3a*S*,5*S*,7a*R*,13b*R*)-6-benzyl-5-hydroxy-1,3a,4,5,6,13b-hexahydro-7*H*,8*H*-chromeno [4,3-*i*]isoquinoline-7,8-dione (4Ba)**: The reaction was carried out following the general procedure to furnish; after 36 h, the crude product was a 91:9 mixture of diastereoisomers; dr was determined by the integration of ^1^H-NMR signal: *δ_major_* 4.46 ppm (*d*), *δ_minor_* 4.56 ppm (*d*). The crude product was purified by flash column chromatography with 20% EtOAc-hexane to afford product **4Ba** (19.3 mg, 86% yield, >99% ee) as a colorless crystalline solid. The ee value was determined by **HPLC** analysis on a Daicel Chiralpak OD-H column: 90:10 hexane/*i*-PrOH, flow rate 1 mL/min, λ = 210 nm: *τ_major_* = 86.5 min, *τ_minor_* = 79.9 min. [α]_22_^D^ = −227.92 (*c* = 0.5, acetone). **^1^H NMR** (500 MHz, CDCl_3_) δ 7.45 (*d*, *J* = 7.3 Hz, 2H), 7.37 (*t*, *J* = 7.6 Hz, 2H), 7.29 (*d*, *J* = 7.6 Hz, 2H), 7.23 (*m*, 1H), 7.17 (*m*, 1H), 7.08 (*d*, *J* = 8.1 Hz, 1H), 6.04 (*m*, 1H), 5.73 (*dt*, *J* = 10.2, 2.6 Hz, 1H), 5.43 (*d*, *J* = 15.0 Hz, 1H), 4.85 (*m*, 1H), 4.33 (*m*, 2H), 2.78 (*dd*, *J* = 19.0, 6.0 Hz, 1H), 2.57 (*m*, 2H), 2.49 (*m*, 1H), 2.35 (*dt*, *J* = 14.8, 4.5 Hz, 1H), 2.07 (*dt*, *J* = 14.8, 2.5 Hz, 1H). **^13^C NMR** (126 MHz, CDCl_3_) δ 167.42, 166.32, 151.04, 136.71, 128.94, 128.84, 128.67, 128.09, 128.02, 127.56, 126.27, 125.23, 123.49, 116.67, 79.09, 47.72, 34.40, 31.13, 30.04, 21.95 **HRMS** (ESI+) *m*/*z* calcd. for C_23_H_21_NO_4_ [M + H]^+^ 376.1543 found 376.1521; **m.p.** 86–89 °C (180 °C decomp.).

**(3a*S*,5*S*,7a*R*,13b*R*)-5-hydroxy-6-(3-methoxyphenethyl)-1,3a,4,5,6,13b-hexahydro-7*H*,8*H*-chromeno [4,3-*i*]isoquinoline-7,8-dione (4Bb)**: The reaction was carried out following the general procedure to furnish; after 36 h, the crude product was a 87:13 mixture of diastereoisomers; dr was determined by the integration of ^1^H-NMR signal: *δ_major_* 4.43 ppm (*s*), *δ_minor_* 4.51 ppm (*s*). The crude product was purified by flash column chromatography with 20% EtOAc-hexane to afford product **4Bb** (20.8 mg, 94% yield, 74% ee) as a white crystalline solid. The ee value was determined by **HPLC** analysis on a Daicel Chiralpak OD-H column: 90:10 hexane/*i*-PrOH, flow rate 1 mL/min, λ = 210 nm: *τ_major_* = 37.5 min, *τ_minor_* = 43.3 min. [α]_22_^D^ = −236.54 (*c* = 0.5, acetone). **^1^H NMR** (400 MHz, CDCl_3_) δ 7.29 (*m*, 1H), 7.19 (*m*, 3H), 7.07 (*dd*, *J* = 8.0, 1.3 Hz, 1H), 6.93 (*m*, 2H), 6.77 (*ddd*, *J* = 8.2, 2.6, 1.1 Hz, 1H), 5.98 (*m*, 1H), 5.66 (*dt*, *J* = 10.2, 2.7 Hz, 1H), 4.53 (*br*, 1H), 4.29 (*m*, 1H), 3.96 (*ddd*, *J* = 12.9, 7.7, 4.7 Hz, 1H), 3.82 (*s*, 3H), 3.56 (*dt*, *J* = 13.3, 7.9 Hz, 1H), 3.06 (*dt*, *J* = 13.2, 8.0 Hz, 1H), 2.95 (*ddd*, *J* = 13.0, 7.5, 4.8 Hz, 1H), 2.75 (*dd*, *J* = 19.5, 5.2 Hz, 1H), 2.43 (*m*, 3H), 2.27 (*dt*, *J* = 14.7, 4.4 Hz, 1H), 1.95 (*dt*, *J* = 14.9, 2.6 Hz, 1H). **^13^C NMR** (101 MHz, CDCl3) δ 167.44, 165.94, 159.92, 151.06, 141.13, 129.63, 128.99, 128.65, 127.85, 126.30, 125.19, 123.51, 121.56, 116.61, 114.49, 112.55, 81.25, 55.42, 51.75, 48.66, 34.38, 34.36, 31.13, 29.97, 21.94 **HRMS** (ESI+) *m*/*z* calcd. for C_25_H_25_NO_5_ [M + H]^+^ 420.1805 found 420.1801; **m.p.** 83–92 °C (180 °C decomp.).

**(3a*S*,5*S*,7a*R*,13b*R*)-5-hydroxy-6-(2-(thiophen-3-yl)ethyl)-1,3a,4,5,6,13b-hexahydro-7*H*,8*H*-chromeno [4,3-*i*]isoquinoline-7,8-dione (4Bd)**: The reaction was carried out following the general procedure to furnish; after 36 h, the crude product was a 83:17 mixture of diastereoisomers; dr was determined by the integration of ^1^H-NMR signal: *δ_major_* 5.67 ppm (*m*), *δ_minor_* 5.49 ppm (*m*). The crude product was purified by flash column chromatography with 20% EtOAc-hexane to afford product **4Bd** (22.5 mg, 95% yield, 98.6% ee) as a colorless crystalline solid. The ee value was determined by **HPLC** analysis on a Daicel Chiralpak OD-H column: 90:10 hexane/*i*-PrOH, flow rate 1 mL/min, λ = 210 nm: *τ_major_* = 31.8 min, *τ_minor_* = 41.0 min. [α]_22_^D^ = −197.44 (*c* = 0.5, acetone). **^1^H NMR** (400 MHz, CDCl_3_) δ 7.29 (*m*, 1H), 7.22 (*dt*, *J* = 7.8, 1.6 Hz, 1H), 7.16 (*m*, 2H), 7.07 (*dd*, *J* = 8.0, 1.2 Hz, 1H), 7.01 (*dd*, *J* = 3.4, 1.1 Hz, 1H), 6.94 (*dd*, *J* = 5.1, 3.4 Hz, 1H), 5.99 (*m*, 1H), 5.68 (*dt*, *J* = 10.2, 2.7 Hz, 1H), 4.64 (*dd*, *J* = 11.2, 4.65 Hz, 1H) 4.28 (*d*, *J* = 4.6 Hz, 1H), 3.94 (*ddd*, *J* = 13.1, 8.0, 4.9 Hz, 1H), 3.64 (*dt*, *J* = 13.2, 7.8 Hz, 1H), 3.32 (*dt*, *J* = 15.4, 7.8 Hz, 1H), 3.20 (*m*, 1H), 2.75 (*dd*, *J* = 19.1, 5.7 Hz, 1H), 2.45 (*m*, 3H), 2.33 (*dt*, *J* = 14.7, 4.4 Hz, 1H), 1.99 (*dt*, *J* = 14.7, 2.6 Hz, 1H). **^13^C NMR** (101 MHz, CDCl_3_) δ 167.38, 166.00, 151.05, 141.54, 128.94, 128.68, 127.95, 127.21, 126.29, 126.14, 125.22, 124.06, 123.49, 116.65, 81.39, 51.73, 48.91, 34.33, 31.20, 29.96, 28.30, 21.97 **HRMS** (ESI+) *m*/*z* calcd. for C_22_H_21_NO_4_S [M + H]^+^ 396.1264 found 396.1293; **m.p.** 111–117 °C (205 °C decomp.).

**(3a*S*,5*S*,7a*R*,13b*R*)-6-(2-(1H-indol-3-yl)ethyl)-5-hydroxy-1,3a,4,5,6,13b-hexahydro-7H,8H-chromeno [4,3-i]isoquinoline-7,8-dione (4Be)**: The reaction was carried out following the general procedure to furnish; after 36 h, the crude product was a 85:15 mixture of diastereoisomers; dr was determined by the integration of ^1^H-NMR signal: *δ_major_* 6.18 ppm (*m*), *δ_minor_* 6.04 ppm (*m*). The crude product was purified by flash column chromatography with 20% EtOAc-hexane to afford product **4Be** (20.5 mg, 80% yield, 85% ee) as a brown crystalline solid. The ee value was determined by **HPLC** analysis on a Daicel Chiralpak OD-H column: 90:10 hexane/*i*-PrOH, flow rate 1 mL/min, λ = 210 nm: *τ_major_* = 3.8 min, *τ_minor_* = 5.2 min. [α]_22_^D^= −112.94 (*c* = 0.5, acetone). **^1^H NMR** (400 MHz, CDCl_3_) δ 7.99 (*br*, 1H), 7.74 (*d*, *J* = 7.8 Hz, 1H), 7.36 (*m*, 1H), 7.28 (*m*, 2H), 7.17 (*m*, 4H), 7.08 (*dd*, *J* = 8.1, 1.3 Hz, 1H), 5.98 (*dd*, *J* = 10.3, 5.9 Hz, 1H), 5.67 (*d*, *J* = 10.3 Hz, 1H), 4.61 (*m*, 1H), 4.32 (*m*, 1H), 4.00 (*ddd*, *J* = 13.0, 8.1, 4.7 Hz, 1H), 3.69 (*dt*, *J* = 13.3, 7.9 Hz, 1H), 3.26 (*m*, 1H), 3.14 (*ddd*, *J* = 13.6, 7.9, 4.7 Hz, 1H), 2.75 (*dd*, *J* = 18.0, 5.8 Hz, 1H), 2.46 (*m*, 3H), 2.28 (*dt*, *J* = 14.7, 4.3 Hz, 1H), 1.94 (*m*, 1H). **^13^C NMR** (101 MHz, CDCl_3_) δ 171.30, 166.02, 151.07, 144.83, 136.45, 129.05, 128.64, 127.81, 126.33, 125.21, 123.61, 123.01, 122.17, 119.64, 119.11, 116.63, 113.21, 111.23, 81.24, 47.51, 34.41, 31.15, 30.03, 29.85, 23.70, 21.97 **HRMS** (ESI+) *m*/*z* calcd. for C_26_H_24_N_2_O_4_ [M + H]^+^ 429.1809 found 429.1799; **m.p.** 119–125 °C (180–190 °C decomp.).

**(3a*S*,5*S*,7a*R*,13b*R*)-6-benzyl-5-hydroxy-12-methyl-1,3a,4,5,6,13b-hexahydro-7*H*,8*H*-chromeno [4,3-*i*]isoquinoline-7,8-dione (4Bh)**: The reaction was carried out following the general procedure to furnish; after 36 h, the crude product was a >95:5 mixture of diastereoisomers; dr was determined by the integration of ^1^H-NMR signal: *δ_major_* 5.79 ppm (*m*), *δ_minor_* 5.88 ppm (*m*). The crude product was purified by flash column chromatography with 20% EtOAc-hexane to afford product **4Bh** (21.2 mg, 91% yield, >99% ee) as a colorless crystalline solid. The ee value was determined by **HPLC** analysis on a Daicel Chiralpak OD-H column: 90:10 hexane/*i*-PrOH, flow rate 1 mL/min, λ = 210 nm: *τ_major_* = 39.6 min, *τ_minor_* = 47.0 min. [α]_22_^D^= −223.06 (*c* = 0.5, acetone). **^1^H NMR** (400 MHz, CDCl_3_) δ 7.45 (*d*, *J* = 7.2 Hz, 2H), 7.35 (*m*, 3H), 7.29 (*m*, 2H), 6.96 (*d*, *J* = 8.2 Hz, 1H), 6.03 (*m*, 1H), 5.73 (*dt*, *J* = 10.2, 2.6 Hz, 1H), 5.42 (*d*, *J* = 15.0 Hz, 1H), 4.84 (*m*, 1H), 4.33 (*d*, *J* = 15.0 Hz, 1H), 4.29 (*d*, *J* = 3.9 Hz, 1H), 2.76 (*dd*, *J* = 18.9, 6.0 Hz, 1H), 2.55 (*d*, *J* = 4.5 Hz, 2H), 2.47 (*m*, 1H), 2.33 (*m*, 4H), 2.05 (*m*, 1H). **^13^C NMR** (101 MHz, CDCl_3_) δ 167.51, 166.38, 148.91, 136.82, 135.28, 134.72, 128.97, 128.88, 128.70, 127.98, 127.40, 126.61, 116.24, 78.99, 47.61, 34.34, 31.99, 31.45, 31.18, 30.22, 29.97, 29.42, 22.75, 21.93 **HRMS** (ESI+) *m*/*z* calcd. for C_24_H_23_NO_4_ [M + H]^+^ 390.1700 found 390.1717; **m.p.** 114–118 °C (205 °C decomp.).

**(3a*S*,5*S*,7a*R*,13b*R*)-6-benzyl-5-hydroxy-3-methyl-1,3a,4,5,6,13b-hexahydro-7*H*,8*H*-chromeno [4,3-*i*]isoquinoline-7,8-dione (4Ca)**: The reaction was carried out following the general procedure to furnish; after 36 h, the crude product was a 83:17 mixture of diastereoisomers; dr was determined by the integration of ^1^H-NMR signal: *δ_major_* 4.34 ppm (*d*), *δ_minor_* 4.48 ppm (*d*). The crude product was purified by flash column chromatography with 20% EtOAc-hexane to afford product **4Ca** (21.0 mg, 90% yield, 98% ee) as a brown crystalline solid. The ee value was determined by **HPLC** analysis on a Daicel Chiralpak OD-H column: 90:10 hexane/*i*-PrOH, flow rate 1 mL/min, λ = 210 nm: *τ_major_* = 26.0 min, *τ_minor_* = 15.8 min. [α]_22_^D^ = −541.36 (*c* = 0.5, acetone). **^1^H NMR** (400 MHz, CDCl_3_) δ 7.42 (*d*, *J* = 7.2 Hz, 2H), 7.36 (*m*, 2H), 7.29 (*dt*, *J* = 7.5, 1.4 Hz, 2H), 7.17 (*m*, 2H), 7.06 (*dd*, *J* = 8.0, 1.2 Hz, 1H), 5.71 (*m*, 1H), 5.35 (*d*, *J* = 15.0 Hz, 1H), 4.83 (*t*, *J* = 3.3 Hz, 1H), 4.35 (*d*, *J* = 15.0 Hz, 1H), 4.24 (*t*, *J* = 3.8 Hz, 1H), 2.67 (*m*,1H), 2.41 (*m*, 3H), 2.29 (*m*, 2H), 2.91 (*s*, 3H). **^13^C NMR** (101 MHz, CDCl_3_) δ 160.51, 158.95, 142.61, 139.83, 137.21, 133.77, 128.88, 128.16, 126.44, 125.11, 116.55, 114.25, 102.59, 92.72, 62.79, 49.49, 34.83, 32.57, 31.56, 31.07, 28.68, 22.82 **HRMS** (ESI+) *m*/*z* calcd. for C_24_H_23_NO_4_ [M + H]^+^ 390.1700 found 390.1707; **m.p.** 100–106 °C (180 °C decomp.).

**(3a*S*,5*S*,7a*R*,13b*R*)-6-benzyl-5-hydroxy-2,3-dimethyl-1,3a,4,5,6,13b-hexahydro-7*H*,8*H*-chromeno [4,3-*i*]isoquinoline-7,8-dione (4Da)**: The reaction was carried out following the general procedure to furnish; after 36 h, the crude product was a 92:8 mixture of diastereoisomers; dr was determined by the integration of ^1^H-NMR signal: *δ_major_* 4.80 ppm (*m*), *δ_minor_* 4.95 ppm (*m*). The crude product was purified by flash column chromatography with 20% EtOAc-hexane to afford product **4Da** (23.4 mg, 97% yield, 97% ee) as a white crystalline solid. The ee value was determined by **HPLC** analysis on a Daicel Chiralpak OD-H column: 90:10 hexane/*i*-PrOH, flow rate 1 mL/min, λ = 210 nm: *τ_major_* = 22.5 min, *τ_minor_* = 19.5 min. [α]_22_^D^= −1688.96 (*c* = 0.5, acetone). **^1^H NMR** (400 MHz, DMSO-*d*_6_) δ 7.30 (*m*, 5H), 7.18 (*m*, 1H), 7.12 (*m*, 2H), 7.02 (*m*, 1H), 4.81 (*q*, *J* = 6.9 Hz, 1H), 4.59 (*d*, *J* = 15.2 Hz, 1H), 4.33 (*d*, *J* = 15.3 Hz, 1H), 3.79 (*t*, *J* = 7.8 Hz, 1H), 2.74 (*m*, 1H), 2.45 (*m*, 1H), 2.33 (*ddd*, *J* = 14.3, 5.8, 3.8 Hz, 1H), 1.84 (*m*, 2H), 1.65 (*s*, 3H), 1.59 (*m*, 1H), 1.55 (*s*, 3H). **^13^C NMR** (101 MHz, CDCl_3_) δ 167.37, 165.89, 151.67, 136.63, 129.40, 128.80, 128.35, 128.01, 127.84, 126.67, 126.38, 126.04, 124.47, 123.42, 116.85, 109.13, 52.10, 49.29, 38.66, 35.37, 33.39, 18.95, 17.31 **HRMS** (ESI+) *m*/*z* calcd. for C_25_H_25_NO_4_ [M + H]^+^ 404.1856 found 404.1875; **m.p.** 85–88 °C (180 °C decomp.).

**(6a*R*,9*S*,10a*S*,15b*R*)-8-benzyl-9-hydroxy-8,9,10,10a,12,13,14,15,15a,15b-decahydro-6*H*,7*H*-benzo[g]chromeno [4,3-*i*]isoquinoline-6,7-dione (4Ea)**: The reaction was carried out following the general procedure to furnish; after 36 h, the crude product was a 80:20 mixture of diastereoisomers; dr was determined by the integration of ^1^H-NMR signal: *δ_major_* 4.45 ppm (*d*), *δ_minor_* 4.53 ppm (*d*). The crude product was purified by flash column chromatography with 20% EtOAc-hexane to afford product **4Ea** (18.2 mg, 71% yield, >99% ee) as a white crystalline solid. The ee value was determined by **HPLC** analysis on a Daicel Chiralpak OD-H column: 90:10 hexane/*i*-PrOH, flow rate 1 mL/min, λ = 210 nm: *τ_major_* = 21.1 min, *τ_minor_* = 15.3 min. [α]_22_^D^ = −127.54 (*c* = 0.5, acetone). **^1^H NMR** (400 MHz, CDCl_3_) δ 7.33 (*ddd*, *J* = 8.0, 7.3, 1.7 Hz, 1H), 7.27 (*m*, 3H), 7.17 (*dd*, *J* = 8.1, 1.2 Hz, 1H), 7.09 (*td*, *J* = 7.4, 1.2 Hz, 1H), 7.01 (*m*, 3H), 5.91 (*dd*, *J* = 7.7, 3.2 Hz, 1H), 5.55 (*dt*, *J* = 6.6, 2.0 Hz, 1H), 4.99 (*dd*, *J* = 7.8, 2.1 Hz, 1H), 4.81 (*d*, *J* = 14.8 Hz, 1H), 4.12 (*d*, *J* = 14.8 Hz, 1H), 4.03 (*m*, 1H), 2.90 (*d*, *J* = 9.4 Hz, 1H), 2.24 (*m*, 1H), 2.01 (*m*, 1H), 1.89 (*m*, 1H), 1.75 (*m*, 3H), 1.21 (*m*, 3H), 0.93 (*m*, 2H). **^13^C NMR** (101 MHz, CDCl_3_) δ 167.74, 165.76, 152.02, 138.68, 136.57, 129.05, 128.80, 128.29, 128.08, 127.93, 125.53, 125.08, 123.82, 117.39, 116.99, 111.01, 51.86, 49.13, 42.08, 40.42, 34.94, 33.77, 32.51, 27.26, 26.03 **HRMS** (ESI+) *m*/*z* calcd. for C_27_H_27_NO_4_ [M + H]^+^ 430.2013 found 430.2026; **m.p.** 125–133 °C (200 °C decomp.).

***tert*-Butyl (2*S*,4a*R*,10b*R*,16c*S*)-3-benzyl-2-hydroxy-4,5-dioxo-1,3,4,10b,11,16c-hexahydro-5*H*-chromeno [4,3-*b*]pyrido [3,4-*c*]carbazole-12(2*H*)-carboxylate (4Fa)**: The reaction was carried out following the general procedure to furnish; after 36 h, the crude product was a 91:9 mixture of diastereoisomers; dr was determined by the integration of ^1^H-NMR signal: *δ_major_* 5.31 ppm (*m*), *δ_minor_* 4.97 ppm (*m*). The crude product was purified by flash column chromatography with 20% EtOAc-hexane to afford product **4Fa** (30.4 mg, 90% yield, 89% ee) as a pale crystalline solid. The ee value was determined by **HPLC** analysis on a Daicel Chiralpak OD-H column: 90:10 hexane/*i*-PrOH, flow rate 1 mL/min, λ = 210 nm: *τ_major_* = 29.3 min, *τ_minor_* = 20.8 min. [α]_22_^D^= −1034.42 (*c* = 0.5, acetone). **^1^H NMR** (500 MHz, CDCl_3_) δ 8.16 (*d*, *J* = 8.2 Hz, 1H), 7.39 (*m*, 3H), 7.34 (*t*, *J* = 7.6 Hz, 2H), 7.28 (*m*, 2H), 7.22 (*m*, 2H), 7.08 (*m*, 1H), 7.03 (*m*, 2H), 5.31 (*d*, *J* = 15.0 Hz, 1H), 4.85 (*m*, 1H), 4.58 (*m*, 1H), 4.41 (*m*, 1H), 4.27 (*d*, *J* = 14.9 Hz, 1H), 3.96 (*m*, 1H), 3.28 (*m*, 2H), 2.87 (*m*, 1H), 2.60 (*dt*, *J* = 15.2, 4.4 Hz, 1H), 1.75 (*m*, 9H). **^13^C NMR** (101 MHz, CDCl_3_) δ 171.38, 167.63, 165.88, 150.94, 150.15, 136.59, 136.14, 128.72, 128.69, 128.35, 127.96, 127.43, 127.43, 126.95, 125.67, 125.64, 125.45, 125.39, 124.70, 123.25, 119.14, 116.57, 115.99, 85.05, 78.43, 60.54, 52.71, 35.54, 28.48, 28.43, 21.17, 14.28 **HRMS** (ESI+) *m*/*z* calcd. for C_34_H_32_N_2_O_6_ [M + H]^+^ 565.2333 found 565.2373; **m.p.** 141–150 °C (190 °C decomp.).

***tert*-Butyl (2*S*,4a*R*,10b*R*,16c*S*)-2-hydroxy-3-(3-methoxyphenethyl)-4,5-dioxo-1,3,4,10b,11,16c-hexahydro-5*H*-chromeno [4,3-*b*]pyrido [3,4-*c*]carbazole-12(2*H*)-carboxylate (4Fb)**: The reaction was carried out following the general procedure to furnish; after 36 h, the crude product was a 90:10 mixture of diastereoisomers; dr was determined by the integration of ^1^H-NMR signal: *δ_major_* 2.18 ppm (*s*), *δ_minor_* 2.11 ppm (*s*). The crude product was purified by flash column chromatography with 20% EtOAc-hexane to afford product **4Fb** (33.2 mg, 91% yield, 94.4% ee) as a white crystalline solid. The ee value was determined by **HPLC** analysis on a Daicel Chiralpak OD-H column: 90:10 hexane/*i*-PrOH, flow rate 1 mL/min, λ = 210 nm: *τ_major_* = 24.2 min, *τ_minor_* = 30.9 min. [α]_22_^D^= −312.92 (*c* = 0.5, acetone). **^1^H NMR** (400 MHz, CDCl_3_) δ 8.14 (*m*, 1H), 7.36 (*dd*, *J* = 8.1, 5.4 Hz, 1H), 7.23 (*m*, 1H), 7.16 (*m*, 2H), 7.06 (*m*, 4H), 6.89 (*m*, 1H), 6.65 (*m*, 2H), 4.53 (*m*, 1H), 4.31 (*m*, 1H), 4.20 (*dd*, *J* = 8.2, 4.4 Hz, 1H), 3.93 (*m*, 1H), 3.80 (*m*, 3H), 3.63 (*dt*, *J* = 13.5, 6.9 Hz, 1H), 3.46 (*m*, 1H), 3.30 (*m*, 2H), 2.86 (*m*, 1H), 2.74 (*m*, 2H), 2.18 (*m*, 1H), 1.72 (*m*, 9H). **^13^C NMR** (101 MHz, CDCl_3_) δ 168.26, 166.25, 159.81, 151.19, 141.05, 136.25, 129.62, 129.50, 128.74, 127.39, 125.81, 125.40, 124.29, 122.96, 121.14, 119.17, 118.37, 116.63, 116.58, 115.91, 114.48, 111.98, 84.75, 80.49, 78.65, 55.40, 55.31, 52.91, 45.43, 35.77, 34.31, 32.85, 28.52, 28.49 **HRMS** (ESI+) *m*/*z* calcd. for C_36_H_36_N_2_O_7_ [M + H]^+^ 609.2595 found 609.2607; **m.p.** 90–96 °C (180 °C decomp.).

***tert*-Butyl (2*S*,4a*R*,10b*R*,16c*S*)-2-hydroxy-4,5-dioxo-3-(2-(thiophen-3-yl)ethyl)-1,3,4,10b,11,16c-hexahydro-5*H*-chromeno [4,3-*b*]pyrido [3,4-*c*]carbazole-12(2*H*)-carboxylate (4Fd)**: The reaction was carried out following the general procedure to furnish; after 36 h, the crude product was a 91:9 mixture of diastereoisomers; dr was determined by the integration of ^1^H-NMR signal: *δ_major_* 5.78 ppm (*d*), *δ_minor_* 5.17 ppm (*d*). The crude product was purified by flash column chromatography with 20% EtOAc-hexane to afford product **4Fd** (32.9 mg, 94% yield, 96.2% ee) as a colorless crystalline solid. The ee value was determined by **HPLC** analysis on a Daicel Chiralpak OD-H column: 90:10 hexane/*i*-PrOH, flow rate 1 mL/min, λ = 210 nm: *τ_major_* = 49.4 min, *τ_minor_* = 23.5 min. [α]_22_^D^= −23.85 (*c* = 0.5, acetone). **^1^H NMR** (400 MHz, CDCl_3_) δ 8.14 (*d*, *J* = 8.4 Hz, 1H), 7.37 (*d*, *J* = 7.8 Hz, 1H), 7.19 (*m*, 3H), 7.06 (*m*, 4H), 6.95 (*m*, 1H), 6.89 (*dd*, *J* = 5.1, 3.4 Hz, 1H), 4.63 (*ddd*, *J* = 11.6, 4.6, 2.2 Hz, 1H), 4.53 (*m*, 1H), 3.88 (*m*, 2H), 3.60 (*m*, 1H), 3.28 (*m*, 3H), 3.16 (*m*, 1H), 3.03 (*m*, 1H), 2.78 (*m*, 1H), 2.56 (*dt*, *J* = 15.2, 4.4 Hz, 1H), 1.73 (*m*, 9H). **^13^C NMR** (101 MHz, CDCl_3_) δ 167.57, 165.59, 151.02, 150.19, 141.59, 136.23, 128.75, 127.13, 126.96, 126.87, 126.03, 125.71, 125.51, 124.79, 124.30, 124.04, 123.81, 123.31, 122.97, 119.20, 116.62, 116.04, 85.16, 80.65, 60.54, 52.63, 48.57, 35.53, 28.51, 28.07, 14.34 **HRMS** (ESI+) *m*/*z* calcd. for C_33_H_32_N_2_O_6_S [M + H]^+^ 585.2054 found 585.2063; **m.p.** 110–114 °C (180–190 °C decomp.).

***tert*-Butyl (2*S*,4a*R*,10b*R*,16c*S*)-3-(2-(1*H*-indol-3-yl)ethyl)-2-hydroxy-4,5-dioxo-1,3,4,10b,11,16c-hexahydro-5*H*-chromeno [4,3-*b*]pyrido [3,4-*c*]carbazole-12(2*H*)-carboxylate (4Fe)**: The reaction was carried out following the general procedure to furnish; after 36 h, the crude product was a 87:13 mixture of diastereoisomers; dr was determined by the integration of ^1^H-NMR signal: *δ_major_* 4.47 ppm (*m*), *δ_minor_* 4.61 ppm (*m*). The crude product was purified by flash column chromatography with 20% EtOAc-hexane to afford product **4Fe** (34.4 mg, 93% yield, 84% ee) as a white crystalline solid. The ee value was determined by **HPLC** analysis on a Daicel Chiralpak OD-H column: 60:40: hexane/*i*-PrOH, flow rate 1 mL/min, λ = 210 nm: *τ_major_* = 19.4 min, *τ_minor_* = 15 min. [α]_22_^D^= −175.5 (*c* = 0.5, acetone). **^1^H NMR** (400 MHz, CDCl_3_) δ 8.13 (*dt*, *J* = 8.4, 0.9 Hz, 1H), 7.99 (*br*, 1H), 7.69 (*m*, 1H), 7.35 (*m*, 2H), 7.15 (*m*, 9H), 4.61 (*m*, 2H), 3.98 (*m*, 2H), 3.60 (*dt*, *J* = 13.2, 7.9 Hz, 1H), 3.25 (*m*, 3H), 3.09 (*m*, 1H), 2.73 (*m*, 1H), 2.51 (*dt*, *J* = 15.0, 4.3 Hz, 1H), 1.74 (*s*, 9H), 1.60 (*m*, 1H). **^13^C NMR** (101 MHz, CDCl_3_) δ 167.76, 165.36, 151.05, 150.21, 136.43, 136.24, 128.72, 127.43, 126.99, 125.71, 125.51, 124.78, 123.30, 122.87, 122.18, 119.63, 119.19, 119.04, 116.62, 116.04, 113.18, 111.24, 85.16, 80.43, 53.57, 52.64, 46.95, 35.62, 28.56, 28.52, 23.50 **HRMS** (ESI+) *m*/*z* calcd. for C_37_H_35_N_3_O_6_ [M + H]^+^ 618.2599 found 618.2609; **m.p.** 119–123 °C (210 °C decomp.).

**(2*S*,4a*R*,10b*R*,16c*S*)-3-benzyl-2-hydroxy-12-methyl-2,3,10b,11,12,16c-hexahydro-5*H*-chromeno [4,3-*b*]pyrido [3,4-*c*]carbazole-4,5(1*H*)-dione (4Ga)**: The reaction was carried out following the general procedure to furnish; after 36 h, the crude product was a 88:12 mixture of diastereoisomers; dr was determined by the integration of ^1^H-NMR signal: *δ_major_* 5.34 ppm (*m*), *δ_minor_* 5.51 ppm (*m*). The crude product was purified by flash column chromatography with 20% EtOAc-hexane to afford product **4Ga** (20.6 mg, 72% yield, 40% ee) as a pale crystalline solid. The ee value was determined by **HPLC** analysis on a Daicel Chiralpak OD-H column: 90:10 hexane/*i*-PrOH, flow rate 1 mL/min, λ = 210 nm: *τ_major_* = 46.1 min, *τ_minor_* = 52.8 min. [α]_22_^D^ = −40.7 (*c* = 0.5, acetone). **^1^H NMR** (400 MHz, CDCl_3_) δ 7.51–7.21 (*m*, 7H), 7.20 – 6.95 (*m*, 6H), 6.00 (*dd*, *J* = 7.8, 2.3 Hz, 1H), 5.43 (*ddq*, *J* = 5.2, 3.8, 1.8 Hz, 1H), 5.08 (*dd*, *J* = 7.8, 3.6 Hz, 1H), 4.84 (*d*, *J* = 15.0 Hz, 1H), 4.37 (*d*, *J* = 15.0 Hz, 1H), 3.60 (*t*, *J* = 7.3 Hz, 1H), 3.51 (*s*, 1H), 2.57 – 2.47 (*m*, 1H), 2.26 – 2.16 (*m*, 1H), 1.73 (*q*, *J* = 2.0 Hz, 3H). **^13^C NMR** (101 MHz, CDCl_3_) δ 167.16, 165.72, 151.57, 136.57, 133.79, 131.35, 130.31, 129.42, 128.81, 128.62, 128.36, 127.84, 127.00, 126.76, 125.79, 125.50, 124.55, 119.60, 116.80, 108.22, 51.80, 49.37, 37.05, 32.45, 21.65 **HRMS** (ESI+) *m*/*z* calcd. for C_30_H_26_N_2_O_4_ [M + H]^+^ 479.1965 found 479.1955; **m.p.** 160–165 °C (240 °C decomp.).

### 3.4. Experimental Procedure for Compound ***5Ba***

To a stirred solution of the corresponding alcohol **4Ba** (25 mg, 0.066 mmol) in dry toluene (0.7 mL) was added trifluoroacetic acid (7.5 mg, 0.066 mmol). The reaction mixture was stirred at room temperature and monitored by TLC until complete consumption of the starting material. The resulting mixture was concentrated in vacuo, and the residue was purified by flash column chromatography to afford the dehydrated desired compound.

### 3.5. Characterization Data of Product ***5Ba***

**(3a*R*,7a*R*,13b*R*)-6-benzyl-1,3a,6,13b-tetrahydro-7*H*,8*H*-chromeno [4,3-*i*]isoquinoline-7,8-dione (5Ba)**: The reaction was carried out following the general procedure to furnish; after 18 h, the crude product was a single diastereoisomer. The crude product was purified by flash column chromatography with 20% EtOAc-hexane to afford product **5Ba** in 99% yield (23.25 mg) as a white foam. **^1^H NMR** (400 MHz, CDCl_3_) δ 7.30 (m, 3H), 7.16 (m, 6H), 6.05 (d, *J* = 7.7 Hz, 1H), 5.78 (dq, *J* = 9.9, 3.7 Hz, 2H), 5.43 (m, 1H), 5.05 (dd, *J* = 7.8, 5.4 Hz, 2H), 4.91 (d, *J* = 15.3 Hz, 2H), 4.58 (d, *J* = 15.3 Hz, 2H), 3.92 (t, *J* = 5.6 Hz, 1H), 3.23 (s, 1H), 2.55 (m, 2H). **^13^C NMR** (101 MHz, CDCl_3_) δ 166.69, 165.30, 151.35, 136.61, 129.19, 128.82, 128.72, 128.38, 127.74, 127.65, 126.41, 125.45, 124.88, 124.51, 124.07, 116.73, 106.80, 49.79, 32.70, 32.32. **HRMS** (ESI+) *m*/*z* calcd. for C_23_H_19_NO_3_ [M + H]^+^ 358.1438 found 358.1411 *m*/*z*.

### 3.6. Experimental Procedure for Compound ***6Ba***

To a stirred solution of the corresponding alcohol **4Ba** (25 mg, 0.06 mmol) in anhydrous DCM (0.7 mL) at 0 °C, triethylsilane Et_3_SiH (23 μL, 0.12 mmol) and boron trifluoride diethyl etherate BF_3_-Et_2_O (29.6 μL, 0.24 mmol) were added. The reaction mixture was allowed to warm to ambient temperature and stirred for 90 min. Upon completion, as monitored by TLC, the reaction was quenched with saturated NaHCO_3_ solution and extracted with DCM. The combined organic layers were dried over anhydrous Na_2_SO_4_, filtered, and concentrated in vacuo. The crude residue was purified by flash column chromatography to afford the desired reduced product.

### 3.7. Characterization Data of Product ***6Ba***

**(3a*S*,7a*R*,13b*R*)-6-benzyl-1,3a,4,5,6,13b-hexahydro-7*H*,8*H*-chromeno [4,3-*i*]isoquinoline-7,8-dione (6Ba)**: The reaction was carried out following the general procedure to furnish the crude product as a single diastereoisomer. The crude product was purified by flash column chromatography with 20% EtOAc-hexane to afford product **6Ba** in 71% yield (17 mg) as a pale amorphous solid. [α]_D_^22^ = −1621.66 (*c* = 1, acetone). **^1^H NMR** (400 MHz, CDCl_3_) δ 7.31 (*m*, 5H), 7.24 (*m*, 2H), 7.16 (*td*, *J* = 7.5, 1.3 Hz, 1H), 7.07 (*dd*, *J* = 8.0, 1.3 Hz, 1H), 5.92 (*ddd*, *J* = 10.0, 5.3, 2.5 Hz, 1H), 5.35 (*dt*, *J* = 9.9, 1.3 Hz, 1H), 4.72 (*m*, 2H), 4.26 (*br d*, *J* = 4.7 Hz, 1H) 3.18 (*d*, *J* = 3.2 Hz, 1H), 3.16 (*m*, 1H), 2.74 (*m*, 1H), 2.45 (*m*, 2H), 2.22 (*m*, 1H), 1.66 (*m*, 1H). **^13^C NMR** (101 MHz, CDCl_3_) δ 168.23, 166.52, 151.16, 136.79, 128.78, 128.41, 127.84, 127.51, 126.75, 126.10, 125.06, 124.34, 116.56, 52.04, 51.15, 44.05, 34.15, 31.08, 23.66, 22.12 **HRMS** (ESI+) *m*/*z* calcd. for C_23_H_21_NO_3_ [M + H]^+^ 360.1594 found 360.1621; **m.p.** 145–147 °C.

### 3.8. Experimental Procedure for Compound ***7Ba***

To a stirred solution of the corresponding alcohol **4Ba** (25 mg, 0.0664 mmol) in anhydrous DCM (0.7 mL), pyridinium chlorochromate PCC (28.6 mg, 1.1329 mmol) was added. The reaction mixture was stirred at ambient temperature and monitored by TLC until complete consumption of the starting material. The resulting mixture was filtered through a pad of Celite and washed with DCM. The filtrate was concentrated in vacuo, and the residue was purified by flash column chromatography to afford the desired carbonyl compound.

### 3.9. Characterization Data of Product ***7Ba***

**(3a*S*,7a*R*,13b*R*)-6-benzyl-1,3a,4,13b-tetrahydro-5*H*,8*H*-chromeno [4,3-*i*]isoquinoline-5,7,8(6*H*)-trione (7Ba)**: The reaction was carried out following the general procedure to furnish; after 3 h, the crude product was a single diastereoisomer. The crude product was purified by flash column chromatography with 20% EtOAc-hexane to afford product **7Ba** in 92% yield (23 mg) as a yellow semi-solid, which gradually crystallized upon standing at ambient temperature. [α]_22_^D^ = −1297.74 (*c* = 1, acetone). **^1^H NMR** (400 MHz, CDCl_3_) δ 7.26 (*m*, 8H), 7.11 (*m*, 1H), 5.81 (*ddt*, *J* = 10.2, 5.2, 2.4 Hz, 1H), 5.35 (*ddt*, *J* = 10.1, 3.0, 1.7 Hz, 1H), 5.14 (*d*, *J* = 14.2 Hz, 1H), 4.97 (*d*, *J* = 14.3 Hz, 1H), 4.17 (*m*, 1H), 2.96 (*dd*, *J* = 16.9, 5.6 Hz, 1H), 2.66 (*m*, 2H), 2.58 (*m*, 1H), 2.18 (*m*, 1H), **^13^C NMR** (101 MHz, CDCl_3_) δ 169.71, 169.63, 166.46, 150.83, 136.76, 128.99, 128.42, 128.35, 128.05, 127.45, 126.07, 125.93, 125.69, 123.21, 116.89, 52.62, 43.85, 34.94, 34.29, 28.12, 21.86 **HRMS** (ESI+) *m*/*z* calcd. for C_23_H_19_NO_4_ [M + H]^+^ 374.1387 found 374.1410 *m*/*z*.

## 4. Conclusions

In summary, we have developed a new practical and efficient strategy for the construction of chiral, complex spiro-bridged 3,4-dihydrocoumarins through an organocatalytic Diels–Alder/Nucleophilic ring-closing cascade sequence via trienamine activation. In general, the reaction proceeds under mild conditions, providing the desired products in good yields and with high levels of stereocontrol. Several substituents on both the amide moiety and aromatic ring of the coumarin, as well as different trienamine precursors, are well-tolerated. The observed stereochemistry of the new spiro-bridged 3,4-dihydrocoumarins is consistent with an *endo*-approach of the coumarin to the trienamine intermediate, followed by intramolecular nucleophilic attack to the *si*-face of the formyl group. Three different transformations on the piperidone ring demonstrate the synthetic utility of the obtained compounds. Additional transformations to extend the cascade process for access to more complex substrates are currently ongoing in our group.

## Figures and Tables

**Table 1:** Screening of the Diels–Alder/nucleophilic ring-closing cascade reaction ^[a]^.

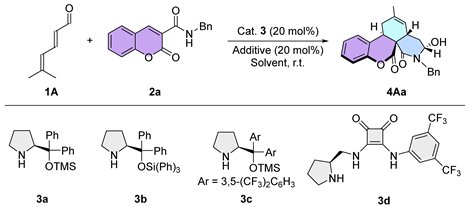								
Entry	Cat.	Solvent	Additive	*t* (h)	Conv. (%)	Yield (%) ^[b]^	dr ^[c]^	ee (%) ^[d]^
1	**3a**	CHCl_3_	PhCO_2_H	36	97	94	>95:5	0
2	**3b**	CHCl_3_	PhCO_2_H	36	98	94	>95:5	76
3	**3c**	CHCl_3_	PhCO_2_H	36	34	31	74:26	n.d.
4 ^[e]^	**3d**	CHCl_3_	DMA	96	15	10	71:29	n.d.
5	**3b**	CH_2_Cl_2_	PhCO_2_H	36	--	--	--	--
6	**3b**	THF	PhCO_2_H	36	66	51	80:20	10
7	**3b**	CH_3_CN	PhCO_2_H	36	66	50	91:9	10
8	**3b**	Dioxane	PhCO_2_H	36	76	68	68:32	97
9	**3b**	Toluene	PhCO_2_H	36	100	95	94:6	96

^[a]^ Reactions were performed with 0.1 mmol of **1A**, 0.066 mmol of **2a**, 20 mol% of catalyst and 20 mol% of additive in 0.7 mL of solvent at room temperature. ^[b]^ Yield of both diastereoisomers of **4Aa** after chromatographic purification on silica gel. ^[c]^ Determined by ^1^H NMR of crude product. ^[d]^ Measured by chiral HPLC. ^[e]^ 0.4 mol% of additive was used.

## Data Availability

Data contained within the article or Supplementary Materials.

## Supplementary Materials

The following supporting information can be downloaded at https://www.mdpi.com/article/10.3390/molecules31162827/s1. File S1. Experimental procedures, characterization data for all synthetized compounds, copies of ^1^H and ^13^C NMR spectra, chiral HPLC chromatograms, and crystallographic data.
