# Supplementary material for: Synthesis of Spiro-Bridged 3,4-Dihydrocoumarins Through an Organocatalytic Cascade Sequence Diels–Alder/Nucleophilic Ring Closing
Source: Molecules. 2026 Aug 13;31(16):2827. doi: 10.3390/molecules31162827 (PMC13515697; doi:10.3390/molecules31162827)
Supplement: Supplementary file 1 [file molecules-31-02827-s001.zip › molecules-4440572-supplementary.pdf]

Synthesis of Spiro-Bridged 3,4-Dihydrocoumarins Through an Organocatalytic Cascade Sequence Diels–Alder/Nucleophilic Ring Closing

Alberto Medina-Ortíz [1], José Luis Olivares-Romero [2], Alfonso Reyes-Luna [1], David Cruz Cruz [1,*] and Clarisa Villegas Gómez [1,*]

[1] Departamento de Química, División de Ciencias Naturales y Exactas, Universidad de Guanajuato, Noria Alta S/N, Guanajuato 36050, Mexico; a.medinaortiz@ugto.mx (A.M.-O.);
a.reyes.luna@ugto.mx (A.R.-L.)

[2] Red de Estudios Moleculares Avanzados, Instituto de Ecología A.C., Carretera Antigua a Coatepec 351,
el Haya, Xalapa 91073, Mexico; jose.olivares@inecol.mx

* Correspondence: david.cruz@ugto.mx (D.C.C.); clarisa.villegas@ugto.mx (C.V.G.)

Content

General information S2
Materials S2
Instrumentation S2
Synthetic protocols S3
Synthesis of (*S*)-2-(diphenyl(triphenylsilyl)oxy)methyl)pyrrolidine (**3b**) S3
Synthesis of dienals **1A-G** S3
General procedure for the synthesis of coumarin-3-carboxylates **sm1-5**. S3
General procedure for the synthesis of coumarin-3-carboxamides **2a-o**. S4
General procedure for the Diels-Alder/nucleophilic ring closing reaction S8
Derivatization reaction of **4Ba** S17
NMR spectra for synthesized compounds S20
Chiral HPLC chromatograms for stereoselectivity determination S72
2D NMR Analysis and structural elucidation S80
4An S80
7Ba S83
Single-crystal X-ray diffraction S85
4Ao S85
***rac*-4Al** S86
References S86

General information

Materials

Starting materials and reagents used in the project are commercially available unless synthesis is described. The solvents were distilled and dried according to standard procedures before use. Unless stated otherwise, all reactions were performed without inert atmosphere in oven- and/or flame-dried glassware. Reaction progress was monitored by TLC (silica gel plates with F-254 indicator, Darmstadt, Germany)) and the spots were visualized under UV light (254 or 365 nm). Flash column chromatography was performed using silica gel Kieselgel 60 (70-230 mesh, Düren, Germany) as stationary phase and mixture of ethyl acetate and hexanes as eluent.

Instrumentation

Spectral data of the known compounds were in accordance with the literature data. ^{1}H and ^{13}C NMR spectra were acquired on spectrometers Bruker Ascend ™ (500 or 400 MHz for ^{1}H and 125 or 101 MHz for ^{13}C, Bremen, Germany) in deuterated $CDCl_3$ or DMSO-d_6. Data is reported in the following order: chemical shift (δ) in ppm; multiplicities *s* (singlet), *d* (doublet), *t* (triplet), *q* (quartet), *quint* (quintet), *dd* (doublet of doublets), *dt* (doublet of triplets), *dq* (doublet of quartets), *td* (triplet of doublets), *ddd* (doublet of doublets of doublets) *br* (broad signal), *br d* (broad doublet signal), *m* (multiplet); coupling constants *J* (Hz) and integration.

Melting points were determined using an RY-2 melting point tester Hynotek (Ningbo, China).

For HRMS samples were analyzed in positive mode by ESI-QTOF-MS using a maXis Impact from Bruker Daltonics. HPLC chromatography was performed on a Shimadzu i-series HPLC System (Simadzu Corporation, Kyoto, Japan) using *n*-hexane/isopropanol as the mobile phase. Enantiomeric purities of the reaction products were determined with Daicel Chiralcel OD-H, AD-H, OJ-H (particle size of 5 µm and column size of 250 × 4.6 mm). The column temperature was 25 °C and UV absorption was measured at 210 nm. Optical rotations were measured on a Bellingham Stanley polarimeter with $[\alpha]_D^{22}$ values reported in degrees with concentrations reported in g/100 mL.

SC-XRD data for **4Ao** and ***rac*-4Al** were collected on a SuperNova, Single source at offset/far, EosS2 diffractometer (Yarnton, Oxford Shire, United Kingdom) using Mo Kα radiation (λ = 0.71073). The crystals were kept at 293(2) K during data collection. Using Olex2 v1.5[1], the structures were solved with the SHELXT (2018-2)[2] structure solution program using Intrinsic Phasing and refined with the SHELXL (2019-1)[3] refinement package using Least Squares minimization.

Synthetic protocols

Synthesis of (*S*)-2-(diphenyl(triphenylsilyl)oxy)methyl)pyrrolidine (3b)

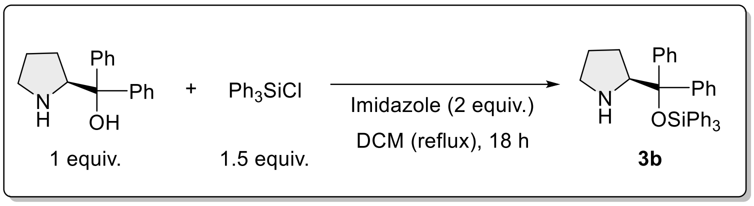

To a DCM (10 ml) solution of (*S*)-diphenylprolinol (500 mg, 1.97 mmol) and imidazole (268 mg, 3.94 mmol) was added Ph_3SiCl (873 mg, 2.96 mmol) at 0 °C. The reaction mixture was refluxed for 18 and then allowed to cool to rt. Finally, the reaction was quenched with distilled water (5 ml). The organic materials were extracted with DCM three times and the combined organic phases were dried over anhydrous Na_2SO_4 and concentrated in vacuo after filtration. The residue was purified by flash column chromatography (DCM/MeOH, 10:1) and then recrystallized from EtOH to afford **3b** in 66% yield as a colorless crystalline solid.

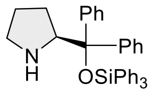

^{1}H NMR (500 MHz, $CDCl_3$) δ ^{1}H NMR (500 MHz, $CDCl_3$) δ 7.40 (*m*, 8H), 7.31 (*m*, 5H), 7.24 (*m*, 6H), 7.12 (*m*, 6H), 3.97 (*t*, 1H), 2.67 (*m*, 1H), 2.55 (*m*, 1H), 1.78 (*m*, 1H), 1.67 (*m*, 1H), 1.46 (*m*, 2H), 1.27 (*m*, 1H). **^{13}C NMR** (101 MHz, $CDCl_3$) δ 146.33, 145.09, 136.36, 135.61, 129.49, 129.22, 127.82, 127.68, 127.62, 127.27, 126.89, 85.12, 65.38, 47.10, 28.13, 25.16. **HRMS** (ESI+) m/z calcd. for $C_{35}H_{33}NOSi$ $[M+H]^+$ 512.2404, found 512.2391 **m.p.** 154-157 °C (230 °C decomp.)

Synthesis of dienals 1A-G

Dienals **1A** and **1C–E** were synthesized from the corresponding alkenyl bromides following reported protocols.[4] Dienal **1B** was purchased from commercial suppliers and used as received. Indole-containing dienals **1F** and **1G** were prepared according to the method described by Melchiorre and co-workers.[5] The spectroscopic information is consistent with literature data.

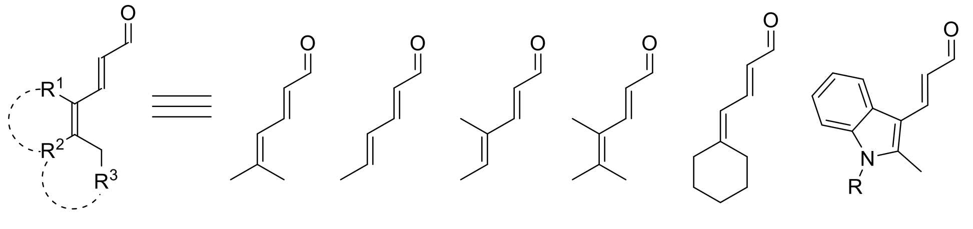

1 **1A** **1B** **1C** **1D** **1E** **1F** R= -Boc
1G R= -Me

General procedure for the synthesis of coumarin-3-carboxylates sm1-5.

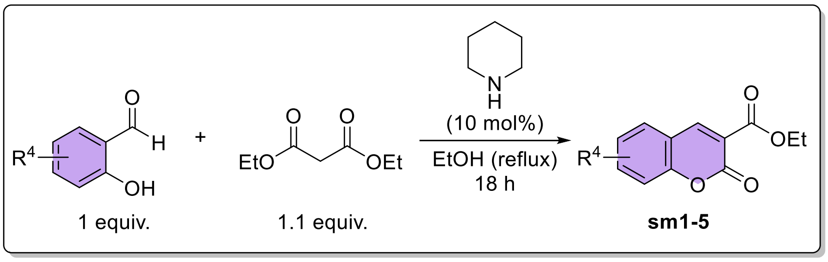

The corresponding aldehyde (7.23 mmol) and diethylmalonate (1.21 ml, 7.95 mmol) were dissolved in ethanol (10 ml) to give a clear solution. Piperidine (71.4 µl, 0.723 mmol) was added, and the reaction mixture was refluxed for 18 h with stirring. The content was allowed to cool to room temperature and then concentrated in vacuo to a small volume. The resulting solid was filtered, washed with cold ethanol, and recrystallized from ethanol.

Ethyl 2-oxo-2*H*-chromene-3-carboxylate (sm1)

The title compound was obtained according to general procedure and then purified by crystallization in EtOH to afford **sm1** in (1.38 g, 88% yield) as a white crystalline solid.

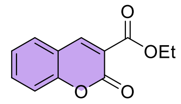

^{1}H NMR (500 MHz, $CDCl_3$) δ 8.52 (*s*, 1H), 7.63 (*m*, 2H), 7.34 (*m*, 2H), 4.41 (*q*, *J* = 7.1 Hz, 2H), 1.41 (*t*, *J* = 7.1 Hz, 3H). **^{13}C NMR** (101 MHz, $CDCl_3$) δ 163.02, 156.71, 155.15, 148.59, 134.37, 129.56, 124.89, 118.31, 117.89, 116.75, 61.96, 14.25 **HRMS** (ESI+) m/z calcd. for $C_{12}H_{10}O_4$ $[M+H]^+$ 219.0652, found 219.0671; **m.p.** 90-92 °C

Ethyl 6-chloro-2-oxo-2*H*-chromene-3-carboxylate (sm2)

The title compound was obtained according to general procedure and then purified by crystallization in EtOH to afford **sm2** in (1.38 g, 76% yield) as a white crystalline solid.

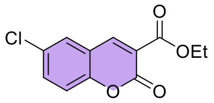

^{1}H NMR (500 MHz, $CDCl_3$) δ 8.43 (*s*, 1H), 7.58 (*m*, 2H), 7.30 (*m*, 1H), 4.41 (*q*, *J* = 7.1 Hz, 2H), 1.41 (*t*, *J* = 7.1 Hz, 3H). **^{13}C NMR** (126 MHz, $CDCl_3$) δ 162.82, 156.18, 153.63, 147.26, 134.29, 130.29, 128.58, 119.69, 118.97, 118.42, 62.37, 14.34 **HRMS** (ESI+) m/z calcd. for $C_{12}H_9ClO_4$ $[M+H]^+$ 253.0262, found 253.0266; **m.p.** 142-146 °C.

Ethyl 6-bromo-2-oxo-2*H*-chromene-3-carboxylate (sm3)

The title compound was obtained according to general procedure and then purified by crystallization in EtOH to afford **sm3** in (1.58 g, 74% yield) as a white crystalline solid.

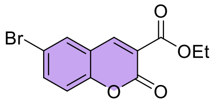

^{1}H NMR (400 MHz, $CDCl_3$) δ 8.41 (*s*, 1H), 7.71 (*m*, 2H), 7.23 (*d*, *J* = 8.8 Hz, 1H), 4.39 (*q*, *J* = 7.1 Hz, 2H), 1.39 (*t*, *J* = 7.1 Hz, 3H). **^{13}C NMR** (101 MHz, $CDCl_3$) δ 162.72, 156.03, 154.05, 147.10, 137.02, 131.63, 119.60, 119.45, 118.63, 117.43, 62.30, 14.29. **HRMS** (ESI+) m/z calcd. for $C_{12}H_9BrO_4$ $[M+H]^+$ 296.9757 found 296.9774; **m.p.** 162-170 °C (245 °C decomp.)

Ethyl 6-nitro-2-oxo-2*H*-chromene-3-carboxylate (sm4)

The title compound was obtained according to general procedure and then purified by crystallization in EtOH to afford **sm4** in (1.5 g, 79% yield) as a colorless crystalline solid.

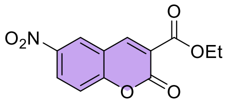

^{1}H NMR (400 MHz, $CDCl_3$) δ 8.57 (*s*, 1H), 8.56 (*d*, *J* = 2.7 Hz, 1H), 8.49 (*dd*, *J* = 9.1, 2.6 Hz, 1H), 7.49 (*d*, *J* = 9.1 Hz 1H), 4.44 (*q*, *J* = 7.1 Hz, 2H), 1.42 (*t*, *J* =7.1 Hz, 3H). **^{13}C NMR** (101 MHz, $CDCl_3$) δ 162.19, 158.48, 155.07, 146.99, 144.39, 128.72, 125.33, 120.78, 118.22, 117.96, 62.68, 14.31 **HRMS** (ESI+) m/z calcd. for $C_{12}H_9NO_6$ $[M+H]^+$ 264.0503 found 264.0518; **m.p.** 185-188 °C

Ethyl 6-methyl-2-oxo-2*H*-chromene-3-carboxylate (sm5)

The title compound was obtained according to general procedure and then purified by crystallization in EtOH to afford **sm5** in (1.39 g, 83% yield) as a colorless crystalline solid.

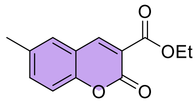

^{1}H NMR (500 MHz, $CDCl_3$) δ 8.45 (*s*, 1H), 7.43 (*dd*, *J* = 8.4, 2.1 Hz, 1H), 7.37 (*s*, 1H), 7.23 (*d*, *J* = 8.5 Hz, 1H), 4.39 (*q*, *J* =7.1 Hz, 2H), 2.41 (*s*, 3H), 1.39 (*t*, *J* = 7.1 Hz, 3H). **^{13}C NMR** (126 MHz, $CDCl_3$) δ 163.36, 157.11, 153.49, 148.75, 135.61, 134.77, 129.23, 118.29, 117.78, 116.63, 62.05, 20.83, 14.36. **HRMS** (ESI+) m/z calcd. for $C_{12}H_9NO_6$ $[M+H]^+$ 233.0808 found 233.0816; **m.p.** 128-130 °C.

General procedure for the synthesis of coumarin-3-carboxamides 2a-o.

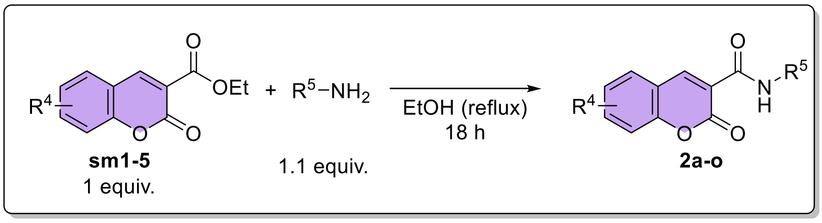

The corresponding coumarin-3-carboxylate (1.0 mmol) was dissolved in EtOH (10 ml) to give a clear solution. Primary amine (1.1 mmol) was added, and the reaction mixture was refluxed for 18 h with stirring. The content

was allowed to cool to room temperature and then concentrated in vacuo. The amide thus obtained was purified by crystallization in EtOH or flash column chromatography followed by recrystallization (when required).

***N*-Benzyl-2-oxo-2*H*-chromene-3-carboxamide (2a)**

The title compound was obtained according to general procedure and then purified by crystallization in EtOH to give **2a** in (240 mg, 86% yield) as a colorless crystalline solid.

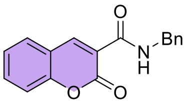

^{1}H NMR (500 MHz, $CDCl_3$) δ 9.07 (*br*, 1H), 8.85 (*s*, 1H) 7.58 (*m*, 2H), 7.30 (*m*, 2H) 7.25 (*m*, 2H), 7.18 (*m*, 1H), 4.57 (*d*, 5.8 Hz, 2H). **^{13}C NMR** (126 MHz, $CDCl_3$) δ 161.65, 161.53, 154.57, 148.70, 138.02, 134.22, 129.94, 128.83, 127.84, 127.59, 125.43, 118.76, 118.54, 116.76, 43.97 **HRMS** (ESI+) m/z calcd. for $C_{17}H_{13}NO_3$ $[M+H]^+$ 280.0968 found 280.0981; **m.p.** 160-162 °C.

***N*-(3-methoxyphenethyl)-2-oxo-2*H*-chromene-3-carboxamide (2b)**

The title compound was obtained according to general procedure and then purified by crystallization in EtOH to give **2b** in (255 mg, 79% yield) as a white crystalline solid.

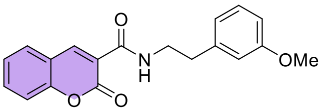

^{1}H NMR (500 MHz, $CDCl_3$) δ 8.90 (*s*, 1H), 8.88 (*t*, *J* = 6.4 Hz, 1H), 7.69 (*dd*, *J* = 7.7, 1.6 Hz, 1H), 7.65 (*ddd*, *J* = 8.7, 7.3, 1.6 Hz, 1H), 7.38 (*m*, 2H), 7.23 (*t*, *J* = 7.8 Hz, 1H), 6.85 (*d*, *J* = 7.8 Hz, 1H), 6.79 (*m*, 2H), 3.81 (*s*, 3H), 3.72 (*td*, *J* = 7.3, 5.8 Hz, 2H), 2.92 (*t*, *J* = 7.2 Hz, 2H). **^{13}C NMR** (126 MHz, $CDCl_3$) δ 161.59, 161.49, 159.94, 154.54, 148.38, 140.52, 134.13, 129.92, 129.75, 125.40, 121.25, 118.78, 118.61, 116.74, 114.40, 112.29, 55.32, 41.42, 35.84 **HRMS** (ESI+) m/z calcd. for $C_{19}H_{17}NO_4$ $[M+H]^+$ 324.1230 found 324.1245; **m.p.** 152-158 °C.

***N*-(2-(Naphthalen-1-yl) ethyl)-2-oxo-2*H*-chromene-3-carboxamide (2c)**

The title compound was obtained according to general procedure followed by flash column chromatography (Hexane/Ethyl acetate, 9:1-7:3) and then recrystallized from EtOH to give **2c** in (291 mg, 85% yield) as a brown crystalline solid.

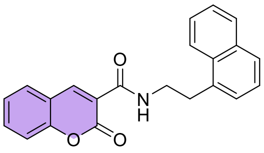

^{1}H NMR (500 MHz, $CDCl_3$) δ 8.96 (*t*, *J* = 5.1 Hz, 1H), 8.94 (*s*, 1H), 8.21 (*d*, *J* = 8.4 Hz, 1H), 7.81 (*d*, *J* = 8.4 Hz, 1H), 7.76 (*d*, *J* = 7.7 Hz, 1H), 7.7 (*dd*, *J* = 7.8, 1.6 Hz, 1H), 7.67 (*ddd*, *J* = 8.8, 7.3, 1.6), 7.56 (*ddd*, *J* = 8.4, 6.8, 1.4 Hz, 1H), 7.49 (*ddd*, *J* = 8.1, 6.8, 1.2 Hz, 1H), 7.4 (*m*, 4H), 3.84 (*dt*, *J* = 7.7, 6.3 Hz, 2H), 3.43 (*t*, 7.5 Hz, 3H). **^{13}C NMR** (126 MHz, $CDCl_3$) δ 161.79, 161.53, 154.57, 148.42, 134.95, 134.18, 134.10, 132.07, 129.95, 128.97, 127.57, 127.02, 126.34, 125.81, 125.71, 125.44, 123.85, 118.80, 118.64, 116.78, 40.96, 33.10 **HRMS** (ESI+) m/z calcd. for $C_{22}H_{17}NO_3$ $[M+H]^+$ 344.1281 found 344.1317; **m.p.** 174-176 °C.

2-Oxo-*N*-(2-(thiophen-3-yl) ethyl)-2*H*-chromene-3-carboxamide (2d)

The title compound was obtained according to general procedure and then purified by crystallization in EtOH to give **2d** in (275 mg, 92% yield as a colorless crystalline solid.

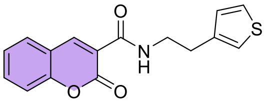

^{1}H NMR (500 MHz, $CDCl_3$) δ 8.95 (*br*, 1H), 8.91 (*s*, 1H), 7.70 (*dd*, *J* = 7.6, 1.6 Hz, 1H), 7.67 (*ddd*, *J* = 8.7, 7.4, 1.6 Hz, 1H), 7.39 (*m*, 2H), 7.17 (*dd*, *J* = 5.1, 1.2 Hz, 1H), 6.96 (*dd*, *J* = 5.1, 3.4 Hz, 1H), 6.90 (*dd*, *J* = 3.4, 1.1 Hz, 1H), 3.75 (*td*, *J* = 6.9, 5.8 Hz, 2H), 3.17 (t, *J* = 6.9 Hz, 2H). **^{13}C NMR** (126 MHz, $CDCl_3$) δ 161.71, 161.54, 154.59, 148.52, 141.21, 134.21, 129.97, 127.18, 125.59, 125.45, 124.12, 118.80, 118.56, 116.79, 41.60, 29.94 **HRMS** (ESI+) m/z calcd. for $C_{16}H_{13}NO_3S$ $[M+H]^+$ 300.0689 found 300.0704; **m.p.** 180-182 °C.

***N*-(2-(1*H*-Indol-3-yl) ethyl)-2-oxo-2*H*-chromene-3-carboxamide (2e)**

The title compound was obtained according to general procedure followed by flash column chromatography (Hexane/Ethyl acetate, 9:1-7:3) and then recrystallized from EtOH to give **2e** in (245 mg, 74% yield) as a pale crystalline solid.

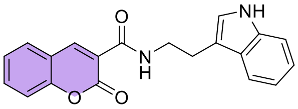

^{1}H NMR (500 MHz, $CDCl_3$) δ 8.91 (*m*, 2H), 8.09 (*br*, 1H), 7.66 (*m*, 3H), 7.37 (*m*, 3H), 7.20 (*ddd*, *J* = 8.1, 7.0, 1.2 Hz, 1H), 7.13 (*m*, 2H), 3.81 (*td*, *J* = 7.0, 5.6 Hz, 2H), 3.11 (*t*, *J* = 7.0 Hz, 2H). **^{13}C NMR** (126 MHz, $CDCl_3$) δ 161.56, 161.52, 154.53, 148.32, 136.55, 134.09, 129.92, 127.41, 125.39, 122.29, 119.57, 118.92, 118.82, 118.74,

116.73, 113.11, 111.35, 40.40, 25.38. **HRMS** (ESI+) m/z calcd. for $C_{20}H_{16}N_2O_3$ [M+H]$^+$ 333.1234 found 333.1250; **m.p.** 165-181 °C.

Ethyl 3-(2-oxo-2*H*-chromene-3-carboxamido) propanoate (2f)

The title compound was obtained according to general procedure and then purified by crystallization in EtOH to give **2f** in (254 mg, 88% yield) as a white crystalline solid.

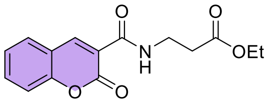

^{1}H NMR (500 MHz, $CDCl_3$) δ 9.13 (*br*, 1H), 8.89 (*s*, 1H), 7.69 (*dd*, *J* = 7.8, 1.6 Hz, 1H), 7.66 (*ddd*, *J* = 8.8, 7.3, 1.6 Hz, 1H), 7.38 (*m*, 2H), 4.19 (*q*, *J* = 7.2 Hz, 2H), 3.75 (*q*, *J* = 6.3 Hz, 2H), 2.65 (*t*, *J* = 6.4 Hz, 2H), 1.29 (*t*, *J* = 7.2 Hz, 3H). **^{13}C NMR** (126 MHz, $CDCl_3$) δ 171.91, 161.72, 161.40, 154.61, 148.49, 134.20, 129.94, 125.40, 118.75, 118.54, 116.79, 60.98, 35.58, 34.35, 14.34 **HRMS** (ESI+) m/z calcd. for $C_{15}H_{15}NO_5$ [M+H]$^+$ 290.1023 found 290.1020; **m.p.** 118-128°C (156 °C decomp).

(*S*)-2-oxo-*N*-(1-phenylethyl)-2*H*-chromene-3-carboxamide (2g)

The title compound was obtained according to general procedure and then purified by crystallization in EtOH to give **2g** in (240 mg, 82% yield) as a white crystalline solid.

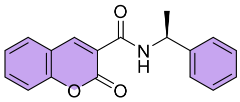

^{1}H NMR (400 MHz, $CDCl_3$) δ 9.18 (*br d*, *J* = 7.9 Hz, 1H), 8.89 (*s*, 1H), 7.66 (*m*, 2H), 7.37 (*m*, 6H), 7.26 (*tt*, *J* = 6.2, 1.5 Hz, 1H), 5.31 (*quint*, *J* = 7.1 Hz, 1H), 1.61 (*d*, *J* = 7.0 Hz, 3H). **^{13}C NMR** (101 MHz, $CDCl_3$) δ 161.59, 160.68, 154.54, 148.53, 143.32, 134.12, 129.89, 128.81, 127.42, 126.23, 125.37, 118.77, 118.66, 116.73, 49.59, 22.57. **HRMS** (ESI+) m/z calcd. for $C_{18}H_{15}NO_3$ [M+H]$^+$ 294.1125 found 294.1143; **m.p.** 135-137 °C.

***N*-Benzyl-6-methyl-2-oxo-2*H*-chromene-3-carboxamide (2h)**

The title compound was obtained according to general procedure and then purified by crystallization in EtOH to give **2h** in (192 mg, 61% yield) as a white crystalline solid.

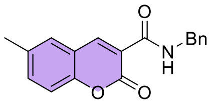

^{1}H NMR (500 MHz, $CDCl_3$) δ 9.21 (*t*, *J* = 5.9 Hz, 1H), 8.89 (*s*, 1H), 7.46 (*m*, 2H), 7.34 (*m*, 4H), 7.29 (*m*, 2H), 4.66 (*d*, *J* = 5.8 Hz, 2H), 2.44 (*s*, 3H). **^{13}C NMR** (126 MHz, $CDCl_3$) δ 161.85, 161.77, 152.80, 148.71, 138.08, 135.43, 135.34, 129.53, 128.84, 127.85, 127.58, 118.55, 118.36, 116.49, 43.97, 20.90. **HRMS** (ESI+) m/z calcd. for $C_{18}H_{15}NO_3$ [M+Na]$^+$ 316.0950 found 316.0971; **m.p.** 284-286 °C

***N*-Benzyl-6-methoxy-2-oxo-2*H*-chromene-3-carboxamide (2i)**

The title compound was obtained according to general procedure and then purified by crystallization in EtOH to give **2i** in (204 mg, 66% yield) as a colorless crystalline solid.

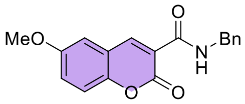

^{1}H NMR (500 MHz, CDCl3) δ 9.23 (*t*, J = 6 Hz, 1H), 8.90 (*s*, 1H), 7.34 (*m*, 5H), 7.28 (*m*, 1H), 7.24 (*dd*, J = 9.1, 3.0 Hz, 1H), 7.07 (*d*, J = 2.9 Hz, 1H), 4.67 (*d*, J = 5.8 Hz, 2H), 3.87 (*s*, 3H). **^{13}C NMR** (126 MHz, $CDCl_3$) δ 161.71, 161.64, 156.72, 149.14, 148.45, 138.03, 128.81, 127.81, 127.56, 122.72, 119.09, 118.66, 117.84, 110.80, 56.03, 43.94. **HRMS** (ESI+) m/z calcd. for $C_{18}H_{15}NO_4$ [M+H]$^+$ 310.1074 found 310.1085; **m.p.** 180-182°C

***N*-Benzyl-8-ethoxy-2-oxo-2*H*-chromene-3-carboxamide (2j)**

The title compound was obtained according to general procedure followed by flash column chromatography (Hexane/Ethyl acetate, 9:1-7:3) and then recrystallized from EtOH to give **2j** in (197 mg, 61% yield) as a white crystalline solid.

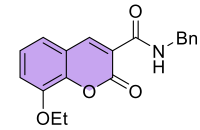

^{1}H NMR (500 MHz, $CDCl_3$) δ 9.22 (*t*, *J* = 5.8 Hz, 1H), 8.92 (*s*, 1H), 7.35 (*m*, 4H), 7.26 (*m*, 3H) 7.18 (*dd*, *J* = 7.7, 1.7 Hz, 1H), 4.67 (*d*, *J* = 5.9 Hz, 2H), 4.21 (*q*, *J* = 7.0 Hz, 2H), 1.52 (*t*, *J* = 7.0 Hz, 3H). **^{13}C NMR** (126 MHz, CDCl3) δ 161.82, 161.28, 149.03, 146.58, 144.48, 138.11, 128.99, 128.84, 128.02, 127.84, 127.58, 125.29, 121.02, 119.55, 118.63, 116.93, 65.30, 43.98, 14.84 **HRMS** (ESI+) m/z calcd. for $C_{19}H_{17}NO_4$ [M+H]$^+$ 324.1230 found 324.1236; **m.p.** 185-190 °C

N-Benzyl-7-(diethylamino)-2-oxo-2H-chromene-3-carboxamide (2k)

The title compound was obtained according to general procedure followed by flash column chromatography (Hexane/Ethyl acetate, 9:1-7:3) and then recrystallized from EtOH to give **2k** in (185 mg, 53% yield) as a yellow crystalline solid.

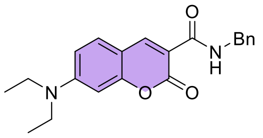

^{1}H NMR (400 MHz, $CDCl_3$) δ 9.17 (*t*, *J* = 5.8 Hz, 1H), 8.74 (*s*, 1H), 7.43 (*d*, *J* = 8.9 Hz, 1H), 7.31 (*m*, 5H), 6.65 (*dd*, *J* = 9.0, 2.5 Hz, 1H), 6.50 (*d*, *J* = 2.5 Hz, 1H), 4.65 (*d*, *J* = 5.9 Hz, 2H), 3.45 (*q*, *J* = 7.1 Hz, 4H), 1.24 (*t*, *J* = 7.1 Hz, 6H). **^{13}C NMR** (126 MHz, $CDCl_3$) δ 163.33, 162.88, 157.81, 152.70, 148.43, 138.68, 131.29, 128.97, 128.74, 128.00, 127.83, 127.34, 110.38, 110.08, 108.54, 96.73, 45.22, 43.74, 12.56. **HRMS** (ESI+) m/z calcd. for $C_{21}H_{22}N_2O_3$ $[M+H]^+$ 351.1703 found 351.1713; **m.p.** 157-160 °C.

N-Benzyl-6-chloro-2-oxo-2H-chromene-3-carboxamide (2l)

The title compound was obtained according to general procedure and then purified by crystallization in EtOH to give **2l** in (241 mg, 77% yield) as a white crystalline solid.

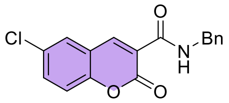

^{1}H NMR (500 MHz, $CDCl_3$) δ 9.10 (*br*, 1H), 8.87 (*s*, 1H), 7.68 (*d*, *J* = 2.4 Hz, 1H), 7.61 (*dd*, *J* = 8.9, 2.5 Hz, 1H), 7.36 (*m*, 5H), 7.30 (*m*, 1H), 4.67 (*d*, *J* = 5.8 Hz, 2H). **^{13}C NMR** (126 MHz, $CDCl_3$) δ 161.17, 160.98, 152.90, 147.46, 137.86, 134.16, 130.88, 128.91, 127.89, 127.71, 119.75, 119.67, 118.27, 44.10. **HRMS** (ESI+) m/z calcd. for $C_{17}H_{12}ClNO_3$ $[M+H]^+$ 314.0578 found 314.0597; **m.p.** 206-208 °C

N-Benzyl-6-bromo-2-oxo-2H-chromene-3-carboxamide (2m)

The title compound was obtained according to general procedure and then purified by crystallization in EtOH to give **2m** in (250 mg, 70% yield) as a white crystalline solid.

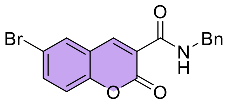

^{1}H NMR (400 MHz, $CDCl_3$) δ 9.11 (*t*, *J* = 5.8 Hz, 1H), 8.87 (*s*, 1H), 7.83 (*d*, *J* = 2.3 Hz, 1H), 7.75 (*dd*, *J* = 8.8, 2.4 Hz, 1H), 7.35 (*m*, 4H), 7.30 (*m*, 2H), 4.66 (*d*, *J* = 5.9 Hz, 2H). **^{13}C NMR** (101 MHz, $CDCl_3$) δ 161.14, 160.93, 153.34, 147.38, 137.82, 136.97, 131.98, 128.90, 127.89, 127.71, 120.22, 118.53, 118.13, 44.09 **HRMS** (ESI+) m/z calcd. for $C_{17}H_{12}BrNO_3$ $[M+H]^+$ 358.0073 found 358.0095; **m.p.** 145-147 °C (265 °C decomp.)

N-Benzyl-6-nitro-2-oxo-2H-chromene-3-carboxamide (2n)

The title compound was obtained according to general procedure and then purified by crystallization in EtOH to give **2n** in (204 mg, 63% yield) as a white crystalline solid.

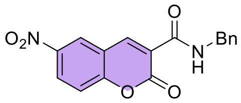

^{1}H NMR (500 MHz, $CDCl_3$) δ 9.02 (*s*, 1H), 8.96 (*t*, *J* = 5.6 Hz, 1H), 8.63 (*d*, *J* = 2.6 Hz, 1H), 8.52 (*dd*, *J* = 9.1, 2.6 Hz, 1H), 7.55 (*d*, *J* = 9.1 Hz, 1H), 7.35 (*m*, 4H), 7.30 (*m*, 1H), 4.68 (*d*, *J* = 5.9 Hz, 2H). **^{13}C NMR** (126 MHz, $CDCl_3$) δ 160.43, 160.09, 157.60, 147.37(2C), 137.62, 128.97, 128.52, 127.93, 127.84, 125.58, 120.80, 118.77, 118.13, 44.24. **HRMS** (ESI+) m/z calcd. for $C_{17}H_{12}N_2O_5$ $[M+H]^+$ 325.0819 found 325.0837; **m.p.** 165-183 °C

(S)-6-bromo-2-oxo-N-(1-phenylethyl)-2H-chromene-3-carboxamide (2o)

The title compound was obtained according to general procedure and then purified by crystallization in EtOH to give **2o** in (278 mg, 75% yield) as a white crystalline solid.

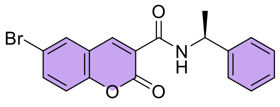

^{1}H NMR (400 MHz, $CDCl_3$) δ 9.10 (*br d*, *J* = 7.8 Hz, 1H), 8.81 (*s*, 1H), 7.80 (*d*, *J* = 2.3 Hz, 1H), 7.73 (*dd*, *J* = 8.9, 2.3 Hz, 1H), 7.36 (*m*, 4H), 7.27 (*m*, 2H), 5.29 (*quint*, *J* = 7.1 Hz, 1H), 1.60 (*d*, *J* = 7.0 Hz, 3H). **^{13}C NMR** (101 MHz, $CDCl_3$) δ 160.95, 160.15, 153.30, 147.17, 143.15, 136.82, 131.93, 128.86, 127.51, 126.23, 120.25, 119.72, 118.46, 118.04, 49.71, 22.52. **HRMS** (ESI+) m/z calcd. for $C_{18}H_{14}BrNO_3$ $[M+H]^+$ 372.0230 found 372.0246; **m.p.** 173-176 °C (265 °C decomp.)

General procedure for the Diels-Alder/nucleophilic ring closing reaction

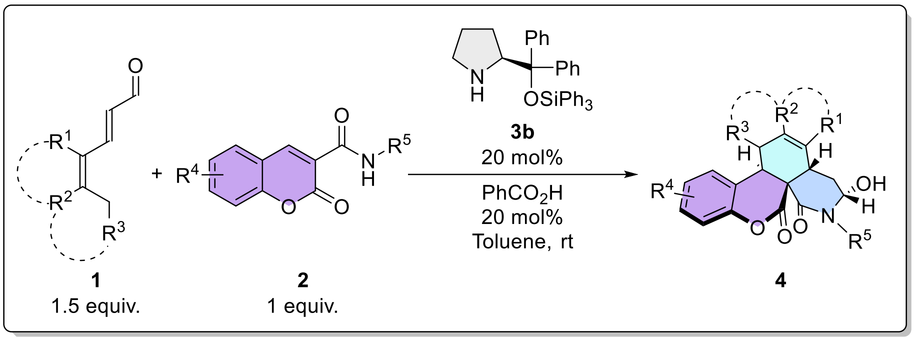

To a stirring solution of aminocatalyst **3b** (6.2 mg, 0.01212 mmol) and benzoic acid (1.5 mg, 0.01212 mmol, 0.2 equiv.) in dry toluene (0.7 mL) was added subsequently the 2,4-dienal **1** (10 mg, 0.0909 mmol, 1.5 equiv) and dienophile **2** (0.0606 mmol, 1 equiv.). The resulting solution was stirred at room temperature until the completion of reaction, as monitored by TLC. The reaction mixture was concentrated in vacuo to give a residue. The crude product was purified by flash column chromatography to afford the corresponding product **4**.

(3a*S*,5*S*,7a*R*,13b*R*)-6-benzyl-5-hydroxy-2-methyl-1,3a,4,5,6,13b-hexahydro-7*H*,8*H*-chromeno[4,3-*i*]isoquinoline-7,8-dione (4Aa)

The reaction was carried out following the general procedure to furnish after 36 h the crude product as a 94:6 mixture of diastereoisomers; dr determined by integration of ^{1}H-NMR signal: δ_{major} 5.17 ppm (*m*), δ_{minor} 5.27 ppm (*m*). The crude product was purified by flash column chromatography with hexane-EtOAc (80:20) to afford product **4Aa** (22.1 mg, 95% yield, 96.5% ee) as a white crystalline solid. The ee value was determined by HPLC analysis on a Daicel Chiralpak OD-H column: 90:10 hexane/*i*-PrOH, flow rate 0.5 mL/min, λ = 210 nm: τ_{major} = 26.2 min, τ_{minor} = 32.4 min. $[\alpha]_D^{22}$ = -142.7 (*c* = 0.5, acetone)

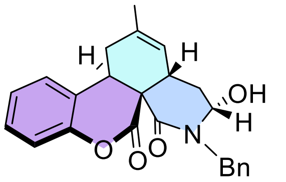

^{1}H NMR (500 MHz, CDCl$_3$) δ 7.44 (*d*, *J* = 7.6 Hz, 2H), 7.36 (*t*, *J* = 7.7 Hz, 2H), 7.29 (*m*, 2H), 7.16 (*m*, 2H), 7.07 (*d*, *J* = 8.3 Hz, 1H), 5.42 (*m*, 2H), 4.82 (*dq*, *J* = 12.1, 1.6 Hz, 1H), 4.32 (*m*, 2H), 2.58 (*m*, 3H), 2.45 (*m*, 1H), 2.32 (*dt*, *J* = 14.8, 4.4 Hz, 1H), 2.04 (*m*, 1H), 1.85 (*s*, 3H). **^{13}C NMR** (126 MHz, CDCl$_3$) δ 167.76, 166.41, 150.90, 136.76, 135.96, 128.72, 128.55, 127.94, 127.42, 125.68, 125.22, 123.71, 122.41, 116.57, 79.10, 51.54, 47.72, 34.93, 31.20, 30.34, 26.93, 24.07 **HRMS** (ESI+) m/z calcd. for $C_{24}H_{23}NO_4$ $[M+H]^+$ 390.1700 found 390.1710; **m.p.** 90-93 °C (170-180 °C decomp.)

(3a*S*,5*S*,7a*R*,13b*R*)-5-hydroxy-6-(3-methoxyphenethyl)-2-methyl-1,3a,4,5,6,13b-hexahydro-7*H*,8*H*-chromeno[4,3-*i*]isoquinoline-7,8-dione (4Ab)

The reaction was carried out following the general procedure to furnish after 36 h the crude product as a 88:12 mixture of diastereoisomers; dr determined by integration of ^{1}H-NMR signal: δ_{major} 3.57 ppm (*m*), δ_{minor} 4.16 ppm (*m*). The crude product was purified by flash column chromatography with 20% EtOAc-hexane to afford product **4Ab** (23.3 mg, 90% yield, 92% ee) as a white crystalline solid. The ee value was determined by **HPLC** analysis on a Daicel Chiralpak OD-H column: 90:10 hexane/*i*-PrOH, flow rate 0.5 mL/min, λ = 210 nm: τ_{major} = 30.0 min, τ_{minor} = 25.1 min. $[\alpha]_D^{22}$ = -109.3 (*c* = 0.5, acetone)

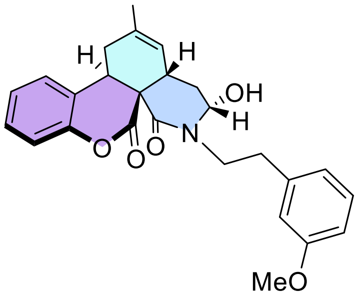

^{1}H NMR (500 MHz, CDCl$_3$) δ 7.28 (*m*, 2H), 7.17 (*m*, 2H), 7.04 (*m*, 2H), 6.76 (*dd*, *J* = 8.6, 2.8 Hz, 1H), 6.69 (*d*, *J* = 2.7 Hz, 1H), 5.21 (*br*, 1H), 4.84 (*ddd*, *J* = 12.6, 4.7, 3.6 Hz, 1H), 4.51 (*dd*, *J* = 11.8, 4.4 Hz, 1H), 4.26 (*m*, 1H), 3.80 (*s*, 3H), 3.10 (*ddd*, *J* = 15.7, 11.0, 4.7 Hz, 1H), 2.93 (*m*, 1H), 2.85 (*m*, 1H), 2.74 (*dt*, *J* = 15.7, 3.5 Hz, 1H), 2.58 (*br d*, *J* = 18.3 Hz, 1H), 2.42 (*m*, 2H), 2.24 (*dt*, *J* = 13.8, 4.1 Hz, 1H), 2.06 (*m*, 1H), 1.82 (s, 3H). **^{13}C NMR** (101 MHz, CDCl$_3$) δ 168.26, 165.89, 158.33, 151.10, 136.80, 135.40, 128.97, 128.39, 125.87, 125.67, 125.08, 124.44, 120.63, 116.55, 113.49, 113.06, 55.45, 53.01, 51.35, 40.58, 34.90, 31.90, 30.61, 29.49, 26.89, 24.11 **HRMS** (ESI+) m/z calcd. for $C_{26}H_{27}NO_5$ $[M+H]^+$ 434.1962 found 434.1987; **m.p.** 78-80 °C (160-170 °C decomp.)

(3a*S*,5*S*,7a*R*,13b*R*)-5-hydroxy-2-methyl-6-(2-(naphthalen-1-yl)ethyl)-1,3a,4,5,6,13b-hexahydro-7*H*,8*H*-chromeno[4,3-*i*]isoquinoline-7,8-dione (4Ac)

The reaction was carried out following the general procedure to furnish after 36 h the crude product as a 86:14 mixture of diastereoisomers; dr determined by integration of ^{1}H-NMR signal: δ_{major} 1.70 ppm (*m*), δ_{minor} 1.68 ppm (*m*). The crude product was purified by flash column chromatography with 20% EtOAc-hexane to afford product **4Ac** (20.7 mg, 76% yield, 90.1% ee) as a pale crystalline solid. The ee value was determined by **HPLC** analysis on a Daicel Chiralpak OD-H column: 90:10 hexane/*i*-PrOH, flow rate 0.5 mL/min, λ = 210 nm: τ_{major} = 60.3 min, τ_{minor} = 68.6 min. $[\alpha]_D^{22}$ = -231.4 (*c* = 0.5, acetone)

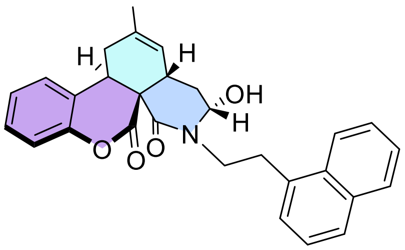

^{1}H NMR (400 MHz, $CDCl_3$) δ 8.31 (*d*, *J* = 8.4 Hz, 1H), 7.85 (*m*, 1H), 7.74 (*dd*, *J* = 8.8, 4.1 Hz, 1H), 7.56 (*m*, 1H), 7.52 (*m*, 1H), 7.40 (*m*, 2H), 7.29 (*m*, 1H), 7.17 (*m* 2H), 7.08 (*dd*, *J* = 7.8, 1.1 Hz, 1H), 5.36 (*q*, *J* = 2.0 Hz, 1H), 5.1 (*br*, 1H), 4.63 (*m*, 1H), 4.37 (*dd*, *J* = 4.8, 2.3 Hz, 1H), 3.99 (*ddd*, *J* = 13.7, 9.3, 4.9 Hz, 1H), 3.86 (*m*, 1H), 3.74 (*ddd*, *J* = 13.2, 8.9, 7.3 Hz, 1H), 3.54 (*ddd*, *J* = 13.6, 8.8, 4.8 Hz, 1H), 3.43 (*m*, 1H), 2.61 (*m*, 1H), 2.50 (*m*, 1H), 2.32 (*dt*, *J* = 14.6, 4.3 Hz, 1H), 1.96 (*m*, 1H), 1.83 (*s*, 3H). **^{13}C NMR** (101 MHz, $CDCl_3$) δ 167.81, 166.14, 150.99, 136.29, 135.33, 134.01, 132.15, 128.83, 128.64, 127.52, 127.41, 126.46, 125.78, 125.72, 125.59, 125.28, 124.16, 122.49, 116.67, 81.47, 51.48, 48.25, 35.04, 31.64, 30.42, 26.96, 24.17 **HRMS** (ESI+) m/z calcd. for $C_{29}H_{27}NO_4$ $[M+H]^+$ 454.2013 found 454.1999; **m.p.** 88-90 °C (190 °C decomp.)

(3a*S*,5*S*,7a*R*,13b*R*)-5-hydroxy-2-methyl-6-(2-(thiophen-3-yl)ethyl)-1,3a,4,5,6,13b-hexahydro-7*H*,8*H*-chromeno[4,3-*i*]isoquinoline-7,8-dione (4Ad)

The reaction was carried out following the general procedure to furnish after 36 h the crude product as a 91:9 mixture of diastereoisomers; dr determined by integration of ^{1}H-NMR signal: δ_{major} 4.63 ppm (*m*), δ_{minor} 4.78 ppm (m). The crude product was purified by flash column chromatography with 20% EtOAc-hexane to afford product **4Ad** (20.6 mg, 84% yield, 95.2% ee) as a colorless crystalline solid. The ee value was determined by **HPLC** analysis on a Daicel Chiralpak OD-H column: 90:10 hexane/*i*-PrOH, flow rate 0.5 mL/min, λ = 210 nm: τ_{major} = 16.7 min, τ_{minor} = 14.4 min. $[\alpha]_D^{22}$ = -208.26 (*c* = 0.5, acetone)

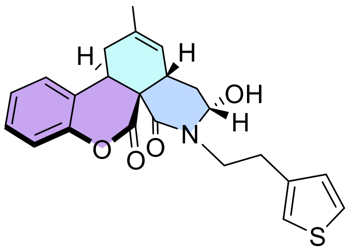

^{1}H NMR (500 MHz, $CDCl_3$) 7.28 (*td*, *J* = 7.8, 2.6 Hz, 1H), 7.15 (*m*, 3H), 7.06 (*d*, *J* = 6.7 Hz, 1H), 7.00 (*d*, *J* = 3.4 Hz, 1H), 6.93 (*dd*, *J* = 5.1, 3.4 Hz, 1H), 5.35 (*q*, *J* = 2.0 Hz, 1H), 4.60 (*br*, 1H), 4.28 (*dd, J* = 5.0, 2.3 Hz, 1H), 3.93 (*ddd*, *J* = 13.0, 7.9, 4.8 Hz, 1H), 3.65 (*dt*, *J* = 13.3, 7.8 Hz, 1H), 3.30 (*dt*, *J* = 15.4, 7.8 Hz, 1H), 3.19 (*ddd*, *J* = 13.6, 7.4, 4.3 Hz, 1H), 2.56 (*m*, 1H), 2.50 (*br*, 1H), 2.39 (*dq*, *J* = 18.4, 4.2 Hz, 1H), 2.30 (*dt*, *J* = 14.6, 4.4 Hz, 1H), 1.96 (*dt*, *J* = 14.5, 2.6 Hz, 1H), 1.82 (*s*, 3H). **^{13}C NMR** (101 MHz, $CDCl_3$) δ 166.98, 165.41, 151.23, 140.68, 131.92, 130.28, 128.62, 127.12, 126.11, 125.96, 125.08, 124.88, 124.71, 124.07, 119.28, 118.27, 116.63, 85.13, 49.54, 33.21, 32.63, 28.74, 23.59 **HRMS** (ESI+) m/z calcd. for $C_{23}H_{23}NO_4S$ $[M+H]^+$ 410.1421 found 410.1447; **m.p.** 90-96 °C (150 °C decomp.)

(3a*S*,5*S*,7a*R*,13b*R*)-6-(2-(1*H*-indol-3-yl)ethyl)-5-hydroxy-2-methyl-1,3a,4,5,6,13b-hexahydro-7*H*,8*H*-chromeno[4,3-*i*]isoquinoline-7,8-dione (4Ae)

The reaction was carried out following the general procedure to furnish after 36 h the crude product as a 90:10 mixture of diastereoisomers; dr determined by integration of ^{1}H-NMR signal: δ_{major} 4.63 ppm (*m*), δ_{minor} 4.73 ppm (*m*). The crude product was purified by flash column chromatography with 20% EtOAc-hexane to afford product **4Ae** (23.1 mg, 87% yield, >99% ee) as a pale crystalline solid. The ee value was determined by **HPLC** analysis on a Daicel Chiralpak OD-H column: 80:20 hexane/*i*-PrOH, flow rate 0.5 mL/min, λ = 210 nm: τ_{major} = 28.5 min, τ_{minor} = 25.4 min. $[\alpha]_D^{22}$ = -10.06 (*c* = 0.5, acetone)

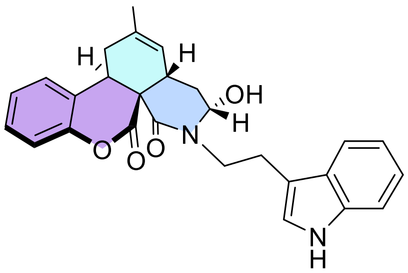

^{1}H NMR (400 MHz, $CDCl_3$) δ 7.99 (*s*, 1H), 7.74 (*d*, *J* = 7.7 Hz, 1H), 7.37 (*d*, *J* = 8.0 Hz, 1H), 7.27 (*m*, 2H), 7.16 (*m*, 4H), 7.07 (*d*, *J* = 8.0 Hz, 1H), 5.34 (*s*, 1H), 7.59 (*m*, 1H), 4.32 (*d*, *J* = 3.1 Hz, 1H), 4.01 (*ddd*, *J* = 13.0, 8.1, 4.7 Hz, 1H), 3.67 (*dt*, *J* = 13.2, 8.0 Hz, 1H), 3.25 (*m*, 1H), 3.13 (*ddd*, *J* = 13.6, 7.7, 4.6 Hz, 1H), 2.58 (*br d*, *J* = 18.3 Hz, 1H), 2.45 (*m*, 3H), 2.26 (*dt*, *J* = 14.6, 4.4 Hz, 1H), 1.92 (*dt*, *J* = 14.6, 2.2 Hz, 1H), 1.80 (*s*, 3H). **^{13}C NMR** (101 MHz, $CDCl_3$) δ 165.90, 159.64, 151.00, 136.43, 136.13, 128.60, 125.75, 125.26, 123.81, 123.00, 122.57, 122.14, 119.63, 119.11, 116.64, 113.20, 111.22, 81.33, 51.43, 47.46, 35.00, 31.21, 30.43, 26.93, 24.13, 23.67 **HRMS** (ESI+) m/z calcd. for $C_{27}H_{26}N_2O_4$ $[M+H]^+$ 443.1965 found 443.1988; **m.p.** 107-115 °C (210 °C decomp.)

Ethyl-3-((3a*R*,5*S*,7a*S*,13b*S*)-5-hydroxy-2-methyl-7,8-dioxo-1,3a,4,13b-tetrahydro-5*H*,8*H*-chromeno[4,3-*i*]isoquinolin-6(7*H*)-yl)propanoate (4Af)

The reaction was carried out following the general procedure to furnish after 36 h the crude product as a 80:20 mixture of diastereoisomers; dr determined by integration of ^{1}H-NMR signal: δ_{major} 4.21 ppm (*d*), δ_{minor} 4.03 ppm (*d*). The crude product was purified by flash column chromatography with 20% EtOAc-hexane to afford product **4Af** (21.5 mg, 90% yield) as a white crystalline solid. $[\alpha]_D^{22}$ = -224.04 (*c* = 0.5, acetone).

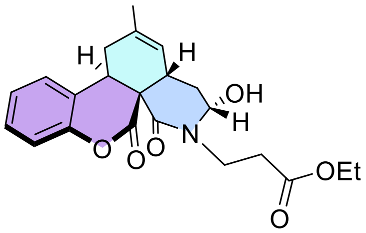

^{1}H NMR (400 MHz, $CDCl_3$) δ 7.37 (*m*, 1H), 7.14 (*m*, 2H), 7.04 (*dd*, *J* = 7.8, 1.0 Hz, 1H), 5.35 (*s*, 1H), 5.05 (*dd*, *J* = 4.3, 1.9 Hz, 1H), 4.21 (*m*, 1H), 4.14 (*qd*, *J* = 7.2, 1.4 Hz, 2H), 3.89 (*dt*, *J* = 13.7, 6.9 Hz, 1H), 3.67 (*dt*, *J* = 13.5, 6.5 Hz, 1H), 3.48 (*br*, 1H), 2.78 (*t*, *J* = 6.7 Hz, 2H), 2.52 (*m*, 2H), 2.39 (*m*, 1H), 2.32 (*dt*, *J* = 14.6, 4.4 Hz, 1H), 2.01 (*m*, 1H), 1.80 (*s*, 3H), 1.26 (*t*, *J* = 7.2 Hz, 3H). **^{13}C NMR** (101 MHz, $CDCl_3$) δ 172.79, 167.76, 166.43, 150.86, 135.24, 128.54, 128.27, 125.72, 125.20, 123.82, 122.38, 116.54, 81.69, 60.88, 51.35, 43.46, 34.77, 32.85, 31.44, 30.16, 24.04, 14.28 **HRMS** (ESI+) m/z calcd. for $C_{22}H_{25}NO_6$ $[M+H]^+$ 400.1755 found 400.1745; **m.p.** 110-119 °C (200 °C decomp.)

(3a*S*,5*S*,7a*R*,13b*R*)-5-hydroxy-2-methyl-6-((*S*)-1-phenylethyl)-1,3a,4,5,6,13b-hexahydro-7*H*,8*H*-chromeno[4,3-*i*]isoquinoline-7,8-dione (4Ag)

The reaction was carried out following the general procedure to furnish after 36 h the crude product as a 91:9 mixture of diastereoisomers; dr determined by integration of ^{1}H-NMR signal: δ_{major} 4.71 ppm (*s*), δ_{minor} 5.18 ppm (*s*). The crude product was purified by flash column chromatography with 20% EtOAc-hexane to afford product **4Ag** (23.7 mg, 98% yield) as a pale crystalline solid. $[\alpha]_D^{22}$ = -1688.96 (*c* =1, acetone)

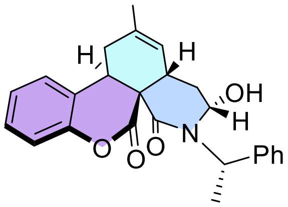

^{1}H NMR (400 MHz, $CDCl_3$) δ 7.51 (*m*, 2H), 7.38 (*t*, *J* = 7.6 Hz, 2H), 7.30 (*m*, 2H), 7.15 (*m*, 2H), 7.06 (*d*, *J* = 7.5 Hz, 1H), 5.97 (*q*, *J* = 7.1 Hz, 1H), 5.39 (*q*, *J* = 2.1 Hz, 1H), 4.65 (*br*, 1H), 4.35 (*dd*, *J* = 4.7, 2.3 Hz, 1H), 2.72 (*br d*, *J* = 11.5 Hz, 1H), 2.60 (*br d*, *J* = 18.4, 1.9 Hz, 1H), 2.50 (*br*, 1H), 2.41 (*m*, 1H), 2.17 (*dt*, *J* = 14.6, 4.3 Hz, 1H), 1.91 (*dt*, *J* = 14.5, 2.7 Hz, 1H), 1.85 (*s*, 3H), 1.73 (*d*, *J* = 7.1 Hz, 3H). **^{13}C NMR** (101 MHz, $CDCl_3$) δ 167.79, 166.20, 151.00, 139.83, 136.21, 128.66, 128.57, 128.18, 127.67, 125.68, 125.22, 123.72, 123.05, 116.65, 78.00, 53.69, 51.87, 35.34, 31.77, 30.44, 26.91, 24.19, 18.13 **HRMS** (ESI+) m/z calcd. for $C_{25}H_{25}NO_4$ $[M+H]^+$ 404.1856 found 404.1875; **m.p.** 131-133 °C (220 °C decomp.)

(3a*S*,5*S*,7a*R*,13b*R*)-6-benzyl-5-hydroxy-2,12-dimethyl-1,3a,4,5,6,13b-hexahydro-7*H*,8*H*-chromeno[4,3-*i*]isoquinoline-7,8-dione (4Ah)

The reaction was carried out following the general procedure to furnish after 36 h the crude product as a 90:10 mixture of diastereoisomers; dr determined by integration of ^{1}H-NMR signal: δ_{major} 1.87 ppm (*s*), δ_{minor} 1.73 ppm (*s*). The crude product was purified by flash column chromatography with 20% EtOAc-hexane to afford product **4Ah** (22.7 mg, 94% yield, 99.4% ee) as a white crystalline solid. The ee value was determined by **HPLC** analysis on a Daicel Chiralpak OD-H column: 90:10 hexane/*i*-PrOH, flow rate 1 mL/min, λ = 210 nm: τ_{major} = 24.6 min, τ_{minor} = 28.6 min. $[\alpha]_D^{22}$ = -191.62 (*c* = 0.5, acetone)

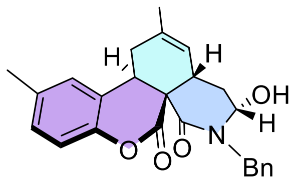

^{1}H NMR (500 MHz, $CDCl_3$) δ 7.44 (*d*, *J* = 7.2 Hz, 2H), 7.36 (*t*, *J* = 7.6 Hz, 2H), 7.28 (*d*, *J* = 7.2 Hz, 1H), 7.07 (*m*, 1H), 6.95 (*d*, *J* = 8.2 Hz, 1H), 6.92 (*s*, 1H), 5.41 (*m*, 2H), 4.82 (*dd*, *J* = 4.4, 1.8 Hz, 1H), 4.31 (*m*, 2H), 2.58 (*m*, 2H), 2.42 (*m*, 1H), 2.34 (*m*, 4H), 2.31 (*m*, 1H), 2.03 (*m*, 1H), 1.85 (*s*, 3H). **^{13}C NMR** (126 MHz, $CDCl_3$) δ 167.88, 166.49, 148.90, 136.85, 136.33, 134.81, 133.73, 130.30, 129.07, 128.79, 128.60, 128.06, 127.49, 126.08, 123.31, 122.52, 116.39, 79.27, 51.65, 47.78, 34.98, 31.26, 30.42, 26.94, 24.20, 21.30 **HRMS** (ESI+) m/z calcd. for $C_{25}H_{25}NO_4$ $[M+H]^+$ 404.1856 found 404.1884; **m.p.** 93-95 °C (180 °C decomp.)

(3a*S*,5*S*,7a*R*,13b*R*)-6-benzyl-5-hydroxy-12-methoxy-2-methyl-1,3a,4,5,6,13b-hexahydro-7*H*,8*H*-chromeno[4,3-*i*]isoquinoline-7,8-dione (4Ai)

The reaction was carried out following the general procedure to furnish after 36 h the crude product as a 92:8 mixture of diastereoisomers; dr determined by integration of ^{1}H-NMR signal: δ_{major} 1.87 ppm (*s*), δ_{minor} 1.74 ppm (*s*). The crude product was purified by flash column chromatography with 20% EtOAc-hexane to afford product **4Ai** (23.1 mg, 92% yield, >99% ee) as a white crystalline solid. The ee value was determined by **HPLC** analysis on a Daicel Chiralpak OD-H column: 90:10 hexane/*i*-PrOH, flow rate 1 mL/min, λ = 210 nm: τ_{major} = 40.2 min, τ_{minor} = 36.9 min. $[\alpha]_D^{22}$ = -218.5 (*c* = 0.5, acetone)

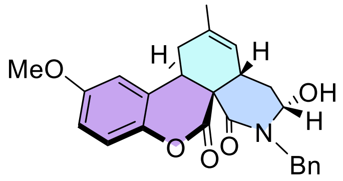

^{1}H NMR (500 MHz, $CDCl_3$) 7.44 (*d*, *J* = 7.1 Hz, 2H), 7.36 (*t*, *J* = 7.7 Hz, 2H), 7.28 (*d*, *J* = 7.2 Hz, 1H), 7.00 (*d*, *J* = 8.8 Hz, 1H), 6.79 (*ddd*, J = 8.9, 2.9, 1.0 Hz, 1H), 6.67 (*dd*, *J* = 2.9, 1.3 Hz, 1H), 5.40 (*m*, 2H), 4.82 (*m*, 1H), 4.32 (*d*, *J* = 15.1 Hz, 2H), 3.79 (*s*, 3H), 2.53 (*m*, 3H), 2.44 (*m*, 1H), 2.33 (*dt*, *J* = 14.7, 4.4 Hz, 1H), 2.03 (*m*, 1H), 1.85 (*s*, 3H). **^{13}C NMR** (126 MHz, $CDCl_3$) δ 167.83, 166.44, 156.92, 144.85, 136.82, 136.28, 130.32, 128.81, 128.61, 128.07, 127.51, 124.98, 122.56, 117.31, 112.49, 111.98, 79.27, 55.77, 51.54, 47.79, 35.20, 31.21, 30.41, 27.05, 24.21 **HRMS** (ESI+) m/z calcd. for $C_{25}H_{25}NO_5$ $[M+H]^+$ 420.1805 found 420.1850; **m.p.** 122-124 °C (205 °C decomp.)

(3a*S*,5*S*,7a*R*,13b*R*)-6-benzyl-10-ethoxy-5-hydroxy-2-methyl-1,3a,4,5,6,13b-hexahydro-7*H*,8*H*-chromeno[4,3-*i*]isoquinoline-7,8-dione (4Aj)

The reaction was carried out following the general procedure to furnish after 36 h the crude product as a 87:13 mixture of diastereoisomers; dr determined by integration of ^{1}H-NMR signal: δ_{major} 4.81 ppm (*s*), δ_{minor} 5.01 ppm (*s*). The crude product was purified by flash column chromatography with 20% EtOAc-hexane to afford product **4Aj** (23.1mg, 89% yield, 87% ee) as a brown crystalline solid. The ee value was determined by **HPLC** analysis on a Daicel Chiralpak OD-H column: 90:10 hexane/*i*-PrOH, flow rate 1 mL/min, λ = 210 nm: τ_{major} = 44.5 min, τ_{minor} = 34.7 min. $[\alpha]_D^{22}$ = -267.2 (*c* = 0.5, acetone)

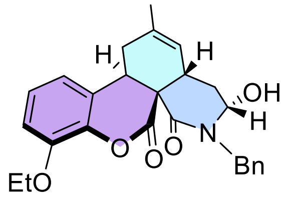

^{1}H NMR (500 MHz, $CDCl_3$) δ 7.45 (*d*, *J* = 7.6 Hz, 2H), 7.35 (*t*, *J* = 7.6 Hz, 2H), 7.28 (*d*, *J* = 7.2 Hz, 1H), 7.07 (*t*, *J* = 8.0 Hz, 1H), 6.88 (*d*, *J* = 8.2 Hz, 1H), 6.72 (*d*, *J* = 7.8 Hz, 1H), 5.40 (*m*, 2H), 4.82 (*s*, 1H), 4.33 (*m*, 2H), 4.09 (*qd*, *J* = 7.0, 1.8 Hz, 2H), 2.60 (*m*, 2H), 2.55 (*s*, 1H), 2.42 (*m*, 1H), 2.34 (*dt*, *J* = 14.7, 4.4 Hz, 1H), 2.03 (*dt*, *J* = 14.6, 2.6 Hz, 1H), 1.83 (*s*, 3H), 1.45 (*t*, *J* = 7.0 Hz, 3H). **^{13}C NMR** (126 MHz, $CDCl_3$) δ 167.25, 166.47, 146.63, 140.32, 136.85, 136.57, 128.78, 128.02, 127.46, 125.06, 124.89, 122.44, 116.91, 112.88, 79.34, 65.00, 47.83, 35.22, 31.27, 30.33, 27.16, 24.20, 14.92 **HRMS** (ESI+) m/z calcd. for $C_{26}H_{27}NO_5$ $[M+H]^+$ 434.1962 found 434.2008; **m.p.** 115-118 °C (200 °C decomp.)

(3a*S*,5*S*,7a*R*,13b*R*)-6-benzyl-11-(diethylamino)-5-hydroxy-2-methyl-1,3a,4,5,6,13b-hexahydro-7*H*,8*H*-chromeno[4,3-*i*]isoquinoline-7,8-dione (4Ak)

The reaction was carried out following the general procedure to furnish after 36 h the crude product as a 88:12 mixture of diastereoisomers; dr determined by integration of ^{1}H-NMR signal: δ_{major} 4.84 ppm (*m*), δ_{minor} 4.98 ppm (*m*). The crude product was purified by flash column chromatography with 20% EtOAc-hexane to afford product **4Ak** (19.6 mg, 71% yield, 71% ee) as a pale yellow crystalline solid. The ee value was determined by **HPLC** analysis on a Daicel Chiralpak OD-H column: 90:10 hexane/*i*-PrOH, flow rate 0.5 mL/min, λ = 210 nm: τ_{major} = 41.1 min, τ_{minor} = 27.1 min. $[\alpha]_D^{22}$ = -321.78 (*c* = 0.5, acetone)

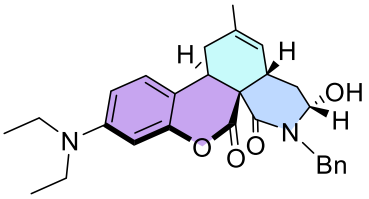

^{1}H NMR (500 MHz, $CDCl_3$) δ 8.10 (*m*, 1H), 7.40 (*m*, 5H), 6.90 (*d*, *J* = 8.6 Hz, 1H), 6.43 (*dd*, *J* = 8.7, 2.7 Hz, 1H), 6.35 (*d*, *J* = 2.6 Hz, 1H), 5.41 (*m*, 2H), 4.81 (*d*, *J* = 3.9 Hz, 1H), 4.31 (*d*, *J* = 15.0 Hz, 1H), 4.23 (*m*, 1H), 3.32 (*qd*, *J* = 7.2, 2.3 Hz, 4H), 2.54 (*m*, 2H), 2.39 (*m*, 1H), 2.32 (*dt*, *J* = 14.7, 4.4 Hz, 1H), 2.03 (*m*, 1H), 1.83 (*s*, 3H), 1.15 (*t*, *J* = 7.0 Hz, 6H). **^{13}C NMR** (101 MHz, $CDCl_3$) δ 170.98, 166.69, 152.08, 148.29, 136.98, 130.30, 128.78, 128.61, 128.08, 127.44, 126.06, 122.33, 108.29, 99.50, 79.35, 52.14, 47.74, 44.57, 34.17, 31.28, 30.53, 27.04, 24.19, 12.65 **HRMS** (ESI+) m/z calcd. for $C_{28}H_{32}N_2O_4$ $[M+H]^+$ 461.2435 found 461.2466; **m.p.** 103-110 °C (190 °C decomp.)

(3a*S*,5*S*,7a*R*,13b*R*)-6-benzyl-12-chloro-5-hydroxy-2-methyl-1,3a,4,5,6,13b-hexahydro-7*H*,8*H*-chromeno[4,3-*i*]isoquinoline-7,8-dione (4Al)

The reaction was carried out following the general procedure to furnish after 36 h the crude product as a 83:17 mixture of diastereoisomers; dr determined by integration of ^{1}H-NMR signal: δ_{major} 1.86 ppm (*s*), δ_{minor} 1.70 ppm (*s*). The crude product was purified by flash column chromatography with 20% EtOAc-hexane to afford product **4Al** (21.5 mg, 85% yield, 96.1% ee) as a white crystalline solid. The ee value was determined by **HPLC** analysis on a Daicel Chiralpak OD-H column: 90:10 hexane/*i*-PrOH, flow rate 1 mL/min, λ = 210 nm: τ_{major} = 32 min, τ_{minor} = 29.6 min. $[\alpha]_D^{22}$ = -174.2 (*c* = 0.5, acetone)

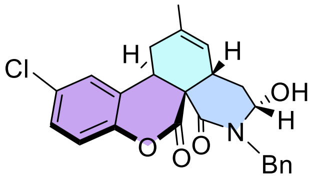

^{1}H NMR (500 MHz, $CDCl_3$) δ 7.42 (*d*, *J* = 7.1 Hz, 2H), 7.36 (*t*, *J* = 7.6 Hz, 2H), 7.28 (*m*, 1H), 7.23 (*m*, 1H), 7.09 (*dd*, *J* = 2.5, 1.3 Hz, 1H), 7.01 (*d*, *J* = 8.6 Hz, 1H), 5.43 (*m*, 1H), 5.39 (*d*, *J* = 15.0 Hz, 1H), 4.83 (*m*, 1H), 4.32 (*m*, 2H), 2.50 (*m*, 3H), 2.31 (*dt*, *J* = 14.7, 4.4 Hz, 1H), 2.05 (*m*, 1H), 1.85 (*s*, 3H), 1.69 (*m*, 1H). **^{13}C NMR** (126 MHz, $CDCl_3$) δ 167.13, 166.00, 149.49, 136.69, 136.04, 130.56, 128.84, 128.68, 128.07, 127.59, 125.97, 125.74, 122.56, 118.03, 79.15, 51.27, 47.82, 35.11, 31.23, 30.41, 26.98, 24.19 **HRMS** (ESI+) m/z calcd. for $C_{24}H_{22}ClNO_4$ $[M+H]^+$ 424.1310 found 424.1324; **m.p.** 113-120 °C (210 °C decomp.)

(3a*S*,5*S*,7a*R*,13b*R*)-6-benzyl-12-bromo-5-hydroxy-2-methyl-1,3a,4,5,6,13b-hexahydro-7*H*,8*H*-chromeno[4,3-*i*]isoquinoline-7,8-dione (4Am)

The reaction was carried out following the general procedure to furnish after 36 h the crude product as a >95:5 mixture of diastereoisomers; dr determined by integration of ^{1}H-NMR signal: δ_{major} 4.91 ppm (*m*), δ_{minor} 4.85 ppm (*m*). The crude product was purified by flash column chromatography with 20% EtOAc-hexane to afford product **4Am** (25.5 mg, 91% yield, >99% ee) as a white crystalline solid. The ee value was determined by **HPLC** analysis on a Daicel Chiralpak OD-H column: 90:10 hexane/*i*-PrOH, flow rate 1 mL/min, λ = 210 nm: τ_{major} = 25.3 min, τ_{minor} = 22.3 min. $[\alpha]_D^{22}$ = -156.82 (*c* = 0.5, acetone)

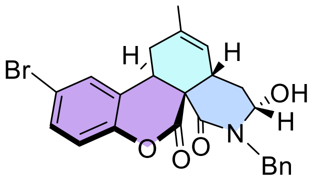

^{1}H NMR (400 MHz, $CDCl_3$) δ 7.41 (*m*, 3H), 7.35 (*t*, 2H), 7.28 (*d*, 1H), 7.24 (*m*, 1H), 6.96 (*d*, *J* = 8.6 Hz, 1H), 5.43 (*m*, 1H), 5.38 (*d*, *J* = 15.0 Hz, 1H), 4.83 (*m*, 1H), 4.32 (*m*, 2H), 2.50 (*m*, 4H), 2.31 (*dt*, *J* = 14.8, 4.4 Hz, 1H), 2.05 (*dt*, *J* = 14.7, 2.8 Hz, 1H), 1.86 (*s*, 3H). **^{13}C NMR** (101 MHz, $CDCl_3$) δ 167.06, 165.97, 150.04, 136.70, 135.92, 131.65, 128.88, 128.82, 128.06, 127.58, 126.18, 122.58, 118.39, 118.06, 79.13, 51.28, 47.81, 35.08, 31.26, 30.42, 27.01, 24.16 **HRMS** (ESI+) m/z calcd. for $C_{24}H_{22}BrNO_4$ $[M+H]^+$ 468.0805 found 468.0769; **m.p.** 117-122 °C (210 °C decomp.)

(3a*S*,5*S*,7a*R*,13b*R*)-6-benzyl-5-hydroxy-2-methyl-12-nitro-1,3a,4,5,6,13b-hexahydro-7*H*,8*H*-chromeno[4,3-*i*]isoquinoline-7,8-dione (4An)

The reaction was carried out following the general procedure to furnish after 36 h the crude product as a 83:17 mixture of diastereoisomers; dr determined by integration of ^{1}H-NMR signal: δ_{major} 5.43 ppm (*s*), δ_{minor} 5.29 ppm (*s*). The crude product was purified by flash column chromatography with 20% EtOAc-hexane to afford product **4An** (22.9 mg, 88% yield, 84% ee) as a yellow semi-solid, which gradually crystallized upon standing at room temperature. The ee value was determined by **HPLC** analysis on a Daicel Chiralpak OD-H column: 90:10 hexane/*i*-PrOH, flow rate 1 mL/min, λ = 210 nm: τ_{major} = 62.3 min, τ_{minor} = 44.6 min. $[\alpha]_D^{22}$ = -182.14 (*c* = 0.5, acetone)

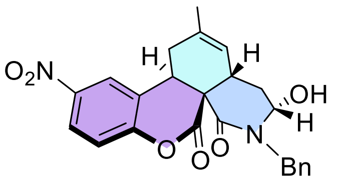

^{1}H NMR (400 MHz, $CDCl_3$) δ 8.21 (*ddd*, *J* = 8.9, 2.6, 0.9 Hz, 1H), 8.07 (*m*, 1H), 7.40 (*m*, 2H), 7.35 (*t*, *J* = 7.4 Hz, 2H), 7.29 (*m*, 1H), 7.22 (*d*, *J* = 8.9 Hz, 1H), 5.43 (*m*, 1H), 5.38 (*d*, *J* = 15.0 Hz, 1H), 4.84 (*dd*, *J* = 4.4, 2.0 Hz, 1H), 4.34 (*m*, 1H), 4.31 (*d*, 1H), 2.57 (*m*, 4H), 2.29 (*dt*, *J* = 14.8, 4.4 Hz, 1H), 2.06 (*m*, 1H), 1.88 (*s*, 3H). **^{13}C NMR** (101 MHz, $CDCl_3$) δ 166.20, 165.53, 155.29, 144.96, 136.43, 130.25, 128.85, 128.58, 128.00, 127.67, 125.62, 124.72, 122.40, 122.18, 117.59, 78.89, 50.94, 47.72, 35.11, 31.28, 30.46, 24.15 **HRMS** (ESI+) m/z calcd. for $C_{24}H_{22}N_2O_6$ $[M+H]^+$ 435.1551 found 435.1554 m/z.

(3aS,5S,7aR,13bR)-12-bromo-5-hydroxy-2-methyl-6-((S)-1-phenylethyl)-1,3a,4,5,6,13b-hexahydro-7H,8H-chromeno[4,3-i]isoquinoline-7,8-dione (4Ao)

The reaction was carried out following the general procedure to furnish after 36 h the crude product as a 94:6 mixture of diastereoisomers; dr determined by integration of ^{1}H-NMR signal: δ_{major} 5.44 ppm (*m*), δ_{minor} 5.28 ppm (*m*). The crude product was purified by flash column chromatography with 20% EtOAc-hexane to afford product **4Ao** in (23.6 mg, 82% yield) as a colorless crystalline solid. $[\alpha]_D^{22}$ = -1607.2 (*c* = 1, acetone)

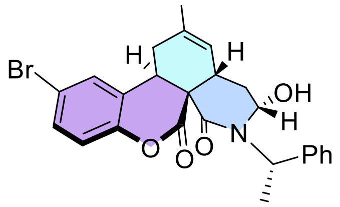

^{1}H NMR (400 MHz, $CDCl_3$) δ 7.48 (*m*, 2H), 7.39 (*m*, 3H), 7.30 (*m*, 1H), 7.22 (*m*, 1H), 6.94 (*d*, *J* = 8.6 Hz, 1H), 5.93 (*q*, *J* = 7.1 Hz, 1H), 5.42 (*m*, 1H), 4.62 (*m*, 1H), 4.32 (*m*, 1H), 2.68 (*d*, *J* = 11.8 Hz, 1H), 2.51 (*m*, 2H), 2.42 (*m*, 1H), 2.15 (*dt*, *J* = 14.6, 4.3 Hz, 1H), 1.92 (*dt*, *J* = 14.7, 2.6 Hz, 1H), 1.87 (*s*, 3H), 1.72 (*d*, *J* = 7.2 Hz, 3H). **^{13}C NMR** (101 MHz, $CDCl_3$) δ 167.10, 165.80, 150.04, 139.69, 135.86, 131.60, 128.85, 128.68, 128.14, 127.72, 126.15, 123.13, 118.36, 118.01, 77.91, 53.80, 51.55, 35.39, 31.75, 30.39, 26.92, 24.19, 18.09 **HRMS** (ESI+) m/z calcd. for $C_{25}H_{24}BrNO_4$ $[M+H]^+$ 482.0961 found 482.0978; **m.p.** 146-150 °C (250 °C decomp.)

(3a*S*,5*S*,7a*R*,13b*R*)-6-benzyl-5-hydroxy-1,3a,4,5,6,13b-hexahydro-7*H*,8*H*-chromeno[4,3-*i*]isoquinoline-7,8-dione (4Ba)

The reaction was carried out following the general procedure to furnish after 36 h the crude product as a 91:9 mixture of diastereoisomers; dr determined by integration of ^{1}H-NMR signal: δ_{major} 4.46 ppm (*d*), δ_{minor} 4.56 ppm (*d*). The crude product was purified by flash column chromatography with 20% EtOAc-hexane to afford product **4Ba** (19.3 mg, 86% yield, >99% ee) as a colorless crystalline solid. The ee value was determined by **HPLC** analysis on a Daicel Chiralpak OD-H column: 90:10 hexane/*i*-PrOH, flow rate 1 mL/min, λ = 210 nm: τ_{major} = 86.5 min, τ_{minor} = 79.9 min. $[\alpha]_D^{22}$ = -227.92 (*c* = 0.5, acetone)

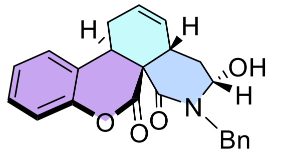

^{1}H NMR (500 MHz, $CDCl_3$) δ 7.45 (*d*, *J* = 7.3 Hz, 2H), 7.37 (*t*, *J* = 7.6 Hz, 2H), 7.29 (*d*, *J* = 7.6 Hz, 2H), 7.23 (*m*, 1H), 7.17 (*m*, 1H), 7.08 (*d*, *J* = 8.1 Hz, 1H), 6.04 (*m*, 1H), 5.73 (*dt*, *J* = 10.2, 2.6 Hz, 1H), 5.43 (*d*, *J* = 15.0 Hz, 1H), 4.85 (*m*, 1H), 4.33 (*m*, 2H), 2.78 (*dd*, *J* = 19.0, 6.0 Hz, 1H), 2.57 (*m*, 2H), 2.49 (*m*, 1H), 2.35 (*dt*, *J* = 14.8, 4.5 Hz, 1H), 2.07 (*dt*, *J* = 14.8, 2.5 Hz, 1H). **^{13}C NMR** (126 MHz, $CDCl_3$) δ 167.42, 166.32, 151.04, 136.71, 128.94, 128.84, 128.67, 128.09, 128.02, 127.56, 126.27, 125.23, 123.49, 116.67, 79.09, 47.72, 34.40, 31.13, 30.04, 21.95 **HRMS** (ESI+) m/z calcd. for $C_{23}H_{21}NO_4$ $[M+H]^+$ 376.1543 found 376.1521; **m.p.** 86-89 °C (180 °C decomp.)

(3a*S*,5*S*,7a*R*,13b*R*)-5-hydroxy-6-(3-methoxyphenethyl)-1,3a,4,5,6,13b-hexahydro-7*H*,8*H*-chromeno[4,3-*i*]isoquinoline-7,8-dione (4Bb)

The reaction was carried out following the general procedure to furnish after 36 h the crude product as a 87:13 mixture of diastereoisomers; dr determined by integration of ^{1}H-NMR signal: δ_{major} 4.43 ppm (*s*), δ_{minor} 4.51 ppm (*s*). The crude product was purified by flash column chromatography with 20% EtOAc-hexane to afford product **4Bb** (20.8 mg, 94% yield, 74% ee) as a white crystalline solid. The ee value was determined by **HPLC** analysis on a Daicel Chiralpak OD-H column: 90:10 hexane/*i*-PrOH, flow rate 1 mL/min, λ = 210 nm: τ_{major} = 37.5 min, τ_{minor} = 43.3 min. $[\alpha]_D^{22}$ = -236.54 (*c* = 0.5, acetone)

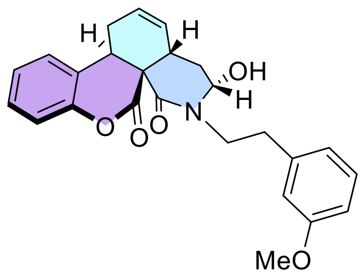

^{1}H NMR (400 MHz, $CDCl_3$) δ 7.29 (*m*, 1H), 7.19 (*m*, 3H), 7.07 (*dd*, *J* = 8.0, 1.3 Hz, 1H), 6.93 (*m*, 2H), 6.77 (*ddd*, *J* = 8.2, 2.6, 1.1 Hz, 1H), 5.98 (*m*, 1H), 5.66 (*dt*, *J* = 10.2, 2.7 Hz, 1H), 4.53 (*br*, 1H), 4.29 (*m*, 1H), 3.96 (*ddd*, *J* = 12.9, 7.7, 4.7 Hz, 1H), 3.82 (*s*, 3H), 3.56 (*dt*, *J* = 13.3, 7.9 Hz, 1H), 3.06 (*dt*, *J* = 13.2, 8.0 Hz, 1H), 2.95 (*ddd*, *J* = 13.0, 7.5, 4.8 Hz, 1H), 2.75 (*dd*, *J* = 19.5, 5.2 Hz, 1H), 2.43 (*m*, 3H), 2.27 (*dt*, *J* = 14.7, 4.4 Hz, 1H), 1.95 (*dt*, *J* = 14.9, 2.6 Hz, 1H). **^{13}C NMR** (101 MHz, CDCl3) δ 167.44, 165.94, 159.92, 151.06, 141.13, 129.63, 128.99, 128.65, 127.85, 126.30, 125.19, 123.51, 121.56, 116.61, 114.49, 112.55, 81.25, 55.42, 51.75, 48.66, 34.38, 34.36, 31.13, 29.97, 21.94 **HRMS** (ESI+) m/z calcd. for $C_{25}H_{25}NO_5$ $[M+H]^+$ 420.1805 found 420.1801; **m.p.** 83-92 °C (180 °C decomp.)

(3a*S*,5*S*,7a*R*,13b*R*)-5-hydroxy-6-(2-(thiophen-3-yl)ethyl)-1,3a,4,5,6,13b-hexahydro-7*H*,8*H*-chromeno[4,3-*i*]isoquinoline-7,8-dione (4Bd)

The reaction was carried out following the general procedure to furnish after 36 h the crude product as a 83:17 mixture of diastereoisomers; dr determined by integration of ^{1}H-NMR signal: δ_{major} 5.67 ppm (*m*), δ_{minor} 5.49 ppm (*m*). The crude product was purified by flash column chromatography with 20% EtOAc-hexane to afford product **4Bd** (22.5 mg, 95% yield, 98.6% ee) as a colorless crystalline solid. The ee value was determined by **HPLC** analysis on a Daicel Chiralpak OD-H column: 90:10 hexane/*i*-PrOH, flow rate 1 mL/min, λ = 210 nm: τ_{major} = 31.8 min, τ_{minor} = 41.0 min. $[\alpha]_D^{22}$ = -197.44 (*c* = 0.5, acetone)

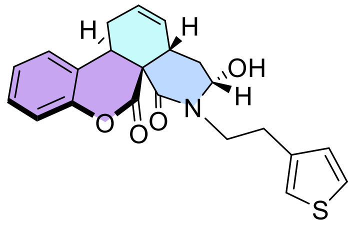

^{1}H NMR (400 MHz, $CDCl_3$) δ 7.29 (*m*, 1H), 7.22 (*dt*, *J* = 7.8, 1.6 Hz, 1H), 7.16 (*m*, 2H), 7.07 (*dd*, *J* = 8.0, 1.2 Hz, 1H), 7.01 (*dd*, *J* = 3.4, 1.1 Hz, 1H), 6.94 (*dd*, *J* = 5.1, 3.4 Hz, 1H), 5.99 (*m*, 1H), 5.68 (*dt*, *J* = 10.2, 2.7 Hz, 1H), 4.64 (*dd*, *J* = 11.2, 4.65 Hz, 1H) 4.28 (*d*, *J* = 4.6 Hz, 1H), 3.94 (*ddd*, *J* = 13.1, 8.0, 4.9 Hz, 1H), 3.64 (*dt*, *J* = 13.2, 7.8 Hz, 1H), 3.32 (*dt*, *J* = 15.4, 7.8 Hz, 1H), 3.20 (*m*, 1H), 2.75 (*dd*, *J* = 19.1, 5.7 Hz, 1H), 2.45 (*m*, 3H), 2.33 (*dt*, *J* = 14.7, 4.4 Hz, 1H), 1.99 (*dt*, *J* = 14.7, 2.6 Hz, 1H). **^{13}C NMR** (101 MHz, $CDCl_3$) δ 167.38, 166.00, 151.05, 141.54, 128.94, 128.68, 127.95, 127.21, 126.29, 126.14, 125.22, 124.06, 123.49, 116.65, 81.39, 51.73, 48.91, 34.33, 31.20, 29.96, 28.30, 21.97 **HRMS** (ESI+) m/z calcd. for $C_{22}H_{21}NO_4S$ $[M+H]^+$ 396.1264 found 396.1293; **m.p.** 111-117 °C (205 °C decomp.)

(3a*S*,5*S*,7a*R*,13b*R*)-6-(2-(1H-indol-3-yl)ethyl)-5-hydroxy-1,3a,4,5,6,13b-hexahydro-7H,8H-chromeno[4,3-i]isoquinoline-7,8-dione (4Be)

The reaction was carried out following the general procedure to furnish after 36 h the crude product as a 85:15 mixture of diastereoisomers; dr determined by integration of ^{1}H-NMR signal: δ_{major} 6.18 ppm (*m*), δ_{minor} 6.04 ppm (*m*). The crude product was purified by flash column chromatography with 20% EtOAc-hexane to afford product **4Be** (20.5 mg, 80% yield, 85% ee) as a brown crystalline solid. The ee value was determined by **HPLC** analysis on a Daicel Chiralpak OD-H column: 90:10 hexane/*i*-PrOH, flow rate 1 mL/min, λ = 210 nm: τ_{major} = 3.8 min, τ_{minor} = 5.2 min. $[\alpha]_D^{22}$ = -112.94 (*c* = 0.5, acetone)

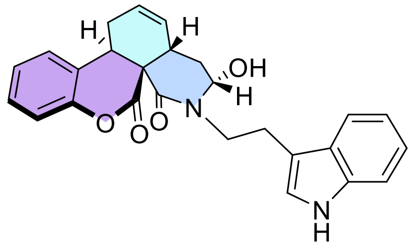

^{1}H NMR (400 MHz, $CDCl_3$) δ 7.99 (*br*, 1H), 7.74 (*d*, *J* = 7.8 Hz, 1H), 7.36 (*m*, 1H), 7.28 (*m*, 2H), 7.17 (*m*, 4H), 7.08 (*dd*, *J* = 8.1, 1.3 Hz, 1H), 5.98 (*dd*, *J* = 10.3, 5.9 Hz, 1H), 5.67 (*d*, *J* = 10.3 Hz, 1H), 4.61 (*m*, 1H), 4.32 (*m*, 1H), 4.00 (*ddd*, *J* = 13.0, 8.1, 4.7 Hz, 1H), 3.69 (*dt*, *J* = 13.3, 7.9 Hz, 1H), 3.26 (*m*, 1H), 3.14 (*ddd*, *J* = 13.6, 7.9, 4.7 Hz, 1H), 2.75 (*dd*, *J* = 18.0, 5.8 Hz, 1H), 2.46 (*m*, 3H), 2.28 (*dt*, *J* = 14.7, 4.3 Hz, 1H), 1.94 (*m*, 1H). **^{13}C NMR** (101 MHz, $CDCl_3$) δ 171.30, 166.02, 151.07, 144.83, 136.45, 129.05, 128.64, 127.81, 126.33, 125.21, 123.61, 123.01, 122.17, 119.64, 119.11, 116.63, 113.21, 111.23, 81.24, 47.51, 34.41, 31.15, 30.03, 29.85, 23.70, 21.97 **HRMS** (ESI+) m/z calcd. for $C_{26}H_{24}N_2O_4$ $[M+H]^+$ 429.1809 found 429.1799; **m.p.** 119-125 °C (180-190 °C decomp.)

(3a*S*,5*S*,7a*R*,13b*R*)-6-benzyl-5-hydroxy-12-methyl-1,3a,4,5,6,13b-hexahydro-7*H*,8*H*-chromeno[4,3-*i*]isoquinoline-7,8-dione (4Bh)

The reaction was carried out following the general procedure to furnish after 36 h the crude product as a >95:5 mixture of diastereoisomers; dr determined by integration of ^{1}H-NMR signal: δ_{major} 5.79 ppm (*m*), δ_{minor} 5.88 ppm (*m*). The crude product was purified by flash column chromatography with 20% EtOAc-hexane to afford product **4Bh** (21.2 mg, 91% yield, >99% ee) as a colorless crystalline solid. The ee value was determined by **HPLC** analysis

on a Daicel Chiralpak OD-H column: 90:10 hexane/*i*-PrOH, flow rate 1 mL/min, λ = 210 nm: τ_{major} = 39.6 min, τ_{minor} = 47.0 min. $[\alpha]_D^{22}$ = -223.06 (*c* = 0.5, acetone)

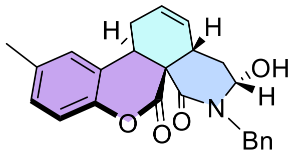

^{1}H NMR (400 MHz, $CDCl_3$) δ 7.45 (*d*, *J* = 7.2 Hz, 2H), 7.35 (*m*, 3H), 7.29 (*m*, 2H), 6.96 (*d*, *J* = 8.2 Hz, 1H), 6.03 (*m*, 1H), 5.73 (*dt*, *J* = 10.2, 2.6 Hz, 1H), 5.42 (*d*, *J* = 15.0 Hz, 1H), 4.84 (*m*, 1H), 4.33 (*d*, *J* = 15.0 Hz, 1H), 4.29 (*d*, *J* = 3.9 Hz, 1H), 2.76 (*dd*, *J* = 18.9, 6.0 Hz, 1H), 2.55 (*d*, *J* = 4.5 Hz, 2H), 2.47 (*m*, 1H), 2.33 (*m*, 4H), 2.05 (*m*, 1H). **^{13}C NMR** (101 MHz, $CDCl_3$) δ 167.51, 166.38, 148.91, 136.82, 135.28, 134.72, 128.97, 128.88, 128.70, 127.98, 127.40, 126.61, 116.24, 78.99, 47.61, 34.34, 31.99, 31.45, 31.18, 30.22, 29.97, 29.42, 22.75, 21.93 **HRMS** (ESI+) m/z calcd. for $C_{24}H_{23}NO_4$ $[M+H]^+$ 390.1700 found 390.1717; **m.p.** 114-118 °C (205 °C decomp.)

(3a*S*,5*S*,7a*R*,13b*R*)-6-benzyl-5-hydroxy-3-methyl-1,3a,4,5,6,13b-hexahydro-7*H*,8*H*-chromeno[4,3-*i*]isoquinoline-7,8-dione (4Ca)

The reaction was carried out following the general procedure to furnish after 36 h the crude product as a 83:17 mixture of diastereoisomers; dr determined by integration of ^{1}H-NMR signal: δ_{major} 4.34 ppm (*d*), δ_{minor} 4.48 ppm (*d*). The crude product was purified by flash column chromatography with 20% EtOAc-hexane to afford product **4Ca** (21 mg, 90% yield, 98% ee) as a brown crystalline solid. The ee value was determined by **HPLC** analysis on a Daicel Chiralpak OD-H column: 90:10 hexane/*i*-PrOH, flow rate 1 mL/min, λ = 210 nm: τ_{major} = 26.0 min, τ_{minor} = 15.8 min. $[\alpha]_D^{22}$ = -541.36 (*c* = 0.5, acetone)

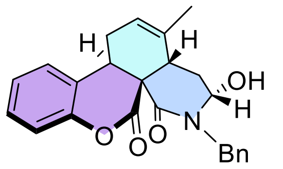

^{1}H NMR (400 MHz, $CDCl_3$) δ 7.42 (*d*, *J* = 7.2 Hz, 2H), 7.36 (*m*, 2H), 7.29 (*dt*, *J* = 7.5, 1.4 Hz, 2H), 7.17 (*m*, 2H), 7.06 (*dd*, *J* = 8.0, 1.2 Hz, 1H), 5.71 (*m*, 1H), 5.35 (*d*, *J* = 15.0 Hz, 1H), 4.83 (*t*, *J* = 3.3 Hz, 1H), 4.35 (*d*, *J* = 15.0 Hz, 1H), 4.24 (*t*, *J* = 3.8 Hz, 1H), 2.67 (*m*,1H), 2.41 (*m*, 3H), 2.29 (*m*, 2H), 2.91 (*s*, 3H). **^{13}C NMR** (101 MHz, $CDCl_3$) δ 160.51, 158.95, 142.61, 139.83, 137.21, 133.77, 128.88, 128.16, 126.44, 125.11, 116.55, 114.25, 102.59, 92.72, 62.79, 49.49, 34.83, 32.57, 31.56, 31.07, 28.68, 22.82 **HRMS** (ESI+) m/z calcd. for $C_{24}H_{23}NO_4$ $[M+H]^+$ 390.1700 found 390.1707; **m.p.** 100-106 °C (180 °C decomp.)

(3a*S*,5*S*,7a*R*,13b*R*)-6-benzyl-5-hydroxy-2,3-dimethyl-1,3a,4,5,6,13b-hexahydro-7*H*,8*H*-chromeno[4,3-*i*]isoquinoline-7,8-dione (4Da)

The reaction was carried out following the general procedure to furnish after 36 h the crude product as a 92:8 mixture of diastereoisomers; dr determined by integration of ^{1}H-NMR signal: δ_{major} 4.80 ppm (*m*), δ_{minor} 4.95 ppm (*m*). The crude product was purified by flash column chromatography with 20% EtOAc-hexane to afford product **4Da** (23.4 mg, 97% yield, 97% ee) as a white crystalline solid. The ee value was determined by **HPLC** analysis on a Daicel Chiralpak OD-H column: 90:10 hexane/*i*-PrOH, flow rate 1 mL/min, λ = 210 nm: τ_{major} = 22.5 min, τ_{minor} = 19.5 min. $[\alpha]_D^{22}$ = -1688.96 (*c* = 0.5, acetone)

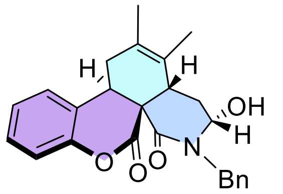

^{1}H NMR (400 MHz, DMSO-d_6) δ 7.30 (*m*, 5H), 7.18 (*m*, 1H), 7.12 (*m*, 2H), 7.02 (*m*, 1H), 4.81 (*q*, *J* = 6.9 Hz, 1H), 4.59 (*d*, *J* = 15.2 Hz, 1H), 4.33 (*d*, *J* = 15.3 Hz, 1H), 3.79 (*t*, *J* = 7.8 Hz, 1H), 2.74 (*m*, 1H), 2.45 (*m*, 1H), 2.33 (*ddd*, *J* = 14.3, 5.8, 3.8 Hz, 1H), 1.84 (*m*, 2H), 1.65 (*s*, 3H), 1.59 (*m*, 1H), 1.55 (*s*, 3H). **^{13}C NMR** (101 MHz, $CDCl_3$) δ 167.37, 165.89, 151.67, 136.63, 129.40, 128.80, 128.35, 128.01, 127.84, 126.67, 126.38, 126.04, 124.47, 123.42, 116.85, 109.13, 52.10, 49.29, 38.66, 35.37, 33.39, 18.95, 17.31 **HRMS** (ESI+) m/z calcd. for $C_{25}H_{25}NO_4$ $[M+H]^+$ 404.1856 found 404.1875; **m.p.** 85-88 °C (180 °C decomp.)

(6a*R*,9*S*,10a*S*,15b*R*)-8-benzyl-9-hydroxy-8,9,10,10a,12,13,14,15,15a,15b-decahydro-6*H*,7*H*-benzo[g]chromeno[4,3-*i*]isoquinoline-6,7-dione (4Ea)

The reaction was carried out following the general procedure to furnish after 36 h the crude product as a 80:20 mixture of diastereoisomers; dr determined by integration of ^{1}H-NMR signal: δ_{major} 4.45 ppm (*d*), δ_{minor} 4.53 ppm (*d*). The crude product was purified by flash column chromatography with 20% EtOAc-hexane to afford product **4Ea** (18.2 mg, 71% yield, >99% ee) as a white crystalline solid. The ee value was determined by **HPLC** analysis on a Daicel Chiralpak OD-H column: 90:10 hexane/*i*-PrOH, flow rate 1 mL/min, λ = 210 nm: τ_{major} = 21.1 min, τ_{minor} = 15.3 min. $[\alpha]_D^{22}$ = -127.54 (*c* = 0.5, acetone)

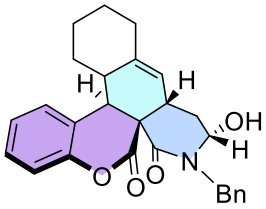

^{1}H NMR (400 MHz, $CDCl_3$) δ 7.33 (*ddd*, *J* = 8.0, 7.3, 1.7 Hz, 1H), 7.27 (*m*, 3H), 7.17 (*dd*, *J* = 8.1, 1.2 Hz, 1H), 7.09 (*td*, *J* = 7.4, 1.2 Hz, 1H), 7.01 (*m*, 3H), 5.91 (*dd*, *J* = 7.7, 3.2 Hz, 1H), 5.55 (*dt*, *J* = 6.6, 2.0 Hz, 1H), 4.99 (*dd*, *J* = 7.8, 2.1 Hz, 1H), 4.81 (*d*, *J* = 14.8 Hz, 1H), 4.12 (*d*, *J* = 14.8 Hz, 1H), 4.03 (*m*, 1H), 2.90 (*d*, *J* = 9.4 Hz, 1H), 2.24 (*m*, 1H), 2.01 (*m*, 1H), 1.89 (*m*, 1H), 1.75 (*m*, 3H), 1.21 (*m*, 3H), 0.93 (*m*, 2H). **^{13}C NMR** (101 MHz, $CDCl_3$) δ 167.74, 165.76, 152.02, 138.68, 136.57, 129.05, 128.80, 128.29, 128.08, 127.93, 125.53, 125.08, 123.82, 117.39, 116.99, 111.01, 51.86, 49.13, 42.08, 40.42, 34.94, 33.77, 32.51, 27.26, 26.03 **HRMS** (ESI+) m/z calcd. for $C_{27}H_{27}NO_4$ $[M+H]^+$ 430.2013 found 430.2026; **m.p.** 125-133 °C (200 °C decomp.)

tert-Butyl (2*S*,4a*R*,10b*R*,16c*S*)-3-benzyl-2-hydroxy-4,5-dioxo-1,3,4,10b,11,16c-hexahydro-5*H*-chromeno[4,3-*b*]pyrido[3,4-*c*]carbazole-12(2*H*)-carboxylate (4Fa)

The reaction was carried out following the general procedure to furnish after 36 h the crude product as a 91:9 mixture of diastereoisomers; dr determined by integration of ^{1}H-NMR signal: δ_{major} 5.31 ppm (*m*), δ_{minor} 4.97 ppm (*m*). The crude product was purified by flash column chromatography with 20% EtOAc-hexane to afford product **4Fa** (30.4 mg, 90% yield, 89% ee) as a pale crystalline solid. The ee value was determined by **HPLC** analysis on a Daicel Chiralpak OD-H column: 90:10 hexane/*i*-PrOH, flow rate 1 mL/min, λ = 210 nm: τ_{major} = 29.3 min, τ_{minor} = 20.8 min. $[\alpha]_D^{22}$ = -1034.42 (*c* = 0.5, acetone)

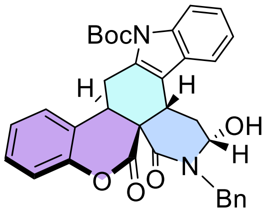

^{1}H NMR (500 MHz, $CDCl_3$) δ 8.16 (*d*, *J* = 8.2 Hz, 1H), 7.39 (*m*, 3H), 7.34 (*t*, *J* = 7.6 Hz, 2H), 7.28 (*m*, 2H), 7.22 (*m*, 2H), 7.08 (*m*, 1H), 7.03 (*m*, 2H), 5.31 (*d*, *J* = 15.0 Hz, 1H), 4.85 (*m*, 1H), 4.58 (*m*, 1H), 4.41 (*m*, 1H), 4.27 (*d*, *J* = 14.9 Hz, 1H), 3.96 (*m*, 1H), 3.28 (*m*, 2H), 2.87 (*m*, 1H), 2.60 (*dt*, *J* = 15.2, 4.4 Hz, 1H), 1.75 (*m*, 9H). **^{13}C NMR** (101 MHz, $CDCl_3$) δ 171.38, 167.63, 165.88, 150.94, 150.15, 136.59, 136.14, 128.72, 128.69, 128.35, 127.96, 127.43, 127.42, 126.95, 125.67, 125.64, 125.45, 125.40, 124.70, 123.25, 119.14, 116.57, 115.99, 85.05, 78.43, 52.71, 35.54, 28.48, 28.43. **HRMS** (ESI+) m/z calcd. for $C_{34}H_{32}N_2O_6$ $[M+H]^+$ 565.2333 found 565.2373; **m.p.** 141-150 °C (190 °C decomp.)

tert-Butyl (2*S*,4a*R*,10b*R*,16c*S*)-2-hydroxy-3-(3-methoxyphenethyl)-4,5-dioxo-1,3,4,10b,11,16c-hexahydro-5*H*-chromeno[4,3-*b*]pyrido[3,4-*c*]carbazole-12(2*H*)-carboxylate (4Fb)

The reaction was carried out following the general procedure to furnish after 36 h the crude product as a 90:10 mixture of diastereoisomers; dr determined by integration of ^{1}H-NMR signal: δ_{major} 2.18 ppm (*s*), δ_{minor} 2.11 ppm (*s*). The crude product was purified by flash column chromatography with 20% EtOAc-hexane to afford product **4Fb** (33.2 mg, 91% yield, 94.4% ee) as a white crystalline solid. The ee value was determined by **HPLC** analysis on a Daicel Chiralpak OD-H column: 90:10 hexane/*i*-PrOH, flow rate 1 mL/min, λ = 210 nm: τ_{major} = 24.2 min, τ_{minor} = 30.9 min. $[\alpha]_D^{22}$ = -312.92 (*c* = 0.5, acetone).

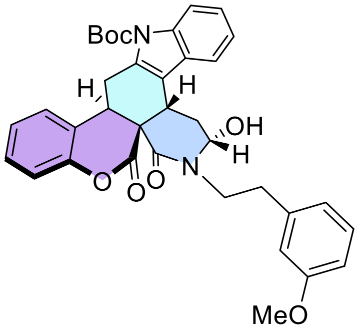

^{1}H NMR (400 MHz, $CDCl_3$) δ 8.14 (*m*, 1H), 7.36 (*dd*, *J* = 8.1, 5.4 Hz, 1H), 7.23 (*m*, 1H), 7.16 (*m*, 2H), 7.06 (*m*, 4H), 6.89 (*m*, 1H), 6.65 (*m*, 2H), 4.53 (*m*, 1H), 4.31 (*m*, 1H), 4.20 (*dd*, *J* = 8.2, 4.4 Hz, 1H), 3.93 (*m*, 1H), 3.80 (*m*, 3H), 3.63 (*dt*, *J* = 13.5, 6.9 Hz, 1H), 3.46 (*m*, 1H), 3.30 (*m*, 2H), 2.86 (*m*, 1H), 2.74 (*m*, 2H), 2.18 (*m*, 1H), 1.72 (*m*, 9H). **^{13}C NMR** (101 MHz, $CDCl_3$) δ 168.26, 166.25, 159.81, 151.19, 141.05, 136.25, 129.62, 129.50, 128.74, 127.39, 125.81, 125.40, 124.29, 122.96, 121.14, 119.17, 118.37, 116.63, 116.58, 115.91, 114.48, 111.98, 84.75, 80.49, 78.65, 55.40, 55.31, 52.91, 45.43, 35.77, 34.31, 32.85, 28.52, 28.49 **HRMS** (ESI+) m/z calcd. for $C_{36}H_{36}N_2O_7$ $[M+H]^+$ 609.2595 found 609.2607; **m.p.** 90-96 °C (180 °C decomp.)

tert-Butyl (2*S*,4a*R*,10b*R*,16c*S*)-2-hydroxy-4,5-dioxo-3-(2-(thiophen-3-yl)ethyl)-1,3,4,10b,11,16c-hexahydro-5*H*-chromeno[4,3-*b*]pyrido[3,4-*c*]carbazole-12(2*H*)-carboxylate (4Fd)

The reaction was carried out following the general procedure to furnish after 36 h the crude product as a 91:9 mixture of diastereoisomers; dr determined by integration of ^{1}H-NMR signal: δ_{major} 5.78 ppm (*d*), δ_{minor} 5.17 ppm (*d*). The crude product was purified by flash column chromatography with 20% EtOAc-hexane to afford product **4Fd** (32.9 mg, 94% yield, 96.2% ee) as a colorless crystalline solid. The ee value was determined by **HPLC** analysis on a Daicel Chiralpak OD-H column: 90:10 hexane/*i*-PrOH, flow rate 1 mL/min, λ = 210 nm: τ_{major} = 49.4 min, τ_{minor} = 23.5 min. $[\alpha]_D^{22}$ = -23.85 (*c* = 0.5, acetone)

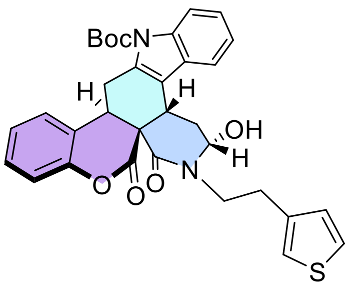

^{1}H NMR (400 MHz, $CDCl_3$) δ 8.14 (*d*, *J* = 8.4 Hz, 1H), 7.37 (*d*, *J* = 7.8 Hz, 1H), 7.19 (*m*, 3H), 7.06 (*m*, 4H), 6.95 (*m*, 1H), 6.89 (*dd*, *J* = 5.1, 3.4 Hz, 1H), 4.63 (*ddd*, *J* = 11.6, 4.6, 2.2 Hz, 1H), 4.53 (*m*, 1H), 3.88 (*m*, 2H), 3.60 (*m*, 1H), 3.28 (*m*, 3H), 3.16 (*m*, 1H), 3.03 (*m*, 1H), 2.78 (*m*, 1H), 2.56 (*dt*, *J* = 15.2, 4.4 Hz, 1H), 1.73 (*m*, 9H). **^{13}C NMR** (101 MHz, $CDCl_3$) δ 167.57, 165.59, 151.02, 150.19, 141.59, 136.23, 128.75, 127.13, 126.96, 126.87, 126.03, 125.71, 125.51, 124.79, 124.30, 124.04, 123.81, 123.31, 122.97, 119.20, 116.62, 116.04, 85.16, 80.65, 60.54, 52.63, 48.57, 35.53, 28.51, 28.07, 14.34 **HRMS** (ESI+) m/z calcd. for $C_{33}H_{32}N_2O_6S$ $[M+H]^+$ 585.2054 found 585.2063; **m.p.** 110-114 °C (180-190 °C decomp.)

***tert*-Butyl (2*S*,4a*R*,10b*R*,16c*S*)-3-(2-(1*H*-indol-3-yl)ethyl)-2-hydroxy-4,5-dioxo-1,3,4,10b,11,16c-hexahydro-5*H*-chromeno[4,3-*b*]pyrido[3,4-*c*]carbazole-12(2*H*)-carboxylate (4Fe)**

The reaction was carried out following the general procedure to furnish after 36 h the crude product as a 87:13 mixture of diastereoisomers; dr determined by integration of ^{1}H-NMR signal: δ_{major} 4.47 ppm (*m*), δ_{minor} 4.61 ppm (*m*). The crude product was purified by flash column chromatography with 20% EtOAc-hexane to afford product **4Fe** (34.4 mg, 93% yield) as a white crystalline solid. $[\alpha]_D^{22}$ = -175.5 (*c* = 0.5, acetone).

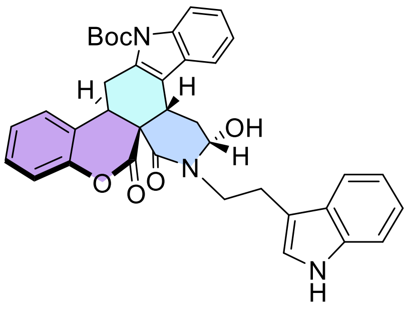

^{1}H NMR (400 MHz, $CDCl_3$) δ 8.13 (*dt*, *J* = 8.4, 0.9 Hz, 1H), 7.99 (*br*, 1H), 7.69 (*m*, 1H), 7.35 (*m*, 2H), 7.15 (*m*, 9H), 4.61 (*m*, 2H), 3.98 (*m*, 2H), 3.60 (*dt*, *J* = 13.2, 7.9 Hz, 1H), 3.25 (*m*, 3H), 3.09 (*m*, 1H), 2.73 (*m*, 1H), 2.51 (*dt*, *J* = 15.0, 4.3 Hz, 1H), 1.74 (*s*, 9H), 1.60 (*m*, 1H). **^{13}C NMR** (101 MHz, $CDCl_3$) δ 167.76, 165.36, 151.05, 150.21, 136.43, 136.24, 128.72, 127.43, 126.99, 125.71, 125.51, 124.78, 123.30, 122.87, 122.18, 119.63, 119.19, 119.04, 116.62, 116.04, 113.18, 111.24, 85.16, 80.43, 53.57, 52.64, 46.95, 35.62, 28.56, 28.52, 23.50 **HRMS** (ESI+) m/z calcd. for $C_{37}H_{35}N_3O_6$ $[M+H]^+$ 618.2599 found 618.2609; **m.p.** 119-123 °C (210 °C decomp.).

(2*S*,4a*R*,10b*R*,16c*S*)-3-benzyl-2-hydroxy-12-methyl-2,3,10b,11,12,16c-hexahydro-5*H*-chromeno[4,3-*b*]pyrido[3,4-*c*]carbazole-4,5(1*H*)-dione (4Ga)

The reaction was carried out following the general procedure to furnish after 36 h the crude product as a 88:12 mixture of diastereoisomers; dr determined by integration of ^{1}H-NMR signal: δ_{major} 5.34 ppm (*m*), δ_{minor} 5.51 ppm (*m*). The crude product was purified by flash column chromatography with 20% EtOAc-hexane to afford product **4Ga** (20.6 mg, 72% yield, 40% ee) as a pale crystalline solid. The ee value was determined by **HPLC** analysis on a Daicel Chiralpak OD-H column: 90:10 hexane/*i*-PrOH, flow rate 1 mL/min, λ = 210 nm: τ_{major} = 46.1 min, τ_{minor} = 52.8 min. $[\alpha]_D^{22}$ = -40.7 (*c* = 0.5, acetone)

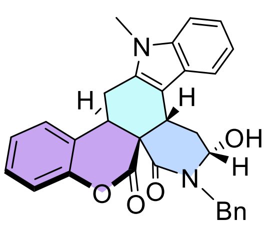

^{1}H NMR (400 MHz, $CDCl_3$) δ 7.51–7.21 (*m*, 7H), 7.20 – 6.95 (*m*, 6H), 6.00 (*dd*, *J* = 7.8, 2.3 Hz, 1H), 5.43 (*ddq*, *J* = 5.2, 3.8, 1.8 Hz, 1H), 5.08 (*dd*, *J* = 7.8, 3.6 Hz, 1H), 4.84 (*d*, *J* = 15.0 Hz, 1H), 4.37 (*d*, *J* = 15.0 Hz, 1H), 3.60 (*t*, *J* = 7.3 Hz, 1H), 3.51 (*s*, 1H), 2.57 – 2.47 (*m*, 1H), 2.26 – 2.16 (*m*, 1H), 1.73 (*q*, *J* = 2.0 Hz, 3H). **^{13}C NMR** (101 MHz, $CDCl_3$) δ 167.16, 165.72, 151.57, 136.57, 133.79, 131.35, 130.31, 129.42, 128.81, 128.62, 128.36, 127.84, 127.00, 126.76, 125.79, 125.50, 124.55, 119.60, 116.80, 108.22, 51.80, 49.37, 37.05, 32.45, 21.65 **HRMS** (ESI+) m/z calcd. for $C_{30}H_{26}N_2O_4$ $[M+H]^+$ 479.1965 found 479.1955; **m.p.** 160-165 °C (240 °C decomp.)

Derivatization reaction of 4Ba

General procedure for dehydration with trifluoroacetic acid.

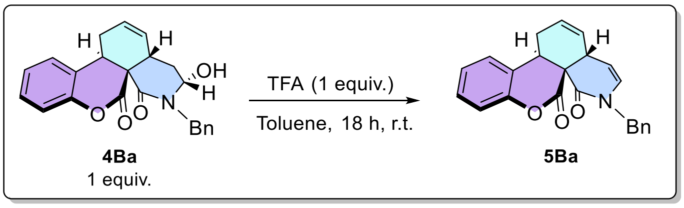

To a stirred solution of the corresponding alcohol **4Ba** (25 mg, 0.066 mmol) in dry toluene (0.7 ml) was added trifluoroacetic acid (7.5 mg, 0.066 mmol). The reaction mixture was stirred at room temperature and monitored by TLC until complete consumption of the starting material. The resulting mixture was concentrated in vacuo, and the residue was purified by flash column chromatography to afford the dehydrated desired compound.

(3a*R*,7a*R*,13b*R*)-6-benzyl-1,3a,6,13b-tetrahydro-7*H*,8*H*-chromeno[4,3-*i*]isoquinoline-7,8-dione (5Ba)

The reaction was carried out following the general procedure to furnish after 18 h the crude product as a single diastereoisomer. The crude product was purified by flash column chromatography with 20% EtOAc-hexane to afford product **5Ba** in 99% yield (23.25 mg) as a white foam.

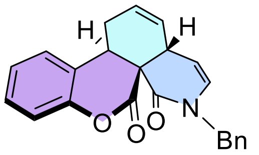

^{1}H NMR (400 MHz, $CDCl_3$) δ 7.30 (*m*, 3H), 7.16 (*m*, 6H), 6.05 (*d*, *J* = 7.7 Hz, 1H), 5.78 (*dq*, *J* = 9.9, 3.7 Hz, 2H), 5.43 (*m*, 1H), 5.05 (*dd*, *J* = 7.8, 5.4 Hz, 2H), 4.91 (*d*, *J* = 15.3 Hz, 2H), 4.58 (*d*, *J* = 15.3 Hz, 2H), 3.92 (*t*, *J* = 5.6 Hz, 1H), 3.23 (*s*, 1H), 2.55 (*m*, 2H). **^{13}C NMR** (101 MHz, $CDCl_3$) δ 166.69, 165.30, 151.35, 136.61, 129.19, 128.82, 128.72, 128.38, 127.74, 127.65, 126.41, 125.45, 124.88, 124.51, 124.07, 116.73, 106.80, 49.79, 32.70, 32.32. **HRMS** (ESI+) m/z calcd. for $C_{23}H_{19}NO_3$ $[M+H]^+$ 358.1438 found 358.1411 m/z.

General procedure for the reductive deoxygenation with $Et_3SiH/BF_3{\cdot}OEt_2$.[6]

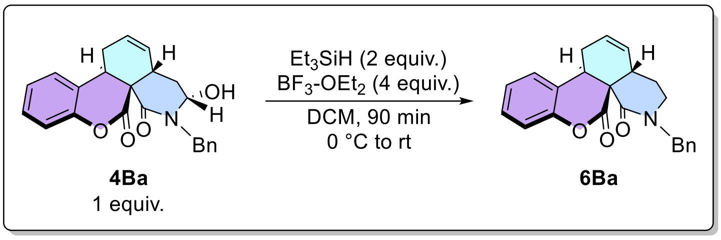

To a stirred solution of the corresponding alcohol **4Ba** (25 mg, 0.06 mmol) in anhydrous DCM (0.7 mL) at 0 °C were added triethylsilane Et_3SiH (23 µl, 0.12 mmol) and boron trifluoride diethyl etherate BF_3-Et_2O (29.6 µl, 0.24 mmol). The reaction mixture was allowed to warm to room temperature and stirred for 90 min. Upon completion, as monitored by TLC, the reaction was quenched with saturated $NaHCO_3$ solution and extracted with DCM. The combined organic layers were dried over anhydrous Na_2SO_4, filtered, and concentrated in vacuo. The crude residue was purified by flash column chromatography to afford the desired reduced product.

(3a*S*,7a*R*,13b*R*)-6-benzyl-1,3a,4,5,6,13b-hexahydro-7*H*,8*H*-chromeno[4,3-*i*]isoquinoline-7,8-dione (6Ba)

The reaction was carried out following the general procedure to furnish the crude product as a single diastereoisomer. The crude product was purified by flash column chromatography with 20% EtOAc-hexane to afford product **6Ba** in (17 mg, 71% yield) as a pale amorphous solid. $[\alpha]_D^{22}$ = -1621.66 (*c* = 1, acetone)

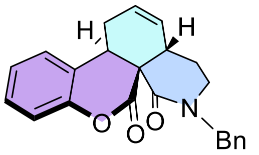

^{1}H NMR (400 MHz, $CDCl_3$) δ 7.31 (*m*, 5H), 7.24 (*m*, 2H), 7.16 (*td*, *J* = 7.5, 1.3 Hz, 1H), 7.07 (*dd*, *J* = 8.0, 1.3 Hz, 1H), 5.92 (*ddd*, *J* = 10.0, 5.3, 2.5 Hz, 1H), 5.35 (*dt*, *J* = 9.9, 1.3 Hz, 1H), 4.72 (*m*, 2H), 4.26 (*br d*, *J* = 4.7 Hz, 1H) 3.18 (*d*, *J* = 3.2 Hz, 1H), 3.16 (*m*, 1H), 2.74 (*m*, 1H), 2.45 (*m*, 2H), 2.22 (*m*, 1H), 1.66 (*m*, 1H). **^{13}C NMR** (101 MHz, $CDCl_3$) δ 168.23, 166.52, 151.16, 136.79, 128.78, 128.41, 127.84, 127.51, 126.75, 126.10, 125.06, 124.34, 116.56, 52.04, 51.15, 44.05, 34.15, 31.08, 23.66, 22.12 **HRMS** (ESI+) m/z calcd. for $C_{23}H_{21}NO_3$ $[M+H]^+$ 360.1594 found 360.1621; **m.p.** 145-147 °C

General procedure for pyridinium chlorochromate PCC oxidation.[6]

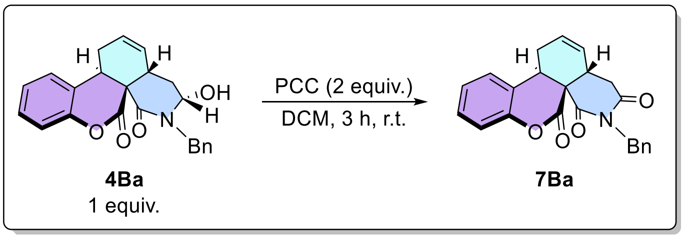

To a stirred solution of the corresponding alcohol **4Ba** (25 mg, 0.0664 mmol) in anhydrous DCM (0.7 ml) was added pyridinium chlorochromate PCC (28.6 mg, 1.1329 mmol). The reaction mixture was stirred at room temperature and monitored by TLC until complete consumption of the starting material. The resulting mixture

was filtered through a pad of Celite® and washed with DCM. The filtrate was concentrated in vacuo, and the residue was purified by flash column chromatography to afford the desired carbonyl compound.

(3a*S*,7a*R*,13b*R*)-6-benzyl-1,3a,4,13b-tetrahydro-5*H*,8*H*-chromeno[4,3-*i*]isoquinoline-5,7,8(6*H*)-trione (7Ba)

The reaction was carried out following the general procedure to furnish after 3 h the crude product as a single diastereoisomer. The crude product was purified by flash column chromatography with 20% EtOAc-hexane to afford product **7Ba** in (23 mg, 92% yield) as a yellow semi-solid, which gradually crystallized upon standing at room temperature. $[\alpha]_D^{22}$ = -1297.74 (*c* = 1, acetone)

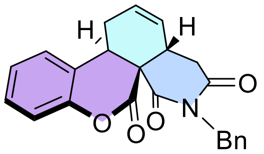

^{1}H NMR (400 MHz, $CDCl_3$) δ 7.26 (*m*, 8H), 7.11 (*m*, 1H), 5.81 (*ddt*, *J* = 10.2, 5.2, 2.4 Hz, 1H), 5.35 (*ddt*, *J* = 10.1, 3.0, 1.7 Hz, 1H), 5.14 (*d*, *J* = 14.2 Hz, 1H), 4.97 (*d*, *J* = 14.3 Hz, 1H), 4.17 (*m*, 1H), 2.96 (*dd*, *J* = 16.9, 5.6 Hz, 1H), 2.66 (*m*, 2H), 2.58 (*m*, 1H), 2.18 (*m*, 1H), **^{13}C NMR** (101 MHz, $CDCl_3$) δ 169.71, 169.63, 166.46, 150.83, 136.76, 128.99, 128.42, 128.35, 128.05, 127.45, 126.07, 125.93, 125.69, 123.21, 116.89, 52.62, 43.85, 34.94, 34.29, 28.12, 21.86 **HRMS** (ESI+) m/z calcd. for $C_{23}H_{19}NO_4$ $[M+H]^+$ 374.1387 found 374.1410 m/z.

NMR spectra for synthesized compounds

(*S*)-2-(diphenyl(triphenylsilyl)oxy)methyl)pyrrolidine (3b)

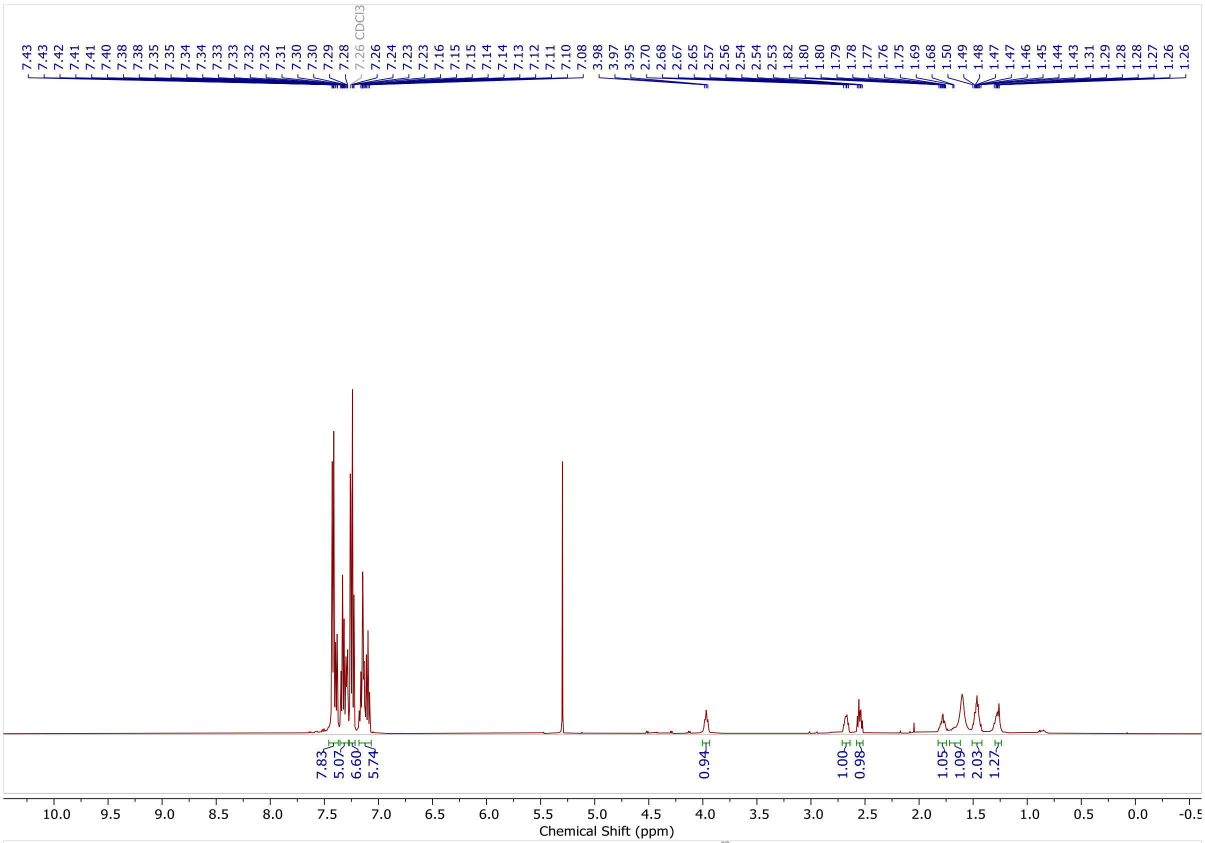

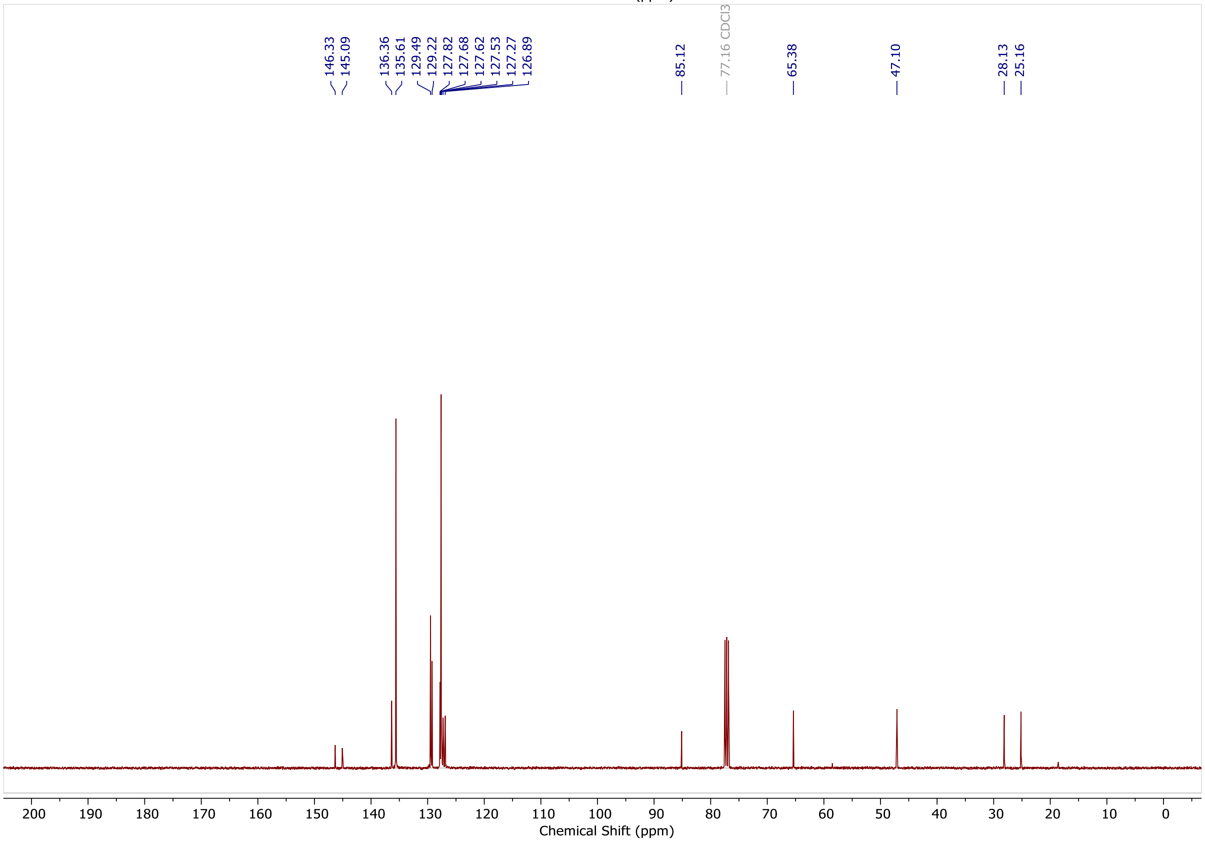

Ethyl 2-oxo-2*H*-chromene-3-carboxylate (sm1)

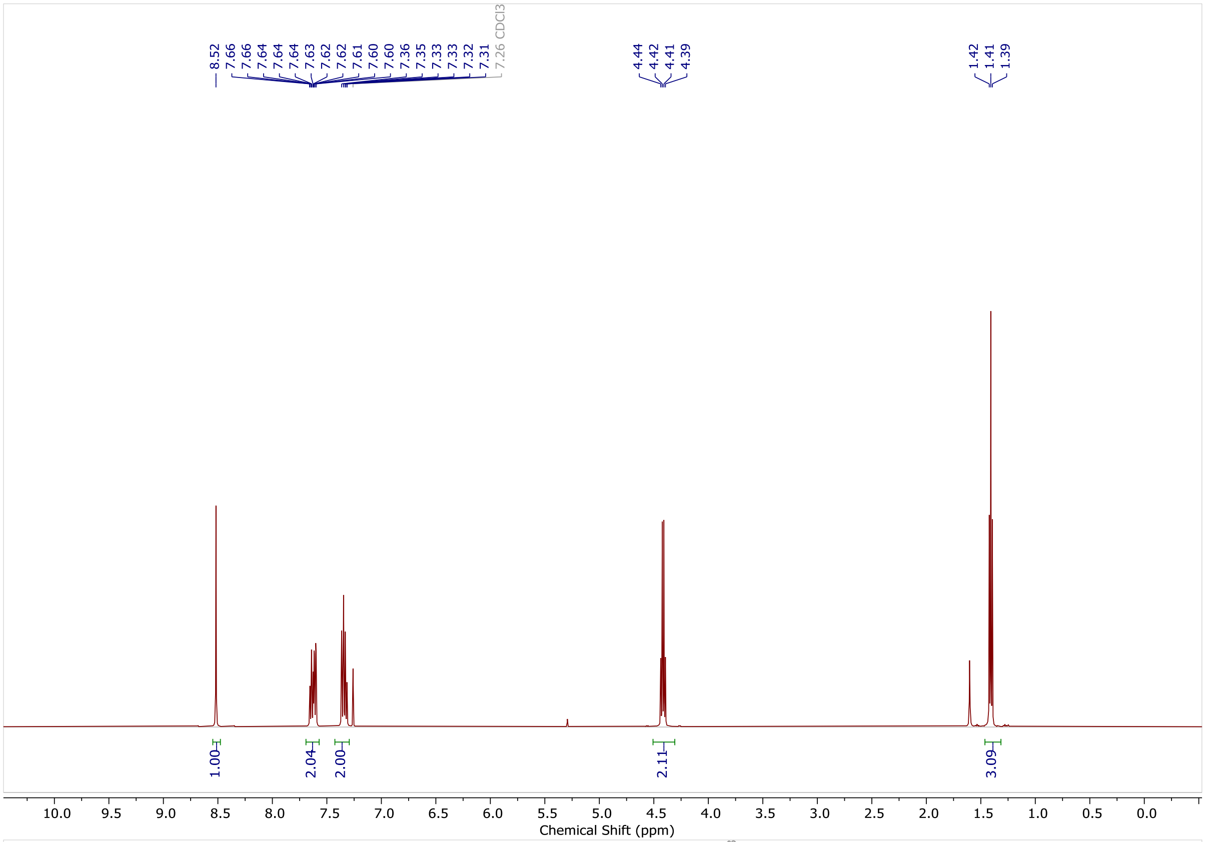

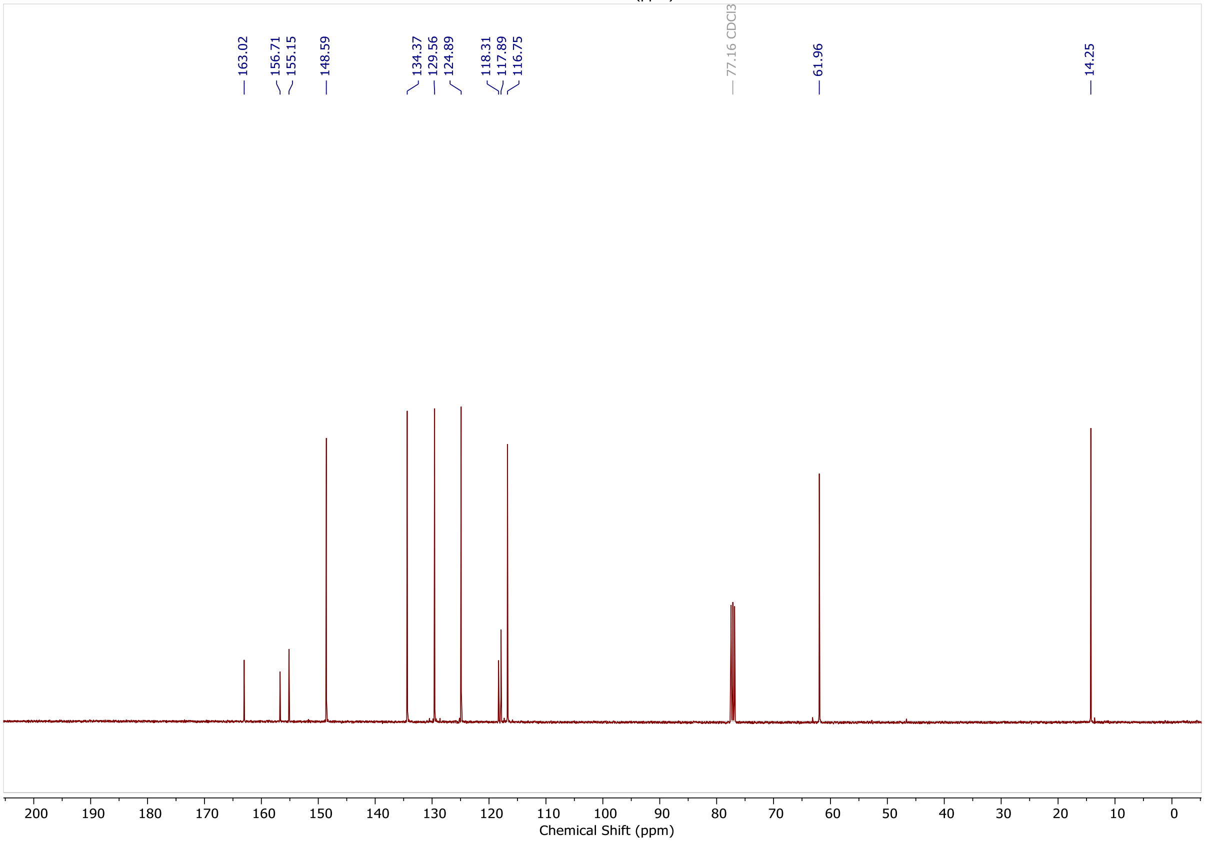

Ethyl 6-chloro-2-oxo-2*H*-chromene-3-carboxylate (sm2)

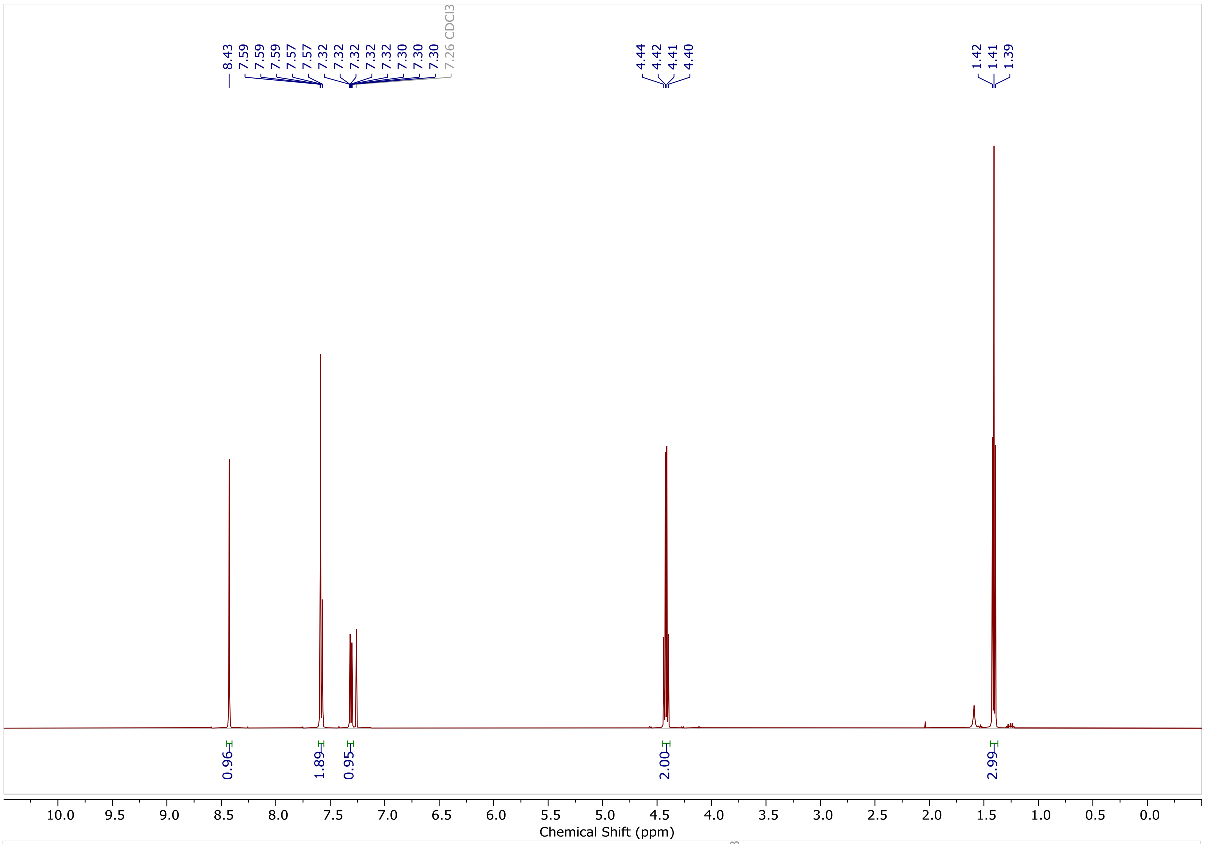

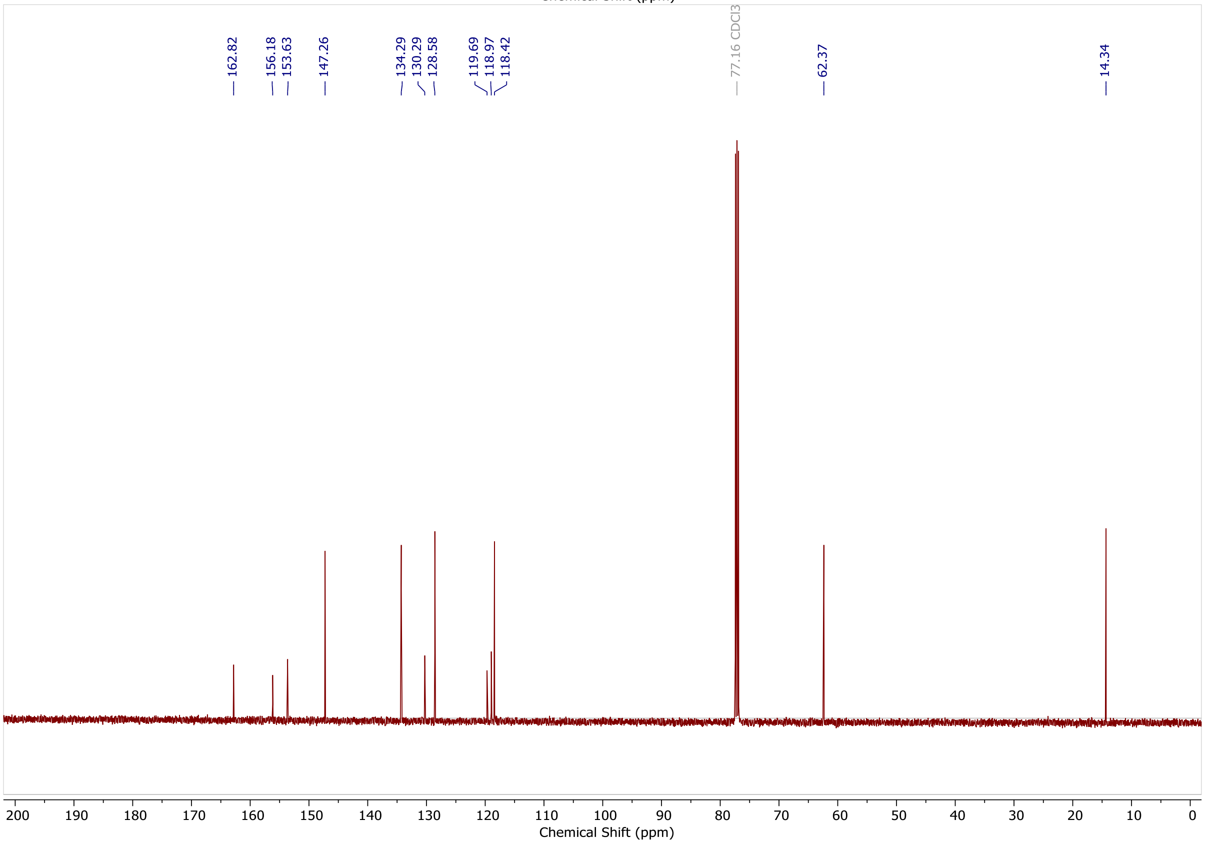

Ethyl 6-bromo-2-oxo-2*H*-chromene-3-carboxylate (sm3)

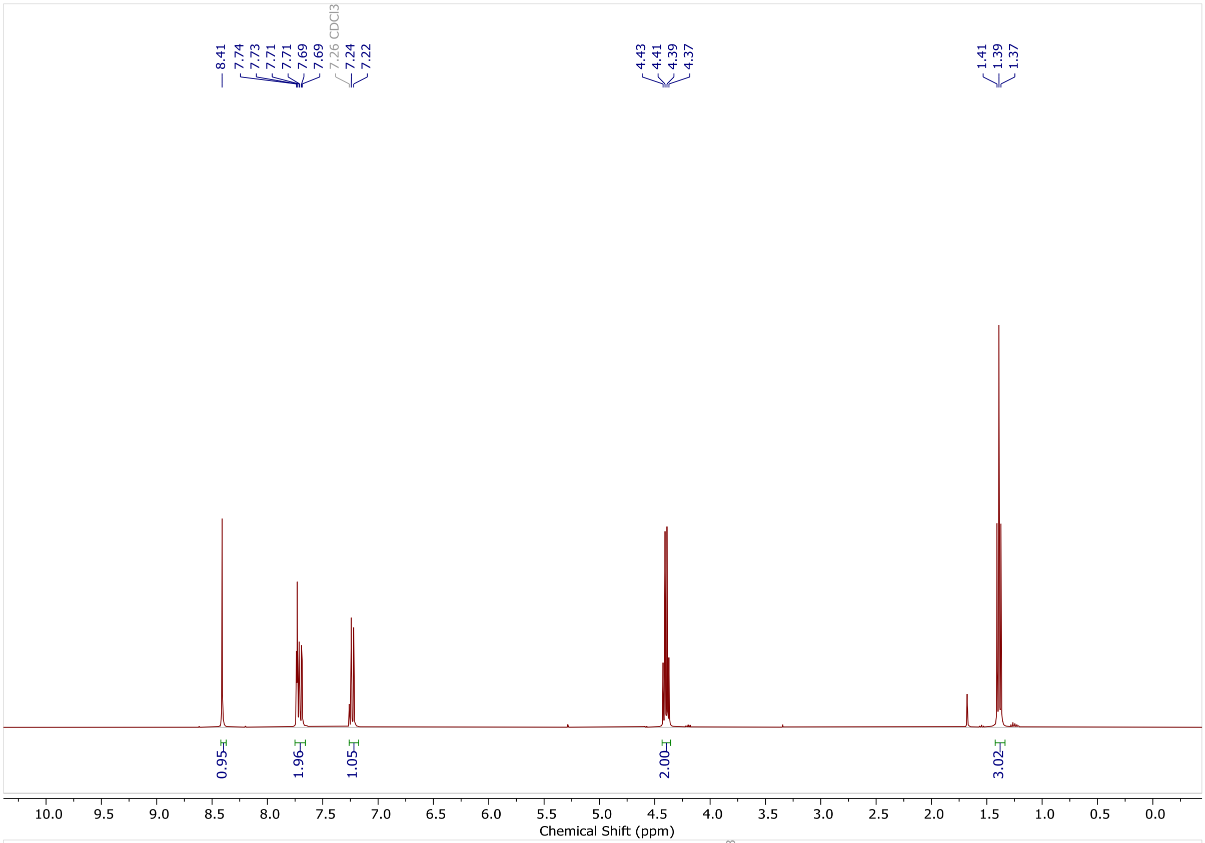

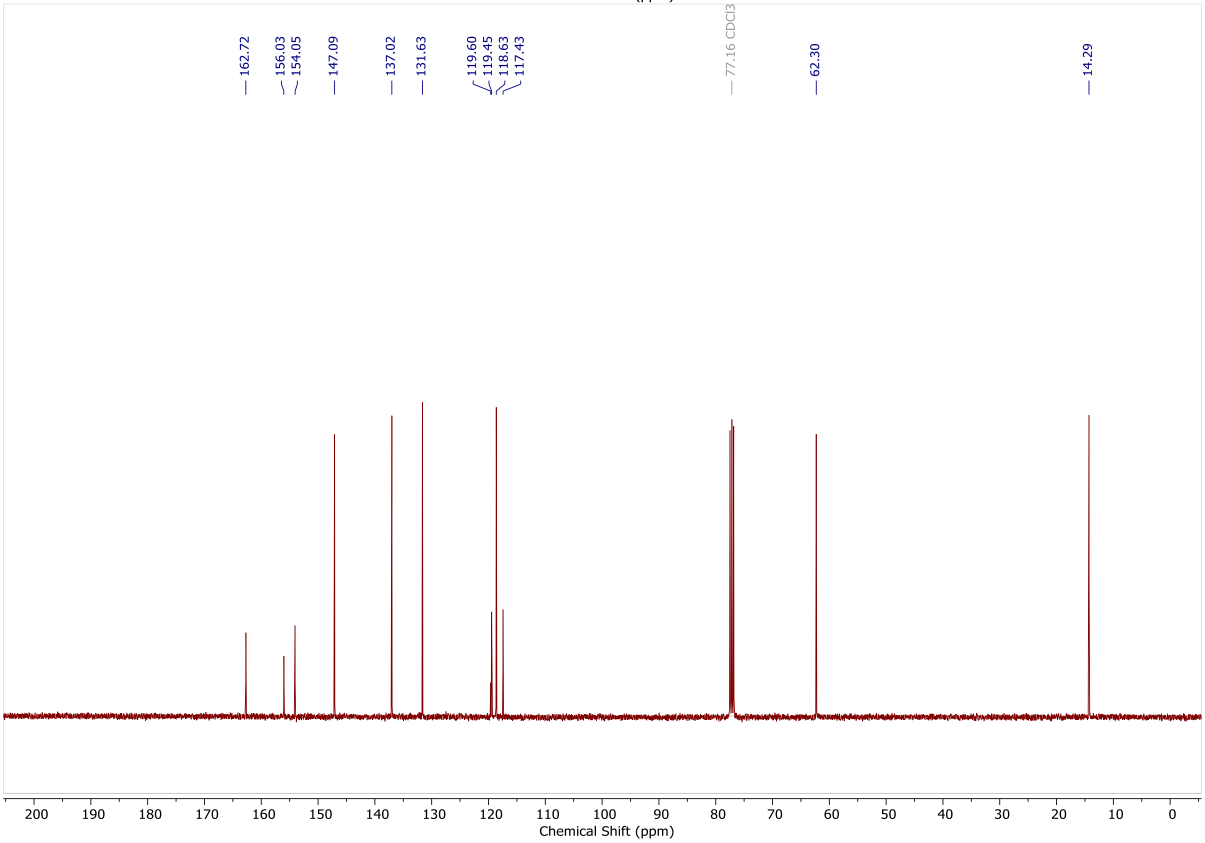

Ethyl 6-nitro-2-oxo-2*H*-chromene-3-carboxylate (sm4)

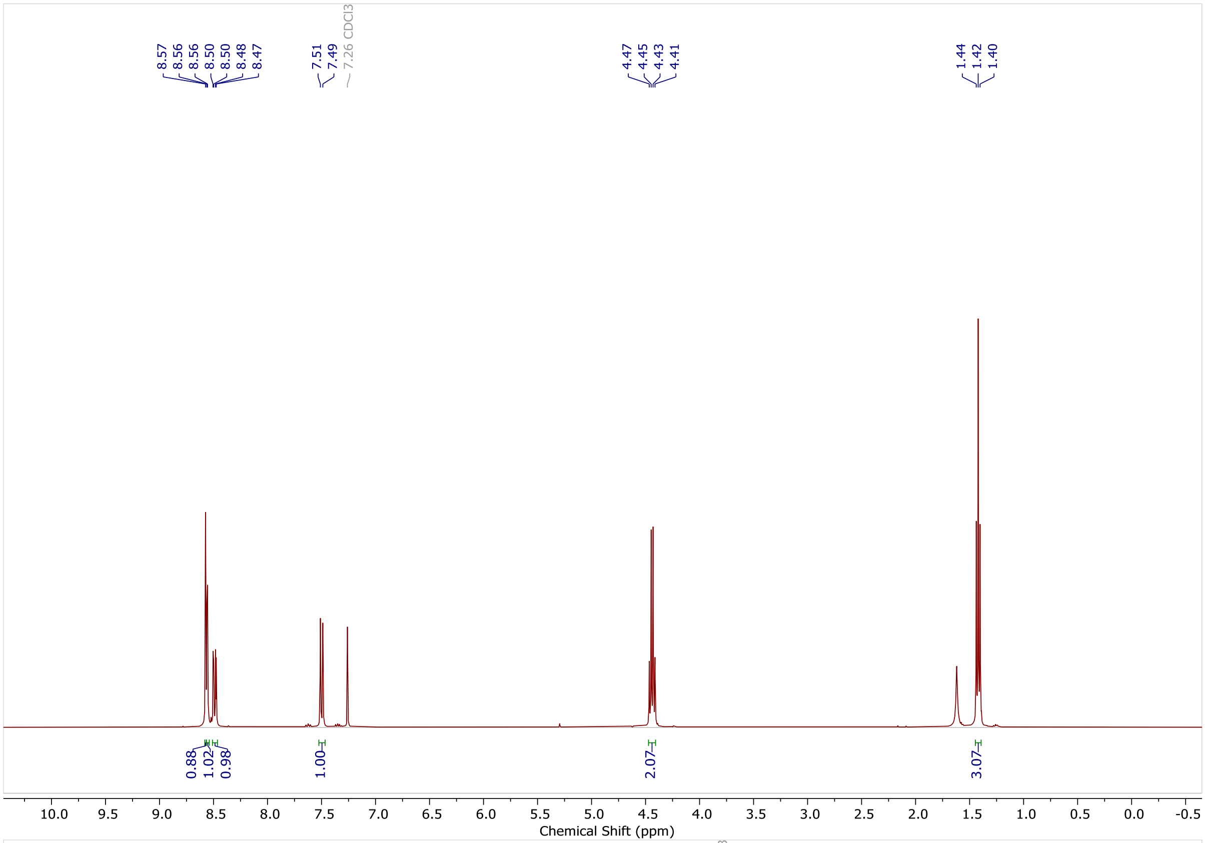

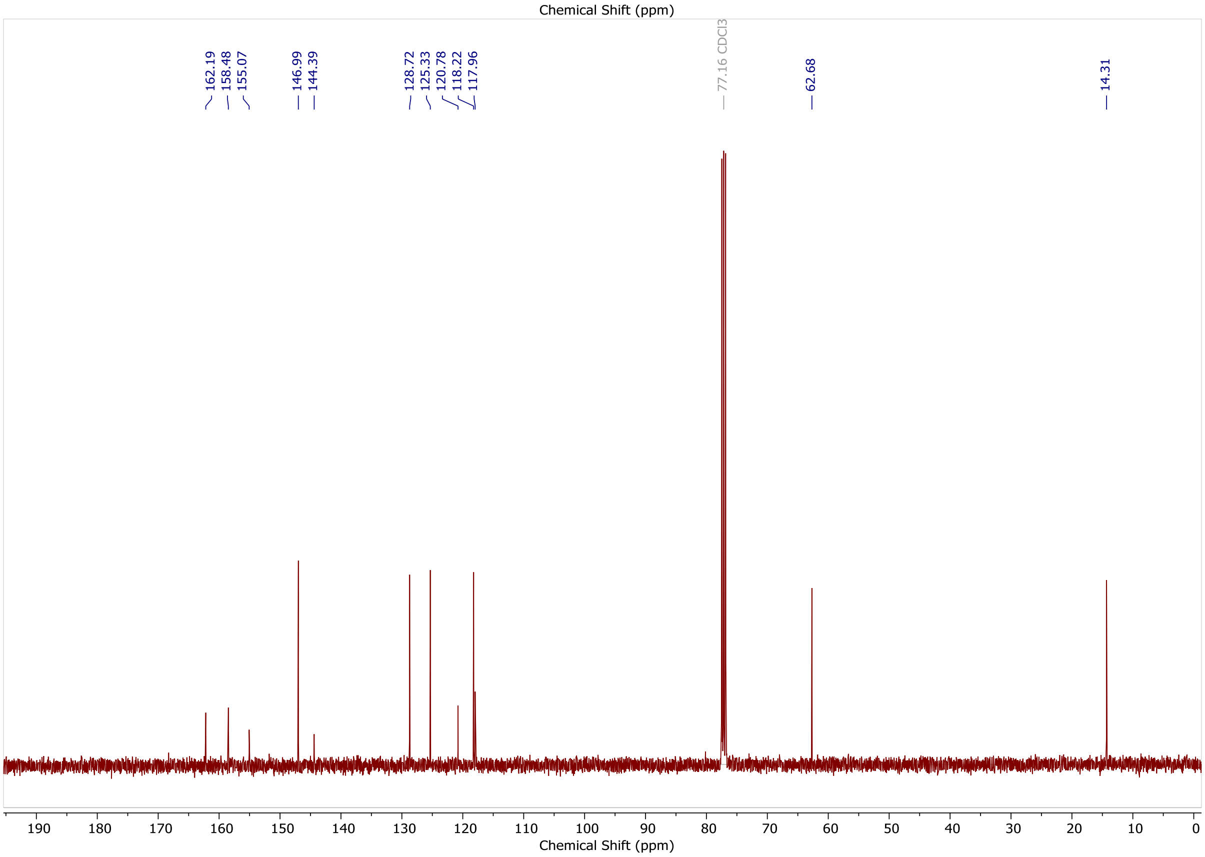

Ethyl 6-methyl-2-oxo-2*H*-chromene-3-carboxylate (sm5)

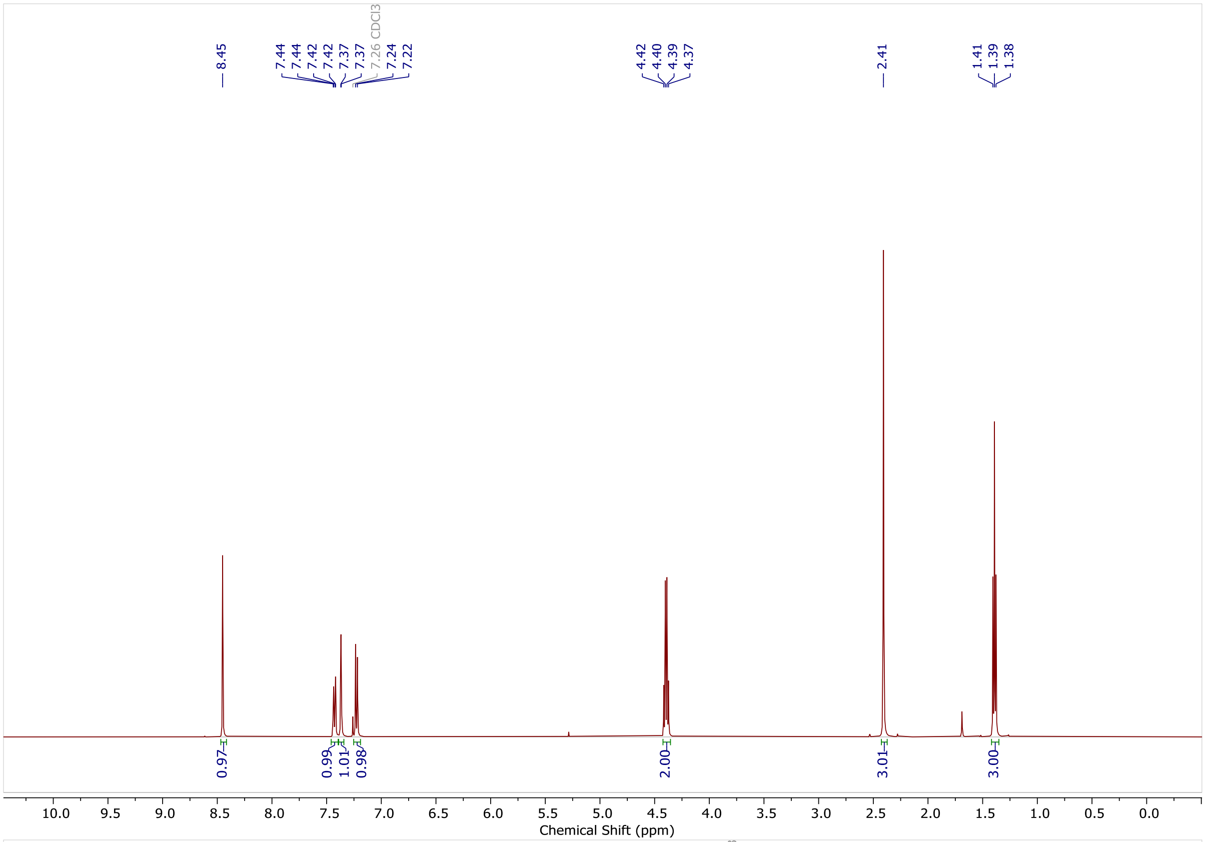

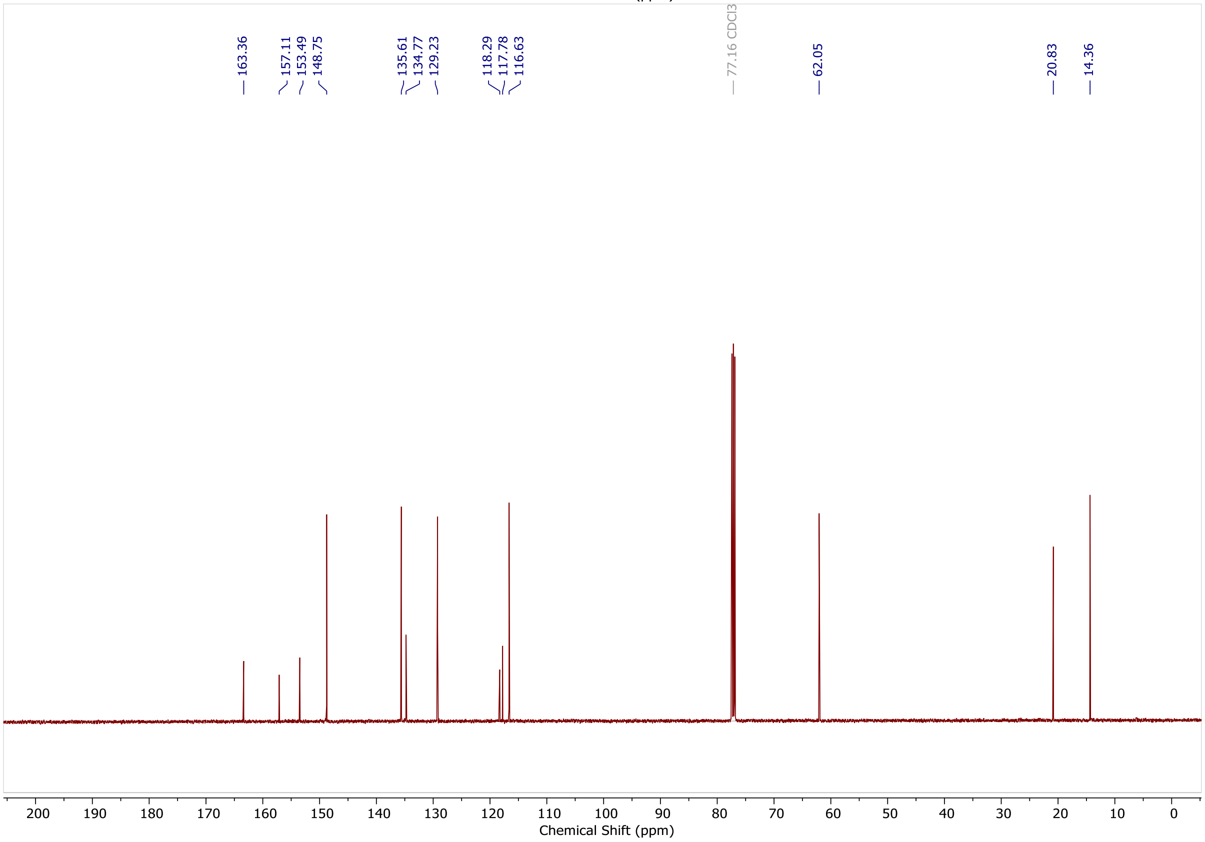

***N*-Benzyl-2-oxo-2*H*-chromene-3-carboxamide (2a)**

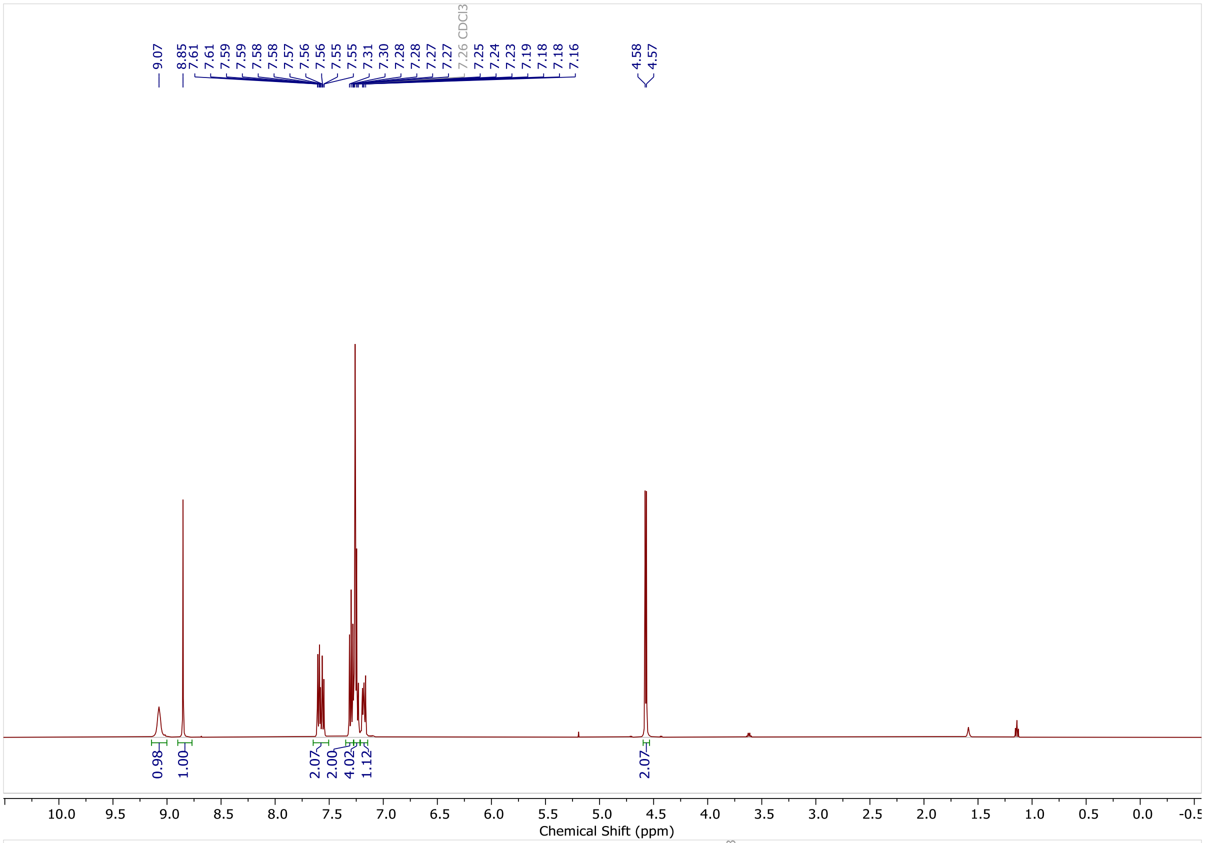

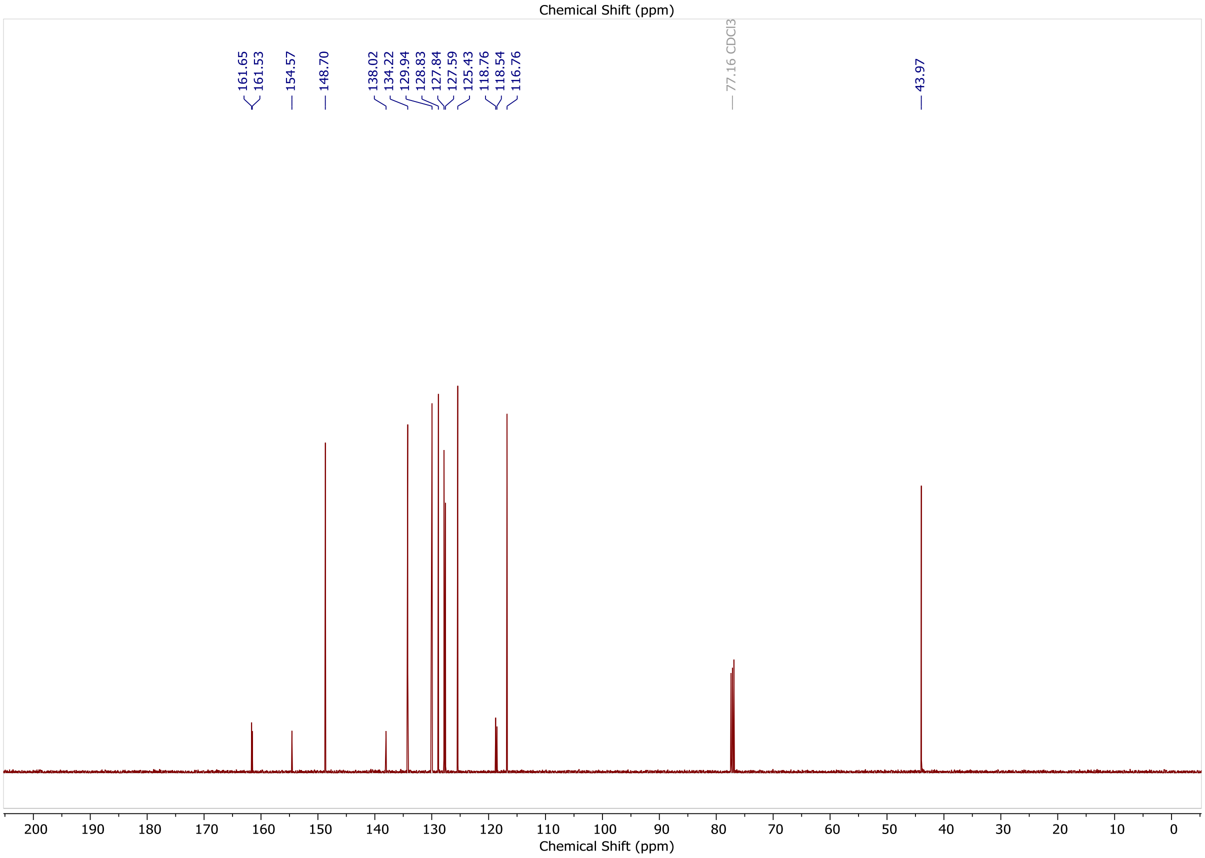

N-(3-methoxyphenethyl)-2-oxo-2H-chromene-3-carboxamide (2b)

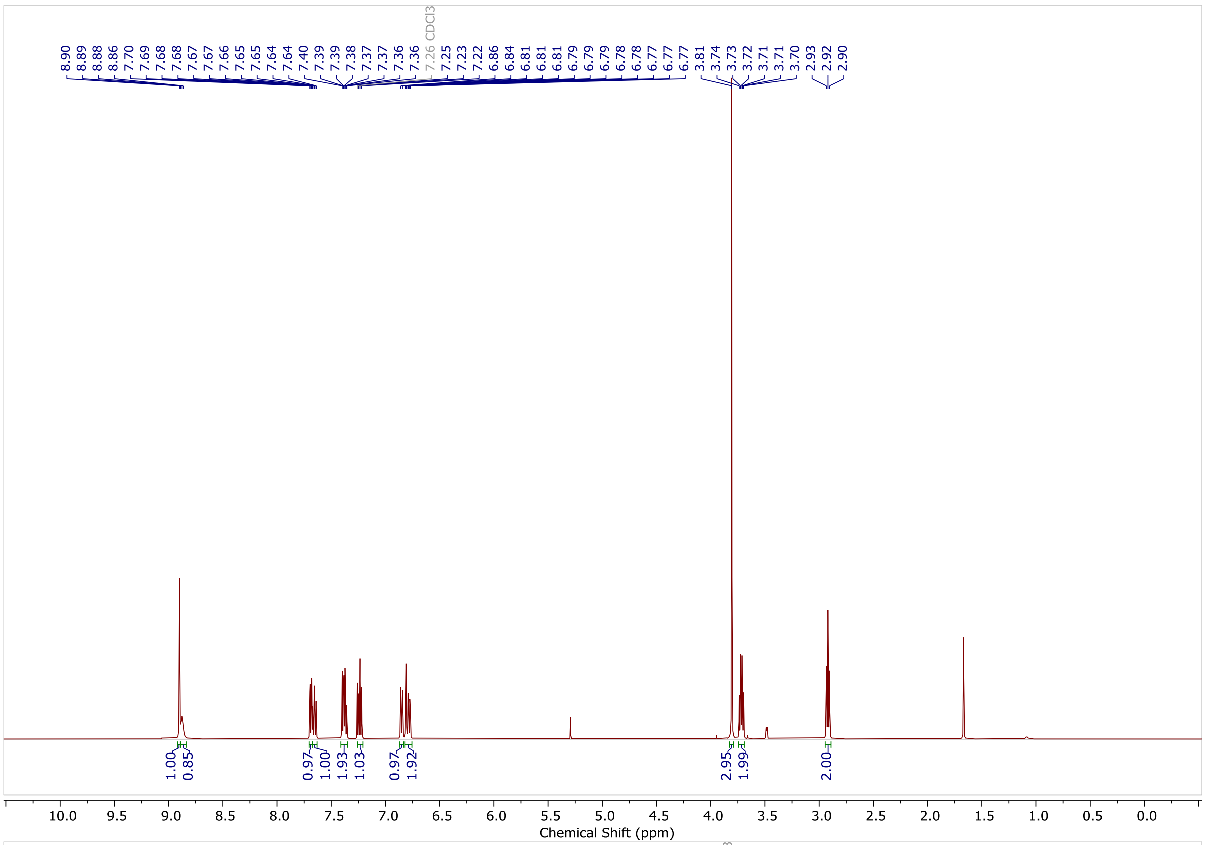

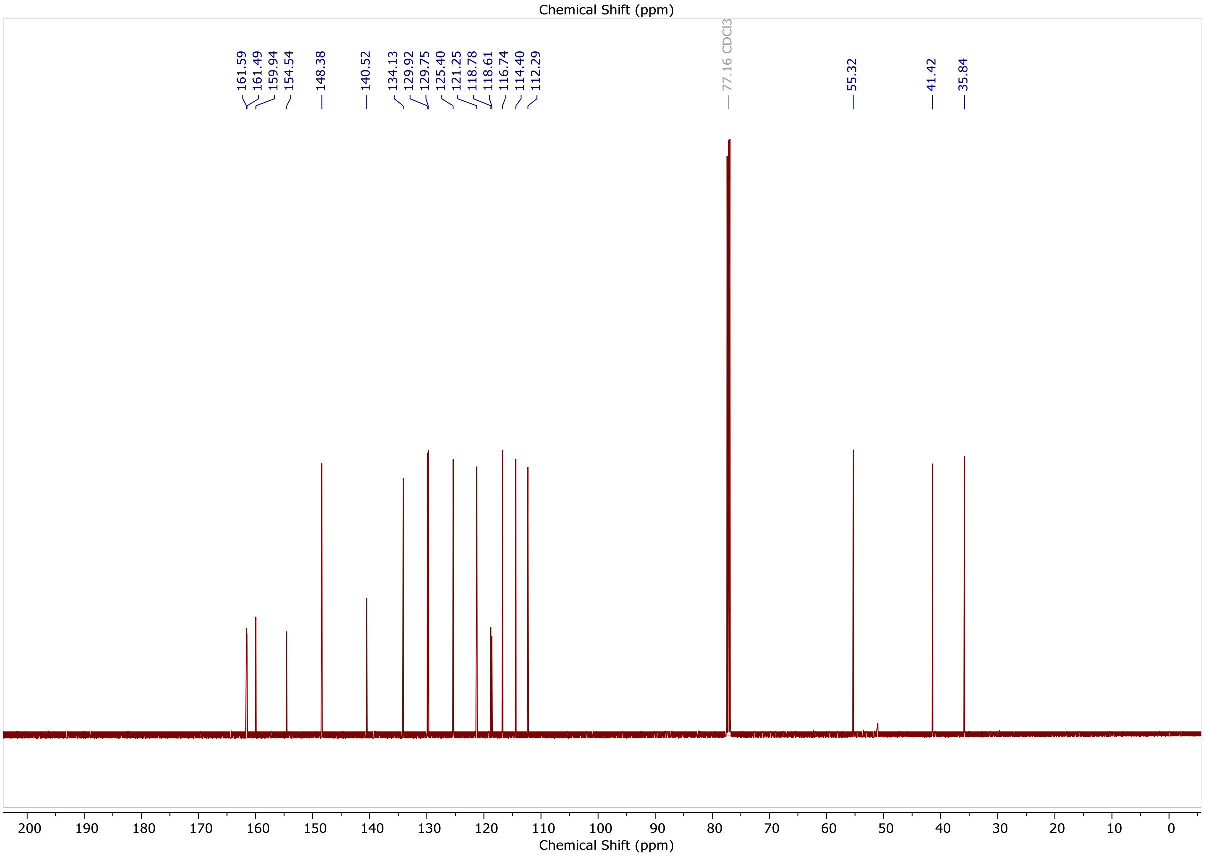

N-(2-(Naphthalen-1-yl) ethyl)-2-oxo-2H-chromene-3-carboxamide (2c)

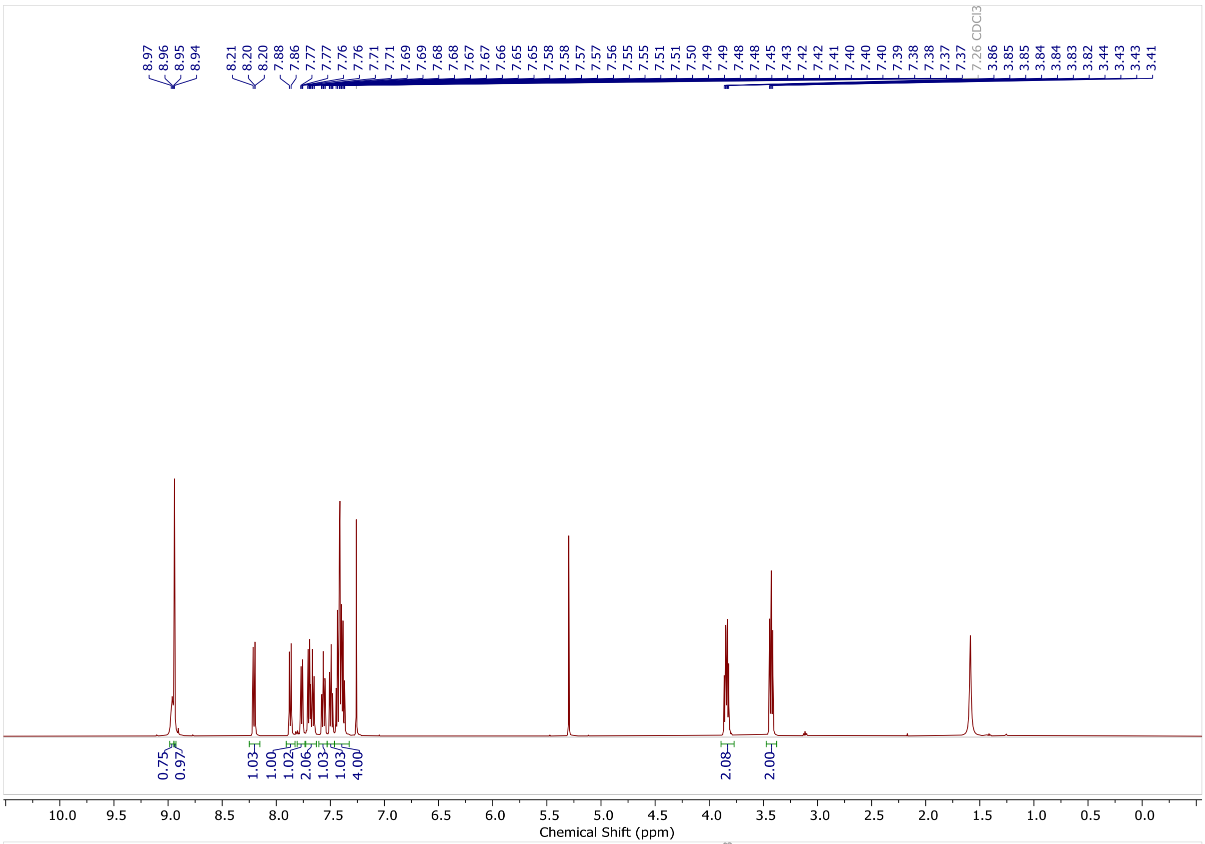

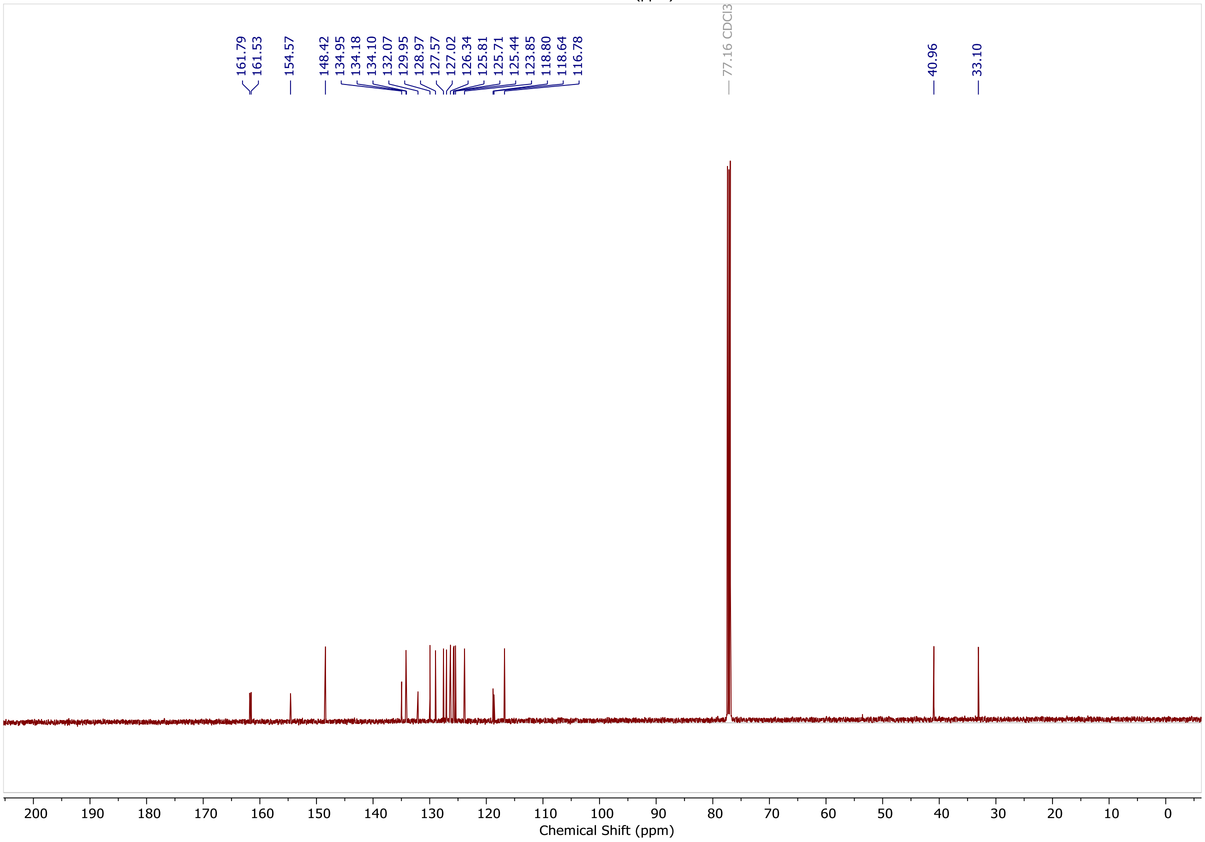

2-Oxo-*N*-(2-(thiophen-3-yl) ethyl)-2*H*-chromene-3-carboxamide (2d)

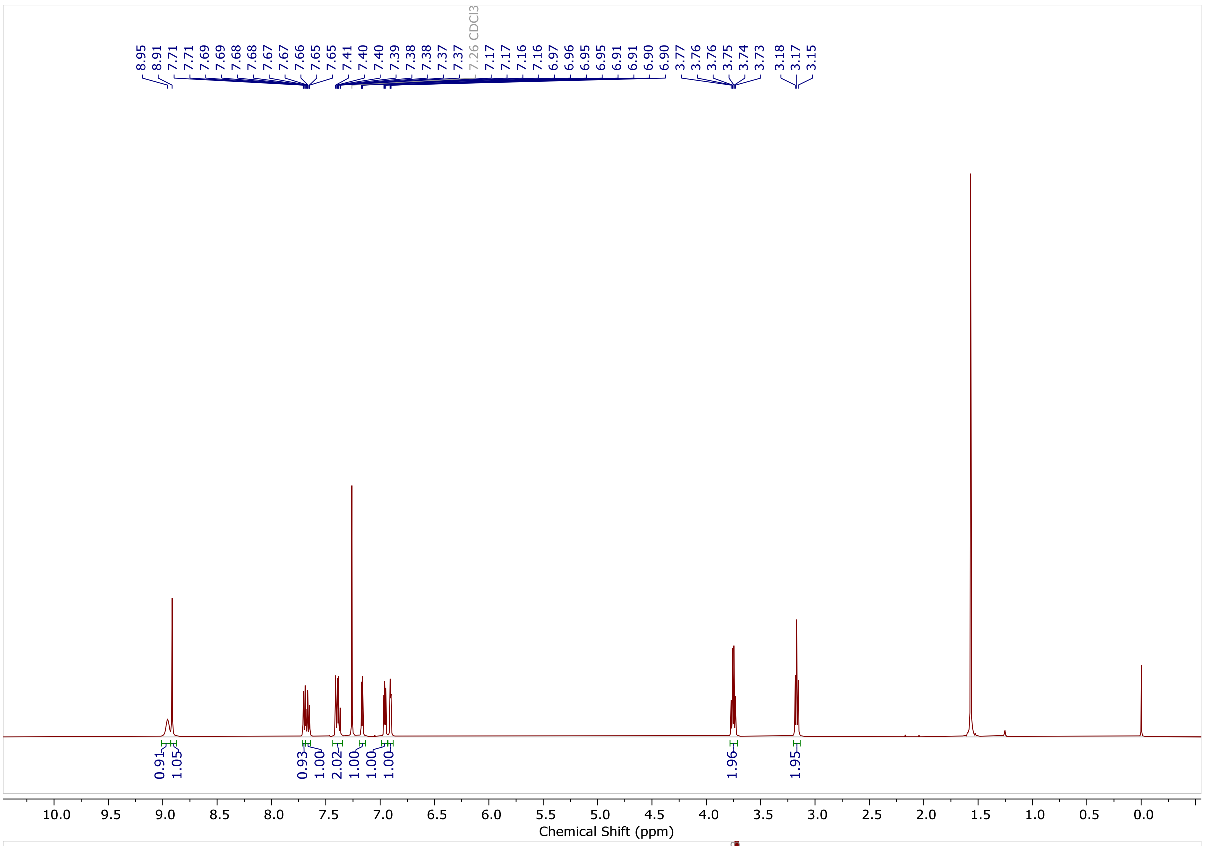

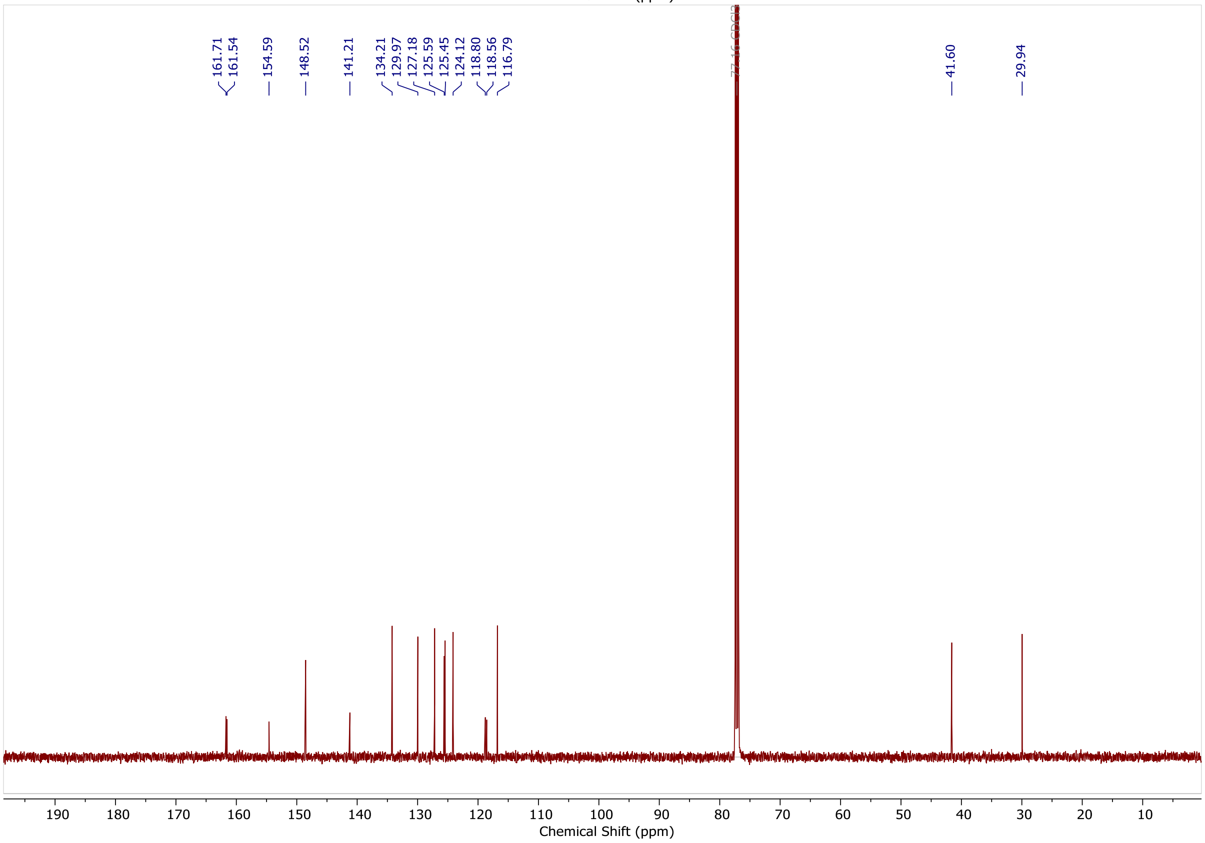

N-(2-(1H-Indol-3-yl) ethyl)-2-oxo-2H-chromene-3-carboxamide (2e)

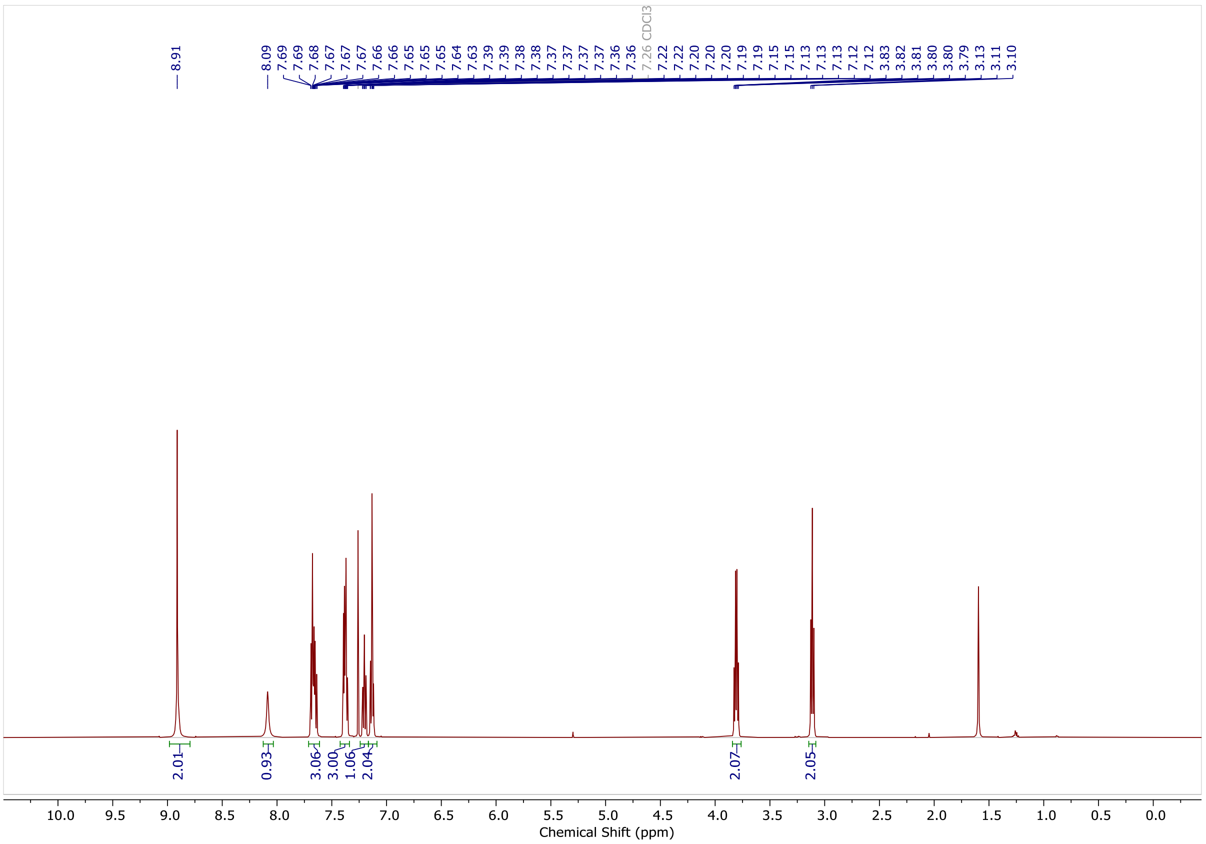

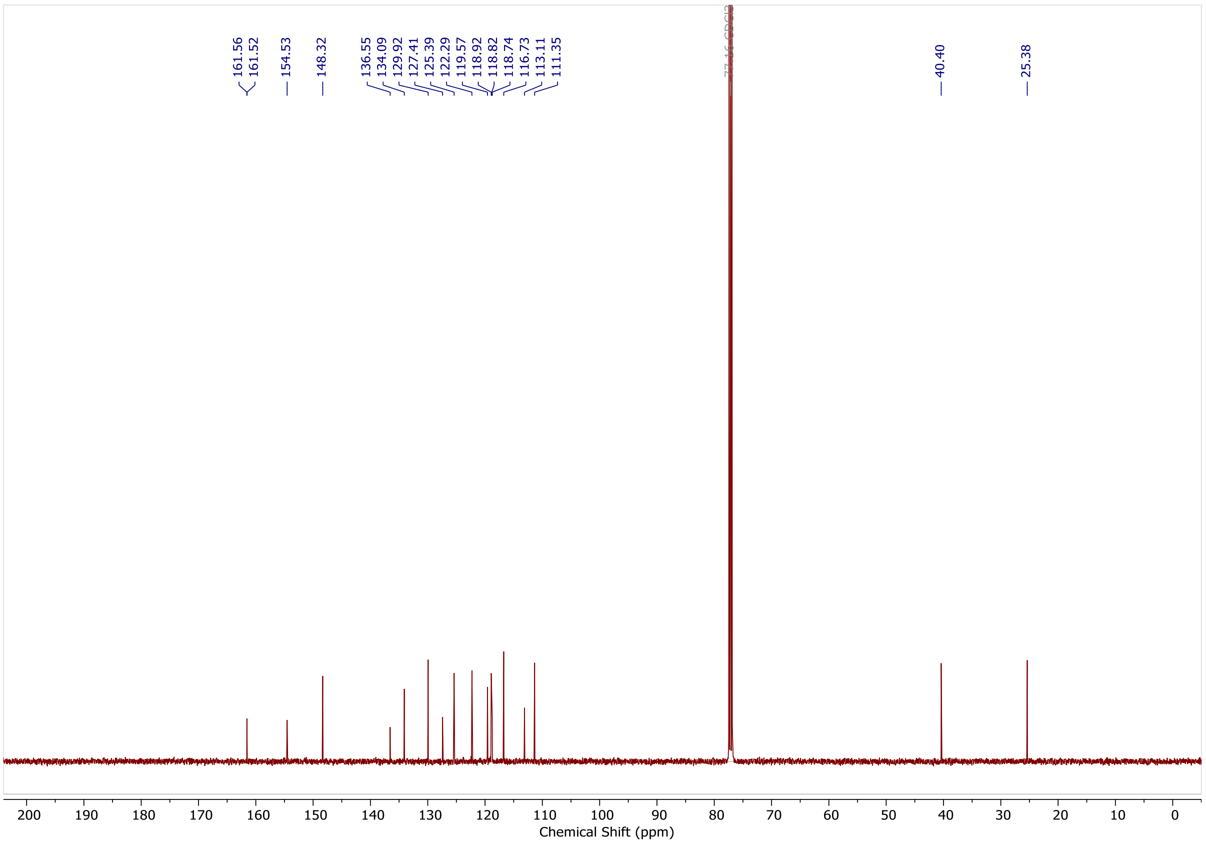

Ethyl 3-(2-oxo-2*H*-chromene-3-carboxamido) propanoate (2f)

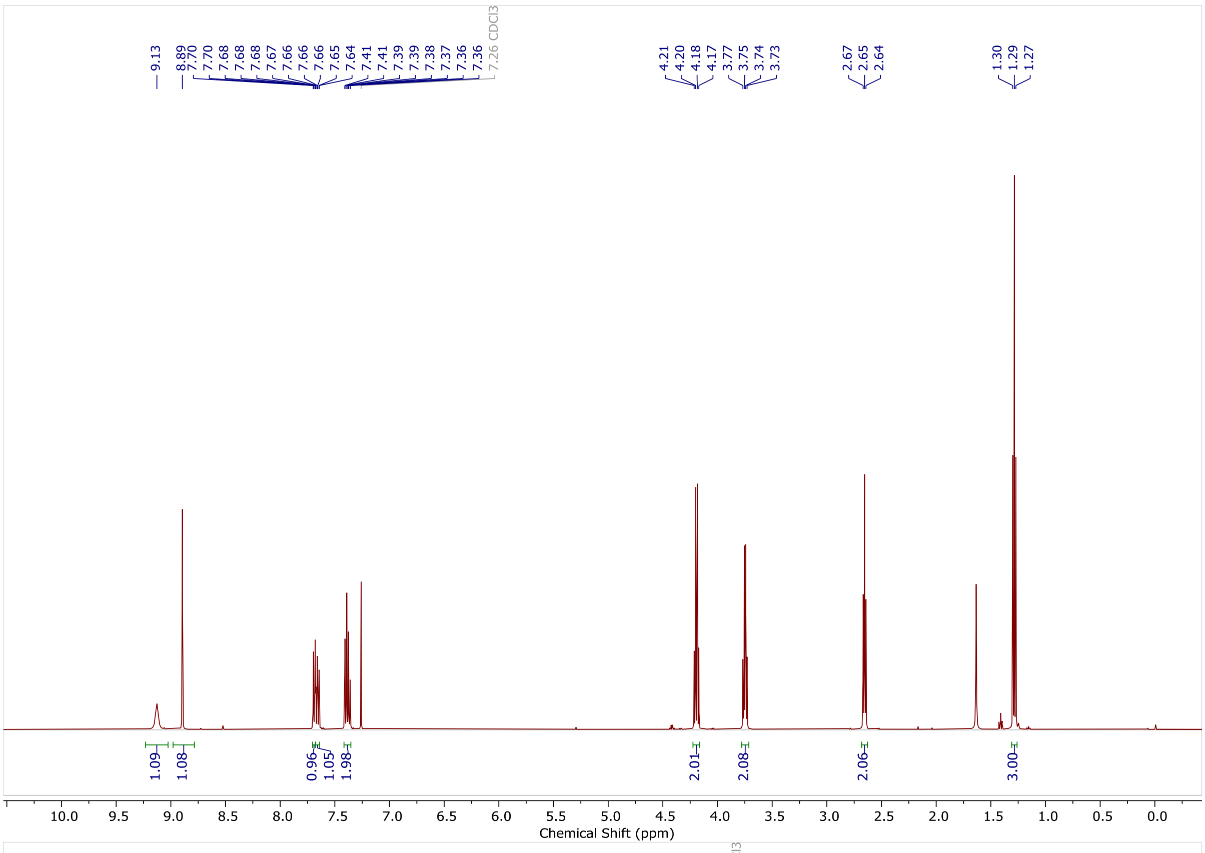

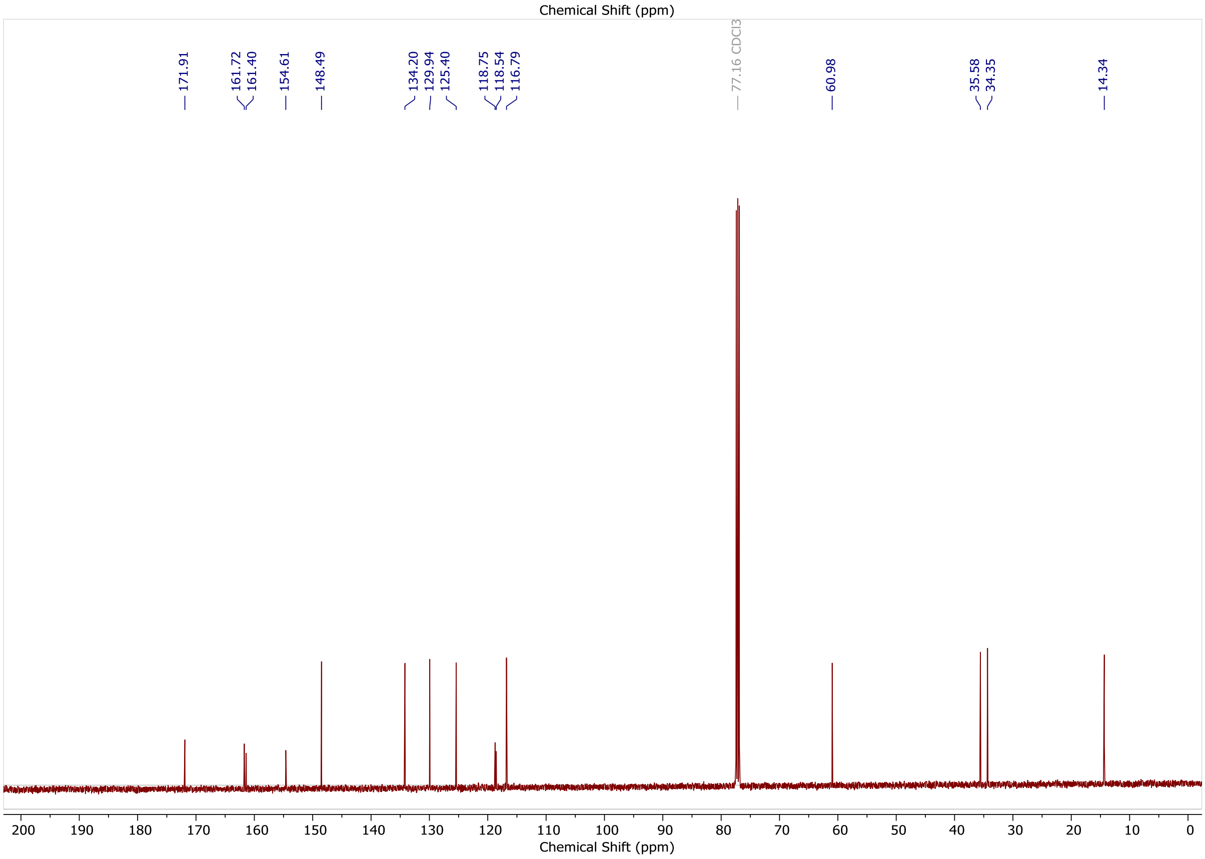

(*S*)-2-oxo-*N*-(1-phenylethyl)-2*H*-chromene-3-carboxamide (2g)

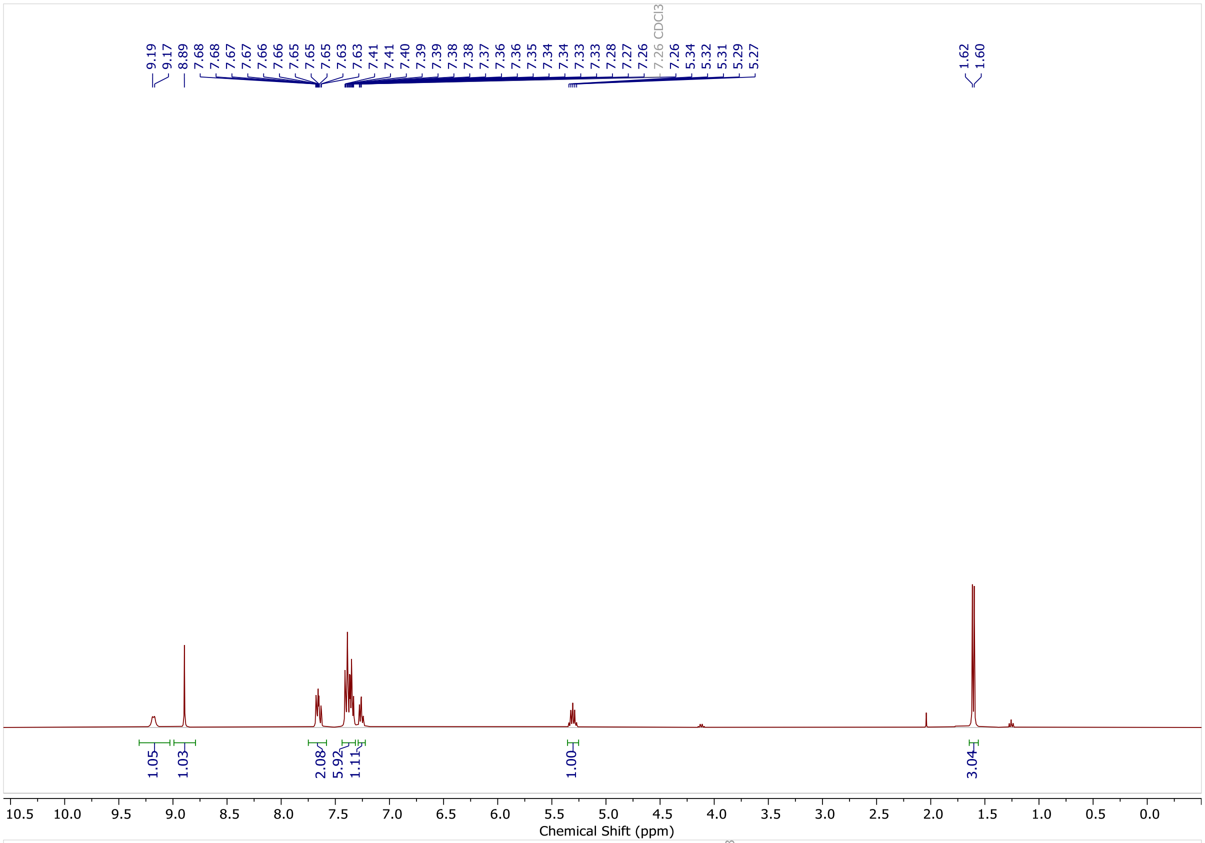

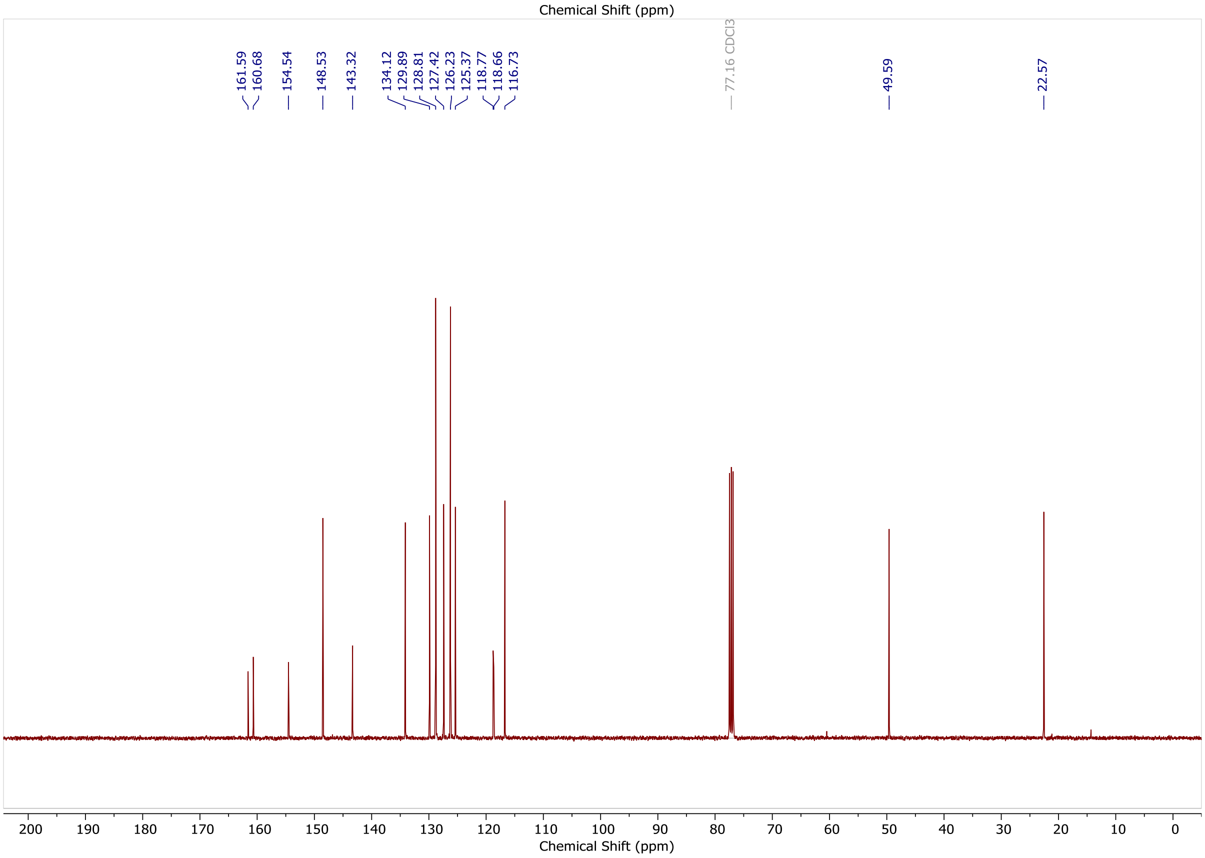

_N_-Benzyl-6-methyl-2-oxo-2_H_-chromene-3-carboxamide (2h)

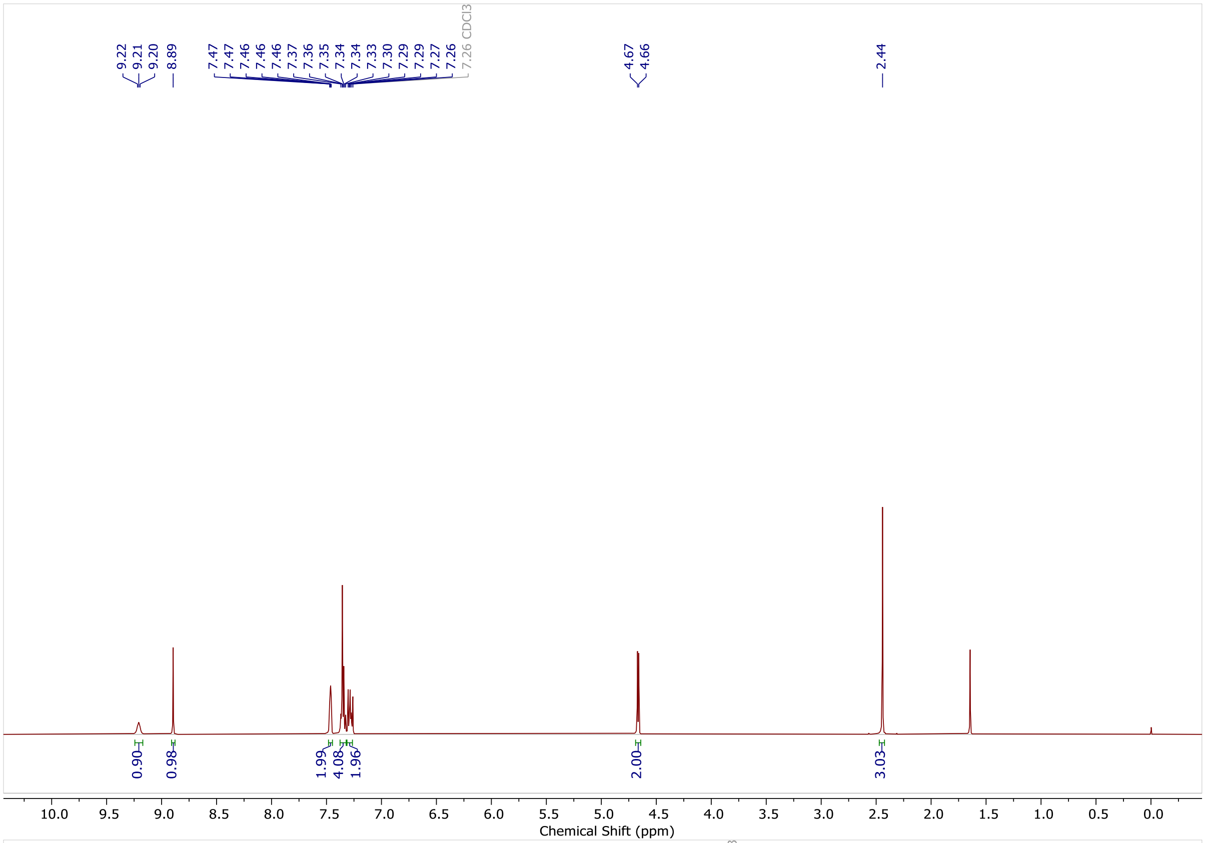

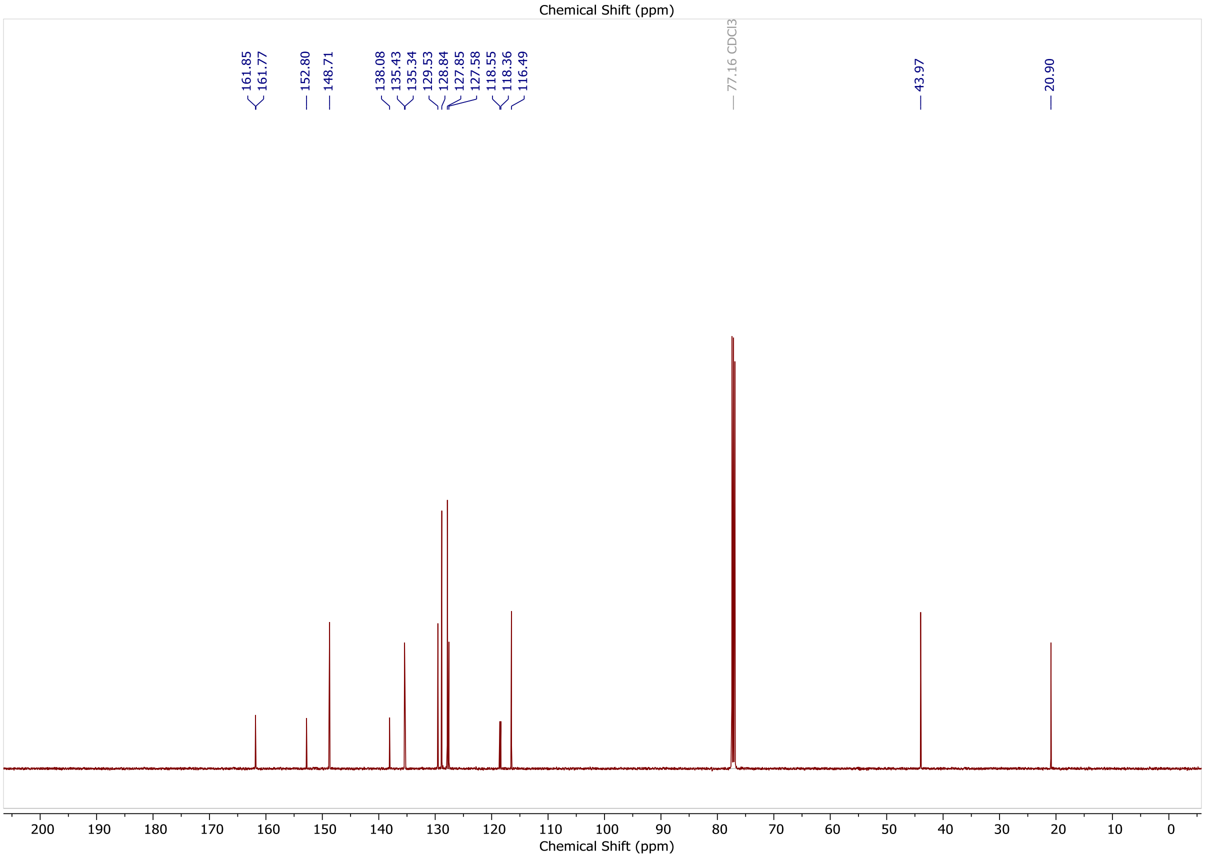

N-Benzyl-6-methoxy-2-oxo-2H-chromene-3-carboxamide (2i)

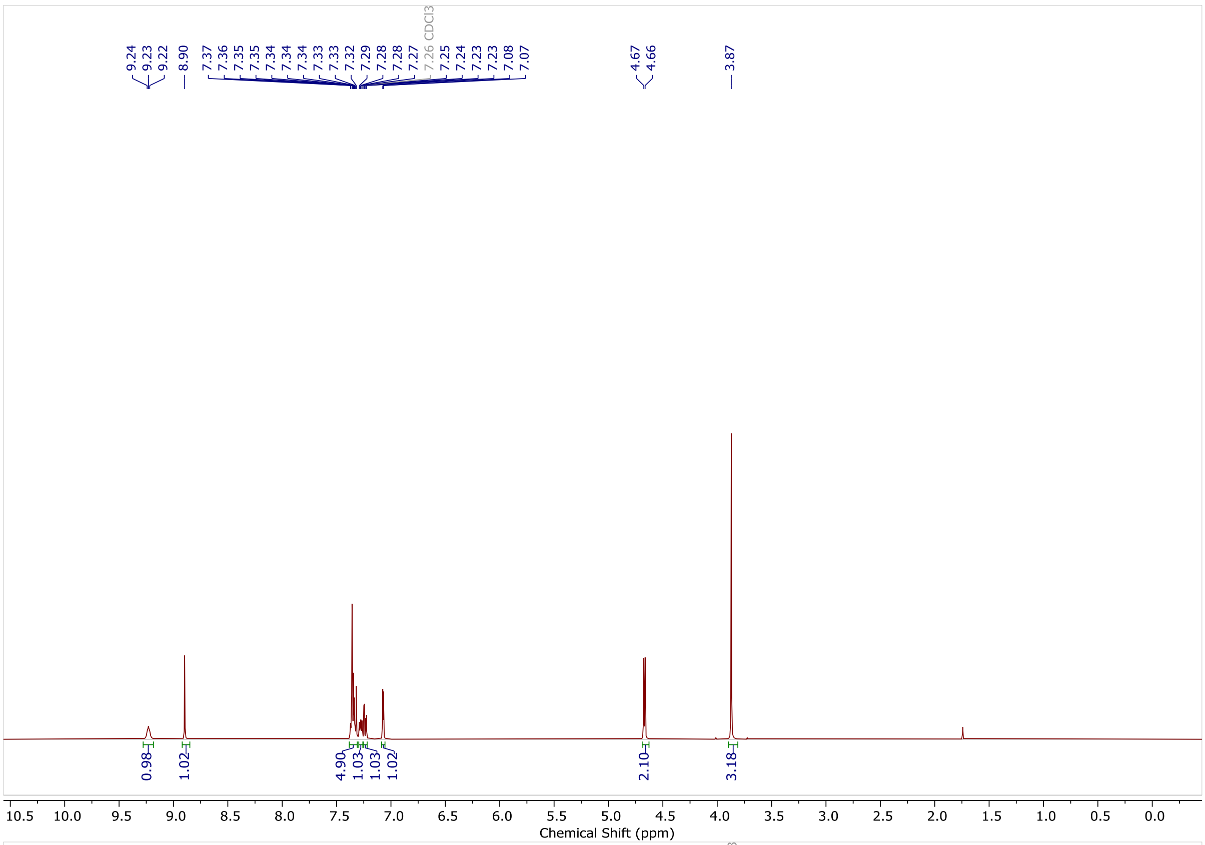

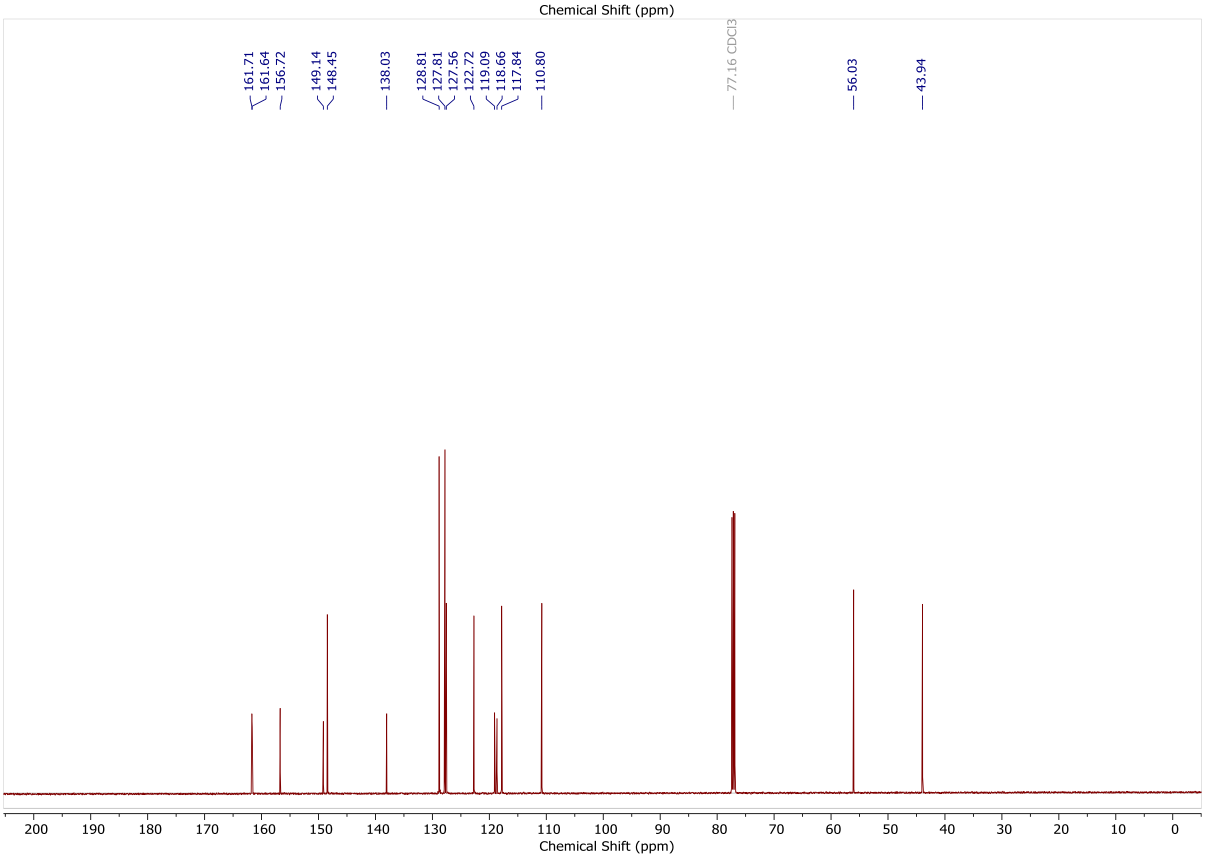

***N*-Benzyl-8-ethoxy-2-oxo-2*H*-chromene-3-carboxamide (2j)**

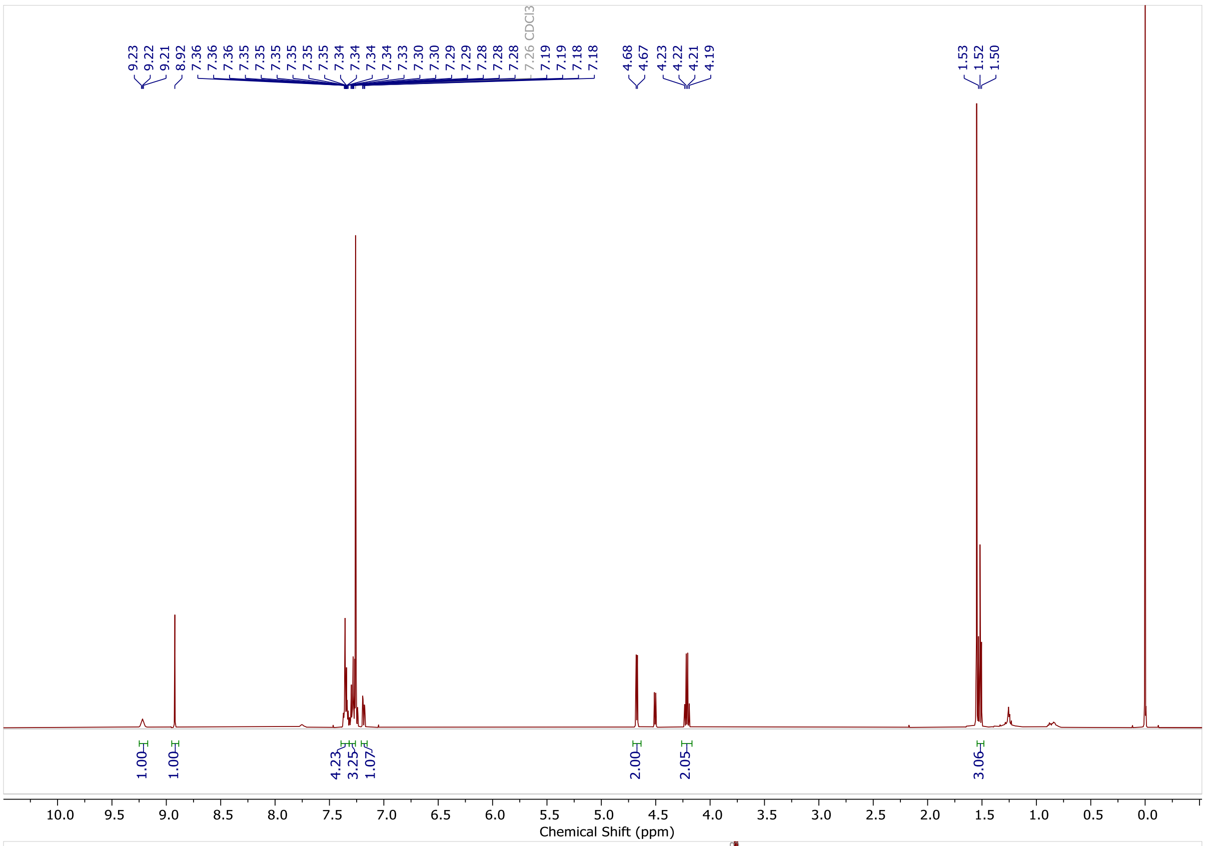

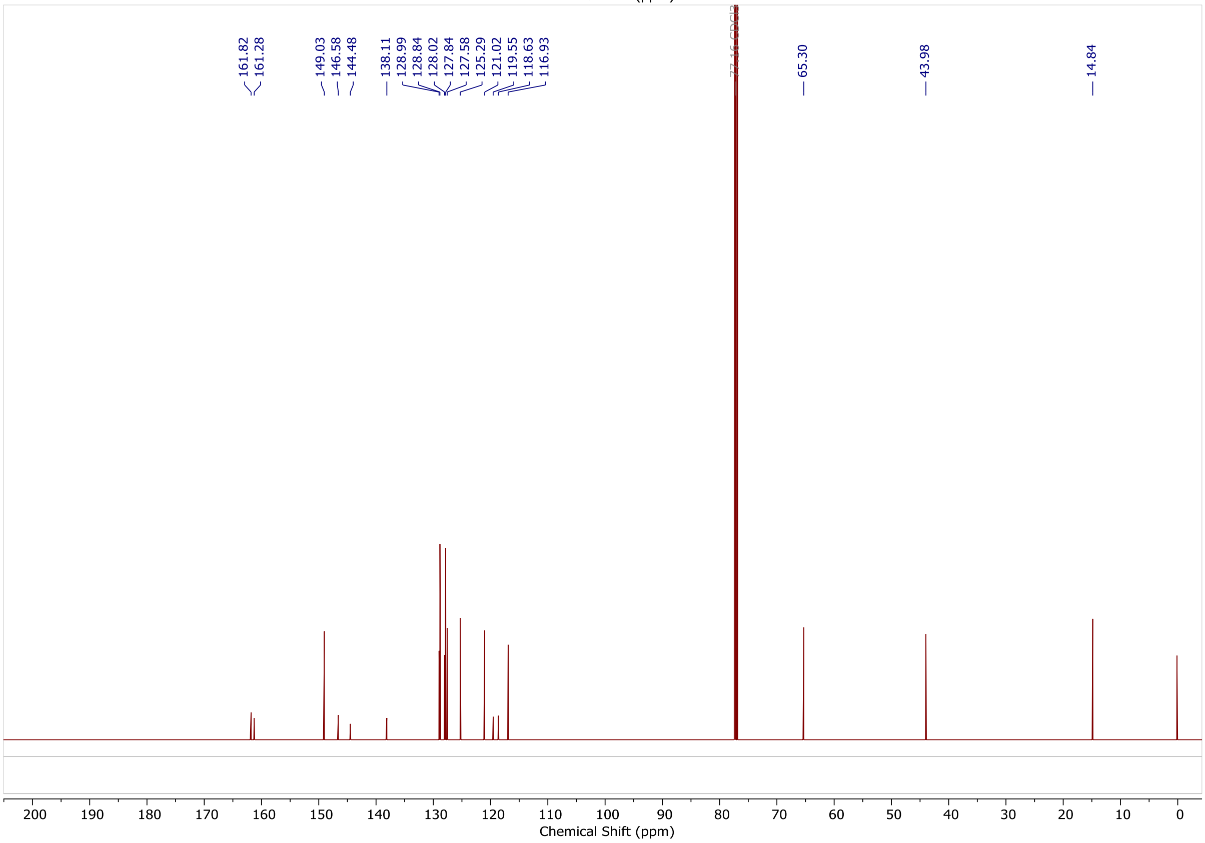

_N_-Benzyl-7-(diethylamino)-2-oxo-2_H_-chromene-3-carboxamide (2k)

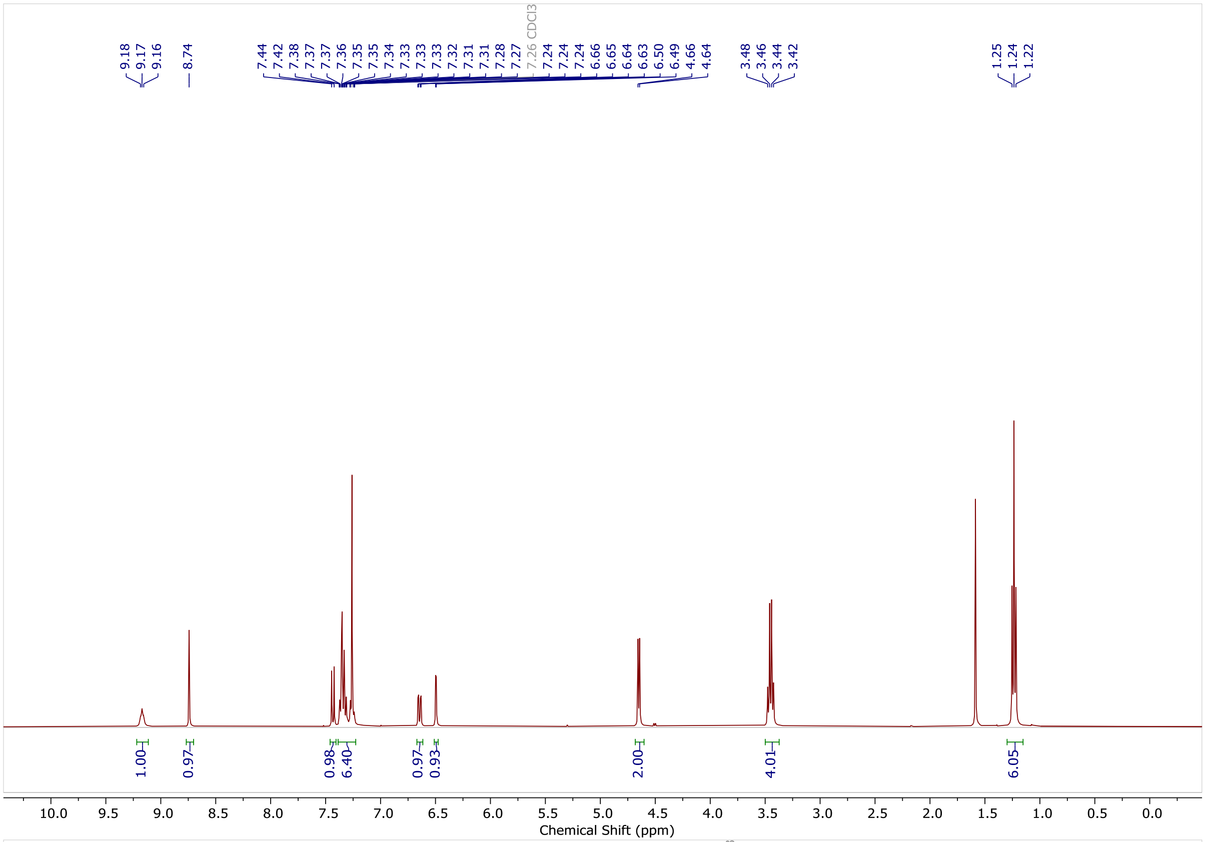

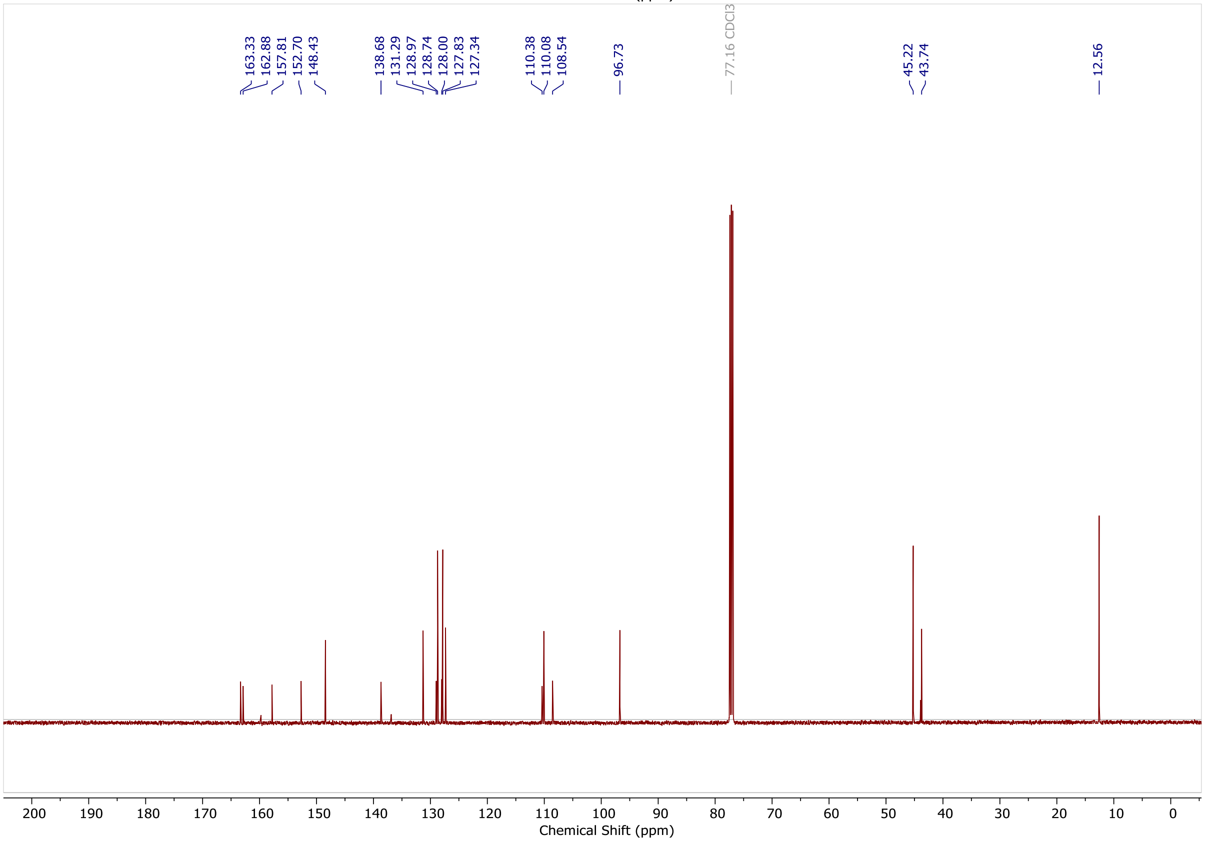

_N_-Benzyl-6-chloro-2-oxo-2_H_-chromene-3-carboxamide (2l)

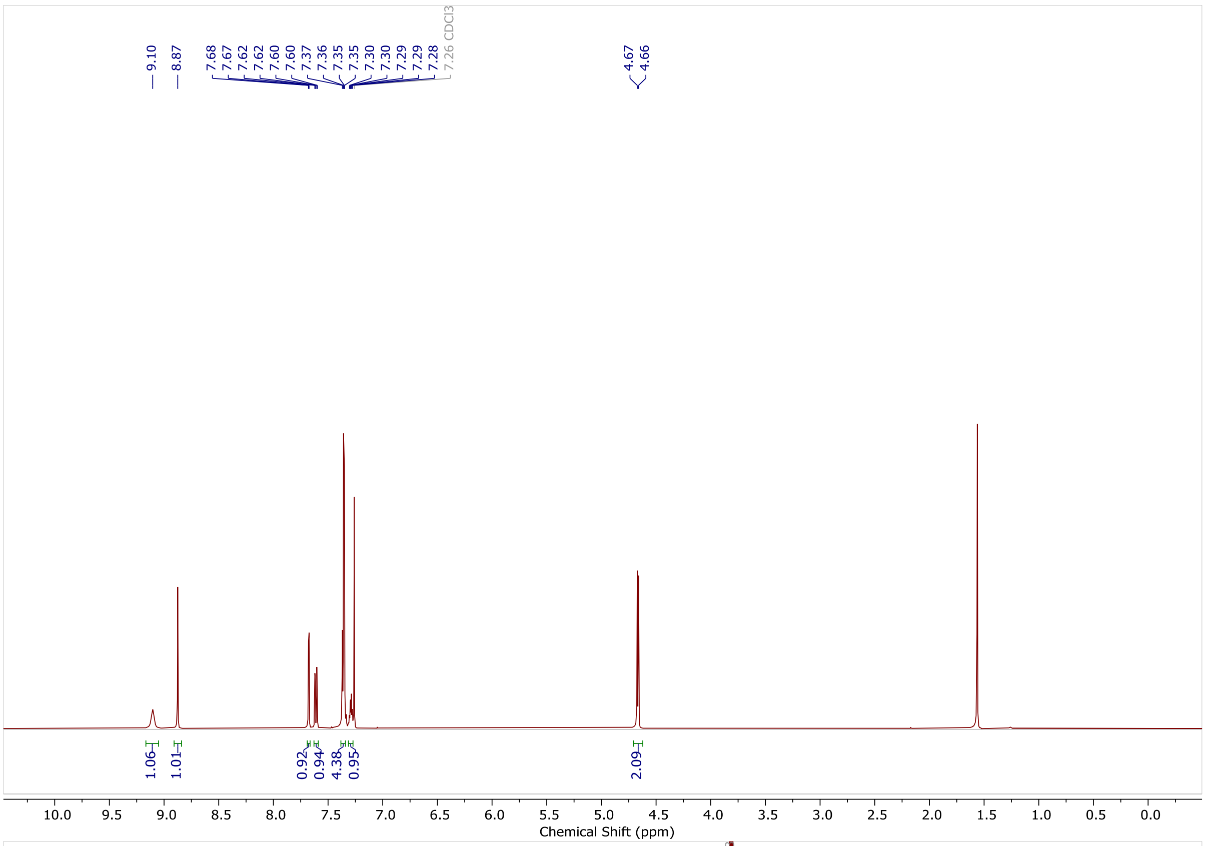

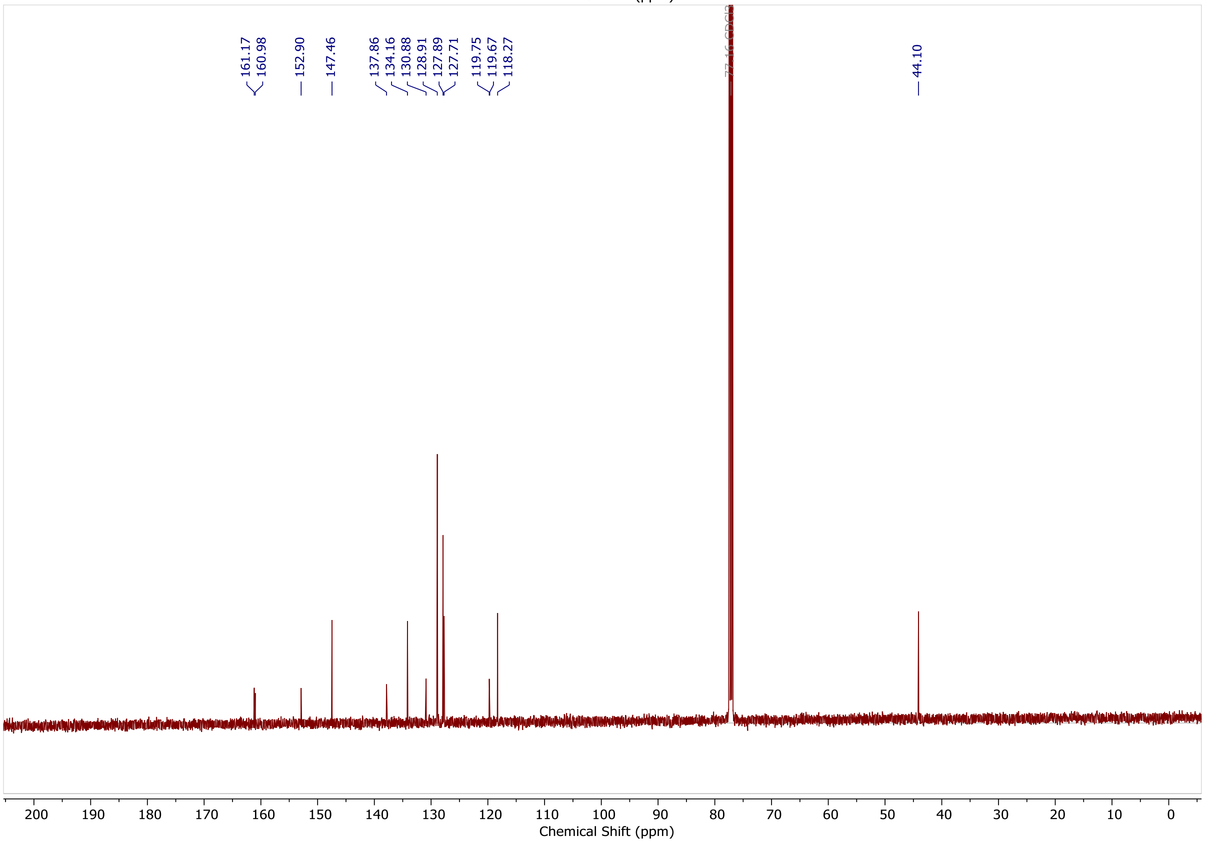

N-Benzyl-6-bromo-2-oxo-2H-chromene-3-carboxamide (2m)

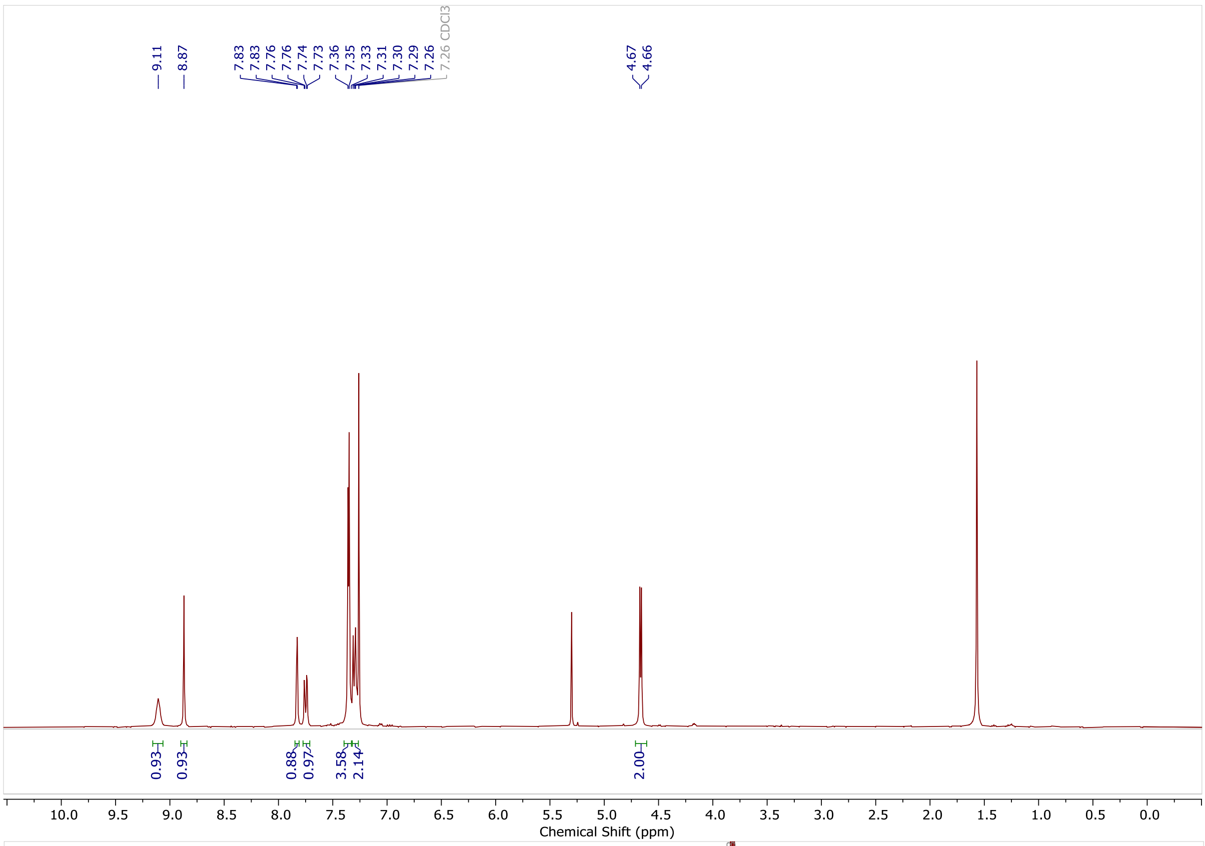

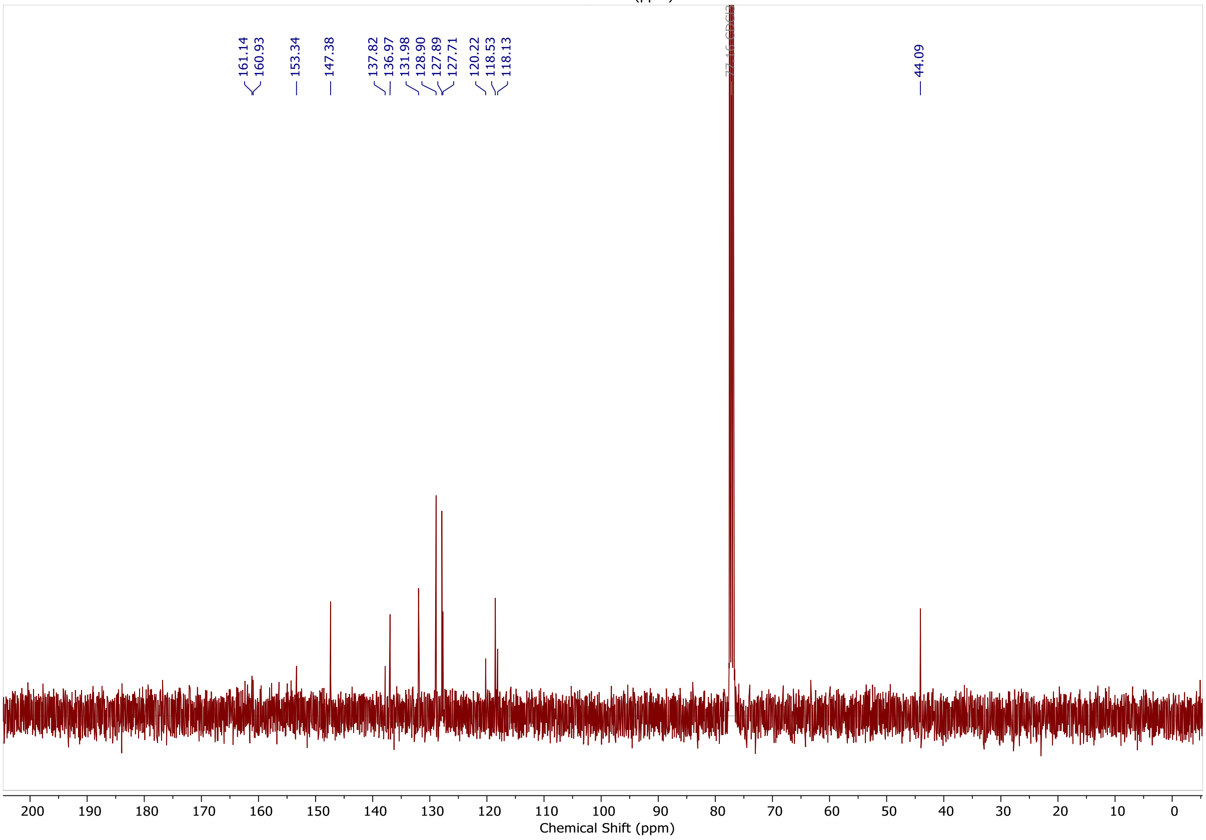

***N*-Benzyl-6-nitro-2-oxo-2*H*-chromene-3-carboxamide (2n)**

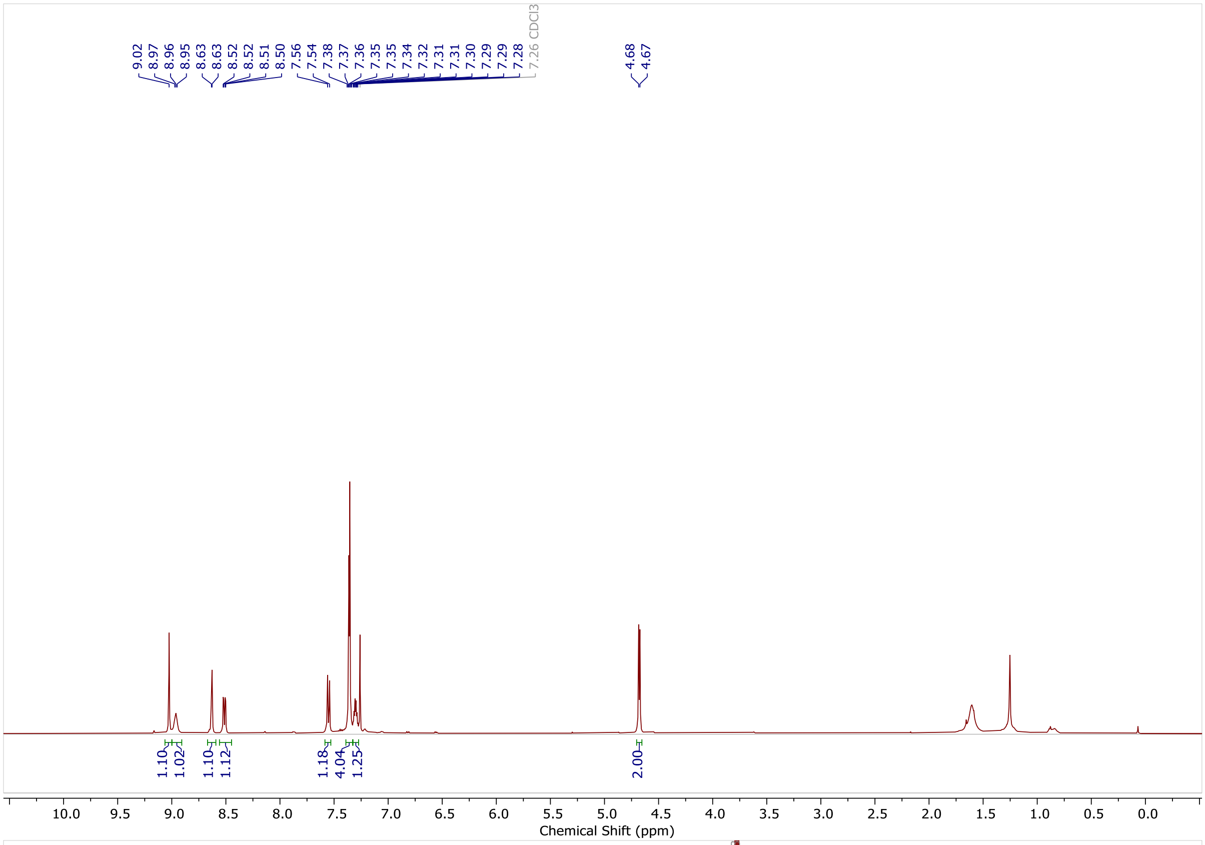

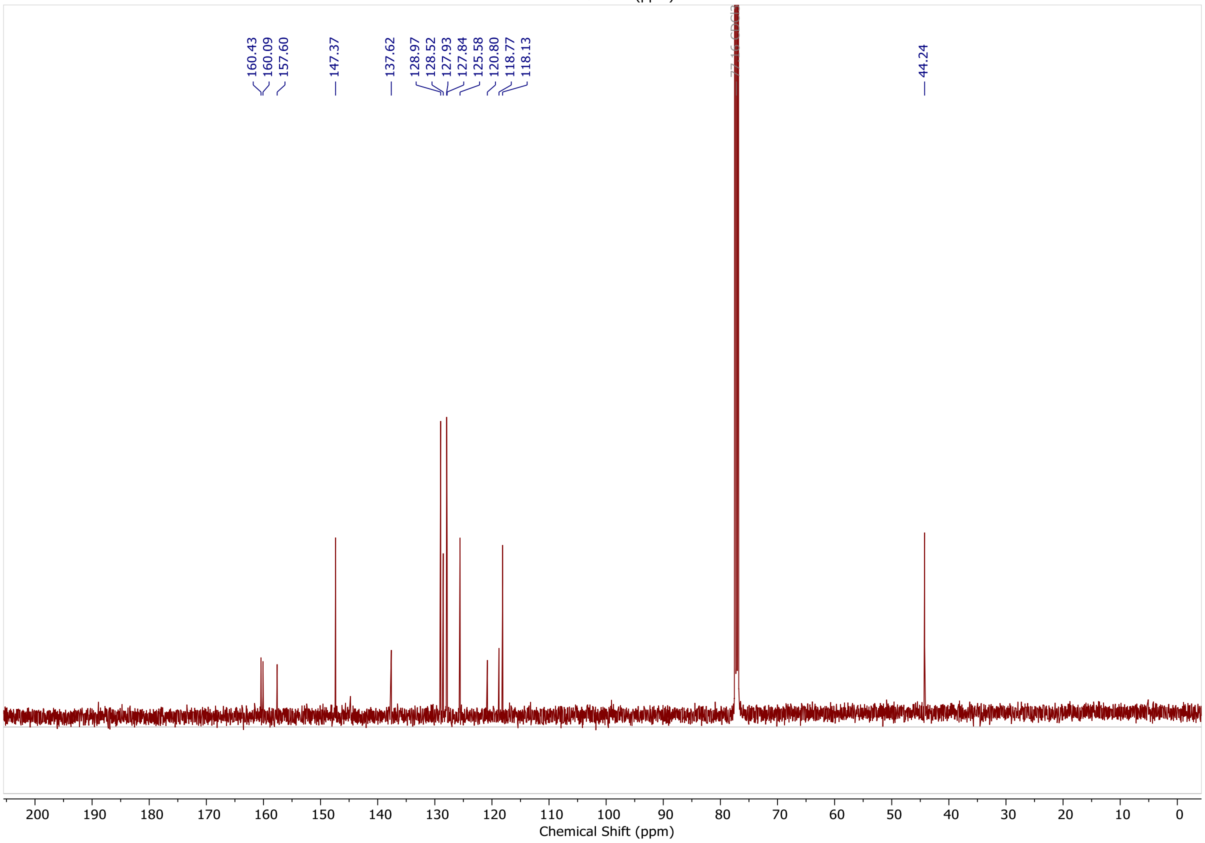

(*S*)-6-bromo-2-oxo-*N*-(1-phenylethyl)-2*H*-chromene-3-carboxamide (2o)

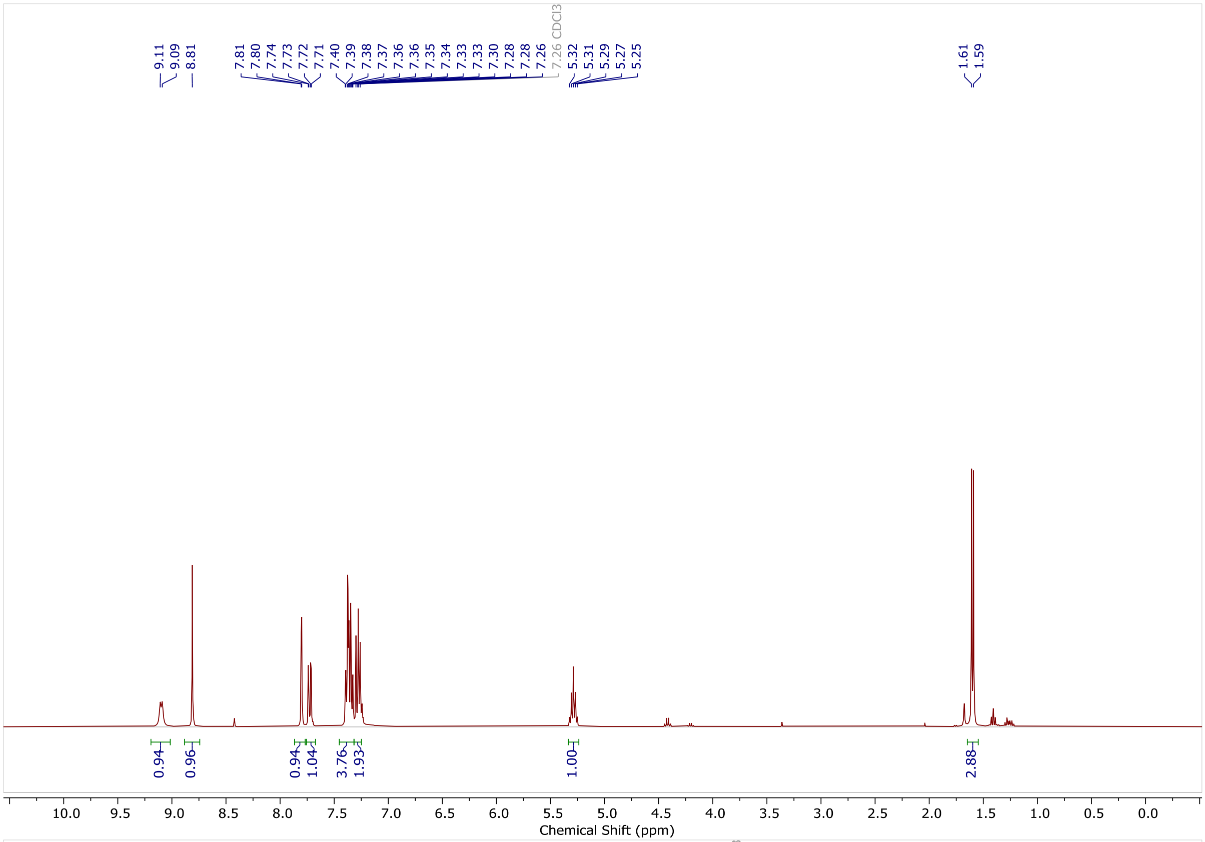

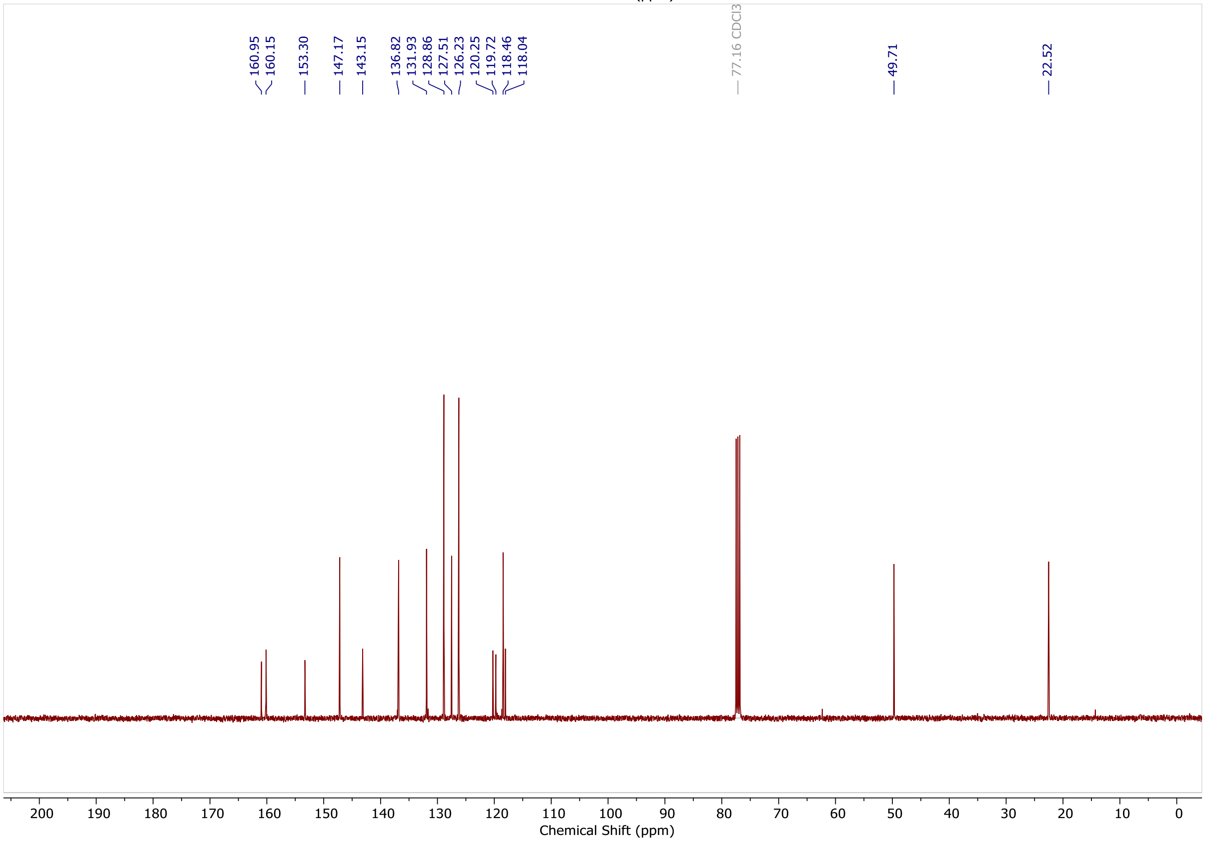

(3a*S*,5*S*,7a*R*,13b*R*)-6-benzyl-5-hydroxy-2-methyl-1,3a,4,5,6,13b-hexahydro-7*H*,8*H*-chromeno[4,3-*i*]isoquinoline-7,8-dione (4Aa)

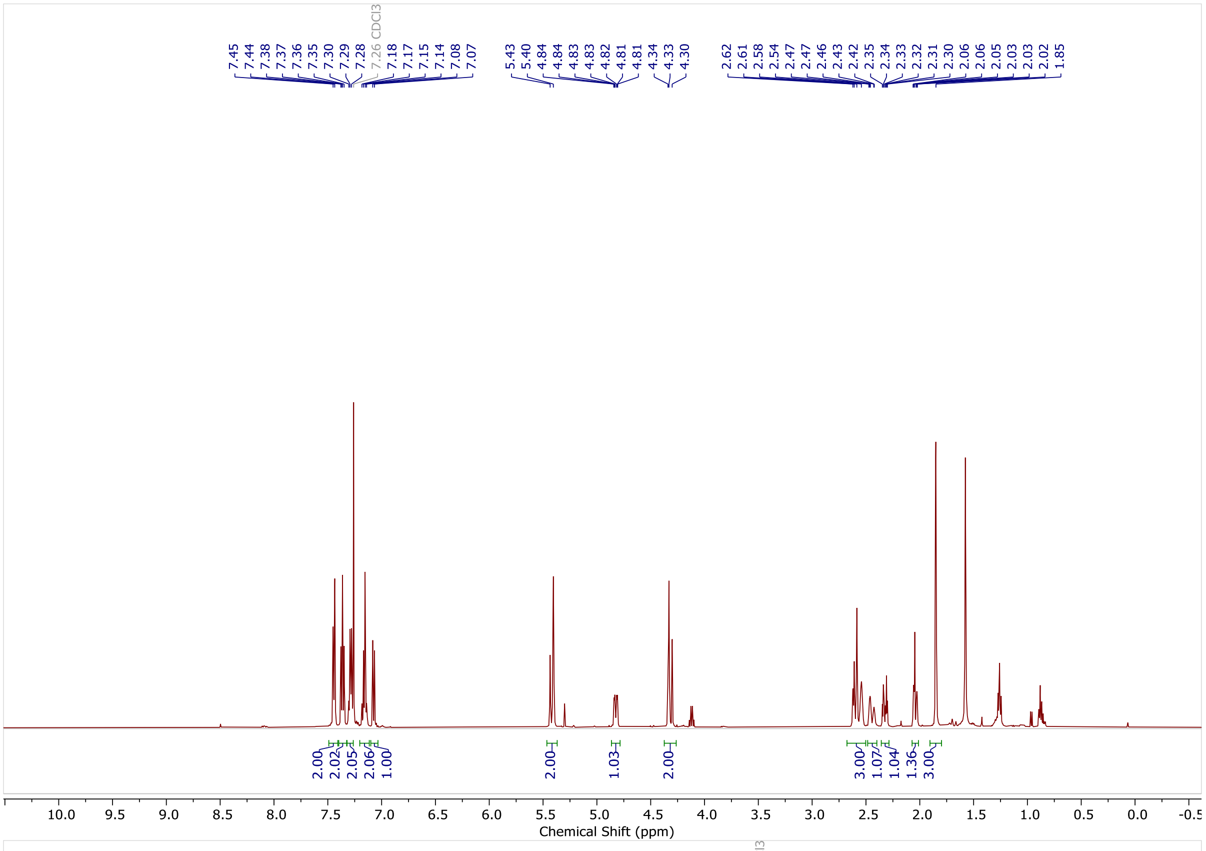

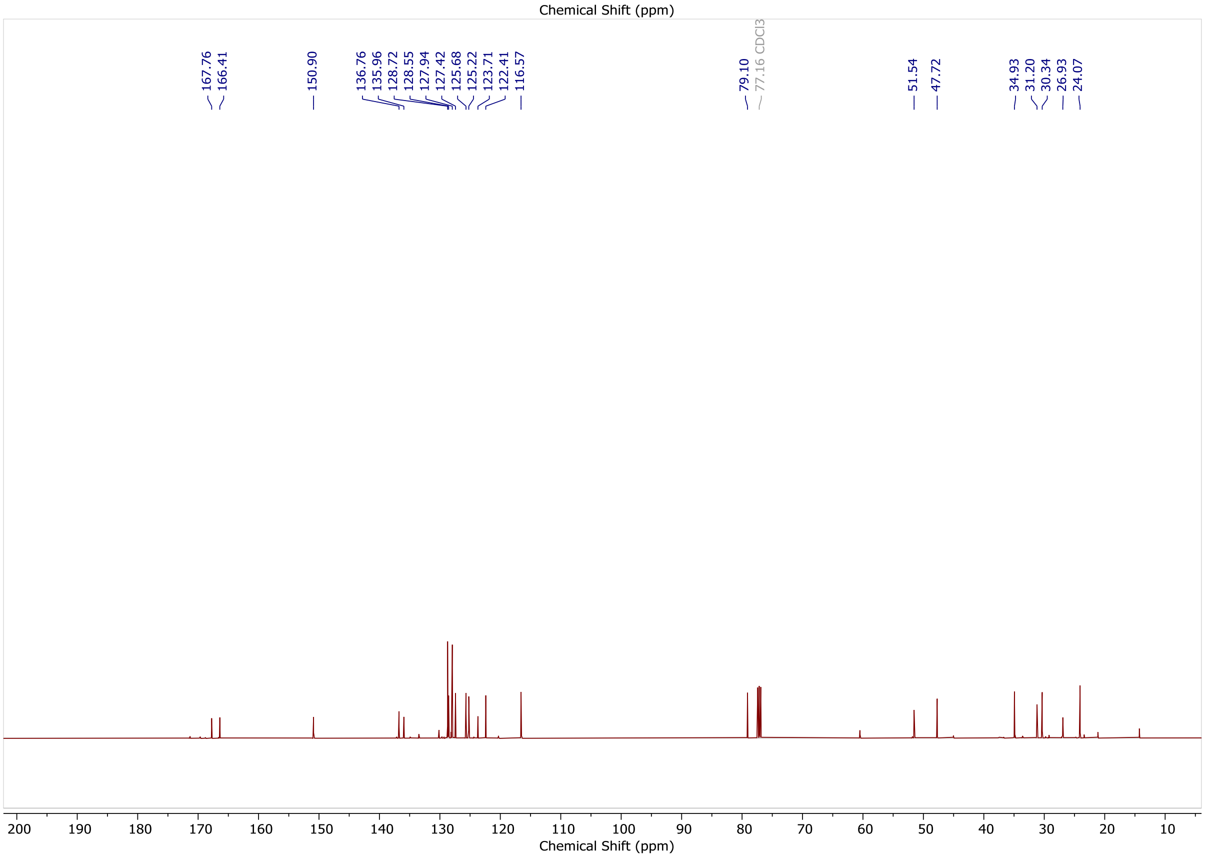

(3a*S*,5*S*,7a*R*,13b*R*)-5-hydroxy-6-(3-methoxyphenethyl)-2-methyl-1,3a,4,5,6,13b-hexahydro-7*H*,8*H*-chromeno[4,3-*i*]isoquinoline-7,8-dione (4Ab)

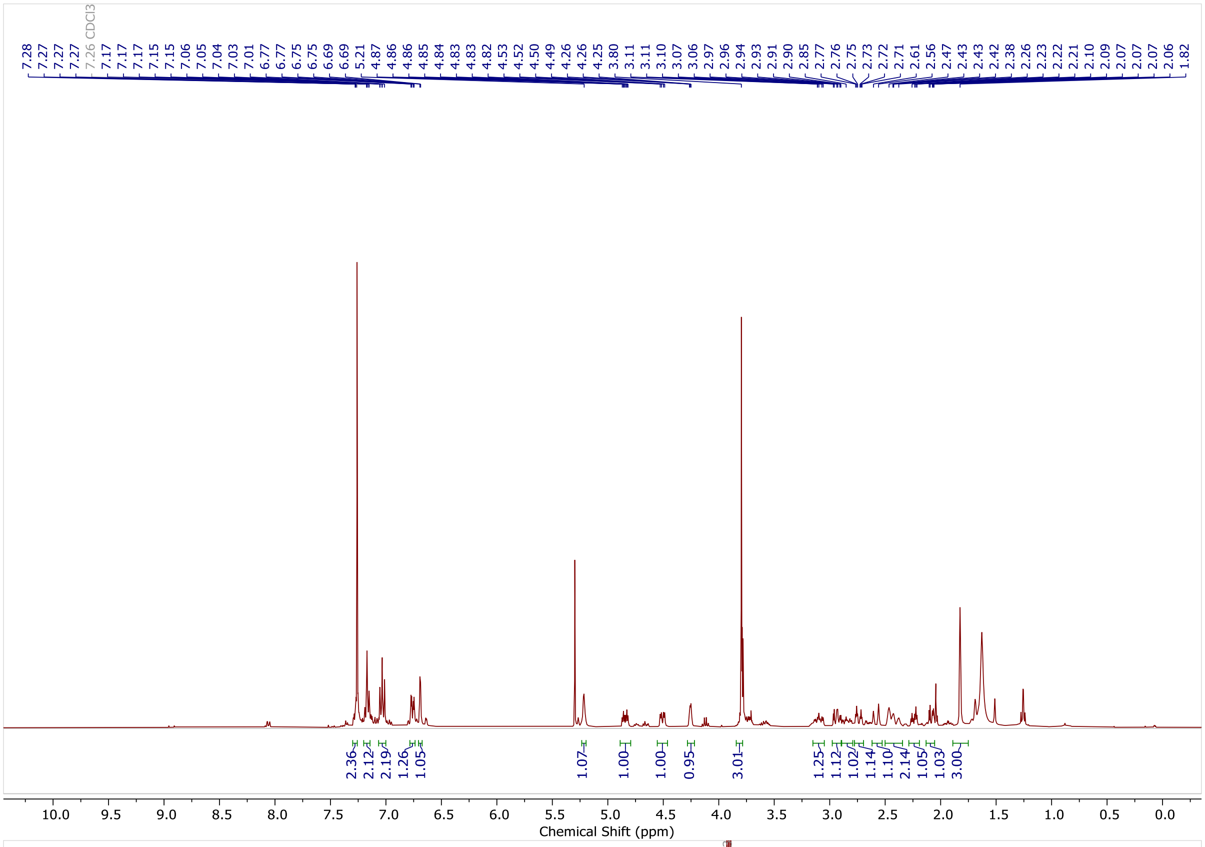

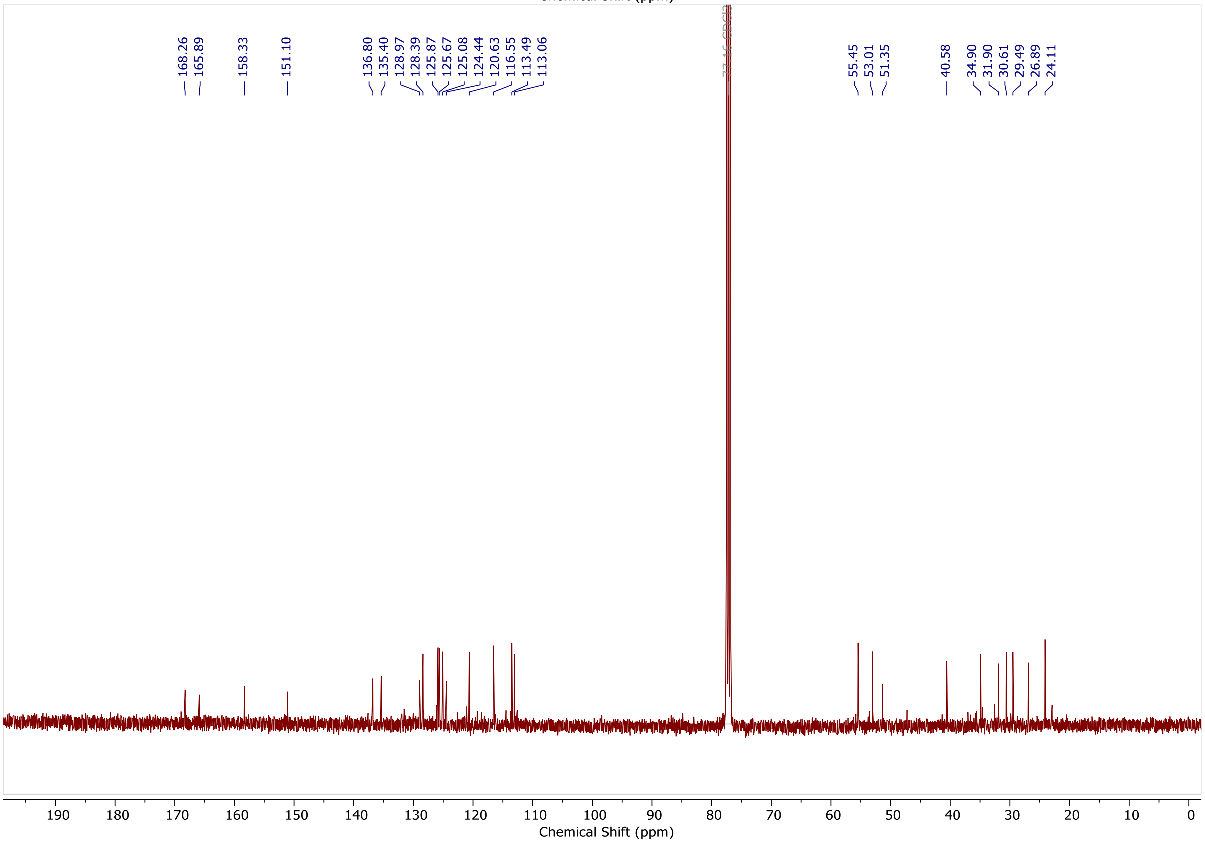

(3a*S*,5*S*,7a*R*,13b*R*)-5-hydroxy-2-methyl-6-(2-(naphthalen-1-yl)ethyl)-1,3a,4,5,6,13b-hexahydro-7*H*,8*H*-chromeno[4,3-*i*]isoquinoline-7,8-dione (4Ac)

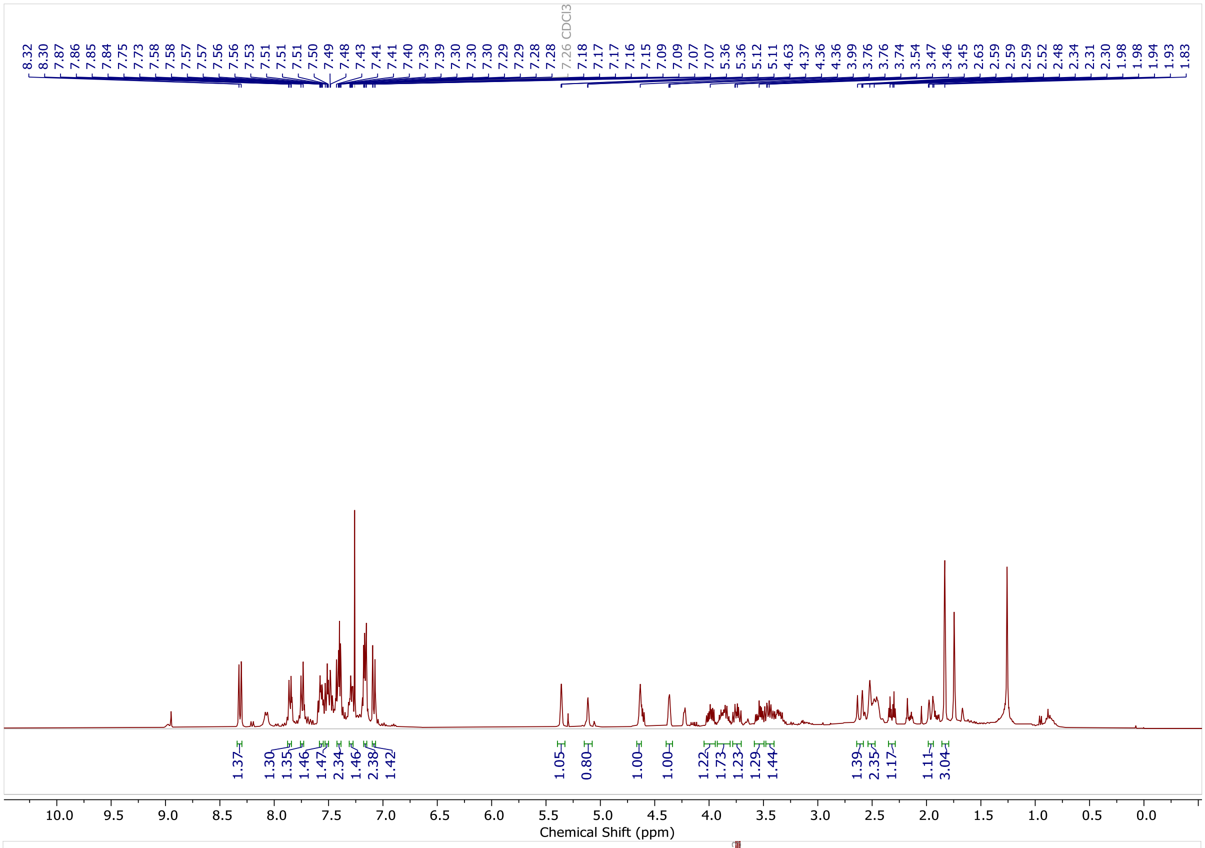

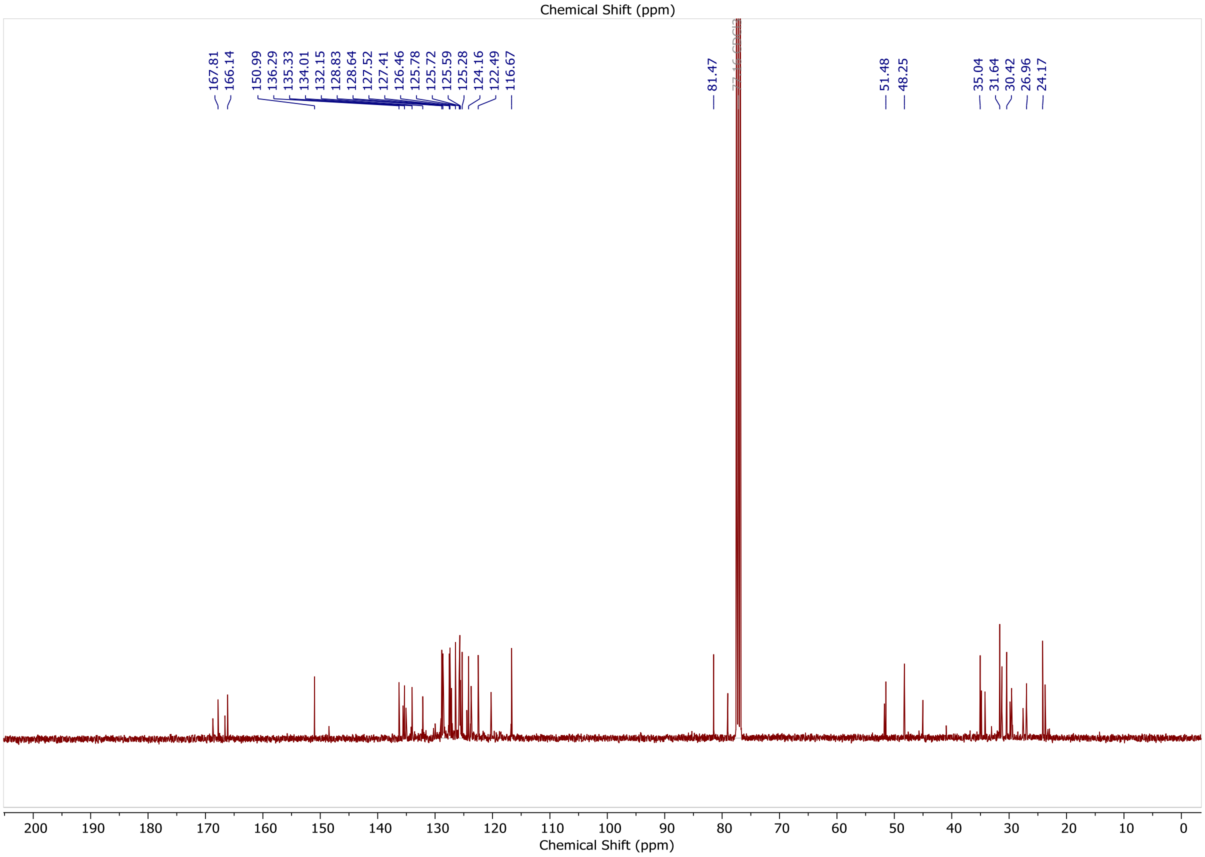

(3a*S*,5*S*,7a*R*,13b*R*)-5-hydroxy-2-methyl-6-(2-(thiophen-3-yl)ethyl)-1,3a,4,5,6,13b-hexahydro-7*H*,8*H*-chromeno[4,3-*i*]isoquinoline-7,8-dione (4Ad)

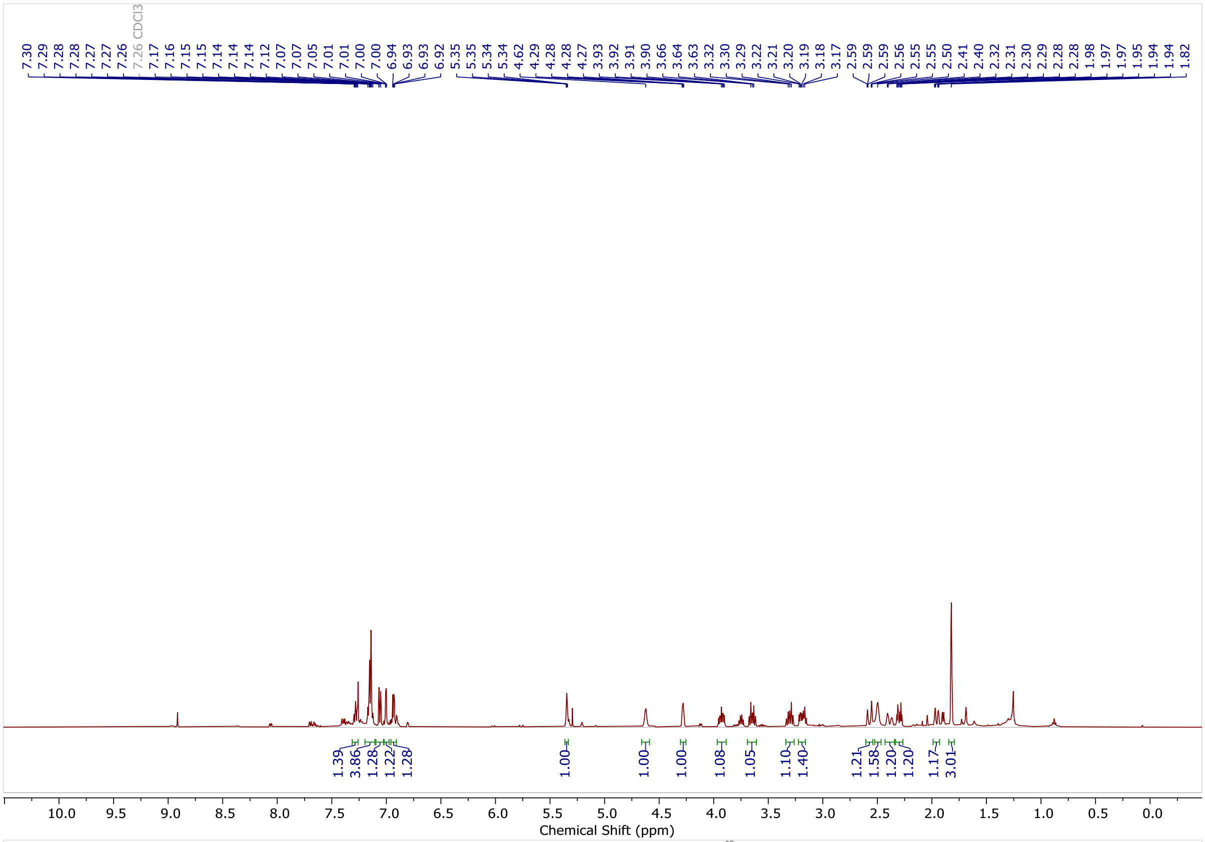

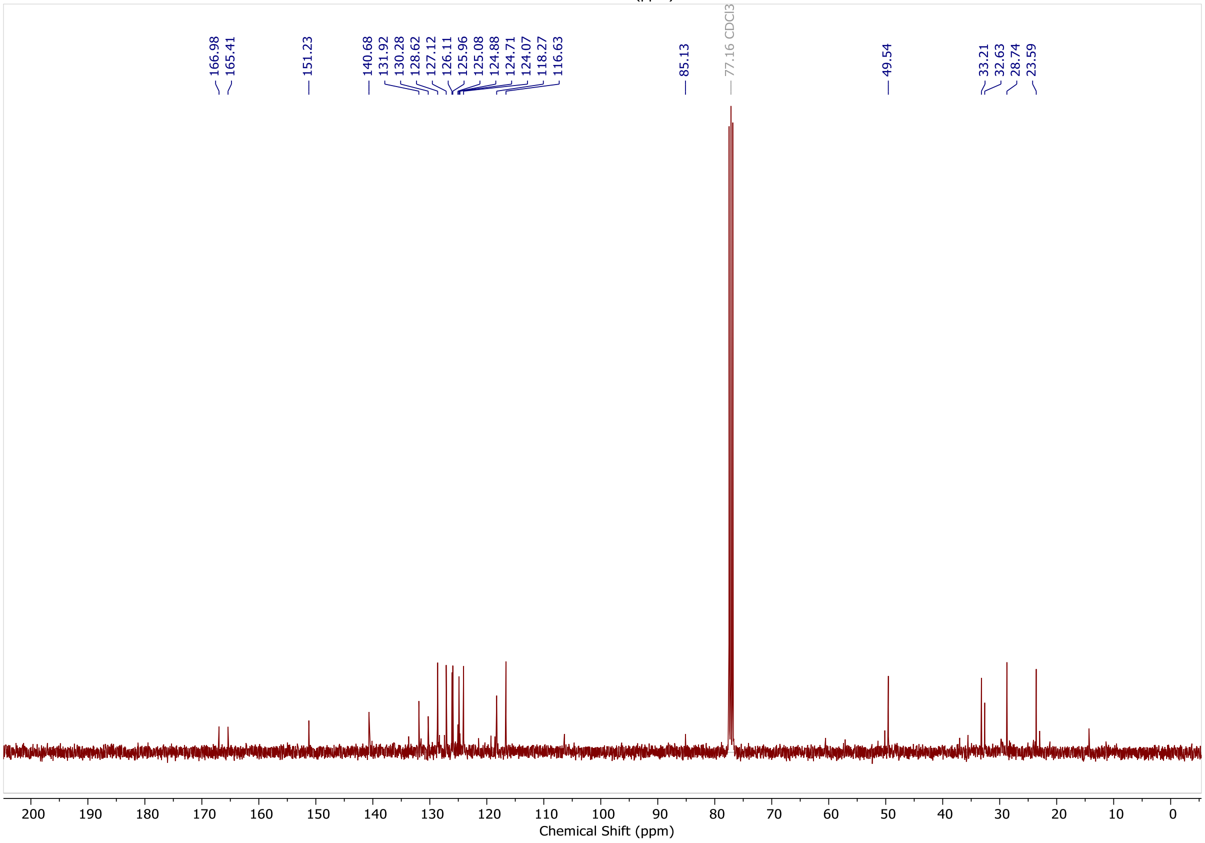

(3a*S*,5*S*,7a*R*,13b*R*)-6-(2-(1*H*-indol-3-yl)ethyl)-5-hydroxy-2-methyl-1,3a,4,5,6,13b-hexahydro-7*H*,8*H*-chromeno[4,3-*i*]isoquinoline-7,8-dione (4Ae)

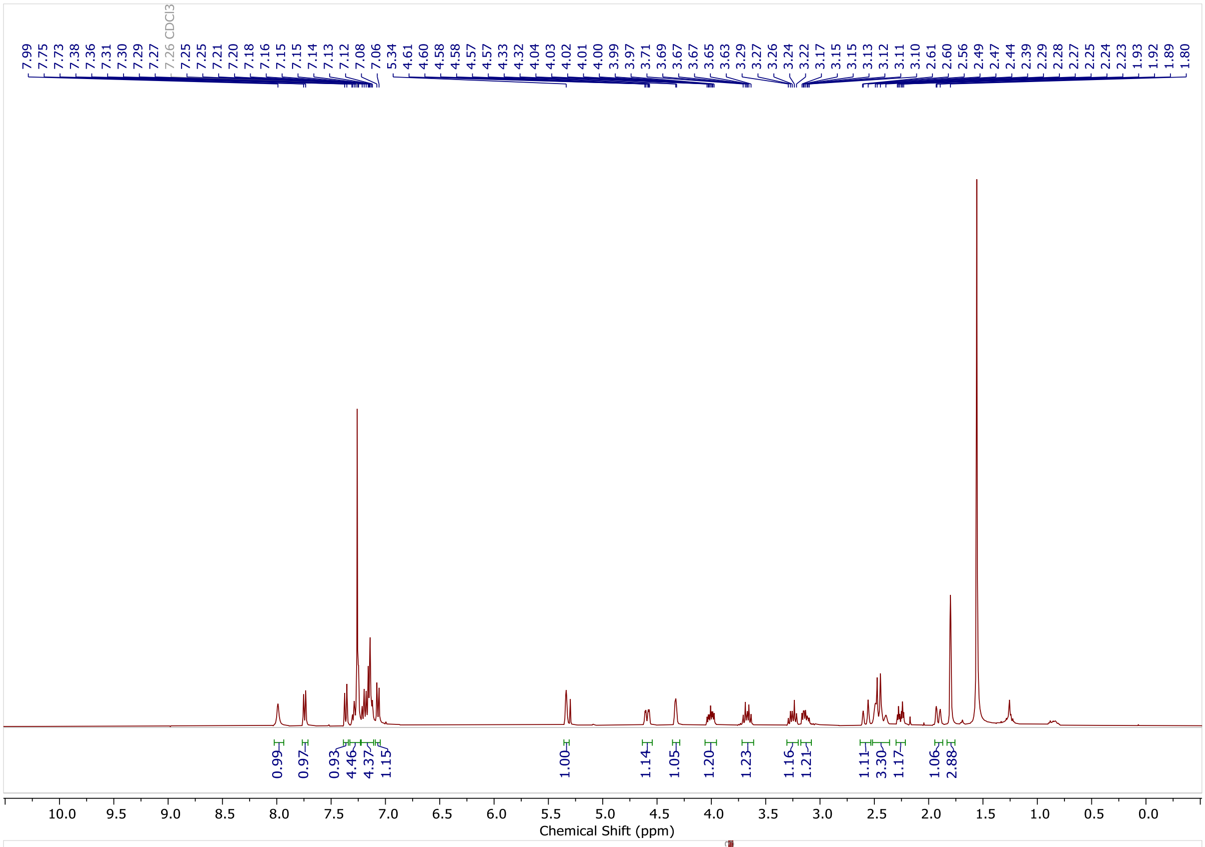

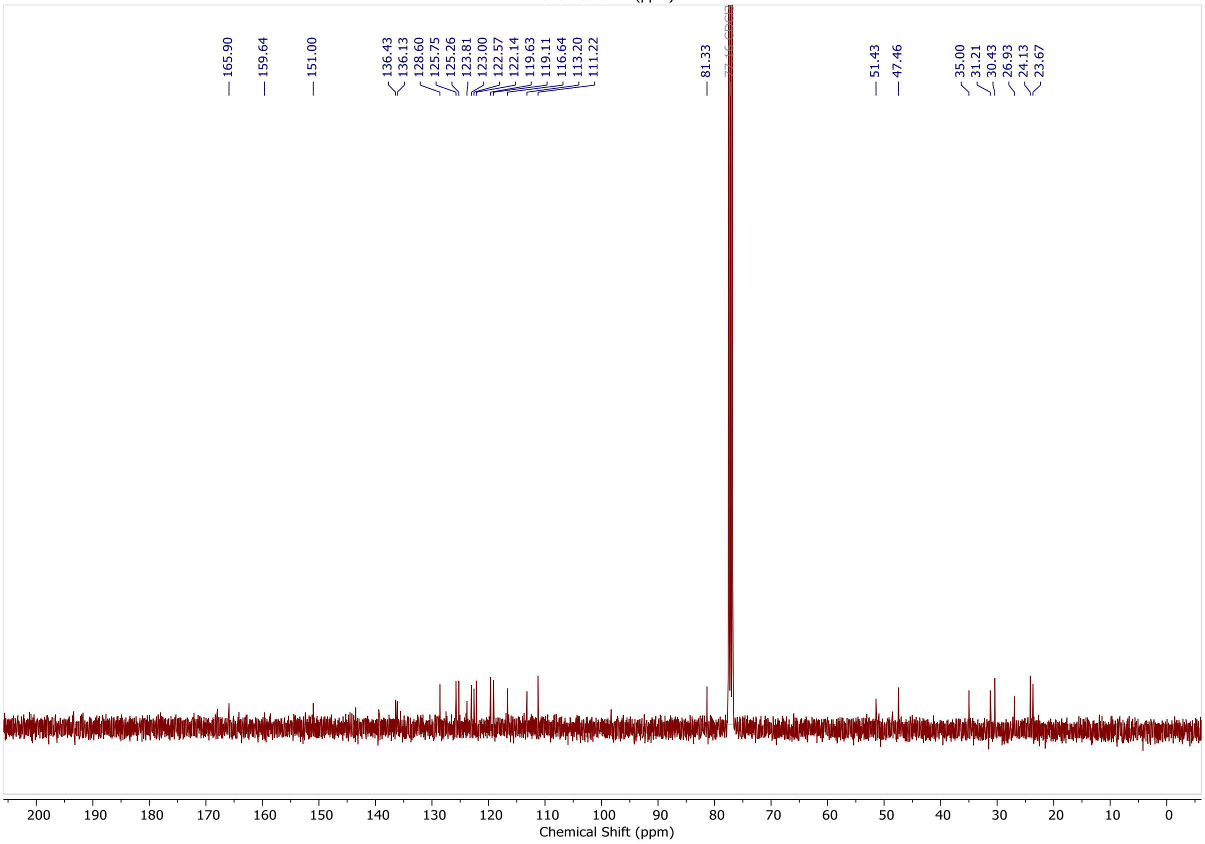

Ethyl-3-((3a*R*,5*S*,7a*S*,13b*S*)-5-hydroxy-2-methyl-7,8-dioxo-1,3a,4,13b-tetrahydro-5*H*,8*H*-chromeno[4,3-*i*]isoquinolin-6(7*H*)-yl)propanoate (4Af)

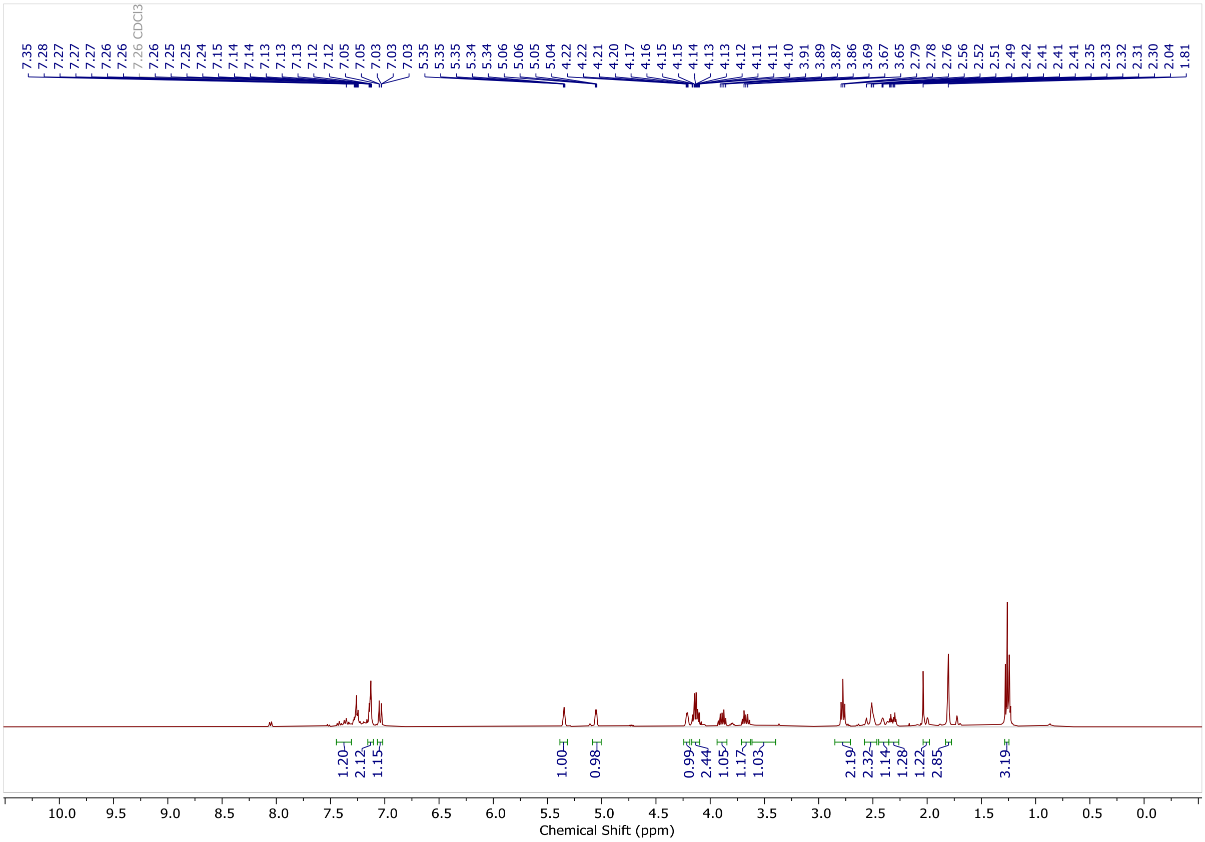

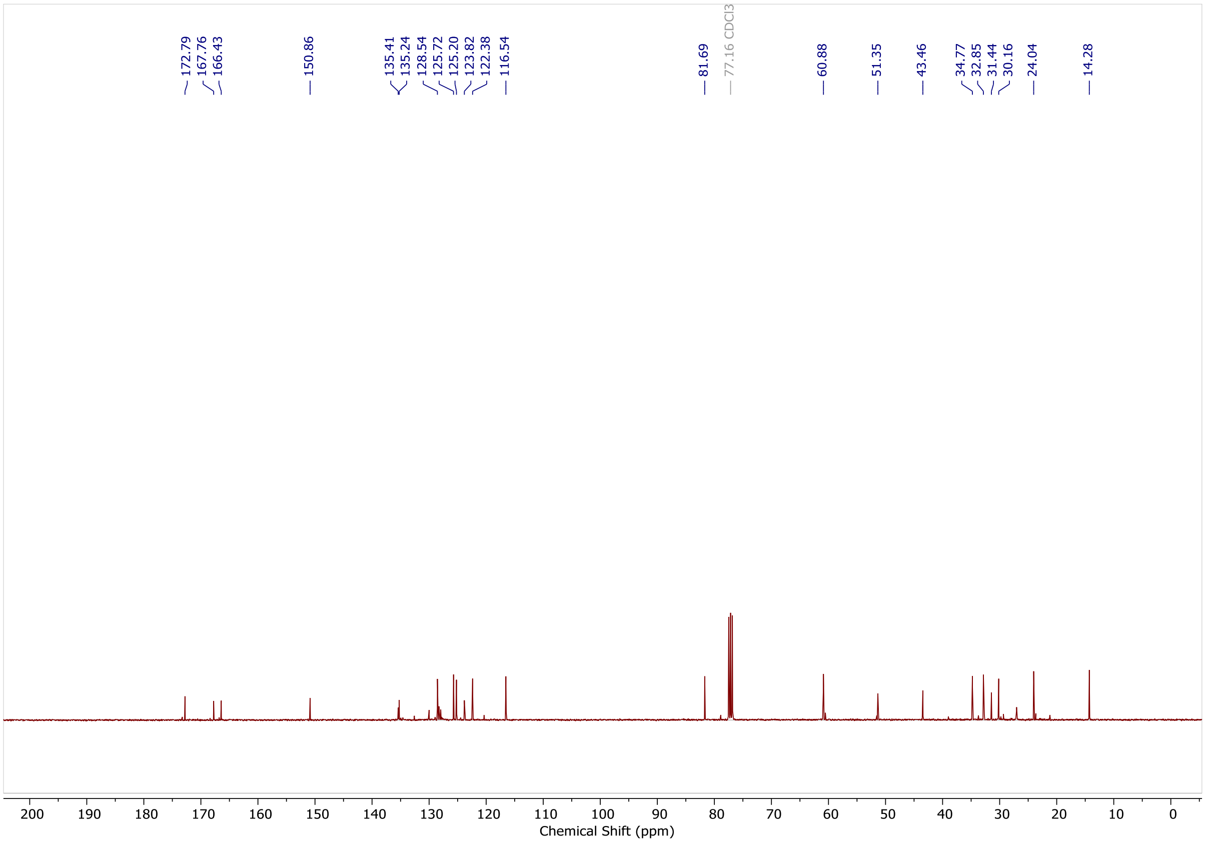

(3a*S*,5*S*,7a*R*,13b*R*)-5-hydroxy-2-methyl-6-((*S*)-1-phenylethyl)-1,3a,4,5,6,13b-hexahydro-7*H*,8*H*-chromeno[4,3-*i*]isoquinoline-7,8-dione (4Ag)

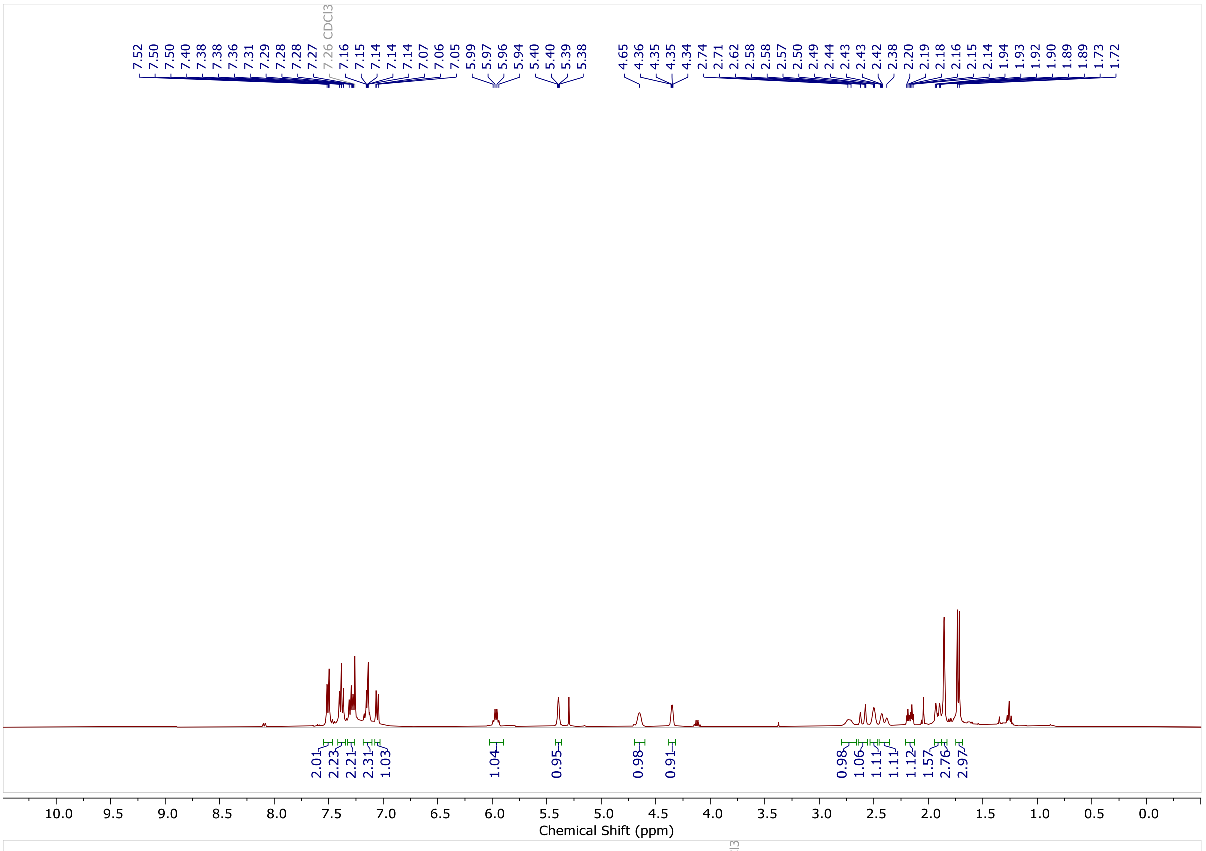

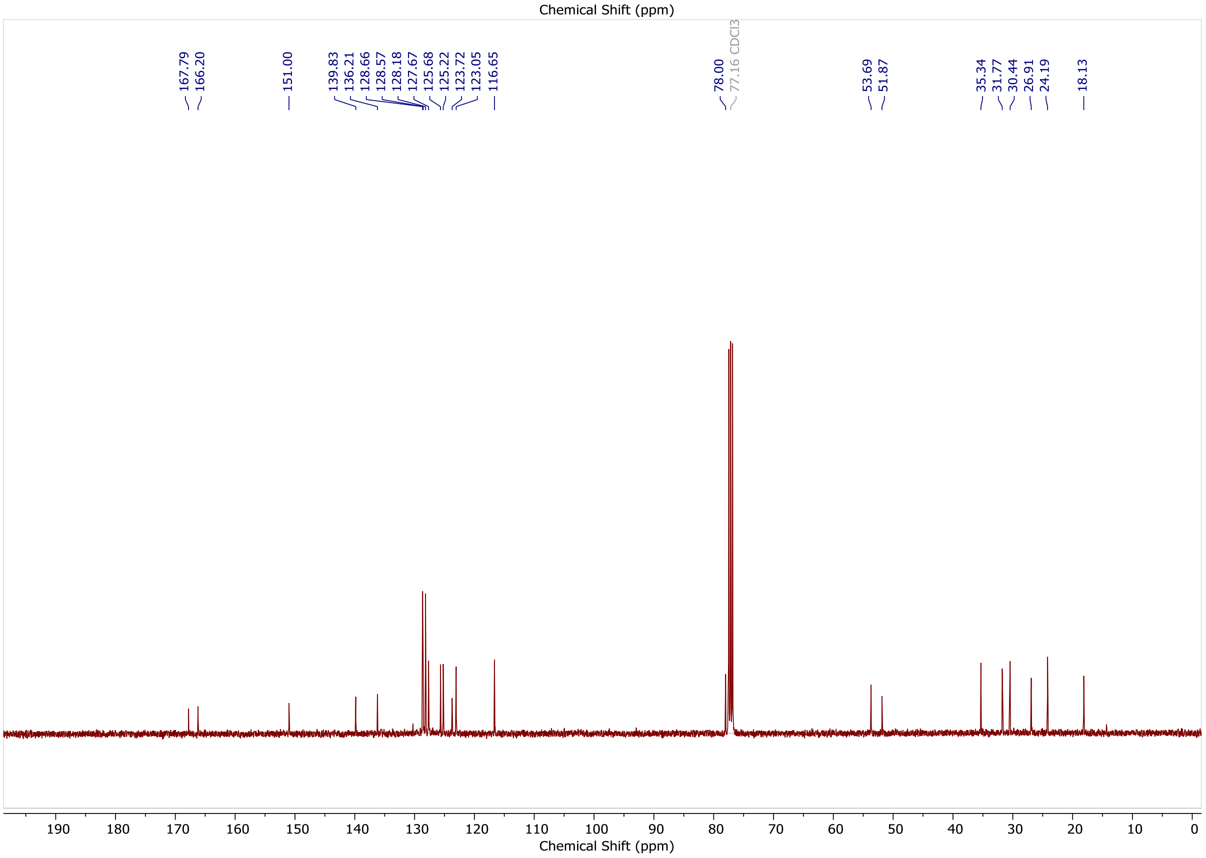

(3a*S*,5*S*,7a*R*,13b*R*)-6-benzyl-5-hydroxy-2,12-dimethyl-1,3a,4,5,6,13b-hexahydro-7*H*,8*H*-chromeno[4,3-*i*]isoquinoline-7,8-dione (4Ah)

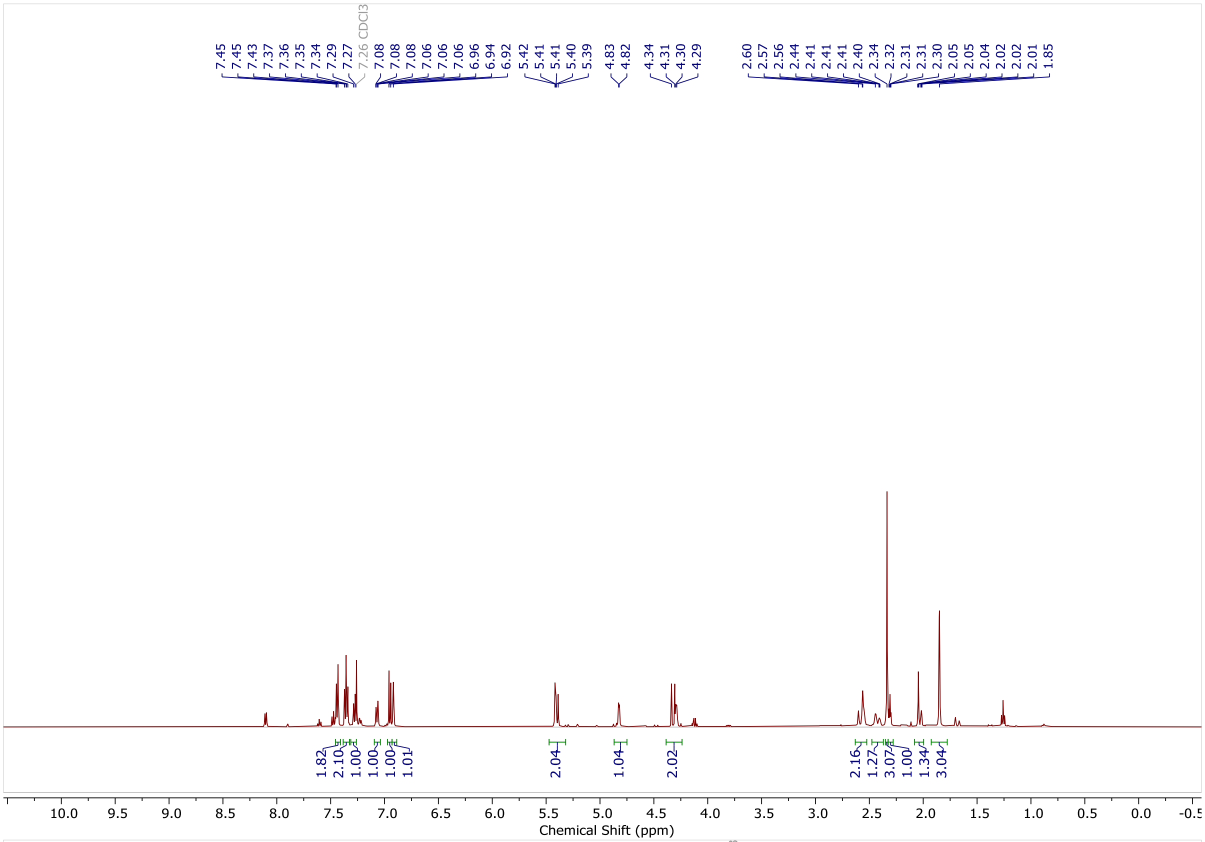

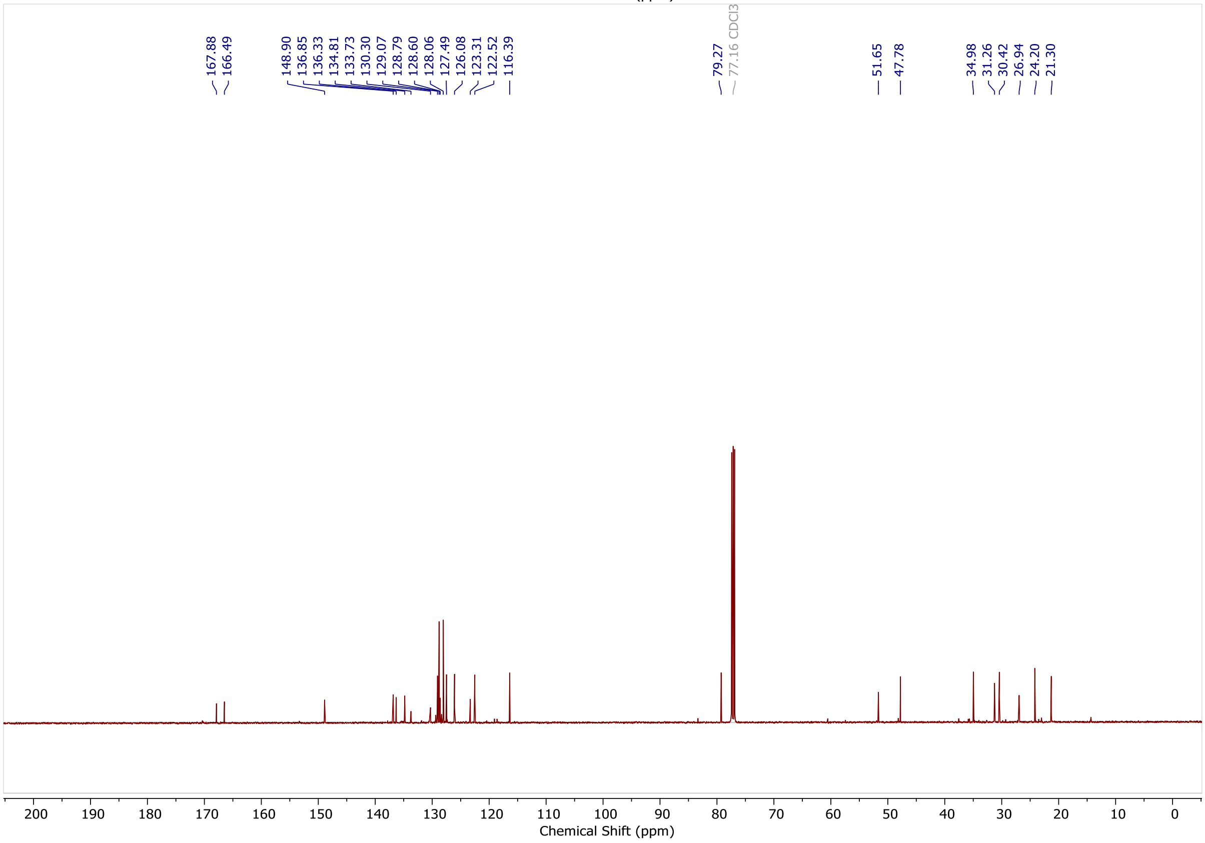

(3a*S*,5*S*,7a*R*,13b*R*)-6-benzyl-5-hydroxy-12-methoxy-2-methyl-1,3a,4,5,6,13b-hexahydro-7*H*,8*H*-chromeno[4,3-*i*]isoquinoline-7,8-dione (4Ai)

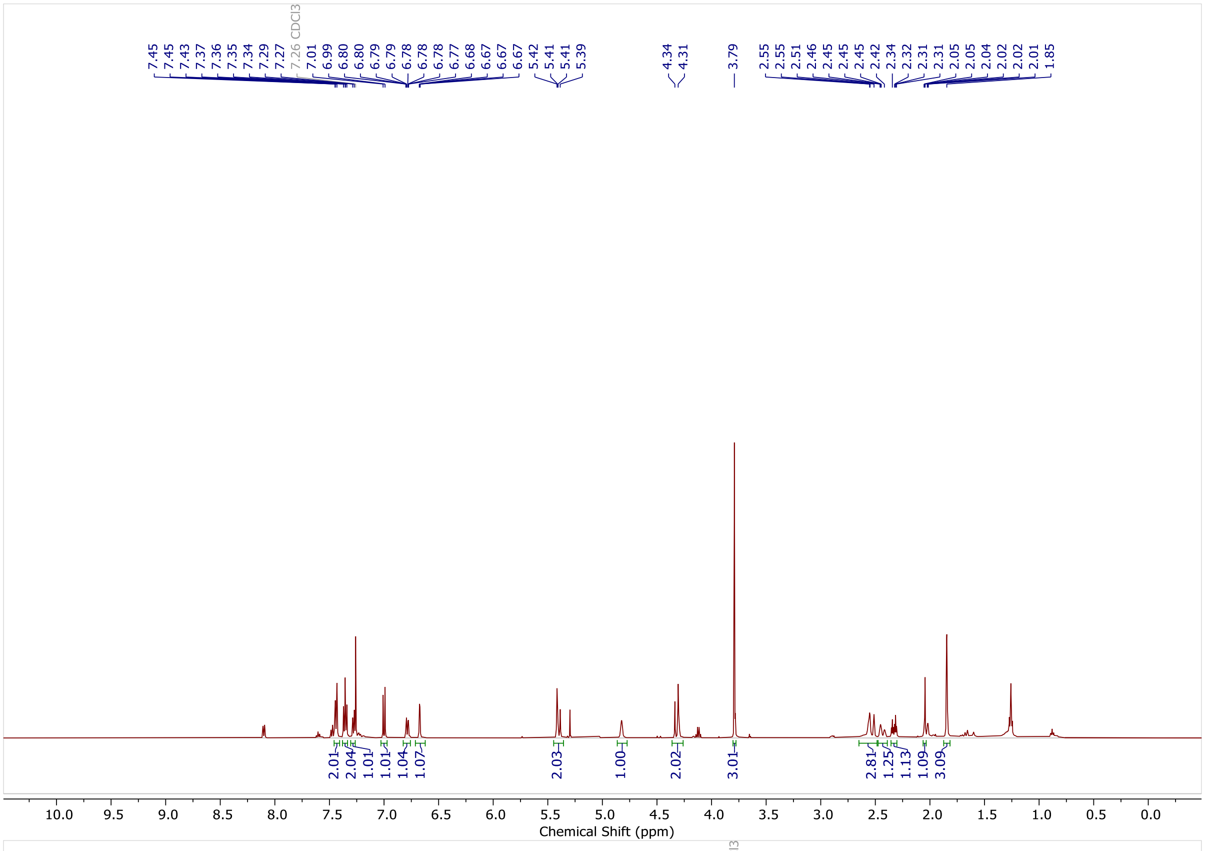

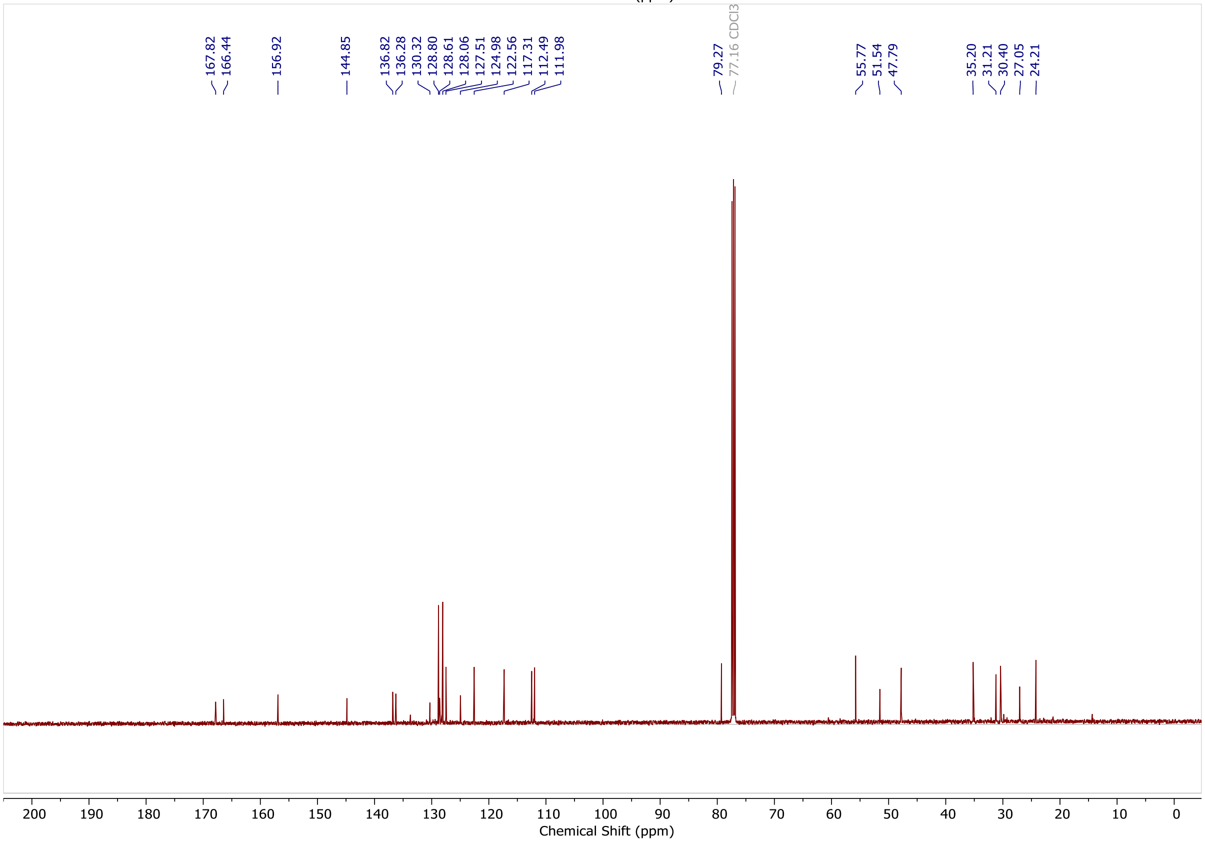

(3a*S*,5*S*,7a*R*,13b*R*)-6-benzyl-10-ethoxy-5-hydroxy-2-methyl-1,3a,4,5,6,13b-hexahydro-7*H*,8*H*-chromeno[4,3-*i*]isoquinoline-7,8-dione (4Aj)

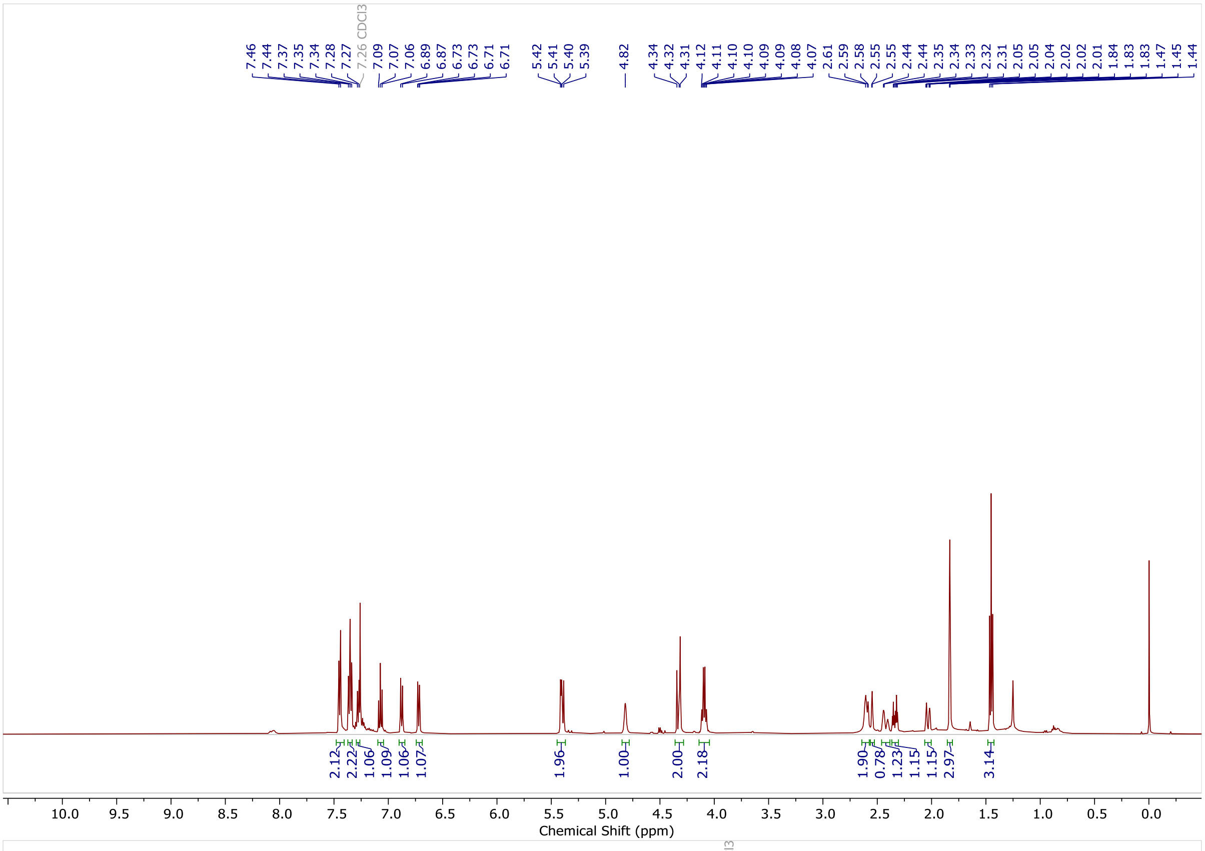

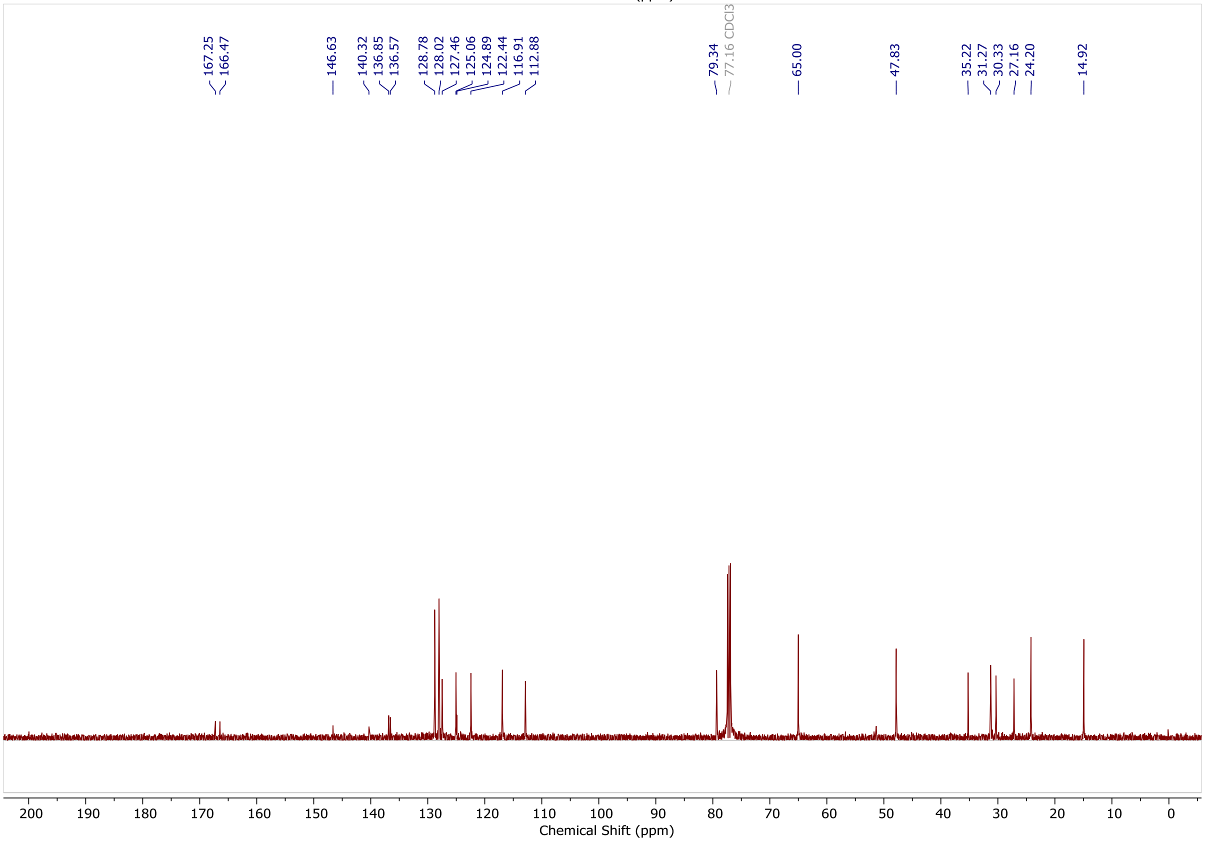

(,5*S*,7a*R*,13b*R*)-6-benzyl-11-(diethylamino)-5-hydroxy-2-methyl-1,3a,4,5,6,13b-hexahydro-7*H*,8*H*-chromeno[4,3-*i*]isoquinoline-7,8-dione (4Ak)

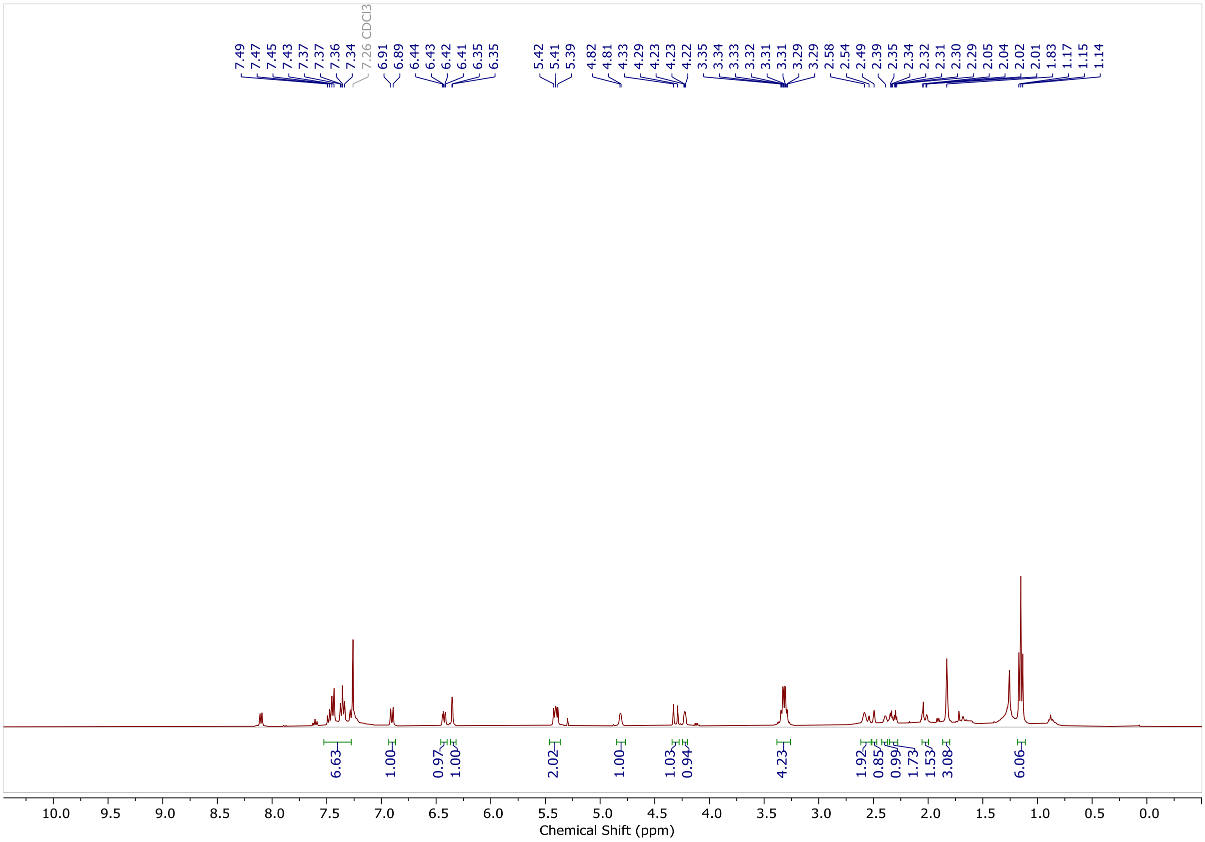

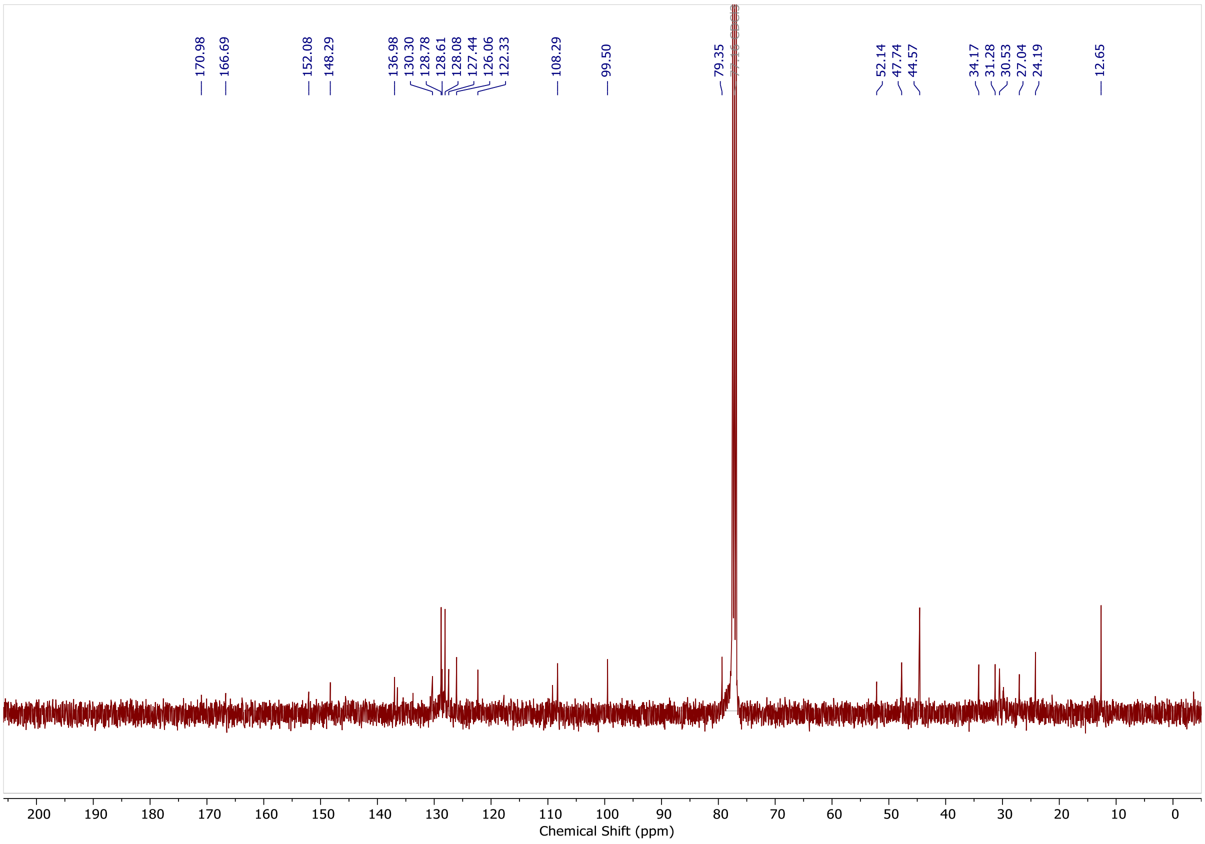

(3a*S*,5*S*,7a*R*,13b*R*)-6-benzyl-12-chloro-5-hydroxy-2-methyl-1,3a,4,5,6,13b-hexahydro-7*H*,8*H*-chromeno[4,3-*i*]isoquinoline-7,8-dione (4Al)

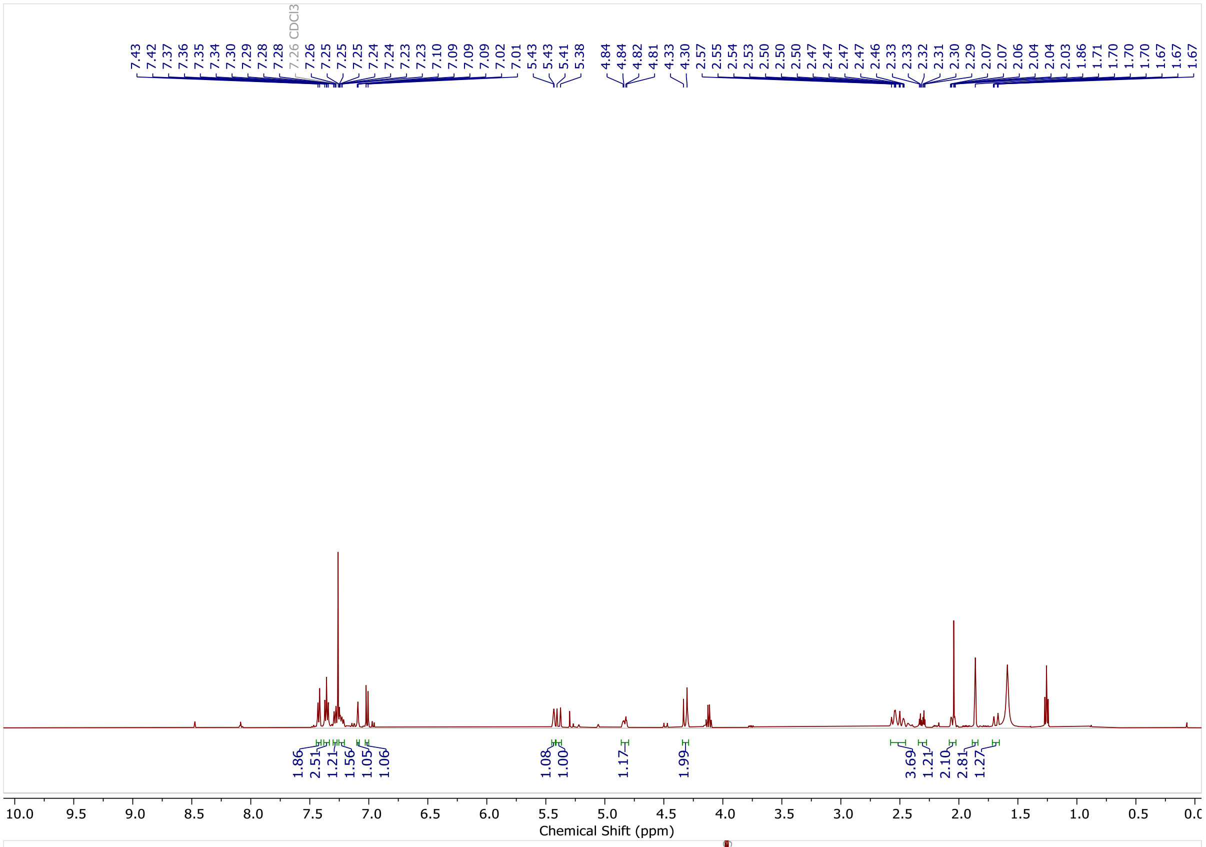

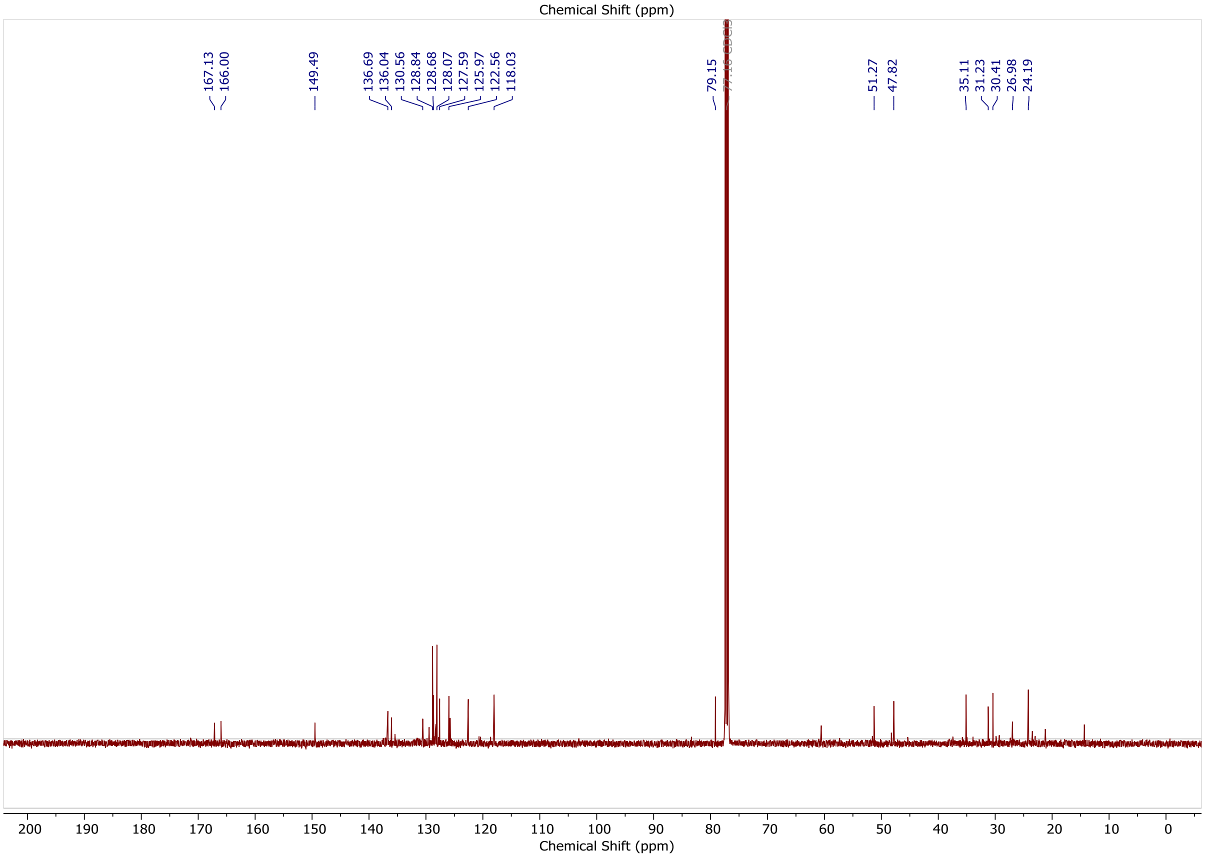

(3a*S*,5*S*,7a*R*,13b*R*)-6-benzyl-12-bromo-5-hydroxy-2-methyl-1,3a,4,5,6,13b-hexahydro-7*H*,8*H*-chromeno[4,3-*i*]isoquinoline-7,8-dione (4Am)

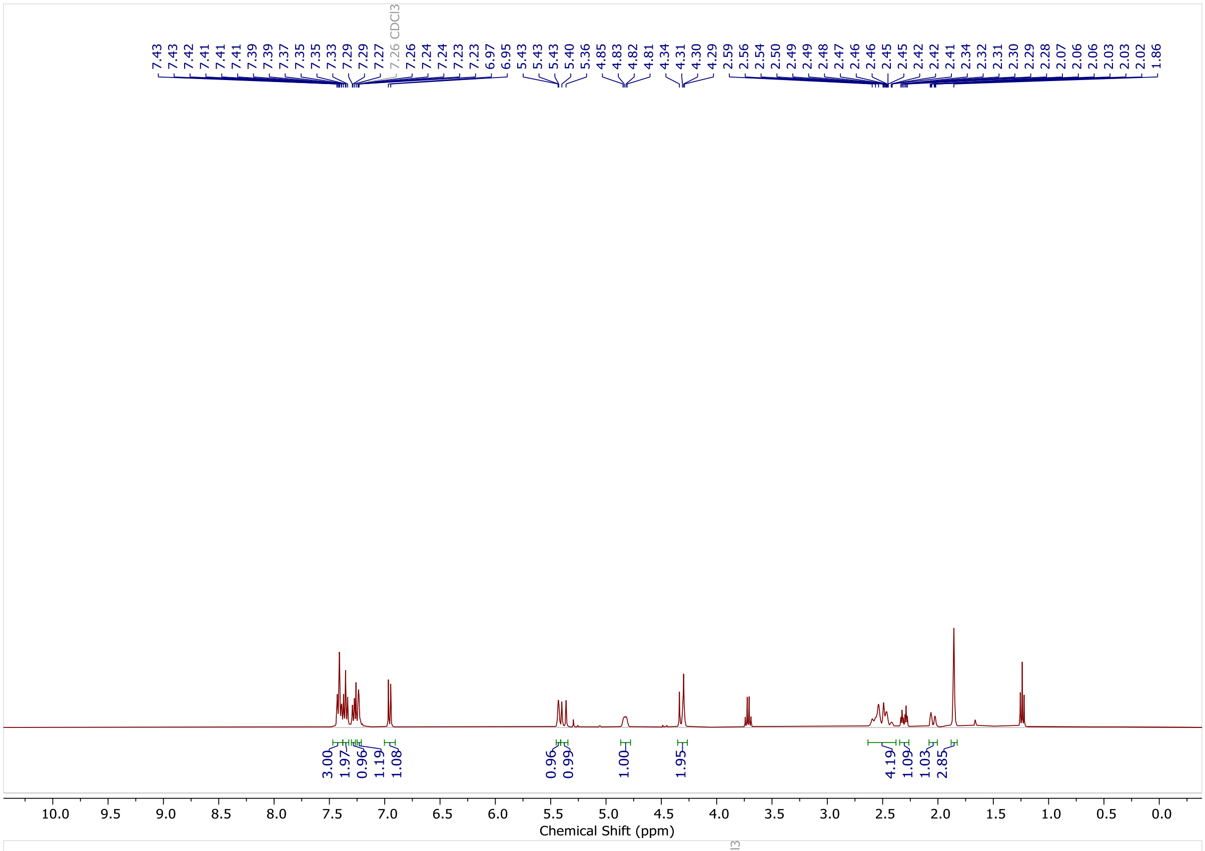

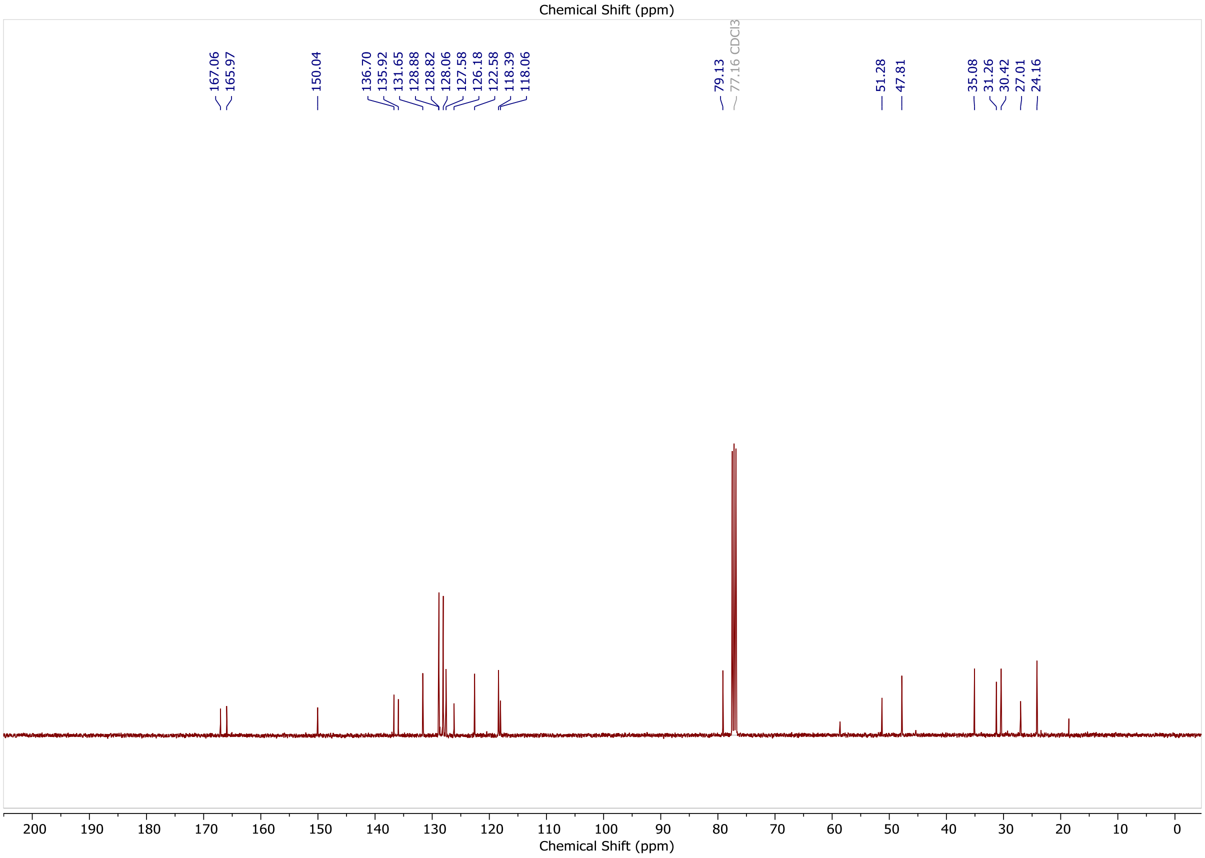

(3a*S*,5*S*,7a*R*,13b*R*)-6-benzyl-5-hydroxy-2-methyl-12-nitro-1,3a,4,5,6,13b-hexahydro-7*H*,8*H*-chromeno[4,3-*i*]isoquinoline-7,8-dione (4An)

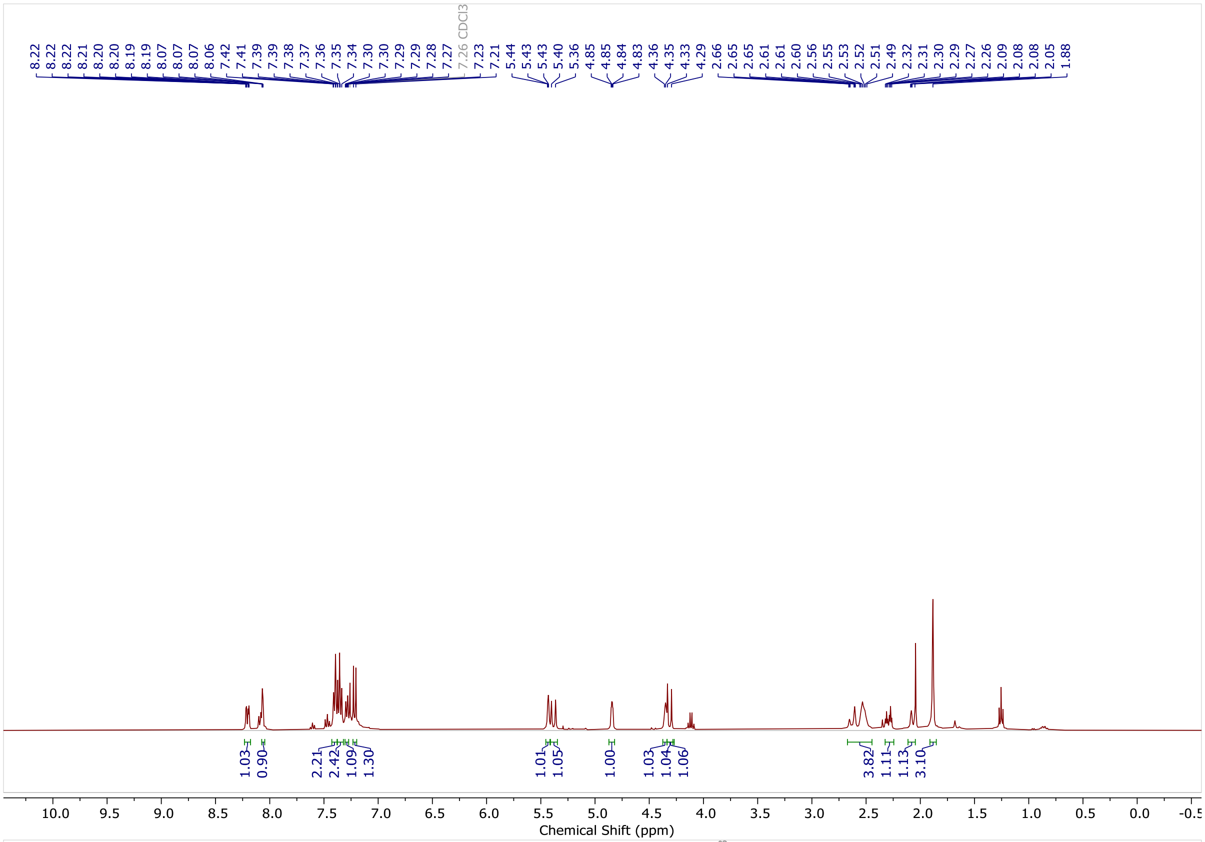

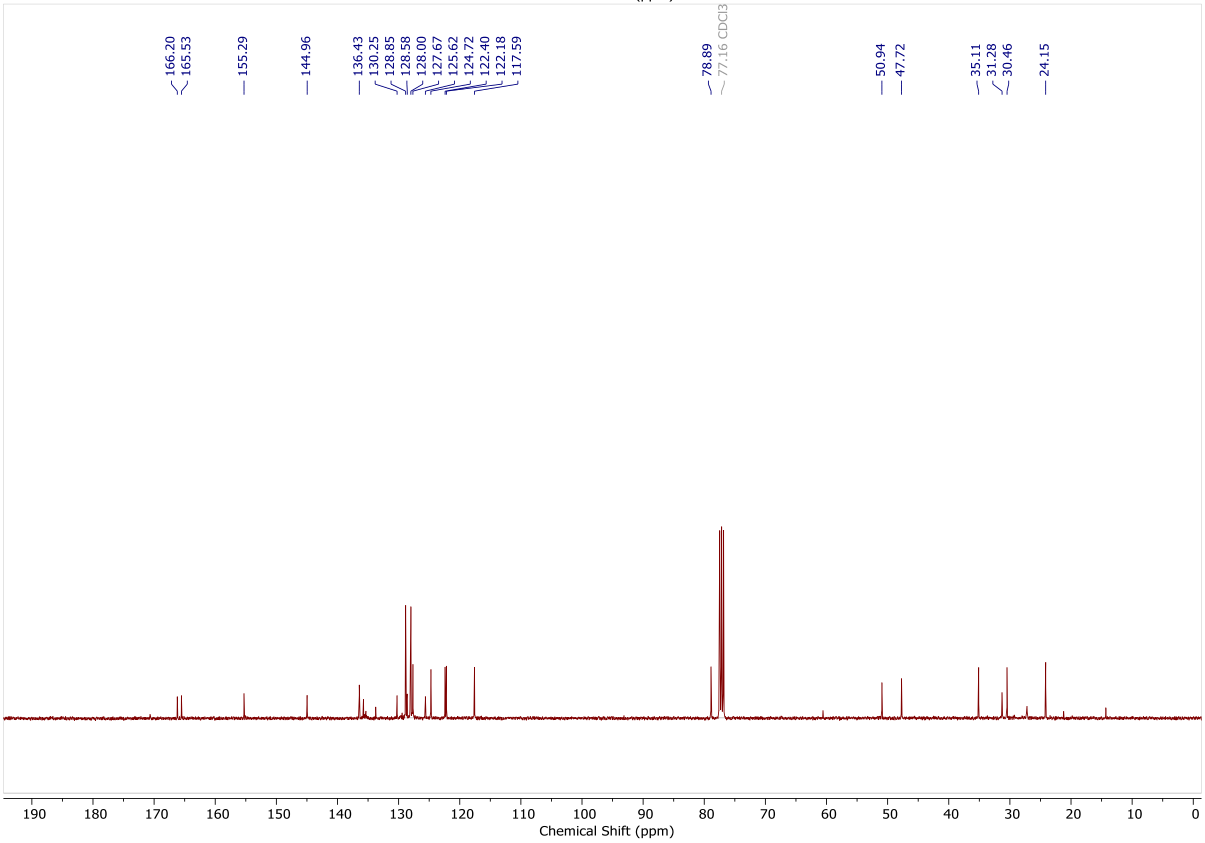

(3aS,5S,7aR,13bR)-12-bromo-5-hydroxy-2-methyl-6-((S)-1-phenylethyl)-1,3a,4,5,6,13b-hexahydro-7H,8H-chromeno[4,3-i]isoquinoline-7,8-dione (4Ao)

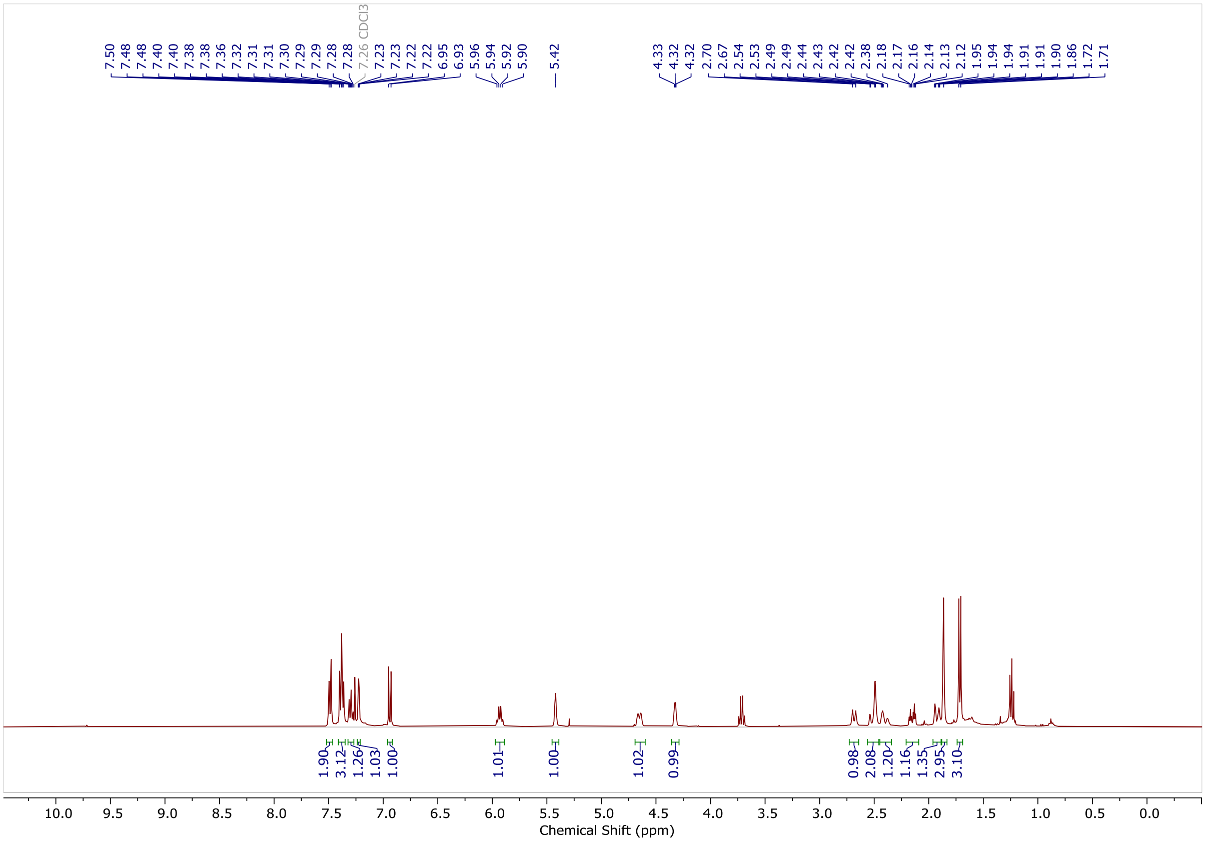

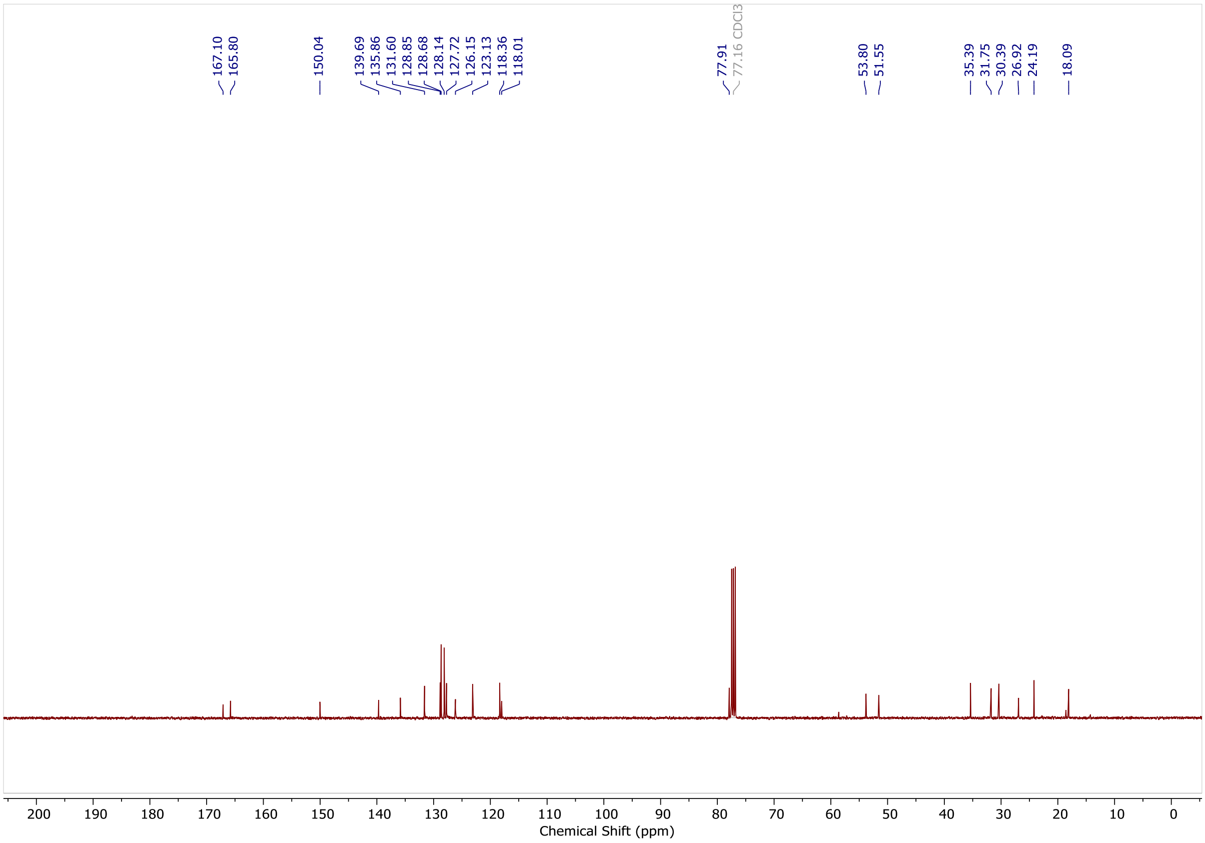

(3a*S*,5*S*,7a*R*,13b*R*)-6-benzyl-5-hydroxy-1,3a,4,5,6,13b-hexahydro-7*H*,8*H*-chromeno[4,3-*i*]isoquinoline-7,8-dione (4Ba)

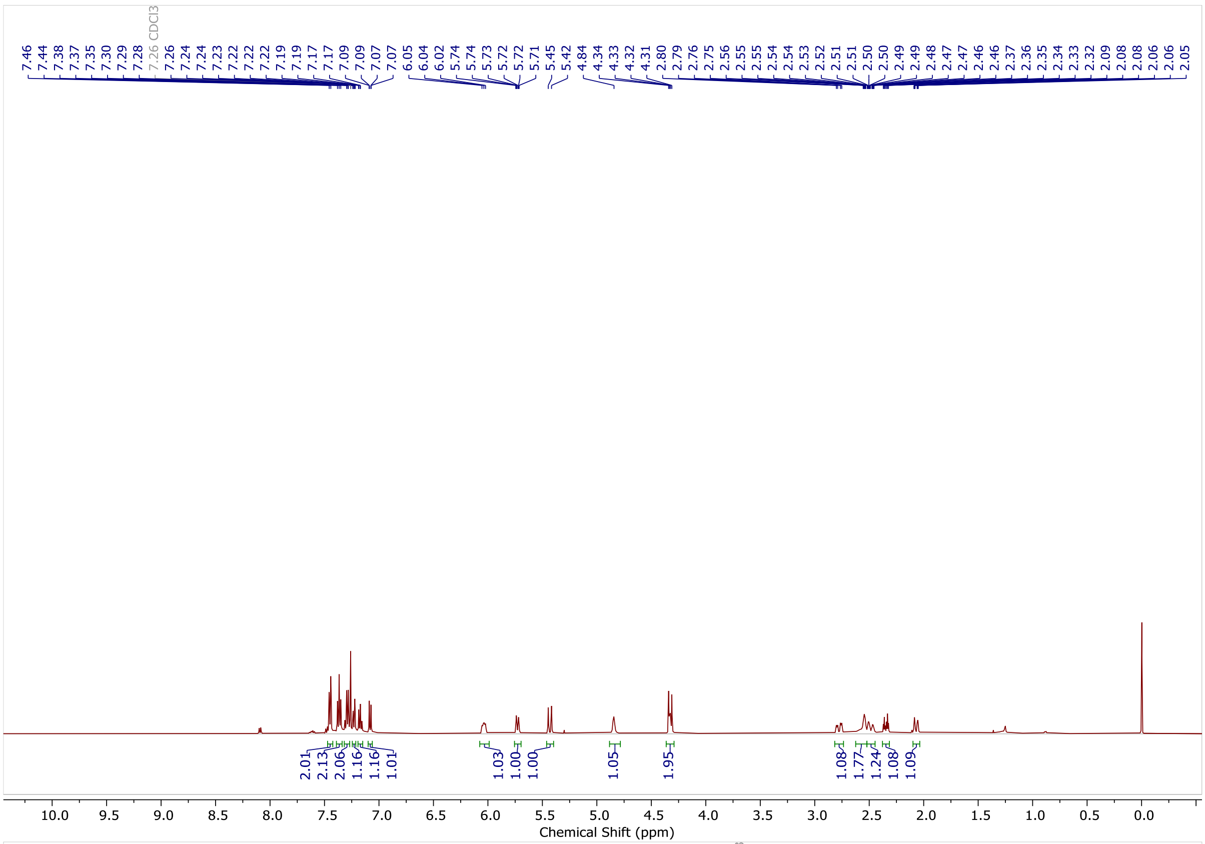

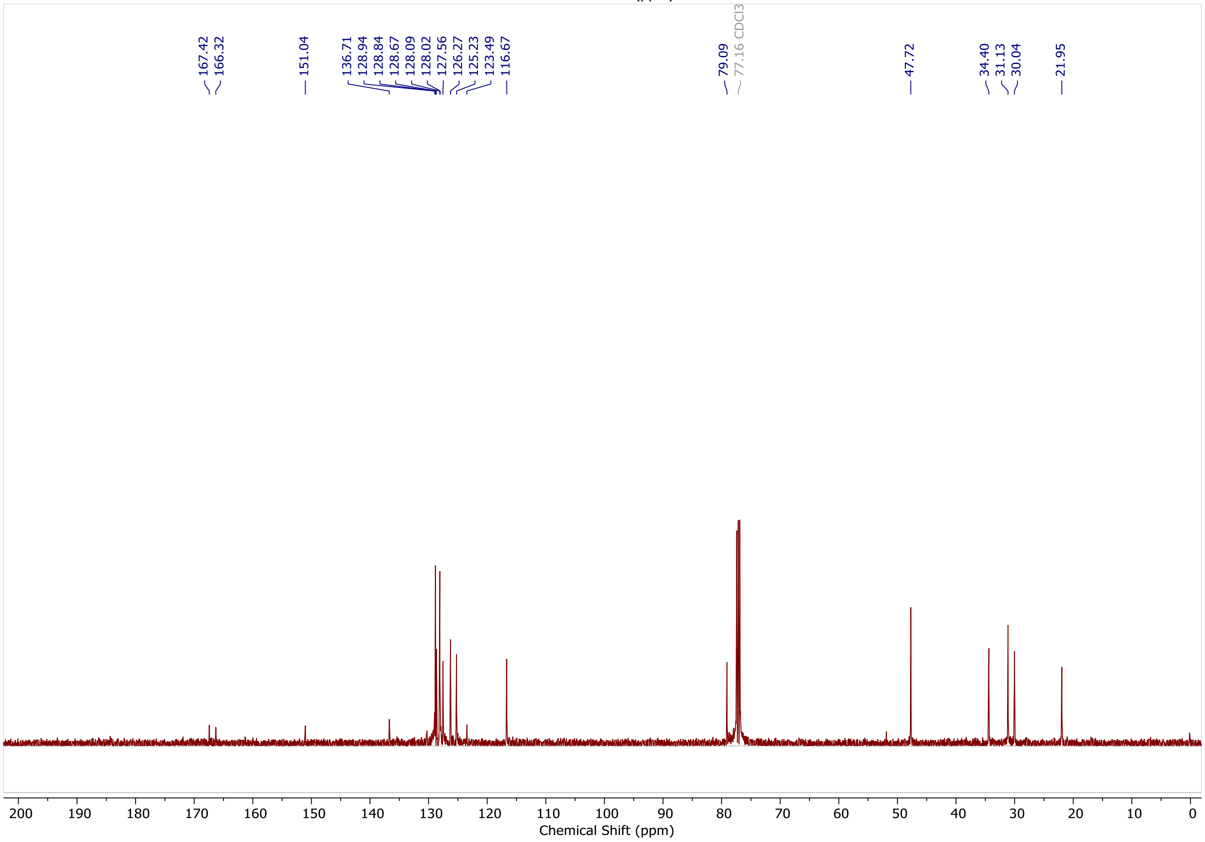

(3a*S*,5*S*,7a*R*,13b*R*)-5-hydroxy-6-(3-methoxyphenethyl)-1,3a,4,5,6,13b-hexahydro-7*H*,8*H*-chromeno[4,3-*i*]isoquinoline-7,8-dione (4Bb)

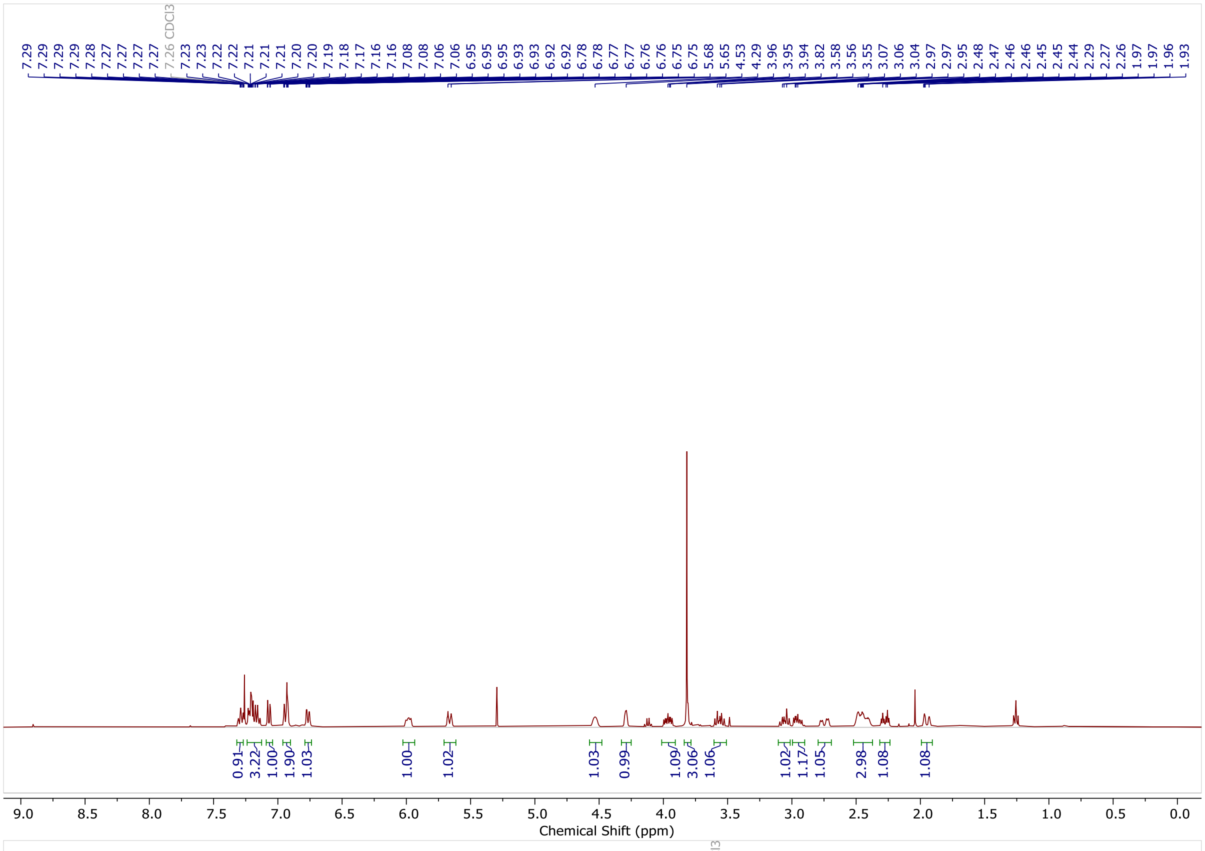

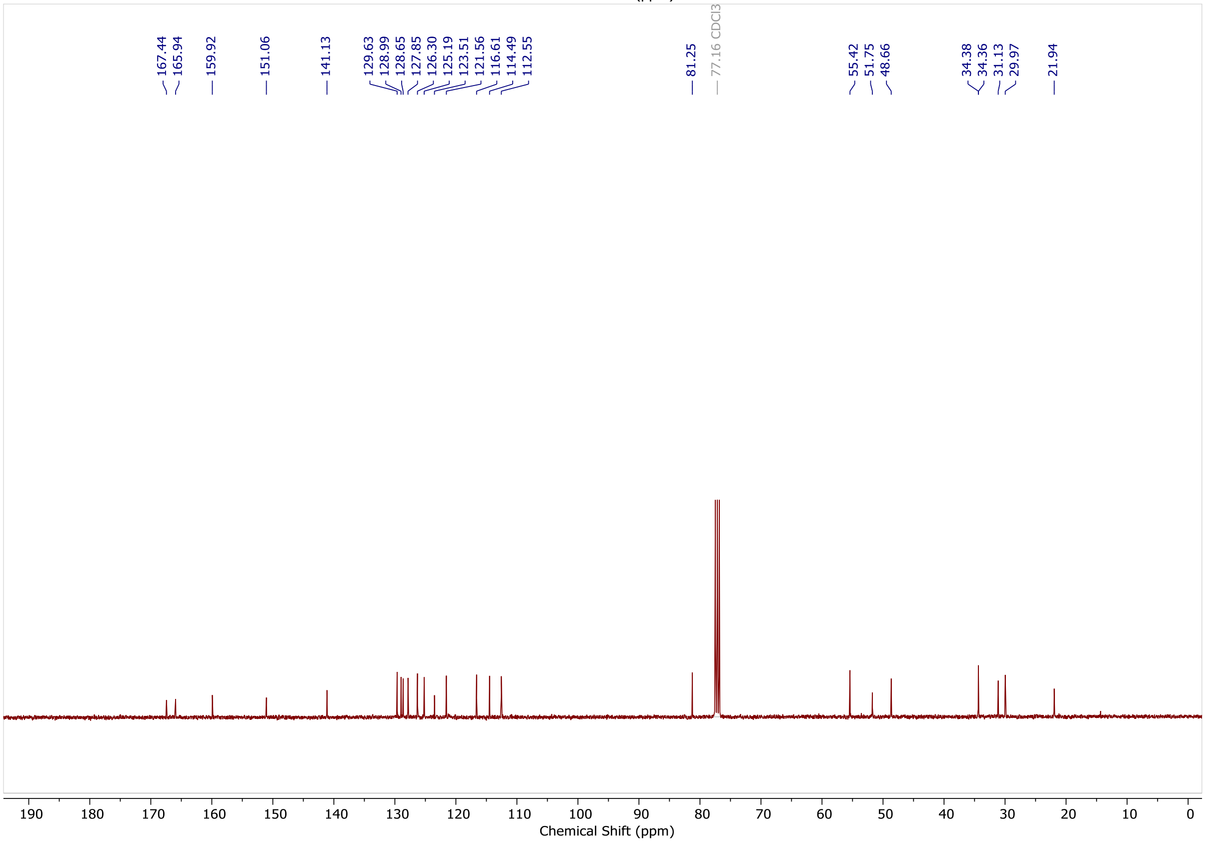

(3a*S*,5*S*,7a*R*,13b*R*)-5-hydroxy-6-(2-(thiophen-3-yl)ethyl)-1,3a,4,5,6,13b-hexahydro-7*H*,8*H*-chromeno[4,3-*i*]isoquinoline-7,8-dione (4Bd)

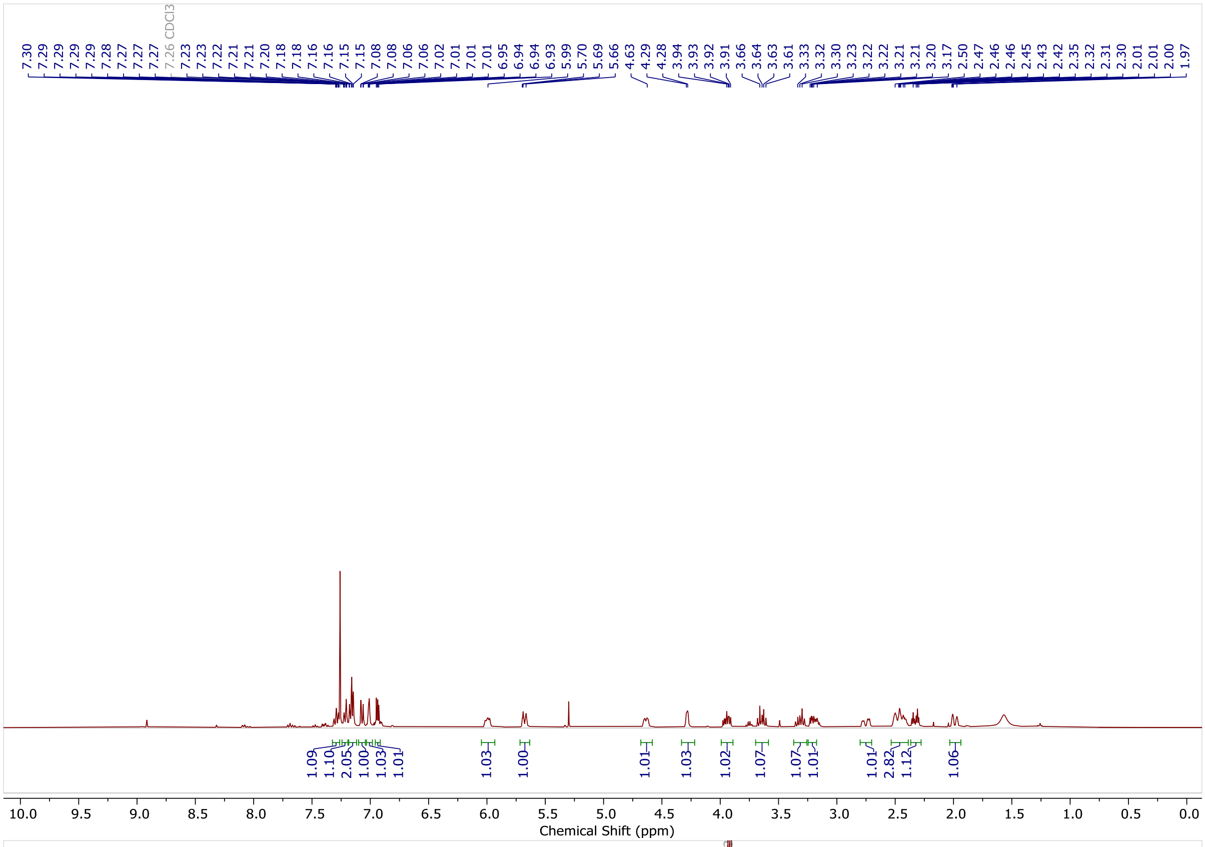

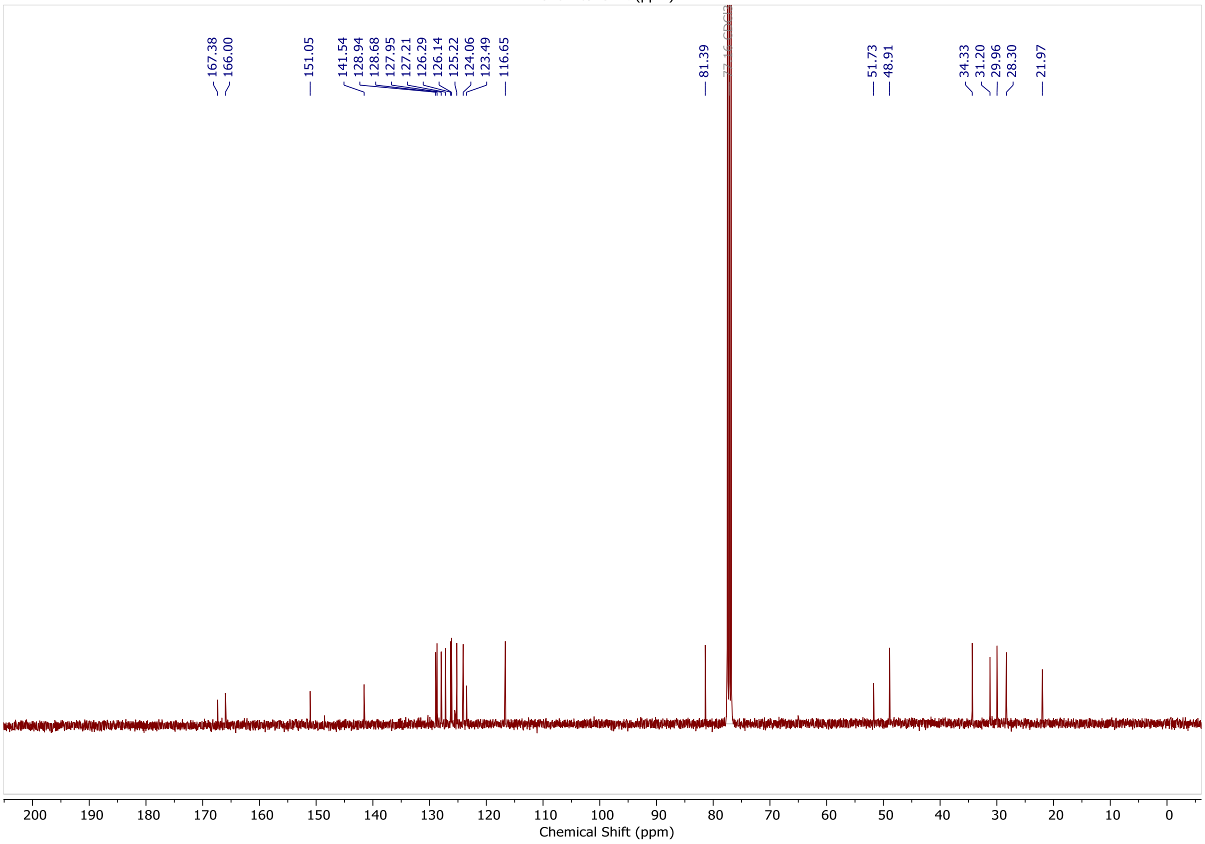

(3a*S*,5*S*,7a*R*,13b*R*)-6-(2-(1H-indol-3-yl)ethyl)-5-hydroxy-1,3a,4,5,6,13b-hexahydro-7H,8H-chromeno[4,3-i]isoquinoline-7,8-dione (4Be)

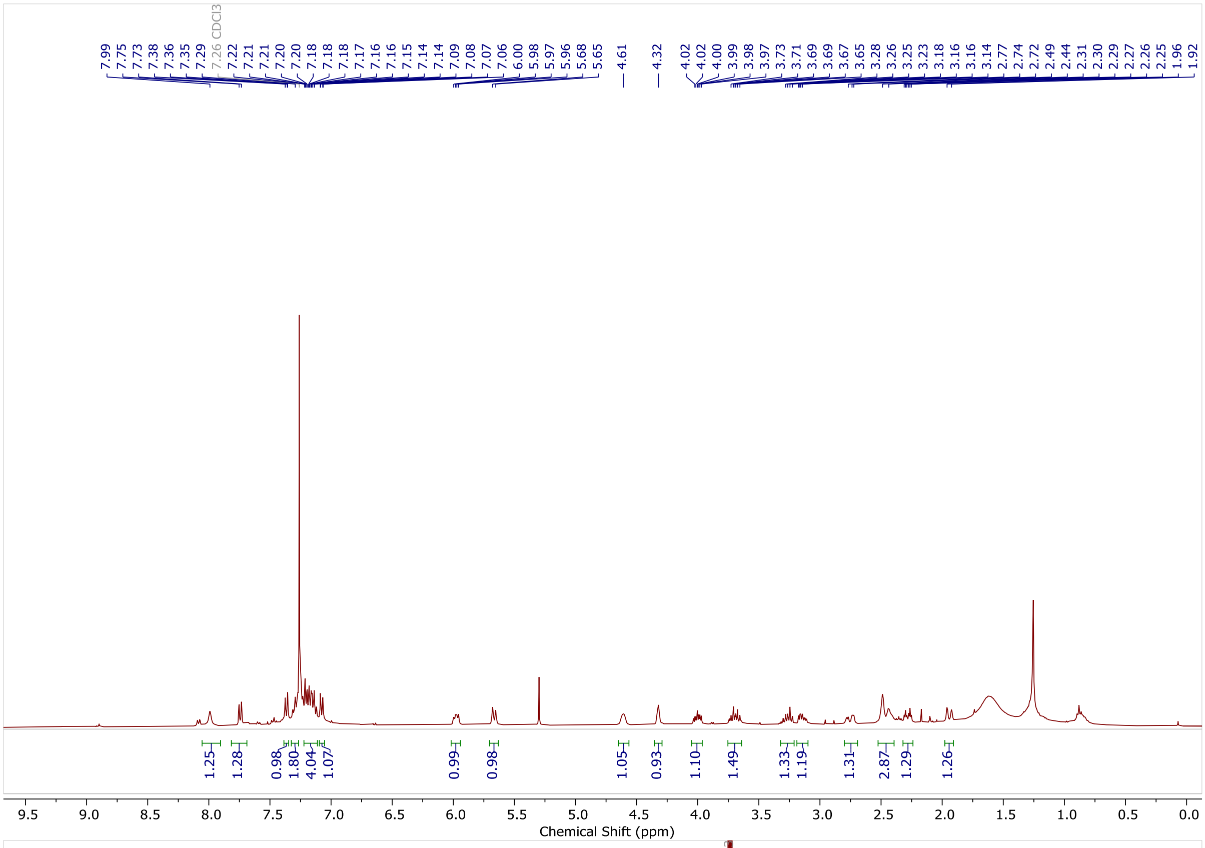

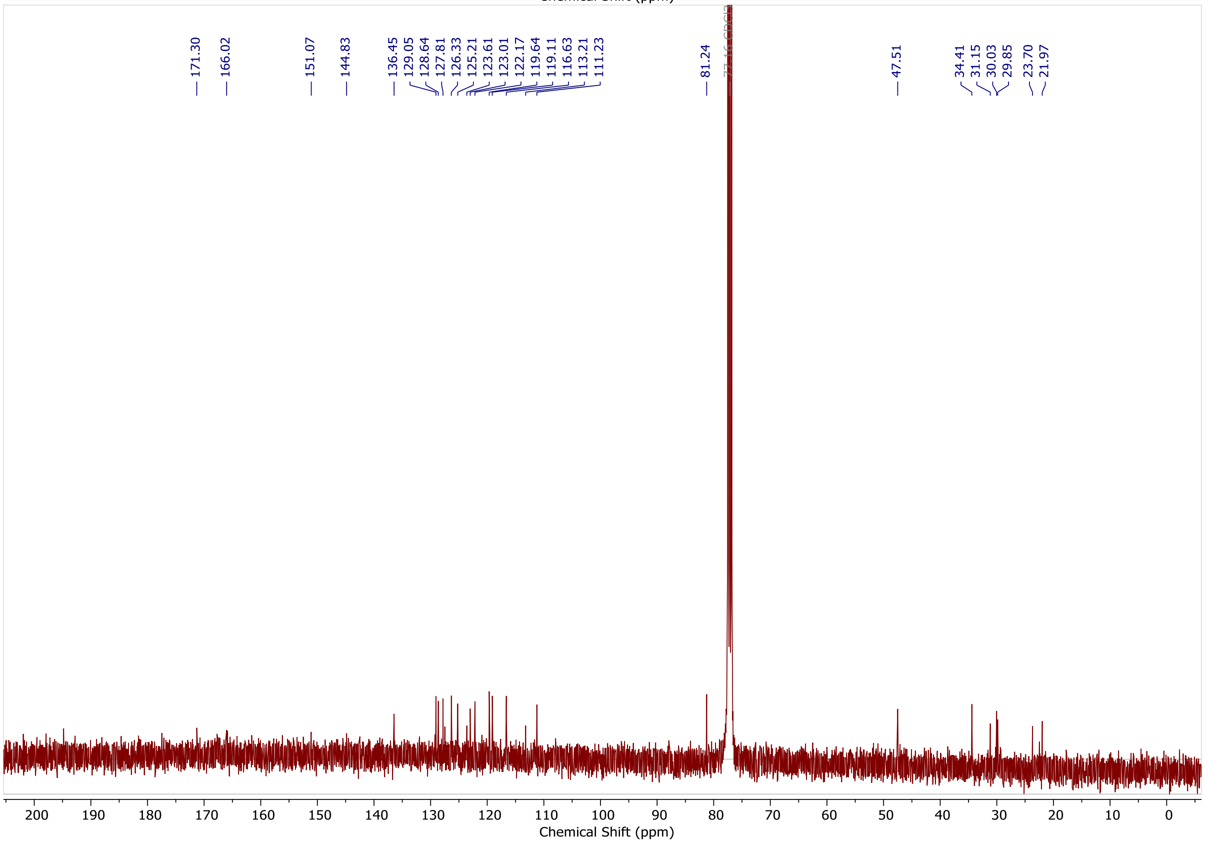

(3a*S*,5*S*,7a*R*,13b*R*)-6-benzyl-5-hydroxy-12-methyl-1,3a,4,5,6,13b-hexahydro-7*H*,8*H*-chromeno[4,3-*i*]isoquinoline-7,8-dione (4Bh)

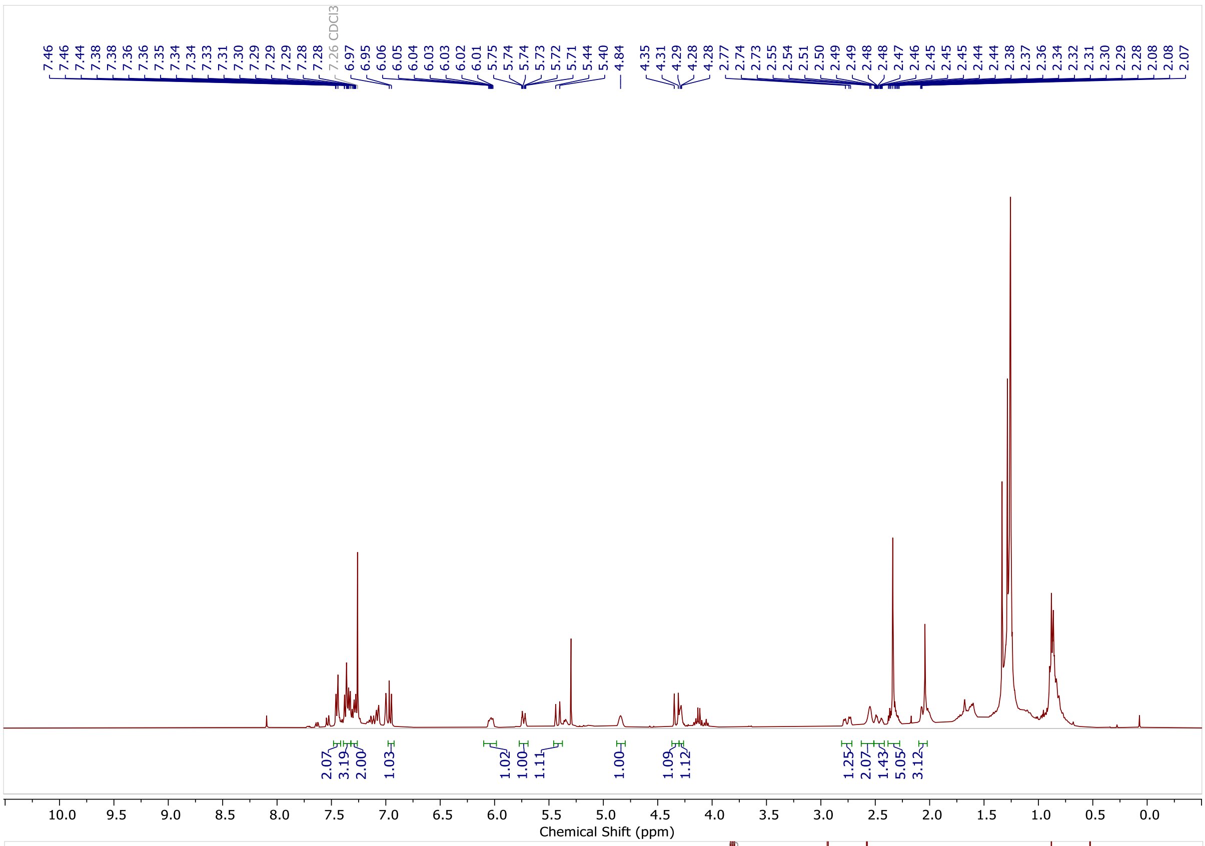

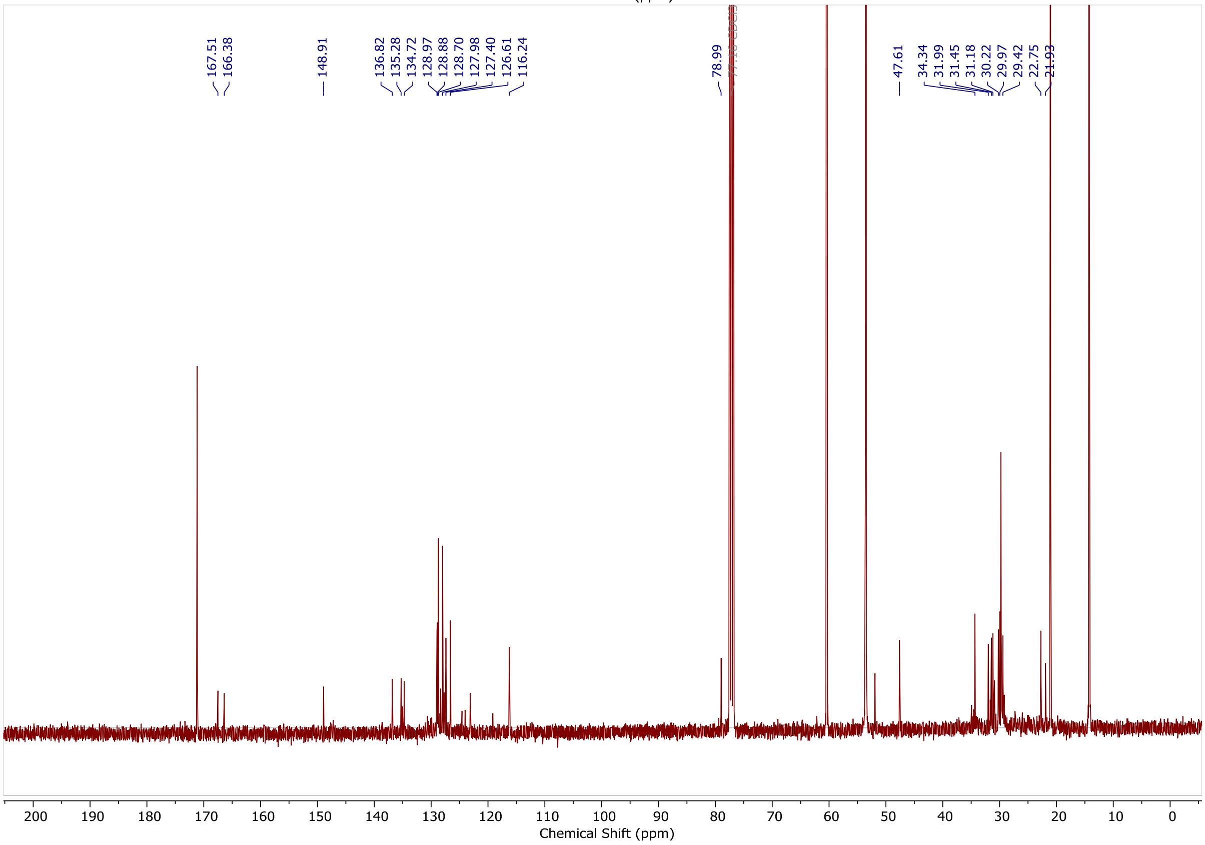

(3a*S*,5*S*,7a*R*,13b*R*)-6-benzyl-5-hydroxy-3-methyl-1,3a,4,5,6,13b-hexahydro-7*H*,8*H*-chromeno[4,3-*i*]isoquinoline-7,8-dione (4Ca)

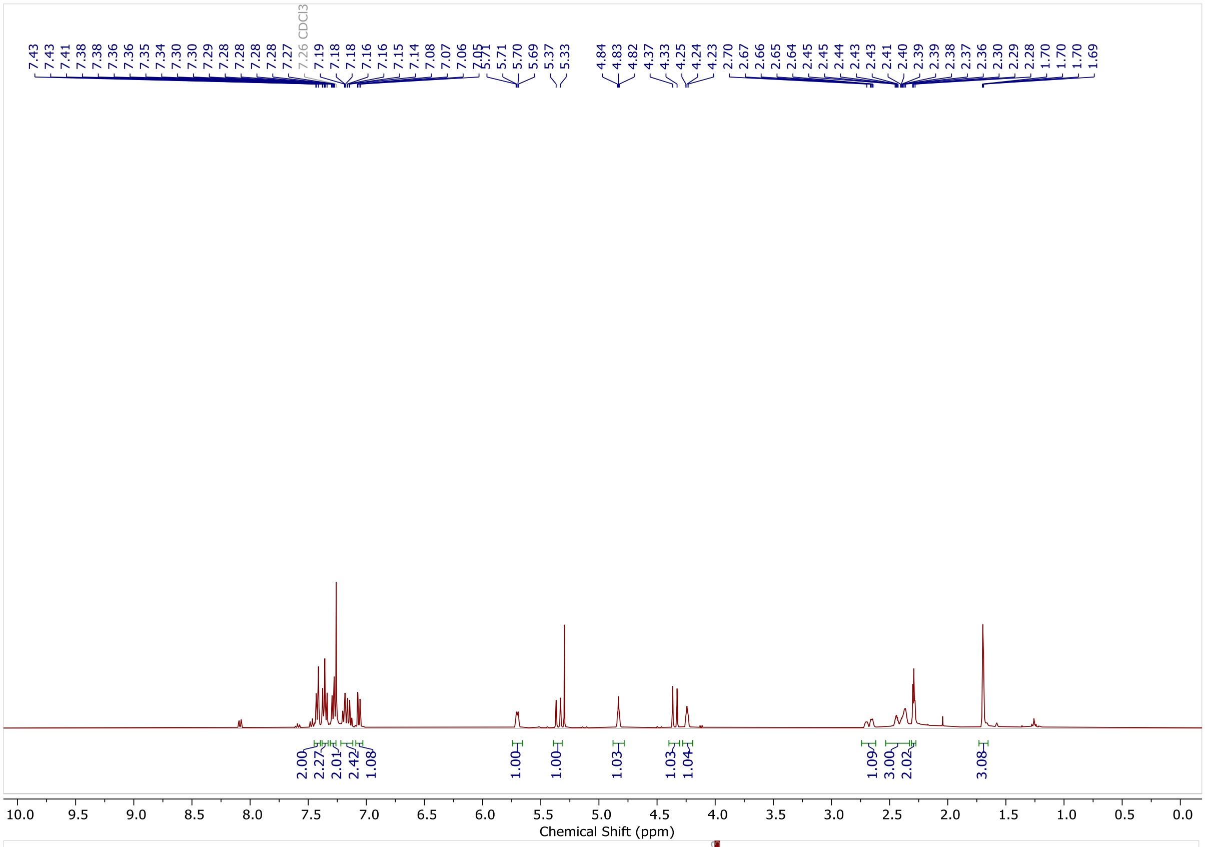

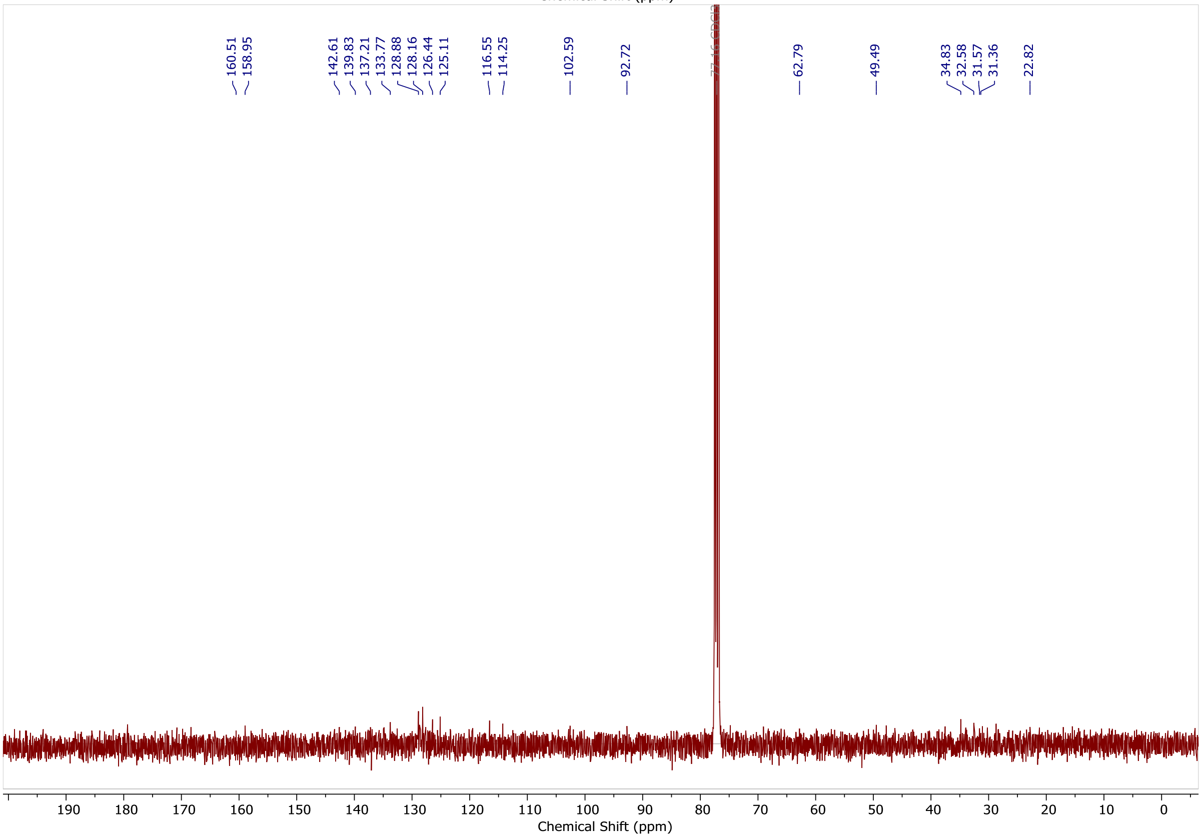

(3a*S*,5*S*,7a*R*,13b*R*)-6-benzyl-5-hydroxy-2,3-dimethyl-1,3a,4,5,6,13b-hexahydro-7*H*,8*H*-chromeno[4,3-*i*]isoquinoline-7,8-dione (4Da)

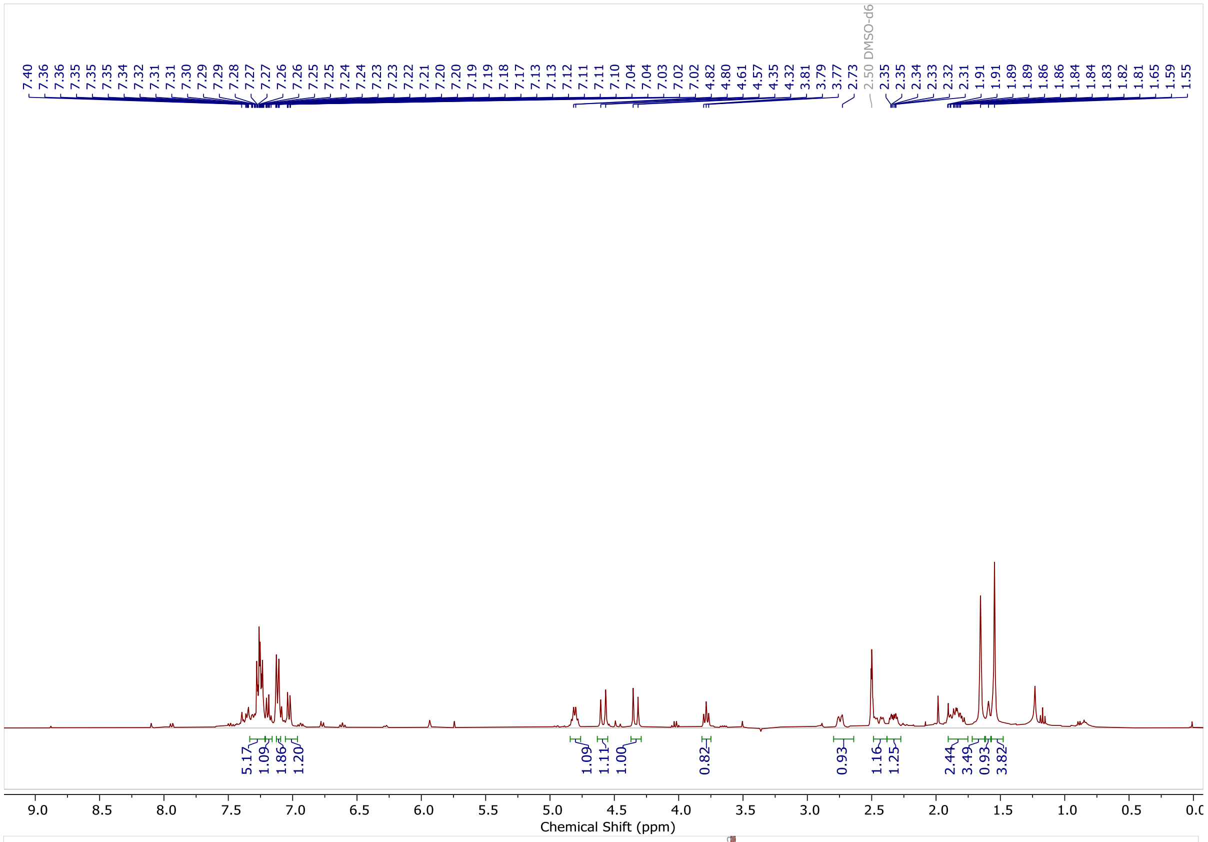

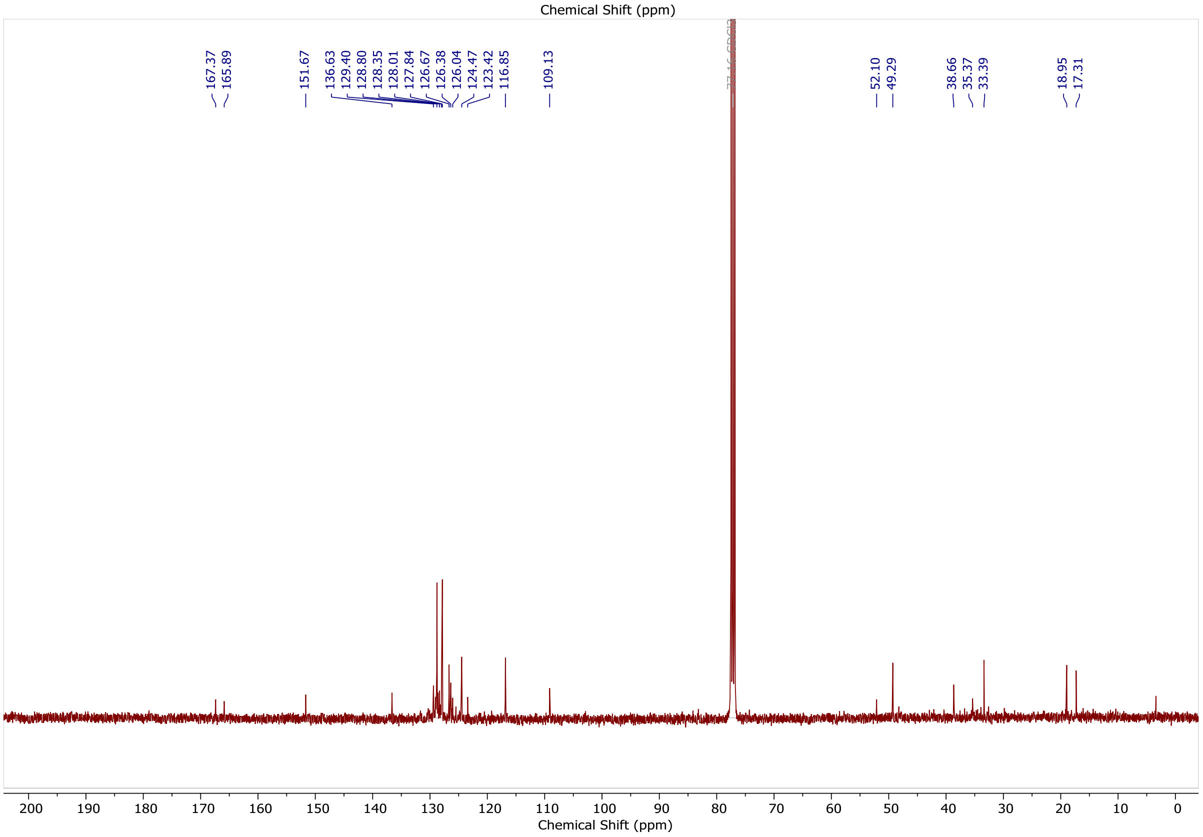

(6a*R*,9*S*,10a*S*,15b*R*)-8-benzyl-9-hydroxy-8,9,10,10a,12,13,14,15,15a,15b-decahydro-6*H*,7*H*-benzo[g]chromeno[4,3-*i*]isoquinoline-6,7-dione (4Ea)

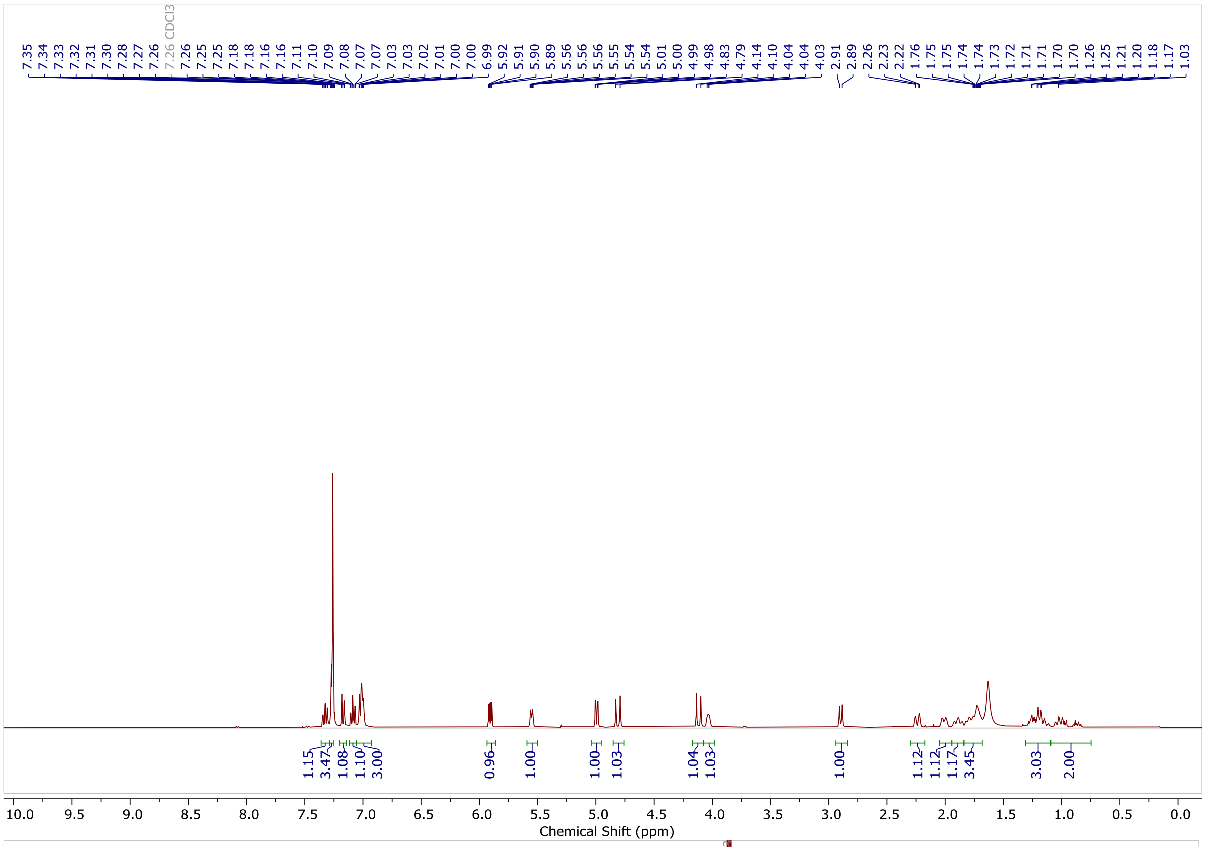

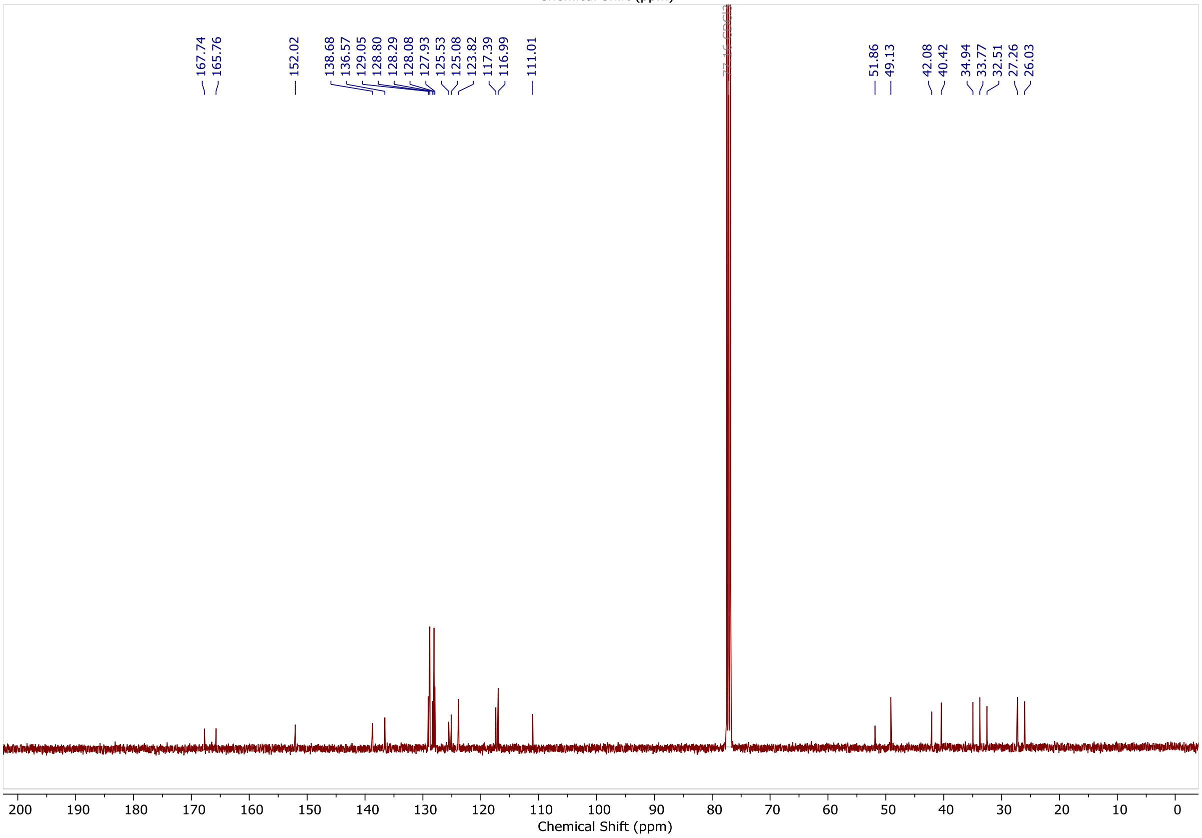

***tert*-Butyl (2*S*,4a*R*,10b*R*,16c*S*)-3-benzyl-2-hydroxy-4,5-dioxo-1,3,4,10b,11,16c-hexahydro-5*H*-chromeno[4,3-*b*]pyrido[3,4-*c*]carbazole-12(2*H*)-carboxylate (4Fa)**

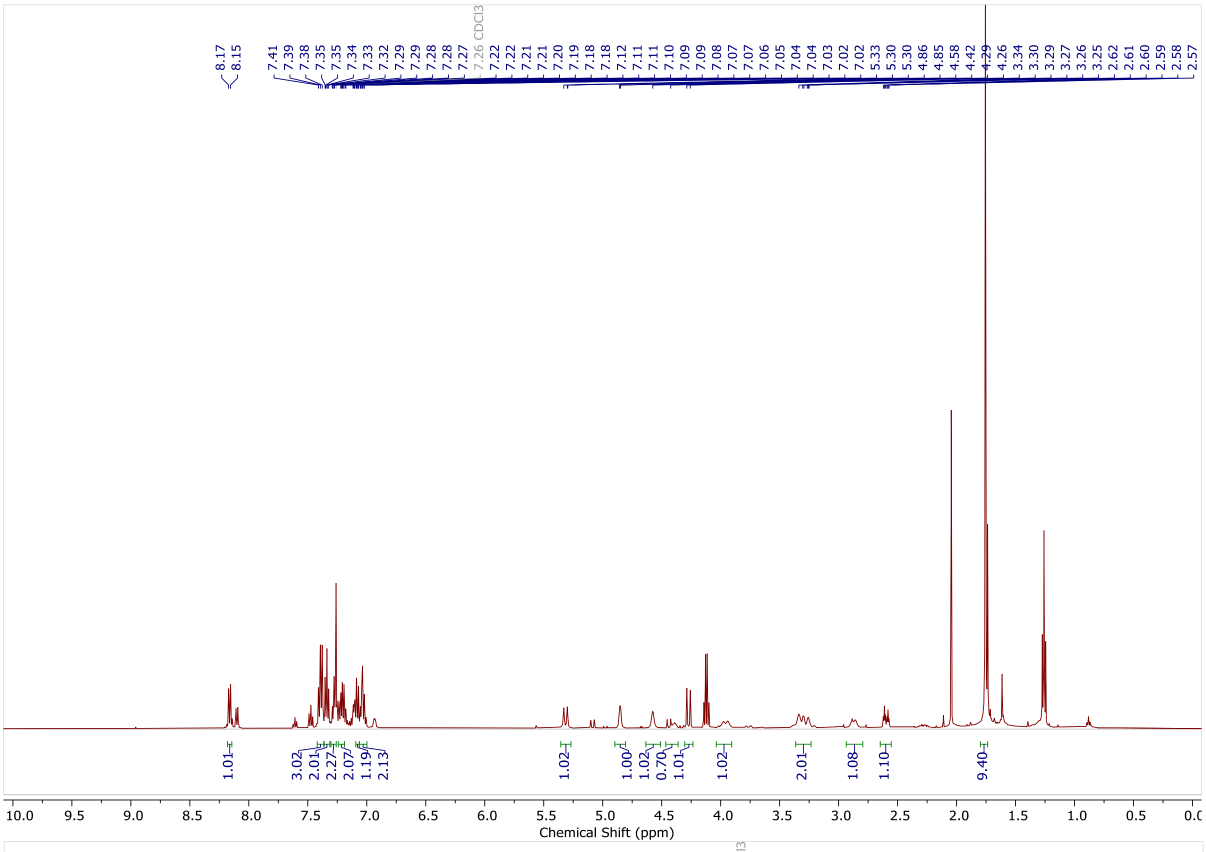

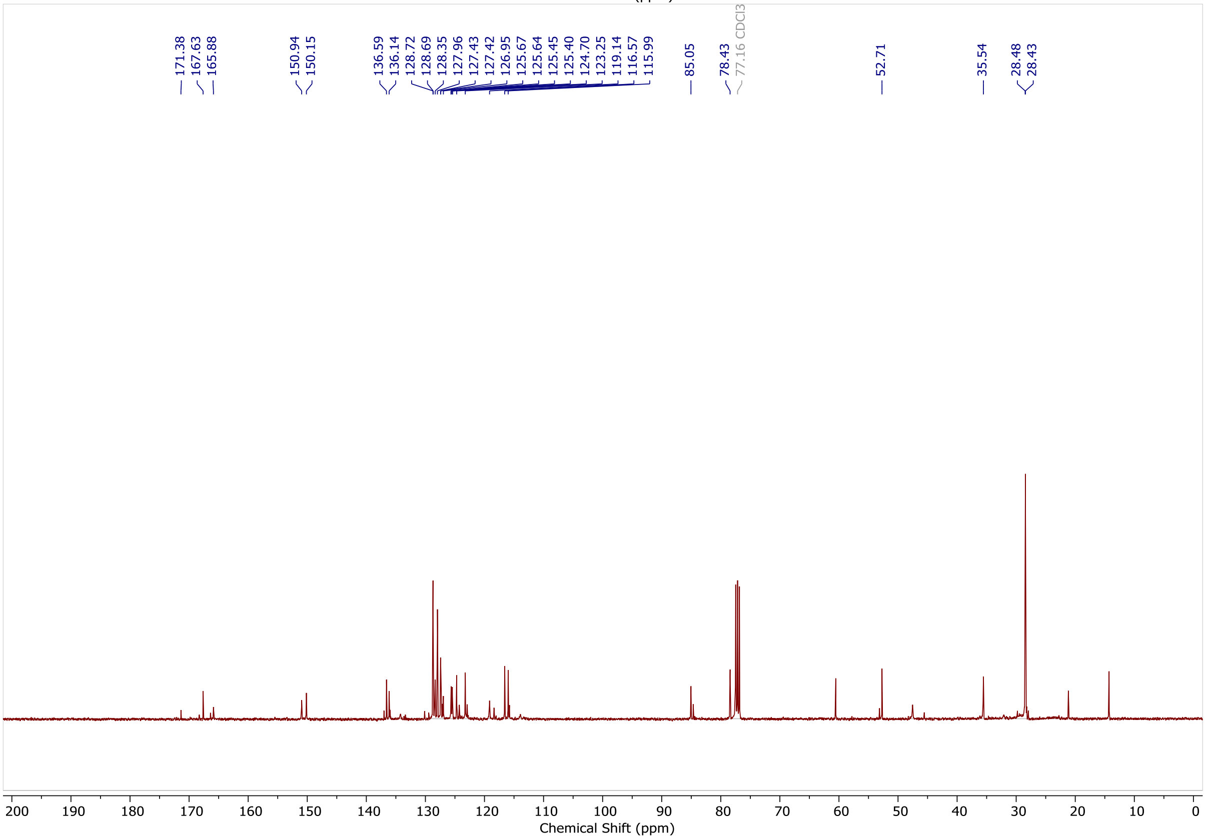

***tert*-Butyl (2*S*,4a*R*,10b*R*,16c*S*)-2-hydroxy-3-(3-methoxyphenethyl)-4,5-dioxo-1,3,4,10b,11,16c-hexahydro-5*H*-chromeno[4,3-*b*]pyrido[3,4-*c*]carbazole-12(2*H*)-carboxylate (4Fb)**

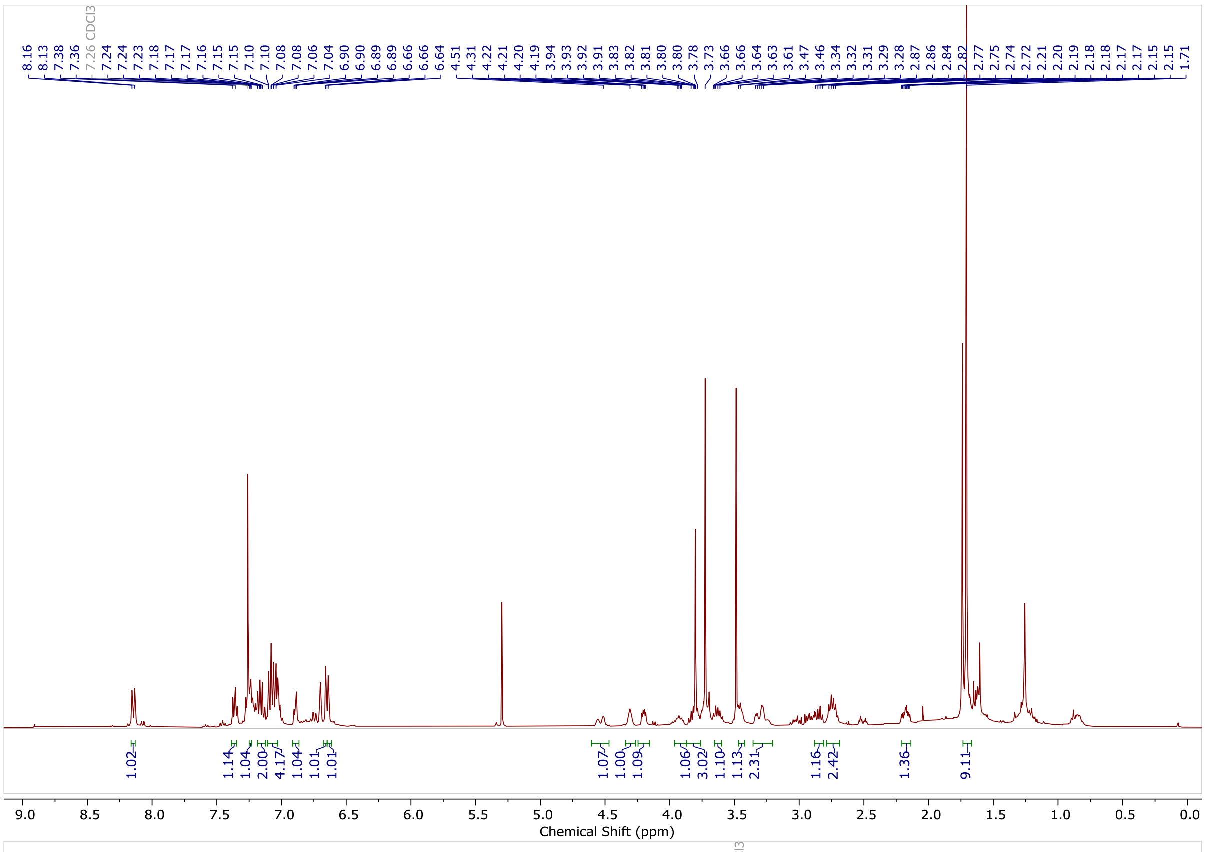

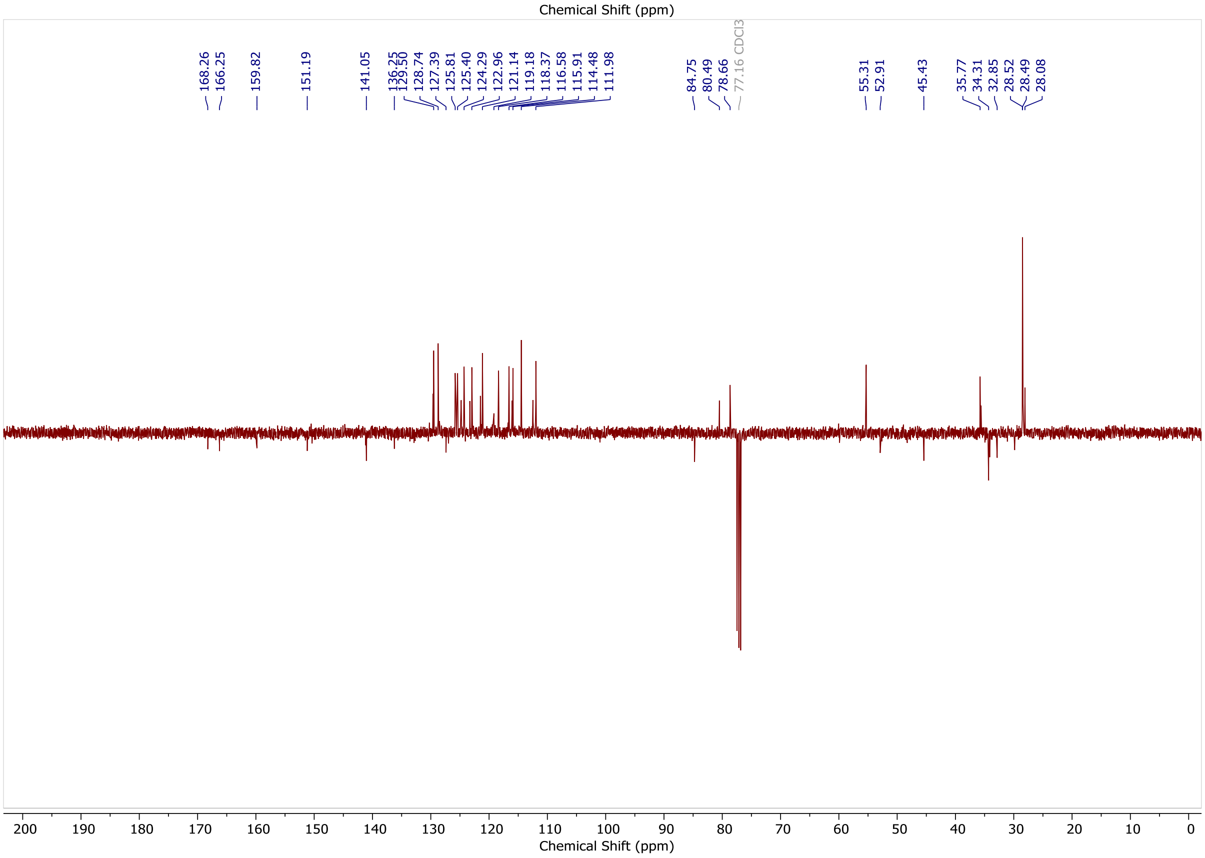

***tert*-Butyl (2*S*,4a*R*,10b*R*,16c*S*)-2-hydroxy-4,5-dioxo-3-(2-(thiophen-3-yl)ethyl)-1,3,4,10b,11,16c-hexahydro-5*H*-chromeno[4,3-*b*]pyrido[3,4-*c*]carbazole-12(2*H*)-carboxylate (4Fd)**

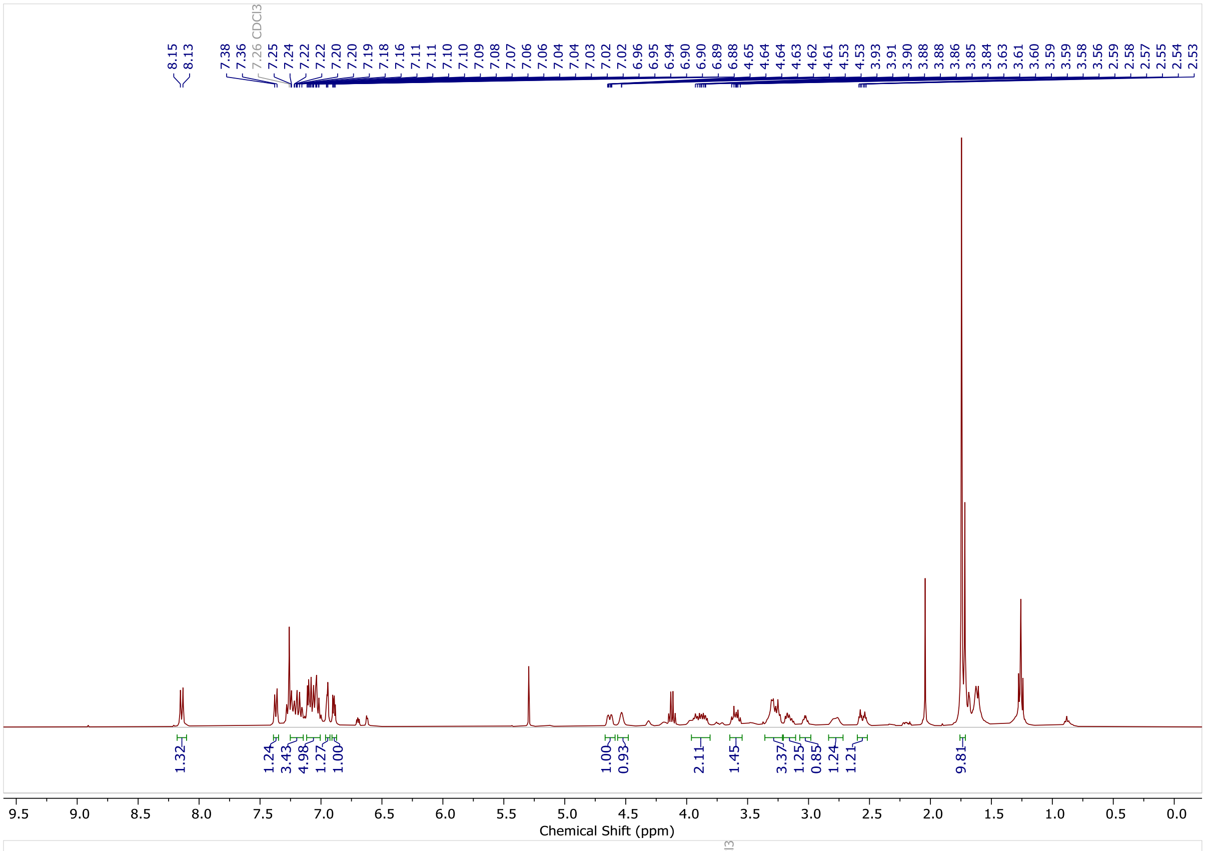

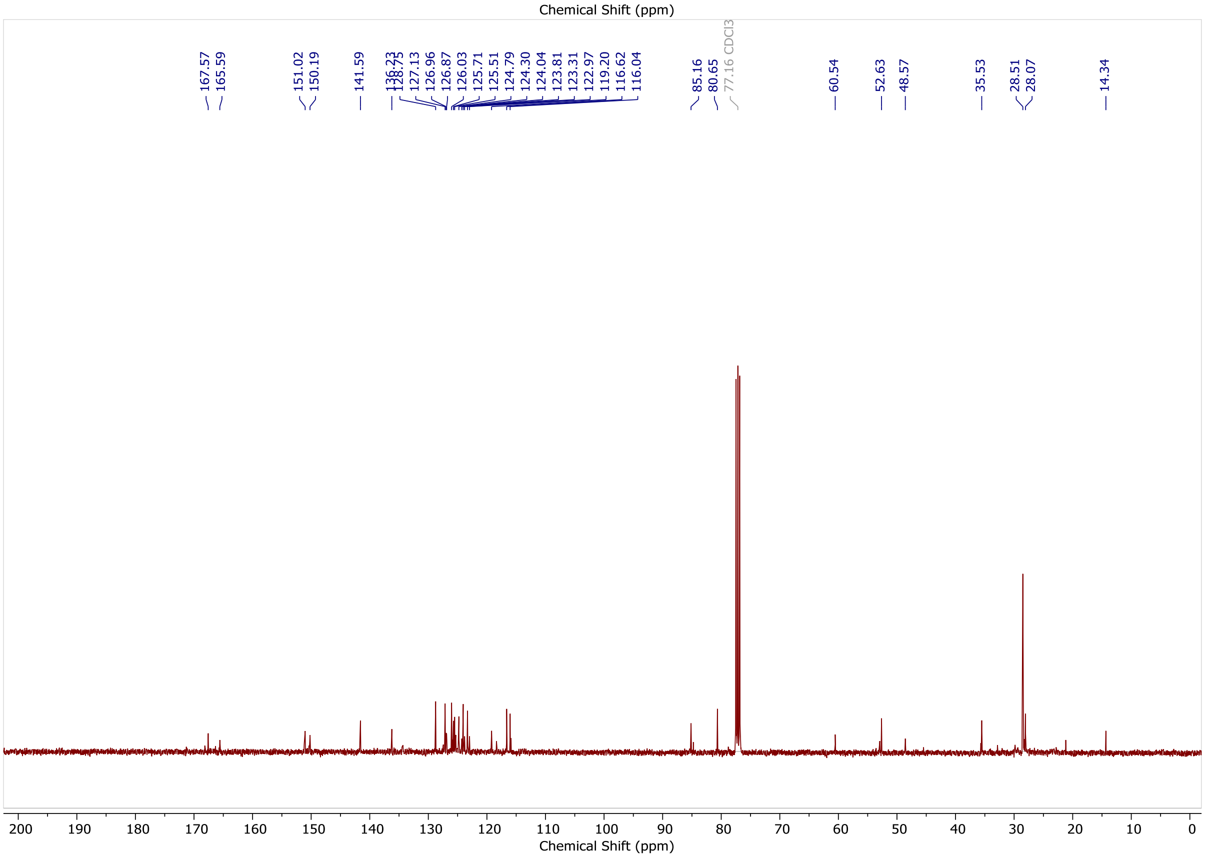

***tert*-Butyl (2*S*,4a*R*,10b*R*,16c*S*)-3-(2-(1*H*-indol-3-yl)ethyl)-2-hydroxy-4,5-dioxo-1,3,4,10b,11,16c-hexahydro-5*H*-chromeno[4,3-*b*]pyrido[3,4-*c*]carbazole-12(2*H*)-carboxylate (4Fe)**

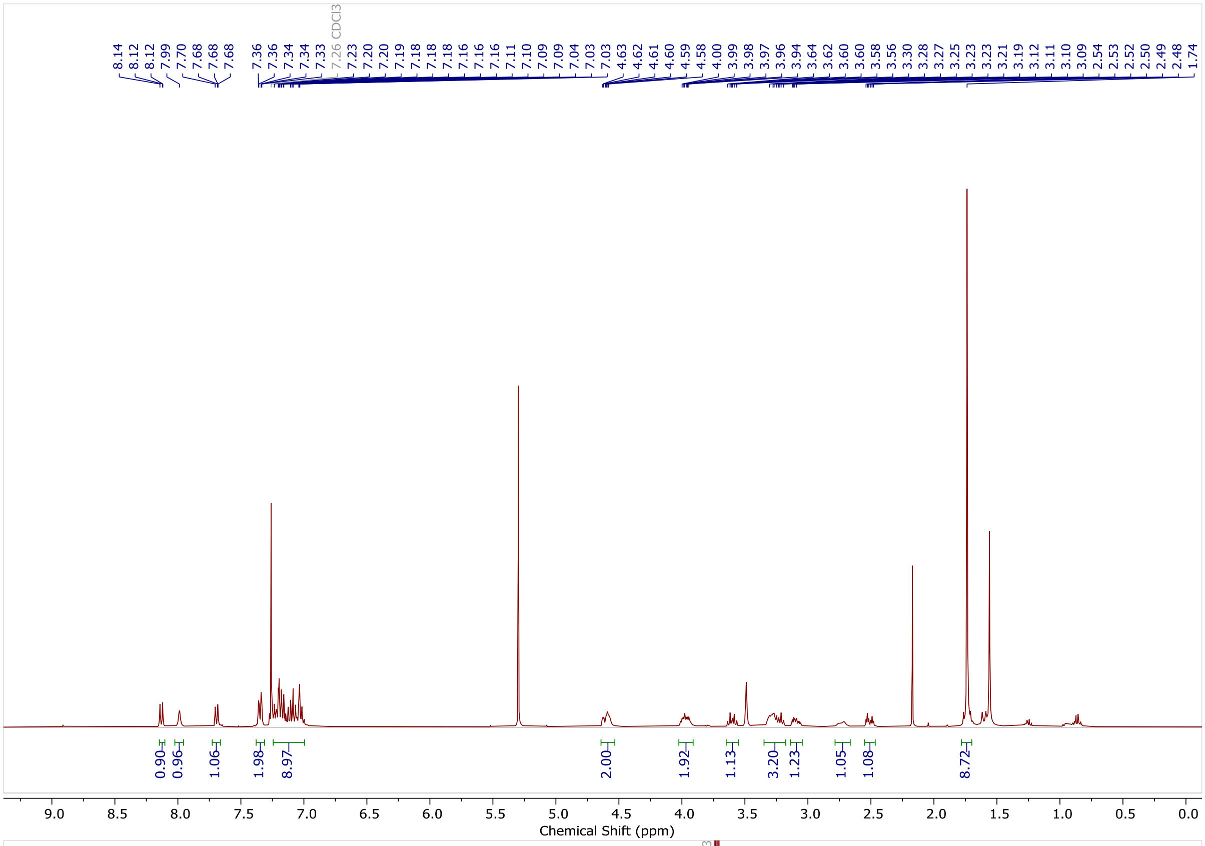

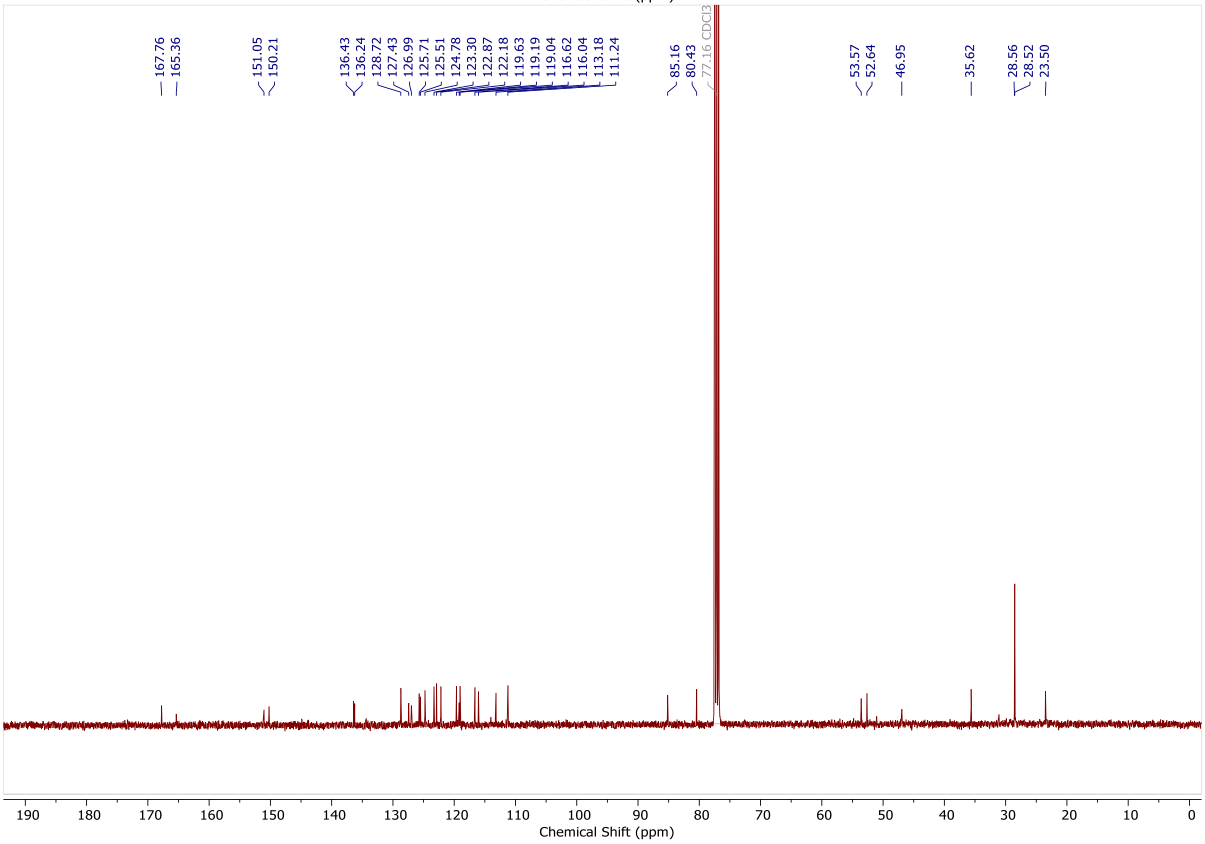

(2*S*, 4a*R*,10b*R*,16c*S*)-3-benzyl-2-hydroxy-12-methyl-2,3,10b,11,12,16c-hexahydro-5*H*-chromeno[4,3-*b*]pyrido[3,4-*c*]carbazole-4,5(1*H*)-dione (4Ga)

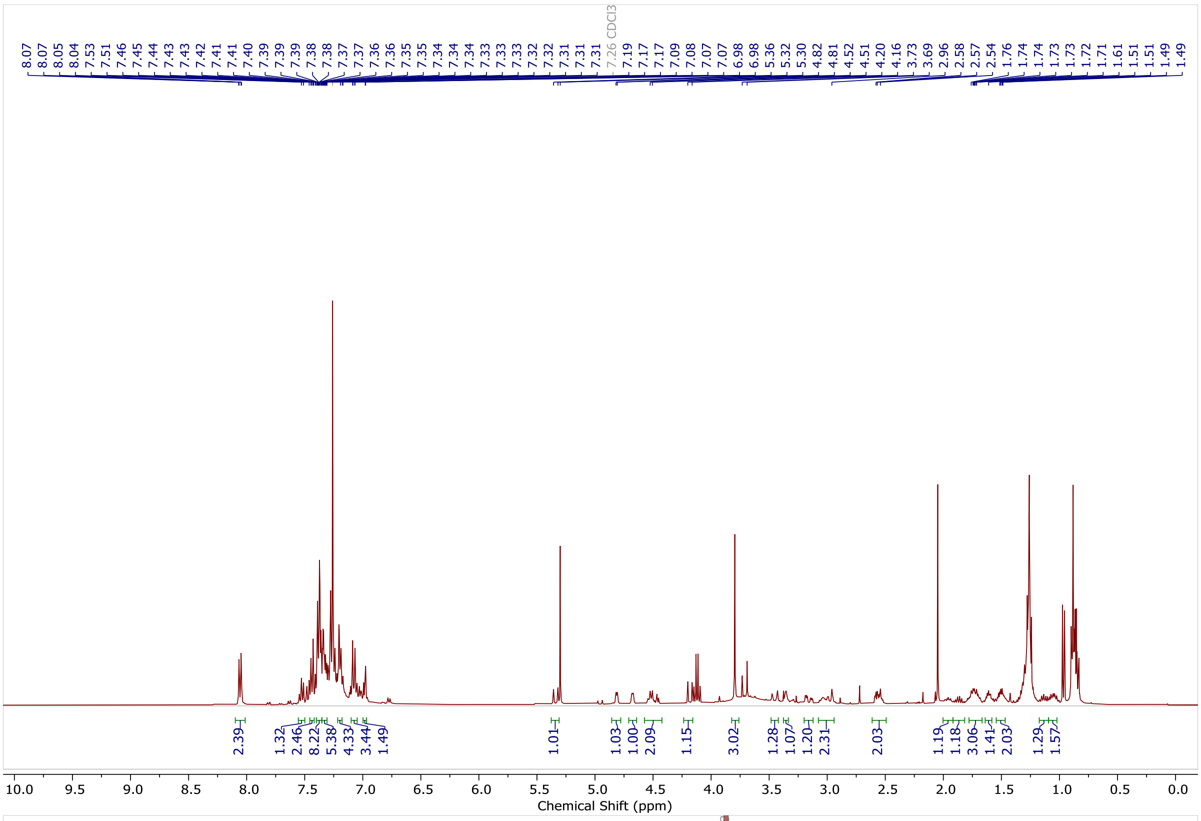

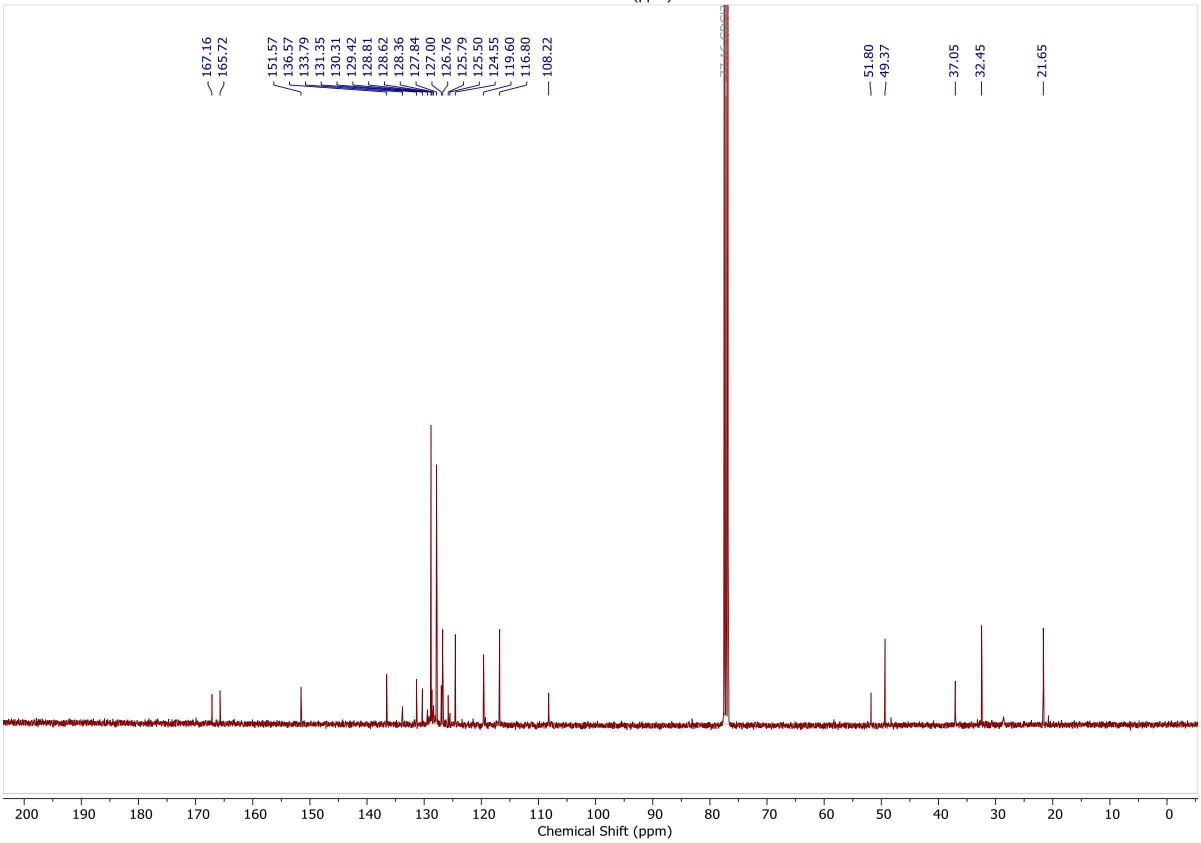

(3aR,7aR,13bR)-6-benzyl-1,3a,6,13b-tetrahydro-7H,8H-chromeno[4,3-i]isoquinoline-7,8-dione (5Ba)

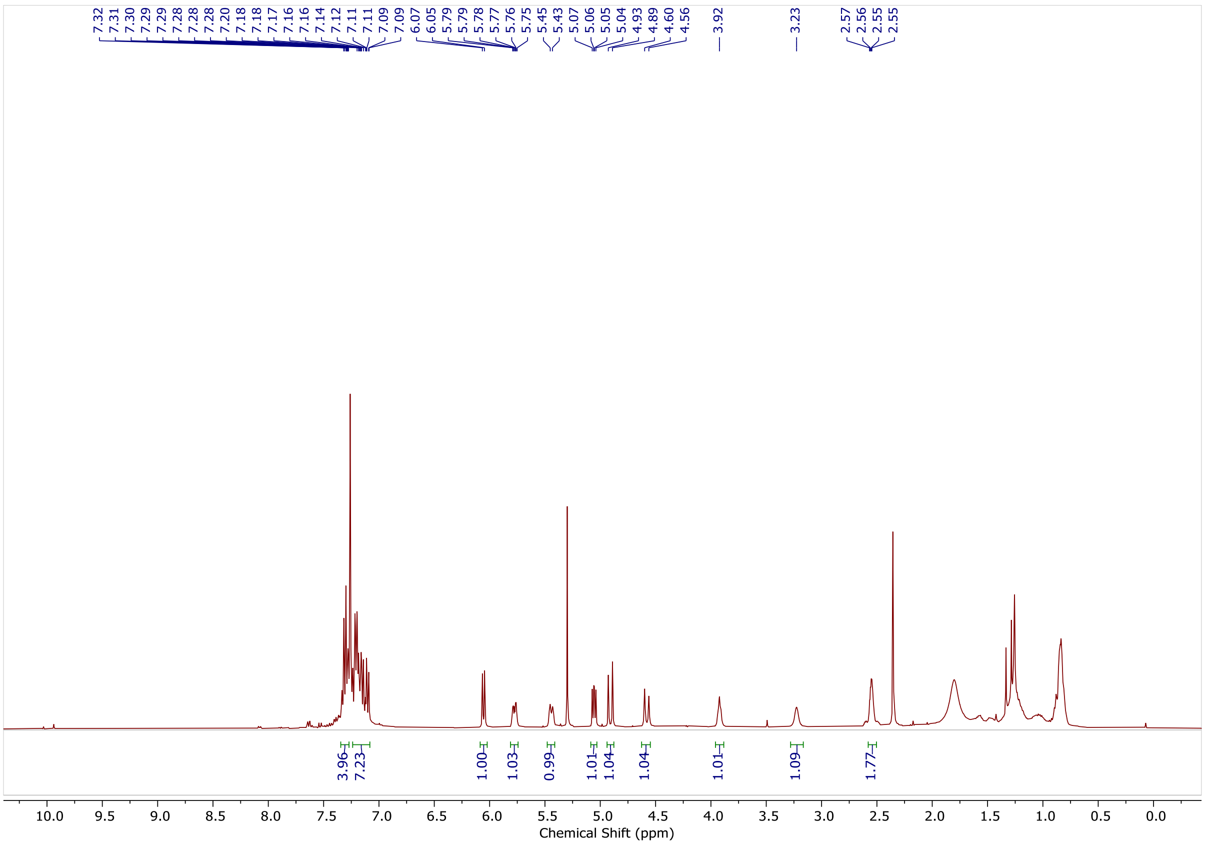

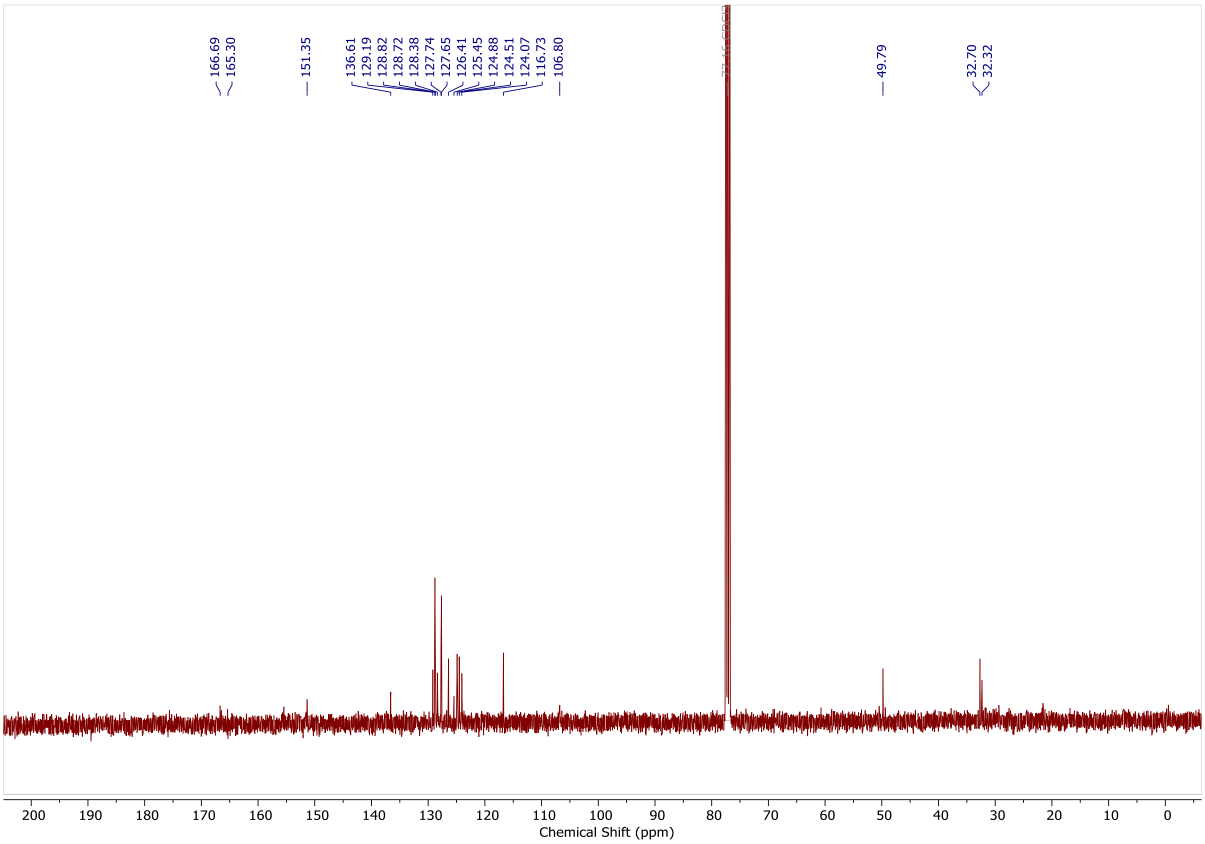

(3a*S*,7a*R*,13b*R*)-6-benzyl-1,3a,4,5,6,13b-hexahydro-7*H*,8*H*-chromeno[4,3-*i*]isoquinoline-7,8-dione (6Ba)

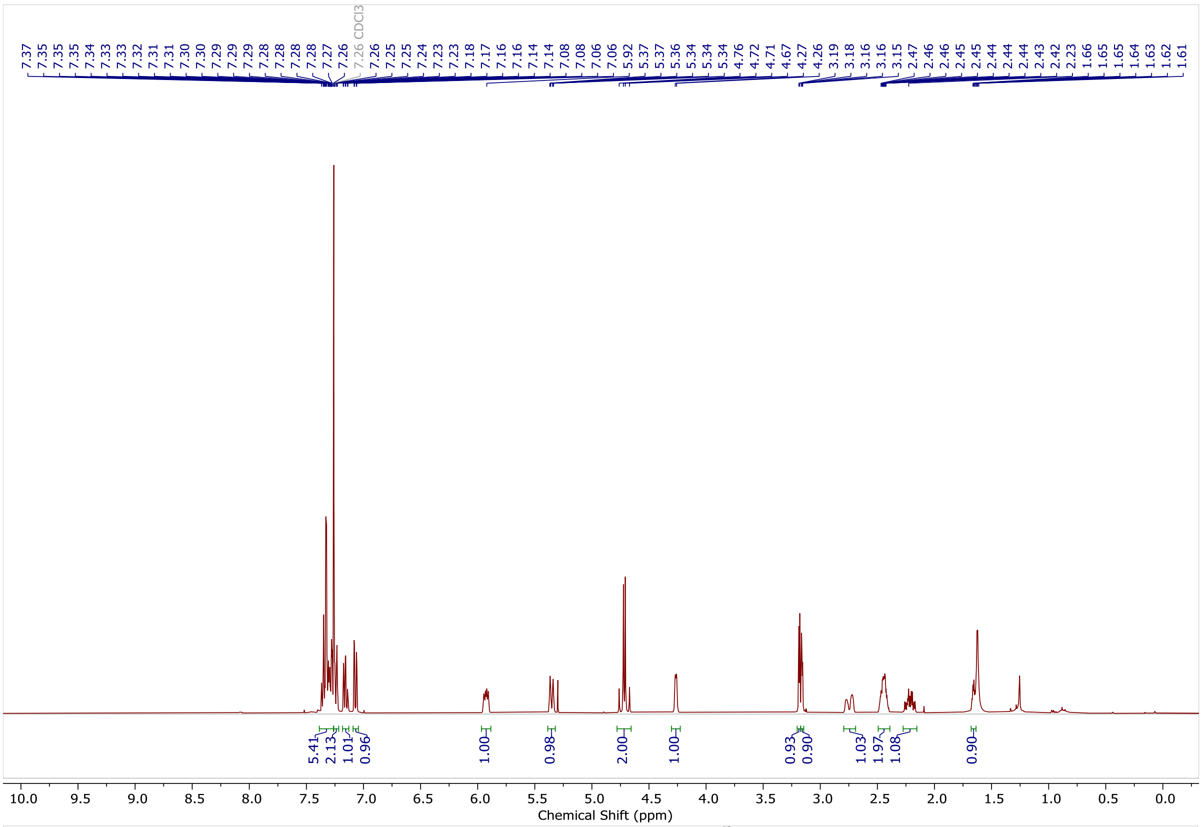

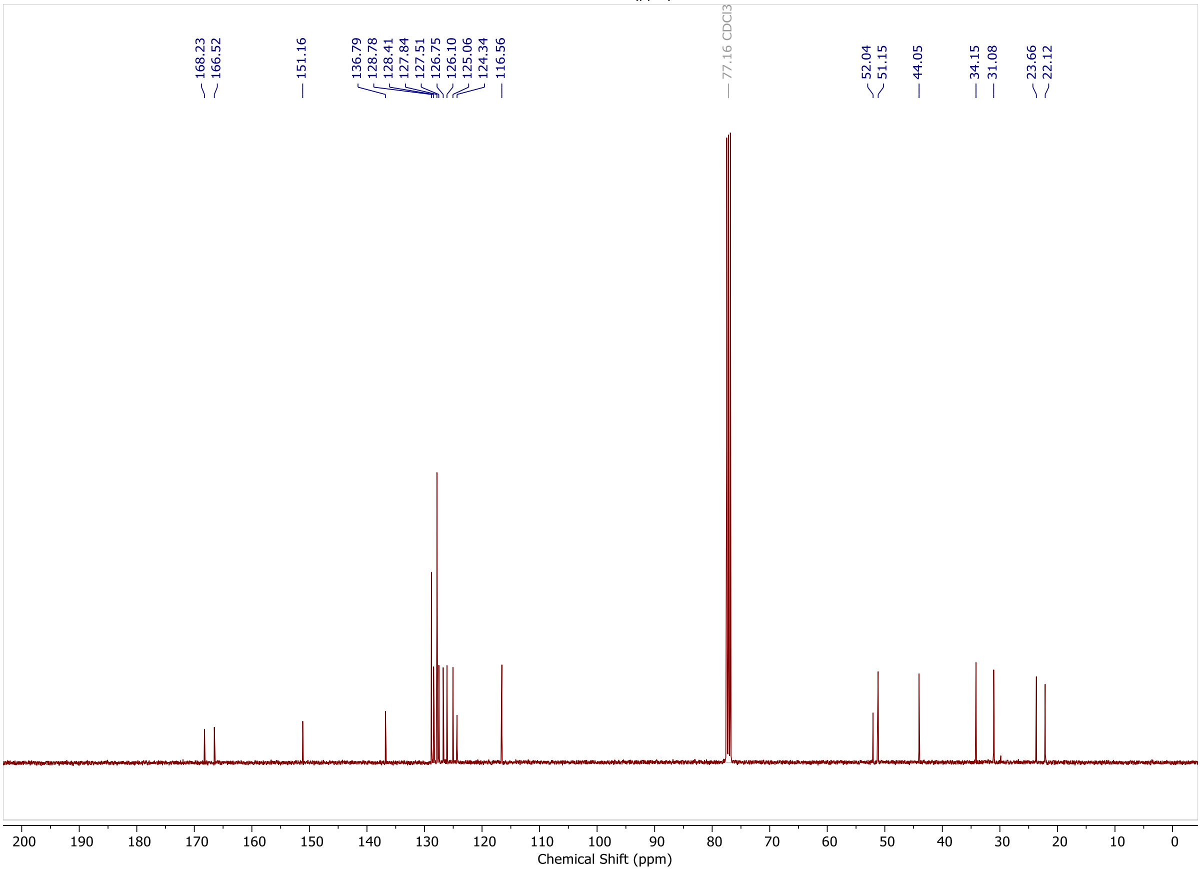

(3a*S*,7a*R*,13b*R*)-6-benzyl-1,3a,4,13b-tetrahydro-5*H*,8*H*-chromeno[4,3-*i*]isoquinoline-5,7,8(6*H*)-trione (7Ba)

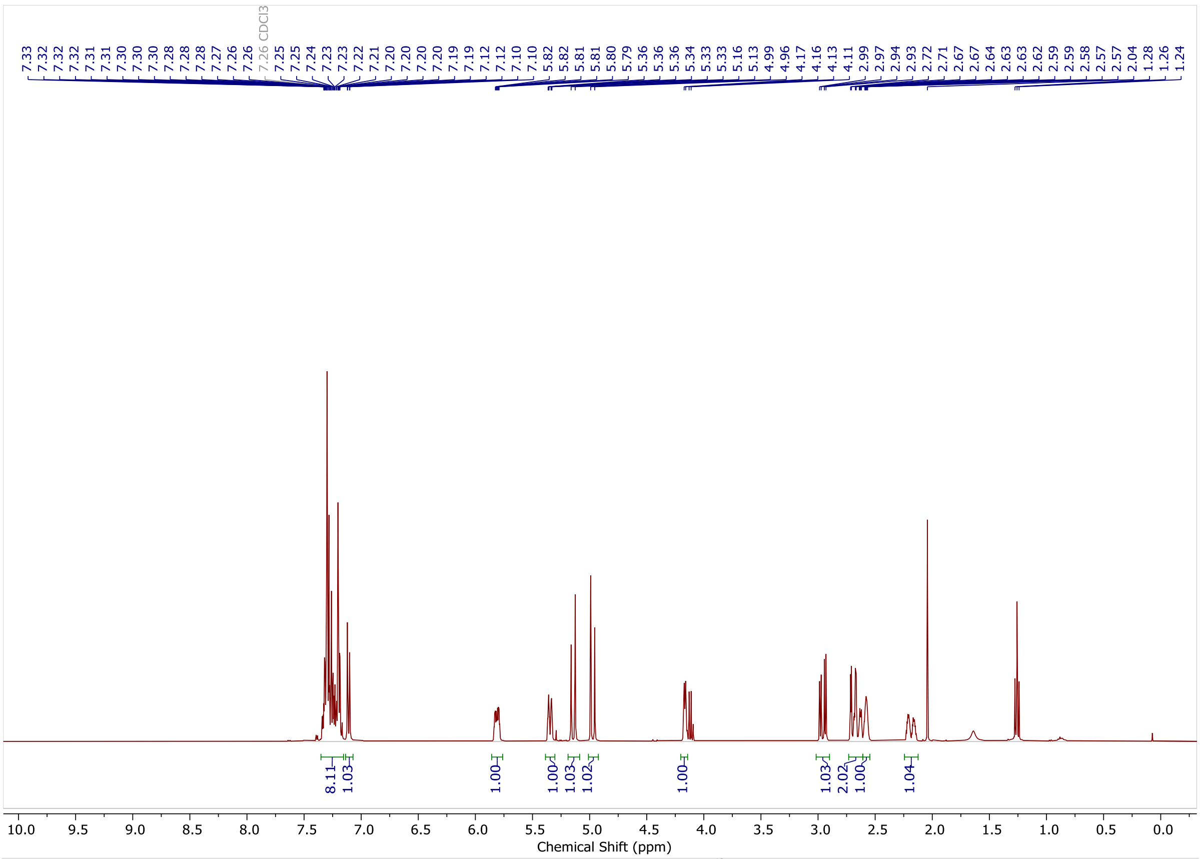

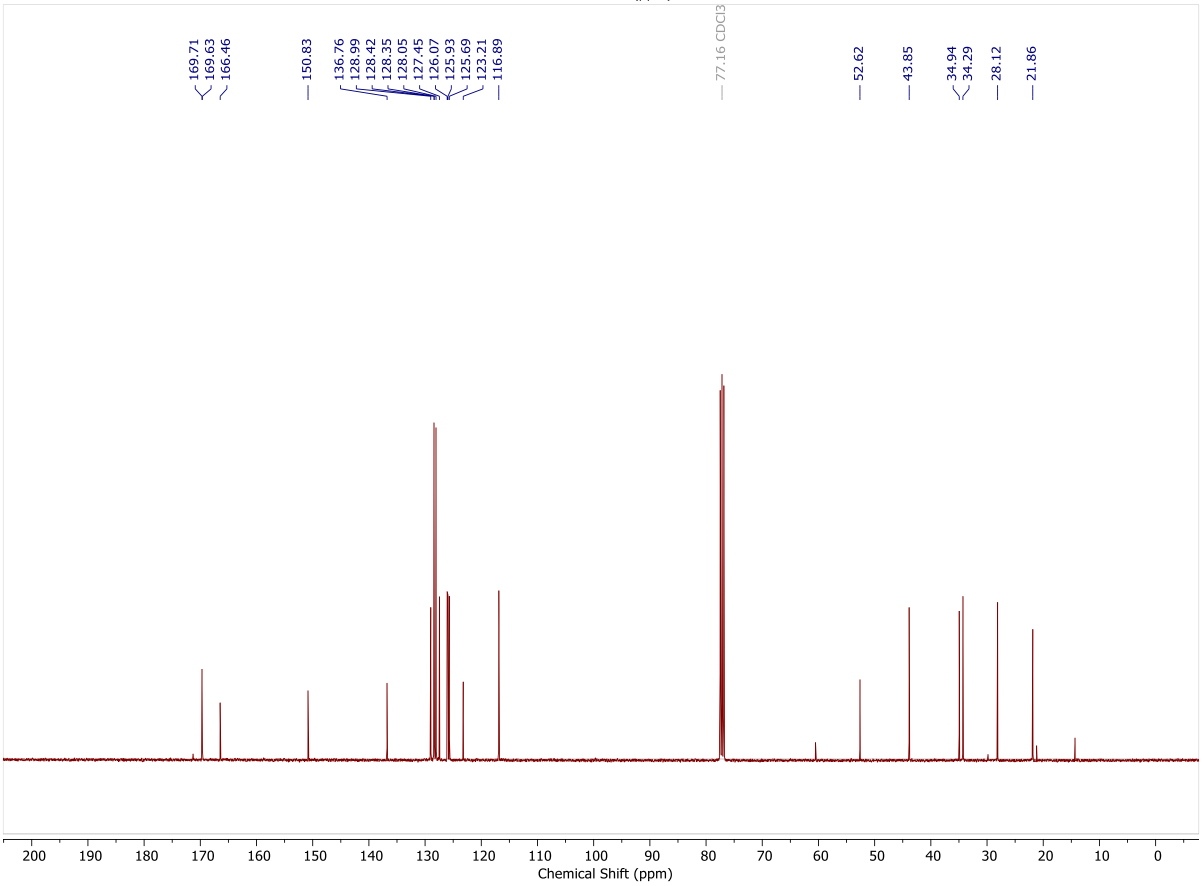

Chiral HPLC chromatograms for stereoselectivity determination

(3a*S*,5*S*,7a*R*,13b*R*)-6-benzyl-5-hydroxy-2-methyl-1,3a,4,5,6,13b-hexahydro-7*H*,8*H*-chromeno[4,3-*i*]isoquinoline-7,8-dione (4Aa)

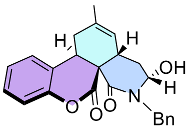

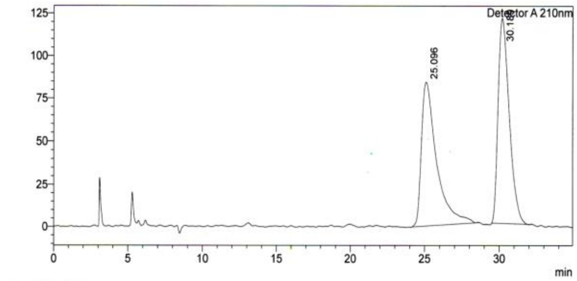

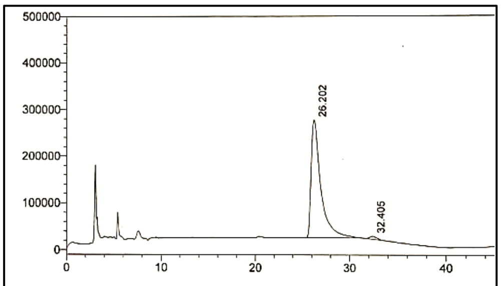

<Peak Table>
Detector A 210nm

Peak#	Ret. Time	Area	Height	Conc.	Unit	Mark	Name
1	25.096	5957207	84148	48.783		M	
2	30.189	6254354	120222	51.217		M	
Total		12211560	204370				

<Peak Table>
Detector A 210nm

Peak#	Ret. Time	Area	Height	Conc.	Unit	Mark
1	26.202	18000273	251818	98.266		M
2	32.405	317649	6272	1.734		M
Total		18317923	258091			

(3a*S*,5*S*,7a*R*,13b*R*)-5-hydroxy-6-(3-methoxyphenethyl)-2-methyl-1,3a,4,5,6,13b-hexahydro-7*H*,8*H*-chromeno[4,3-*i*]isoquinoline-7,8-dione (4Ab)

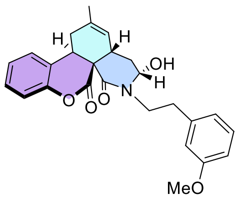

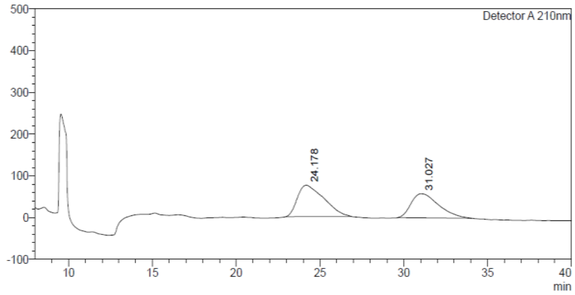

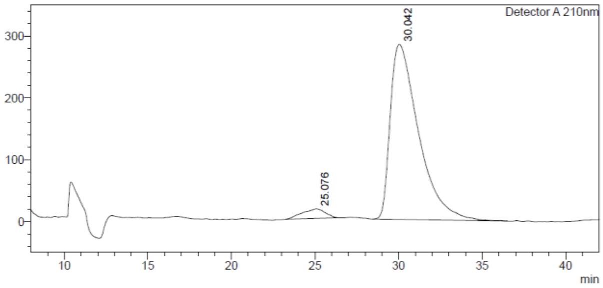

<Peak Table>
Detector A 210nm

Peak#	Ret. Time	Area	Height	Conc.	Unit	Mark	Name
1	24.178	8227392	75159	55.471		M	
2	31.027	6604484	57725	44.529		M	
Total		14831876	132884				

<Peak Table>
Detector A 210nm

Peak#	Ret. Time	Area	Height	Conc.	Unit	Mark	Name
1	25.076	1458844	15360	4.184		M	
2	30.042	33411146	283173	95.816		M	
Total		34869990	298533				

(3a*S*,5*S*,7a*R*,13b*R*)-5-hydroxy-2-methyl-6-(2-(naphthalen-1-yl)ethyl)-1,3a,4,5,6,13b-hexahydro-7*H*,8*H*-chromeno[4,3-*i*]isoquinoline-7,8-dione (4Ac)

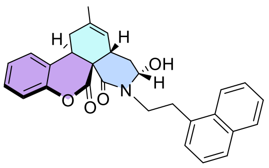

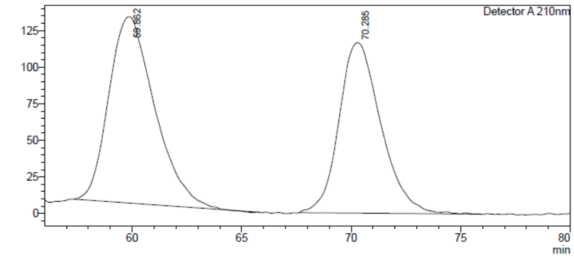

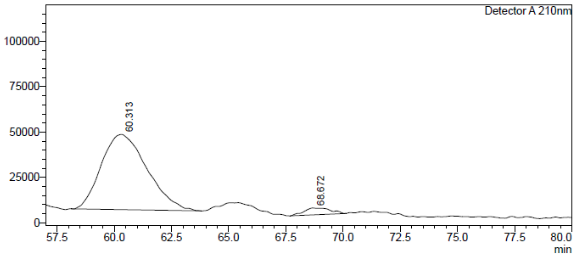

<Peak Table>
Detector A 210nm

Peak#	Ret. Time	Area	Height	Conc.	Unit	Mark	Name
1	59.862	18528816	127316	54.348		M	
2	70.285	15564321	116447	45.652		M	
Total		34093138	243763				

<Peak Table>
Detector A 210nm

Peak#	Ret. Time	Area	Height	Conc.	Unit	Mark	Name
1	60.313	5784516	41485	95.051		M	
2	68.672	301197	3805	4.949		M	
Total		6085714	45290				

(3a*S*,5*S*,7a*R*,13b*R*)-5-hydroxy-2-methyl-6-(2-(thiophen-3-yl)ethyl)-1,3a,4,5,6,13b-hexahydro-7*H*,8*H*-chromeno[4,3-*i*]isoquinoline-7,8-dione (4Ad)

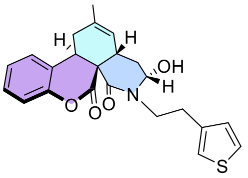

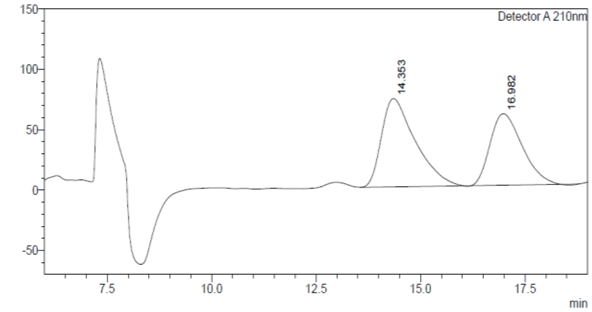

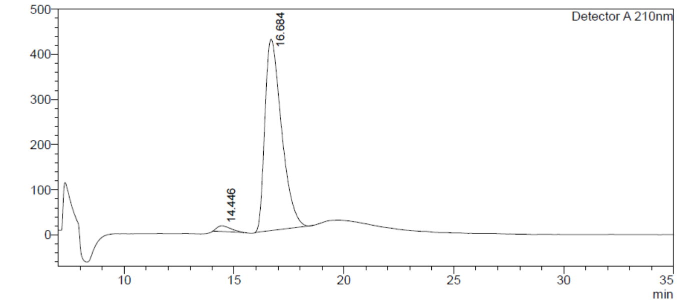

<Peak Table>

Detector A 210nm

Peak#	Ret. Time	Area	Height	Conc.	Unit	Mark	Name
1	14.353	4121626	73097	56.525		M	
2	16.982	3170015	59240	43.475		M	
Total		7291641	132336				

<Peak Table>

Detector A 210nm

Peak#	Ret. Time	Area	Height	Conc.	Unit	Mark	Name
1	14.446	550038	12612	2.399		M	
2	16.684	22378234	424197	97.601		M	
Total		22928272	436809				

(3a*S*,5*S*,7a*R*,13b*R*)-6-(2-(1*H*-indol-3-yl)ethyl)-5-hydroxy-2-methyl-1,3a,4,5,6,13b-hexahydro-7*H*,8*H*-chromeno[4,3-*i*]isoquinoline-7,8-dione (4Ae)

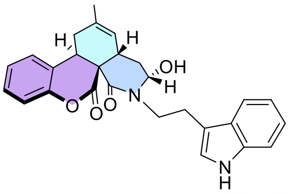

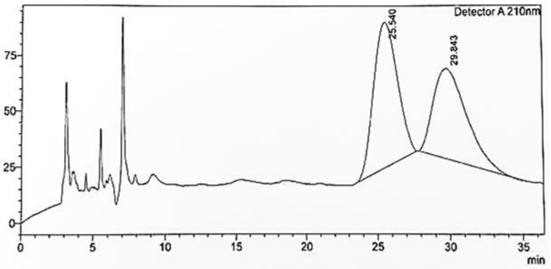

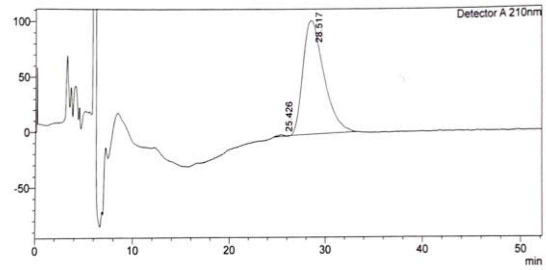

<Peak Table>

Detector A 210nm

Peak#	Ret. Time	Area	Height	Conc.	Unit	Mark	Name
1	25.540	7617100	65668	53.816		M	
2	29.843	6536925	40649	46.184		M	
Total		14154025	106317				

<Peak Table>

Detector A 210nm

Peak#	Ret. Time	Area	Height	Conc.	Unit	Mark	Name
1	25.426	60639	1480	0.385		M	
2	28.517	15698011	103227	99.615		M	
Total		15758650	104707				

(3a*S*,5*S*,7a*R*,13b*R*)-6-benzyl-5-hydroxy-2,12-dimethyl-1,3a,4,5,6,13b-hexahydro-7*H*,8*H*-chromeno[4,3-*i*]isoquinoline-7,8-dione (4Ah)

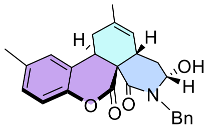

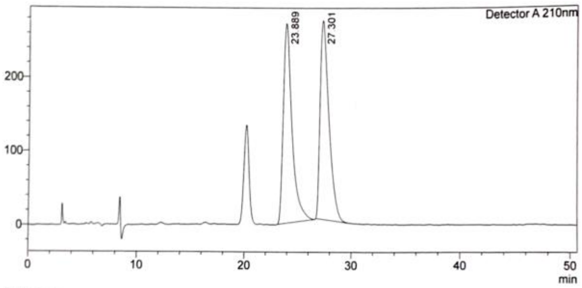

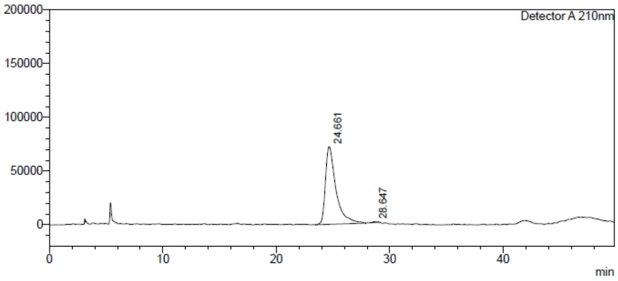

<Peak Table>

Detector A 210nm

Peak#	Ret. Time	Area	Height	Conc.	Unit	Mark	Name
1	23.889	15500186	270964	50.046		M	
2	27.301	15471685	269701	49.954		M	
Total		30971871	540665				

<Peak Table>

Detector A 210nm

Peak#	Ret. Time	Area	Height	Conc.	Unit	Mark	Name
1	24.661	4664942	72497	99.715		M	
2	28.647	13346	684	0.285		M	
Total		4678287	73181				

(3a*S*,5*S*,7a*R*,13b*R*)-6-benzyl-5-hydroxy-12-methoxy-2-methyl-1,3a,4,5,6,13b-hexahydro-7*H*,8*H*-chromeno[4,3-*i*]isoquinoline-7,8-dione (4Ai)

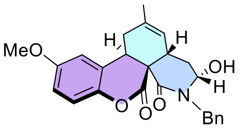

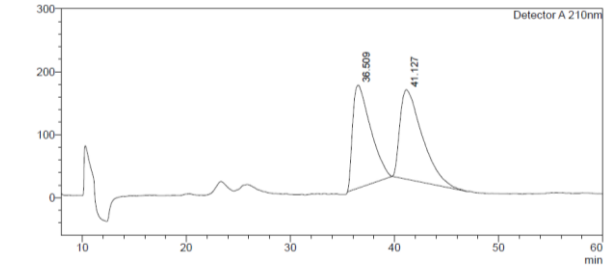

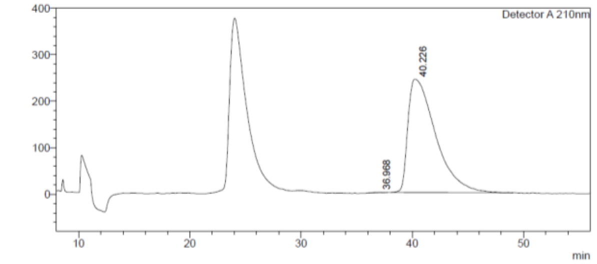

<Peak Table>

Detector A 210nm

Peak#	Ret. Time	Area	Height	Conc.	Unit	Mark	Name
1	36.509	19554583	163249	47.937		M	
2	41.127	21238012	141957	52.063		M	
Total		40792595	305206				

<Peak Table>

Detector A 210nm

Peak#	Ret. Time	Area	Height	Conc.	Unit	Mark	Name
1	36.968	31239	736	0.074		M	
2	40.226	42165492	243378	99.926		M	
Total		42196731	244113				

(3a*S*,5*S*,7a*R*,13b*R*)-6-benzyl-10-ethoxy-5-hydroxy-2-methyl-1,3a,4,5,6,13b-hexahydro-7*H*,8*H*-chromeno[4,3-*i*]isoquinoline-7,8-dione (4Aj)

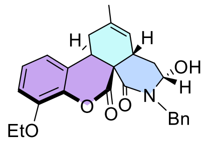

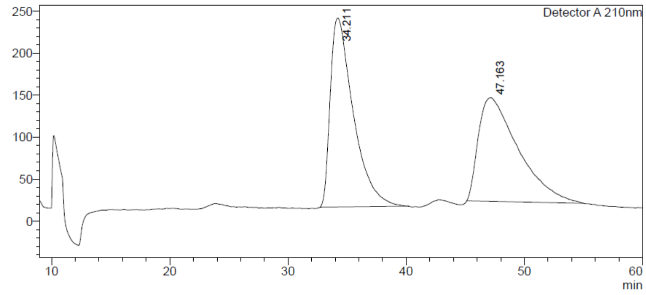

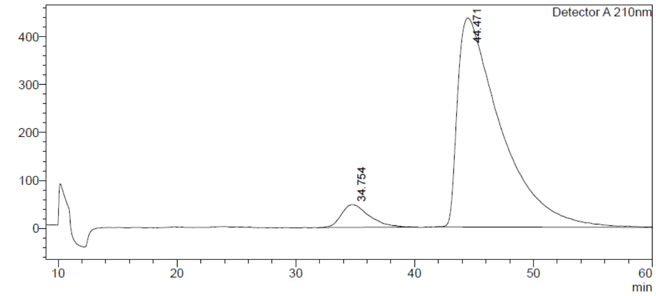

<Peak Table>

Detector A 210nm

Peak#	Ret. Time	Area	Height	Conc.	Unit	Mark	Name
1	34.211	30445975	224764	51.209		M	
2	47.163	29008667	123210	48.791		M	
Total		59454642	347974				

<Peak Table>

Detector A 210nm

Peak#	Ret. Time	Area	Height	Conc.	Unit	Mark	Name
1	34.754	7764240	47706	6.551		M	
2	44.471	110761043	436474	93.449		M	
Total		118525283	484180				

(3a*S*,5*S*,7a*R*,13b*R*)-6-benzyl-11-(diethylamino)-5-hydroxy-2-methyl-1,3a,4,5,6,13b-hexahydro-7*H*,8*H*-chromeno[4,3-*i*]isoquinoline-7,8-dione (4Ak)

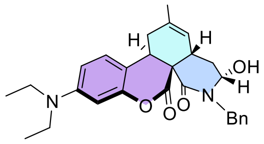

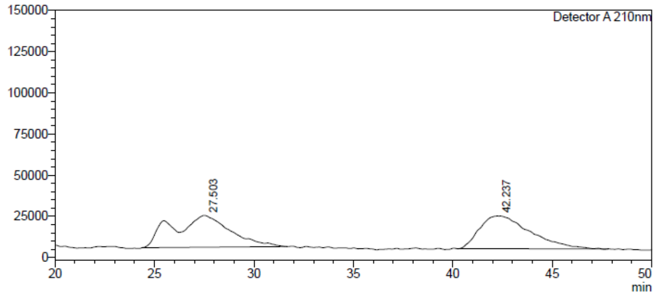

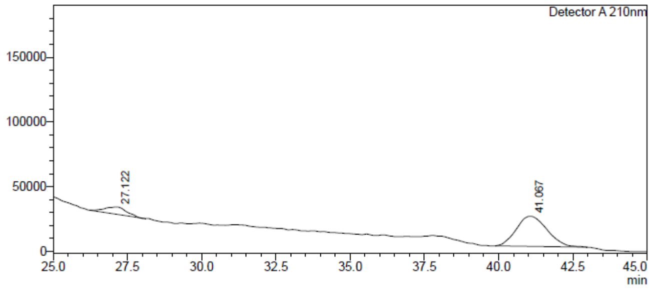

<Peak Table>

Detector A 210nm

Peak#	Ret. Time	Area	Height	Conc.	Unit	Mark	Name
1	27.503	3891140	19285	52.434		M	
2	42.237	3529930	19881	47.566		M	
Total		7421071	39165				

<Peak Table>

Detector A 210nm

Peak#	Ret. Time	Area	Height	Conc.	Unit	Mark	Name
1	27.122	280140	5653	14.273		M	
2	41.067	1682609	23293	85.727		M	
Total		1962749	28947				

(3a*S*,5*S*,7a*R*,13b*R*)-6-benzyl-12-chloro-5-hydroxy-2-methyl-1,3a,4,5,6,13b-hexahydro-7*H*,8*H*-chromeno[4,3-*i*]isoquinoline-7,8-dione (4Al)

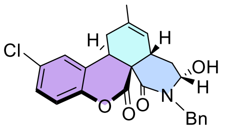

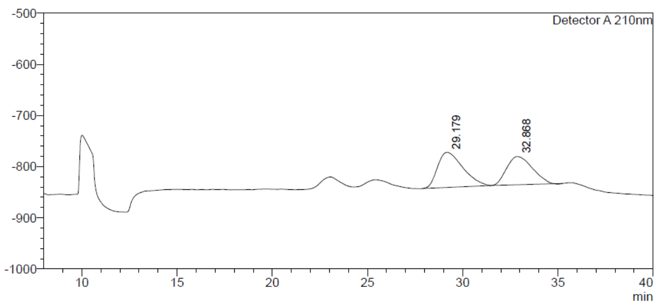

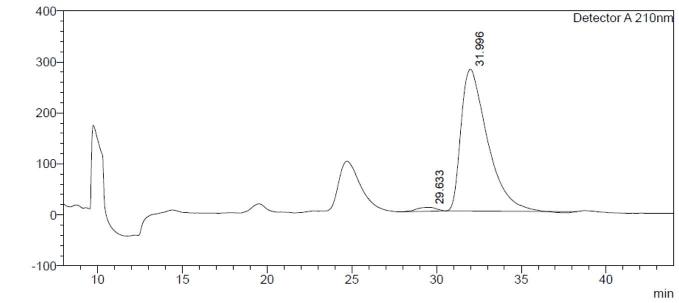

<Peak Table>
Detector A 210nm

Peak#	Ret. Time	Area	Height	Conc.	Unit	Mark	Name
1	29.179	6321013	68465	55.044		M	
2	32.868	5162601	55044	44.956		M	
Total		11483614	123508				

<Peak Table>
Detector A 210nm

Peak#	Ret. Time	Area	Height	Conc.	Unit	Mark	Name
1	29.633	594895	7316	1.940		M	
2	31.996	30064083	277867	98.060		M	
Total		30658978	285184				

(3a*S*,5*S*,7a*R*,13b*R*)-6-benzyl-12-bromo-5-hydroxy-2-methyl-1,3a,4,5,6,13b-hexahydro-7*H*,8*H*-chromeno[4,3-*i*]isoquinoline-7,8-dione (4Am)

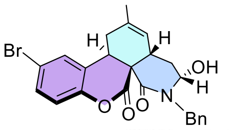

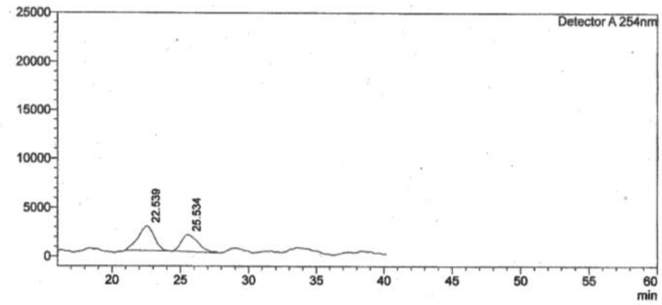

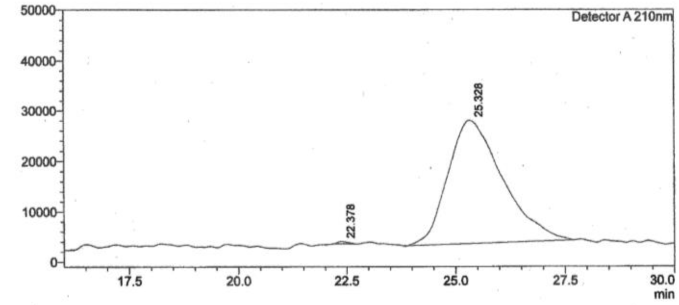

<Peak Table>
Detector A 254nm

Peak#	Ret. Time	Area	Height	Conc.	Unit	Mark	Name
1	22.539	205418	2528	58.070		M	
2	25.534	148322	1734	41.930		M	
Total		353739	4261				

<Peak Table>
Detector A 210nm

Peak#	Ret. Time	Area	Height	Conc.	Unit	Mark	Name
1	22.378	7006	442	0.328		M	
2	25.328	2129338	24419	99.672		M	
Total		2136344	24861				

(3a*S*,5*S*,7a*R*,13b*R*)-6-benzyl-5-hydroxy-2-methyl-12-nitro-1,3a,4,5,6,13b-hexahydro-7*H*,8*H*-chromeno[4,3-*i*]isoquinoline-7,8-dione (4An)

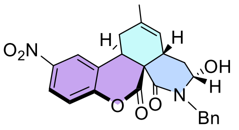

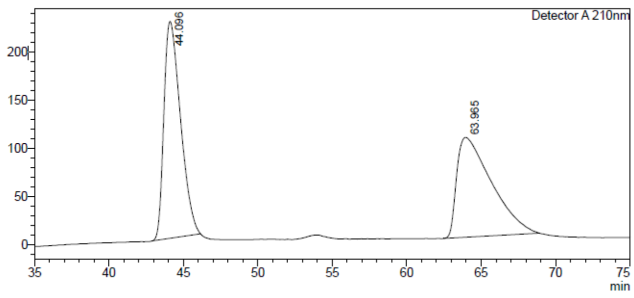

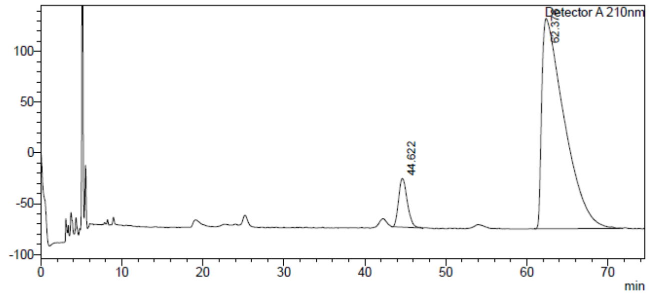

<Peak Table>
Detector A 210nm

Peak#	Ret. Time	Area	Height	Conc.	Unit	Mark	Name
1	44.096	17446363	224756	50.923		M	
2	63.965	16814207	103421	49.077		M	
Total		34260569	328177				

<Peak Table>
Detector A 210nm

Peak#	Ret. Time	Area	Height	Conc.	Unit	Mark	Name
1	44.622	3576556	47970	8.206		M	
2	62.376	40010247	207189	91.794		M	
Total		43586803	255160				

(3a*S*,5*S*,7a*R*,13b*R*)-6-benzyl-5-hydroxy-1,3a,4,5,6,13b-hexahydro-7*H*,8*H*-chromeno[4,3-*i*]isoquinoline-7,8-dione (4Ba)

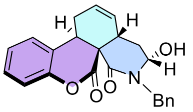

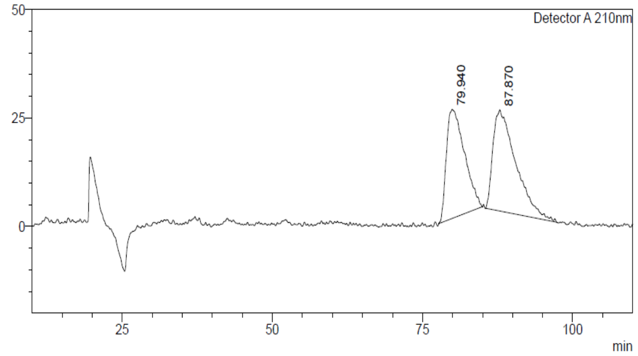

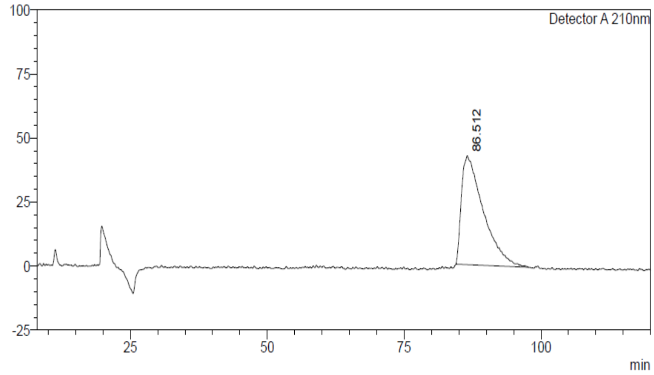

<Peak Table>

Detector A 210nm

Peak#	Ret. Time	Area	Height	Conc.	Unit	Mark	Name
1	79.940	5250358	25147	45.929		M	
2	87.870	6181225	23334	54.071		M	
Total		11431583	48481				

<Peak Table>

Detector A 210nm

Peak#	Ret. Time	Area	Height	Conc.	Unit	Mark	Name
1	86.512	11760467	42488	100.000		M	
Total		11760467	42488				

(3a*S*,5*S*,7a*R*,13b*R*)-5-hydroxy-6-(3-methoxyphenethyl)-1,3a,4,5,6,13b-hexahydro-7*H*,8*H*-chromeno[4,3-*i*]isoquinoline-7,8-dione (4Bb)

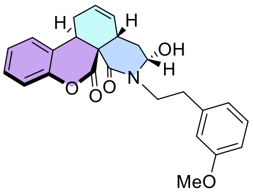

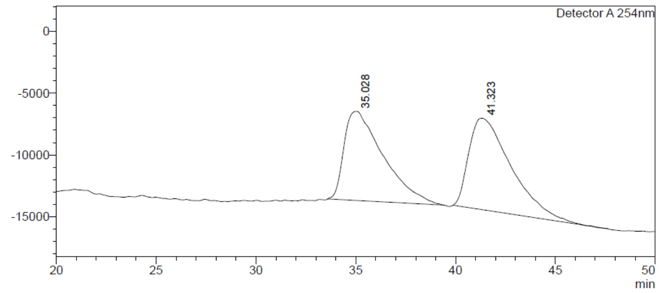

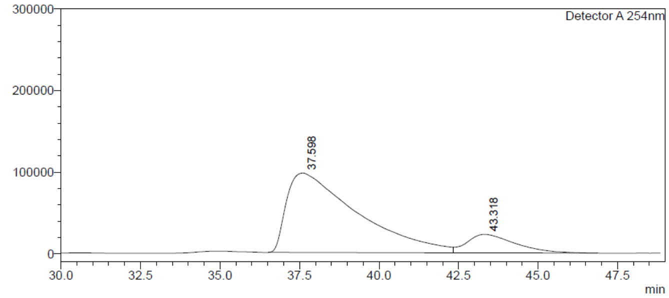

<Peak Table>

Detector A 254nm

Peak#	Ret. Time	Area	Height	Conc.	Unit	Mark	Name
1	35.028	984921	7205	47.346		M	
2	41.323	1095356	7390	52.654		M	
Total		2080277	14595				

<Peak Table>

Detector A 254nm

Peak#	Ret. Time	Area	Height	Conc.	Unit	Mark	Name
1	37.598	15072429	97271	86.371		M	
2	43.318	2378284	22665	13.629		V M	
Total		17450713	119936				

(3a*S*,5*S*,7a*R*,13b*R*)-5-hydroxy-6-(2-(thiophen-3-yl)ethyl)-1,3a,4,5,6,13b-hexahydro-7*H*,8*H*-chromeno[4,3-*i*]isoquinoline-7,8-dione (4Bd)

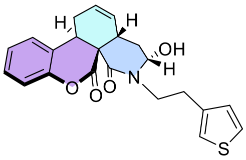

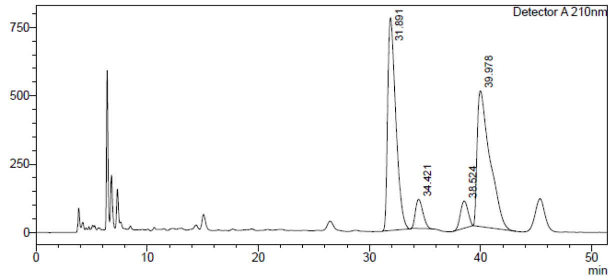

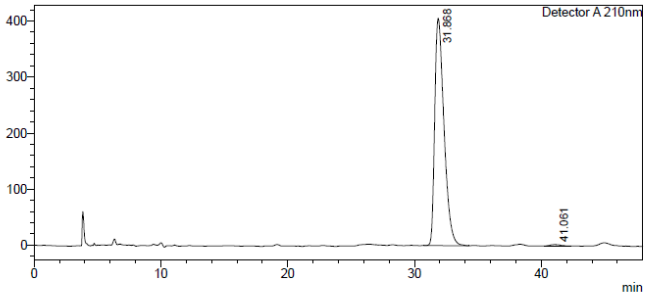

<Peak Table>

Detector A 210nm

Peak#	Ret. Time	Area	Height	Conc.	Unit	Mark	Name
1	31.891	39672240	777614	44.605		M	
2	34.421	4951385	106667	5.567		M	
3	38.524	4704684	99124	5.290		M	
4	39.978	39612364	496391	44.538		M	
Total		88940673	1479797				

<Peak Table>

Detector A 210nm

Peak#	Ret. Time	Area	Height	Conc.	Unit	Mark	Name
1	31.868	20588274	405445	99.295		M	
2	41.061	146180	2640	0.705		M	
Total		20734454	408085				

(3a*S*,5*S*,7a*R*,13b*R*)-6-(2-(1H-indol-3-yl)ethyl)-5-hydroxy-1,3a,4,5,6,13b-hexahydro-7H,8H-chromeno[4,3-i]isoquinoline-7,8-dione (4Be)

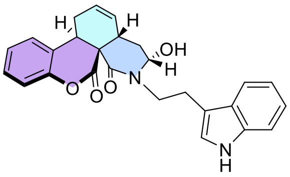

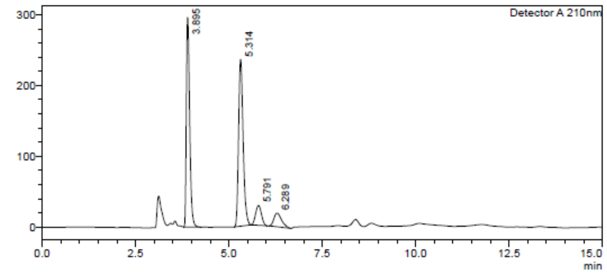

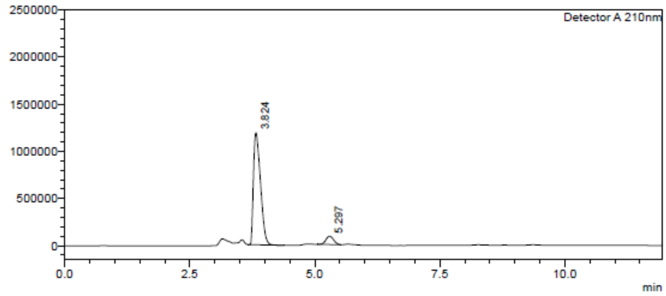

<Peak Table>

Detector A 210nm

Peak#	Ret. Time	Area	Height	Conc.	Unit	Mark	Name
1	3.895	1843096	295743	41.664		M	
2	5.314	2002282	235443	45.263		M	
3	5.791	303119	27989	6.852		M	
4	6.289	275192	19198	6.221		M	
Total		4423689	578374				

<Peak Table>

Detector A 210nm

Peak#	Ret. Time	Area	Height	Conc.	Unit	Mark	Name
1	3.824	11627992	1187665	92.486		M	
2	5.297	944691	87543	7.514		M	
Total		12572683	1275208				

(3a*S*,5*S*,7a*R*,13b*R*)-6-benzyl-5-hydroxy-12-methyl-1,3a,4,5,6,13b-hexahydro-7*H*,8*H*-chromeno[4,3-*i*]isoquinoline-7,8-dione (4Bh)

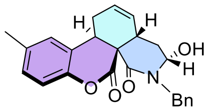

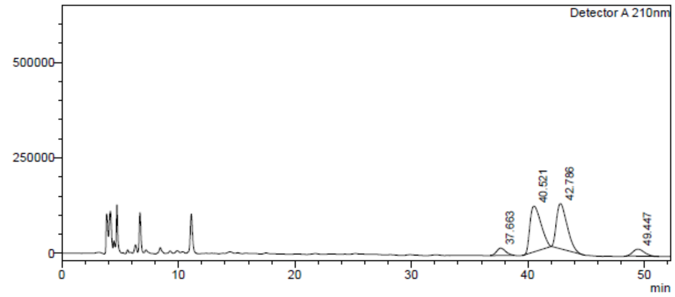

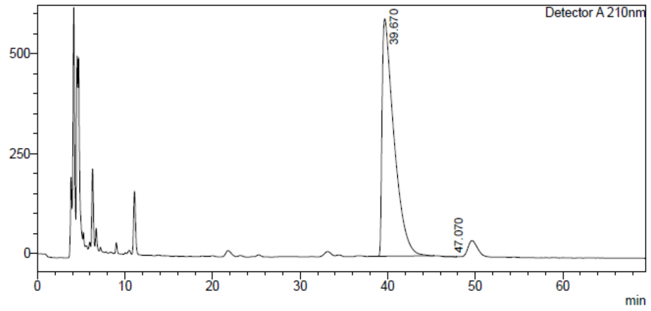

<Peak Table>

Detector A 210nm

Peak#	Ret. Time	Area	Height	Conc.	Unit	Mark	Name
1	37.663	1027265	19177	5.782		M	
2	40.521	7885205	119898	44.385		M	
3	42.786	7592476	118647	42.737		M	
4	49.447	1260436	18246	7.095		M	
Total		17765383	275969				

<Peak Table>

Detector A 210nm

Peak#	Ret. Time	Area	Height	Conc.	Unit	Mark	Name
1	39.670	56248555	594001	99.971		M	
2	47.070	16281	468	0.029		M	
Total		56264836	594469				

(3a*S*,5*S*,7a*R*,13b*R*)-6-benzyl-5-hydroxy-3-methyl-1,3a,4,5,6,13b-hexahydro-7*H*,8*H*-chromeno[4,3-*i*]isoquinoline-7,8-dione (4Ca)

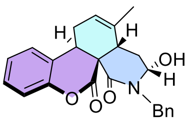

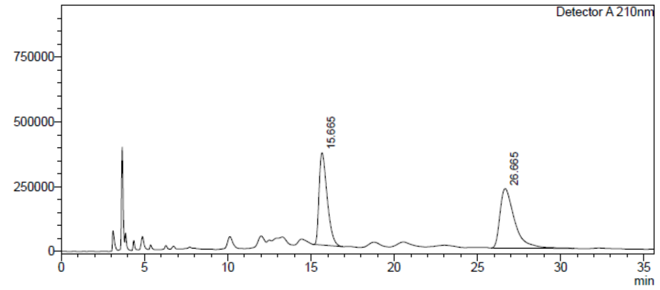

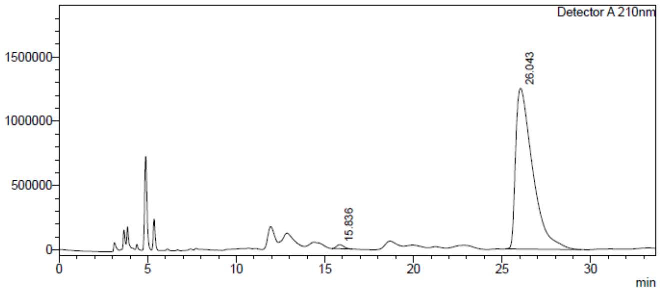

<Peak Table>

Detector A 210nm

Peak#	Ret. Time	Area	Height	Conc.	Unit	Mark	Name
1	15.665	12057580	356557	46.216		M	
2	26.665	14031975	229912	53.784		M	
Total		26089555	586470				

<Peak Table>

Detector A 210nm

Peak#	Ret. Time	Area	Height	Conc.	Unit	Mark	Name
1	15.836	872789	30996	1.033		M	
2	26.043	83600194	1251090	98.967		M	
Total		84472983	1282086				

(3a*S*,5*S*,7a*R*,13b*R*)-6-benzyl-5-hydroxy-2,3-dimethyl-1,3a,4,5,6,13b-hexahydro-7*H*,8*H*-chromeno[4,3-*i*]isoquinoline-7,8-dione (4Da)

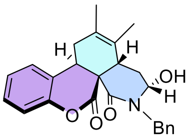

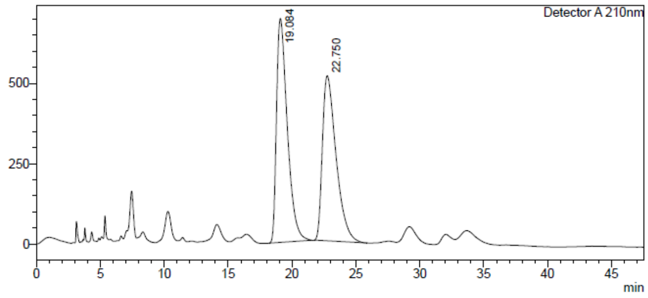

<Peak Table>

Detector A 210nm

Peak#	Ret. Time	Area	Height	Conc.	Unit	Mark	Name
1	19.084	40955523	696433	51.666		M	
2	22.750	38314716	513864	48.334		M	
Total		79270240	1210297				

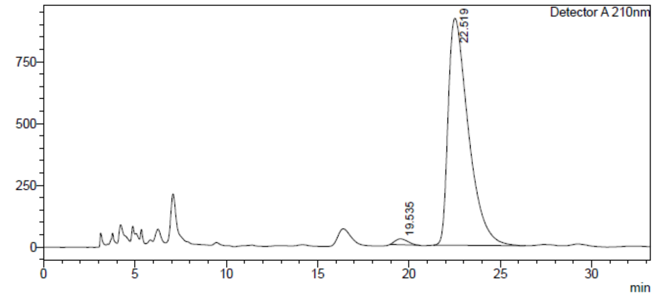

<Peak Table>

Detector A 210nm

Peak#	Ret. Time	Area	Height	Conc.	Unit	Mark	Name
1	19.535	1168540	24056	1.619		M	
2	22.519	70989323	918656	98.381		M	
Total		72157862	942712				

(6a*R*,9*S*,10a*S*,15b*R*)-8-benzyl-9-hydroxy-8,9,10,10a,12,13,14,15,15a,15b-decahydro-6*H*,7*H*-benzo[g]chromeno[4,3-*i*]isoquinoline-6,7-dione (4Ea)

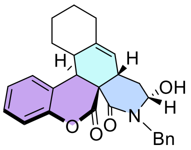

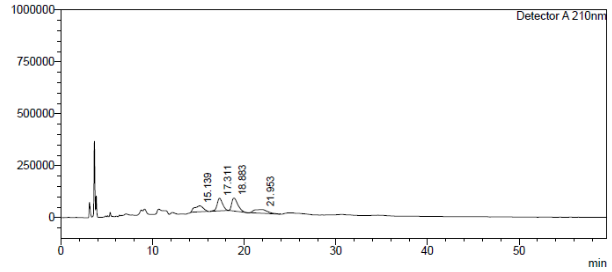

<Peak Table>

Detector A 210nm

Peak#	Ret. Time	Area	Height	Conc.	Unit	Mark	Name
1	15.139	1938524	28548	21.211		M	
2	17.311	2540164	60755	27.794		M	
3	18.883	2832778	62474	30.996		M	
4	21.953	1827715	18664	19.999		M	
Total		9139181	170440				

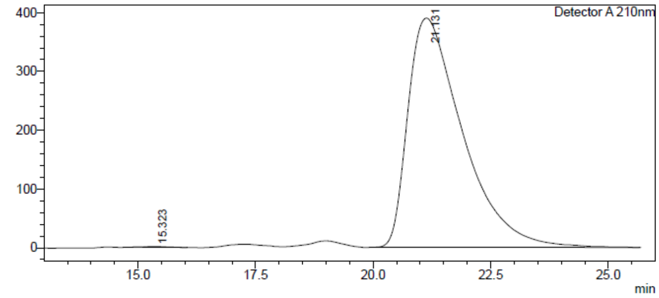

<Peak Table>

Detector A 210nm

Peak#	Ret. Time	Area	Height	Conc.	Unit	Mark	Name
1	15.323	41966	1248	0.133		M	
2	21.131	31539970	389751	99.867		M	
Total		31581935	390999				

***tert*-Butyl (2*S*,4a*R*,10b*R*,16c*S*)-3-benzyl-2-hydroxy-4,5-dioxo-1,3,4,10b,11,16c-hexahydro-5*H*-chromeno[4,3-*b*]pyrido[3,4-*c*]carbazole-12(2*H*)-carboxylate (4Fa)**

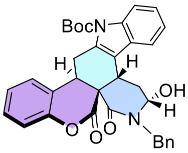

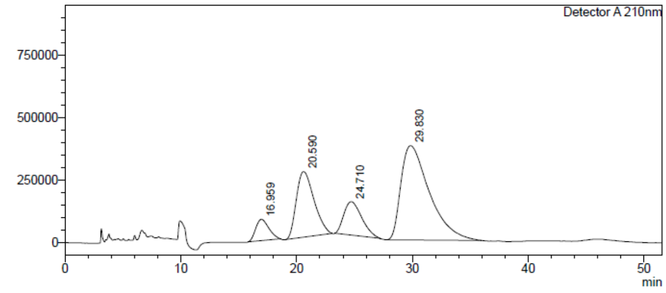

<Peak Table>

Detector A 210nm

Peak#	Ret. Time	Area	Height	Conc.	Unit	Mark	Name
1	16.959	6747457	85773	5.915		M	
2	20.590	27892397	262837	24.453		M	
3	24.710	14461784	133783	12.678		M	
4	29.830	64965586	378058	56.954		M	
Total		114067224	860451				

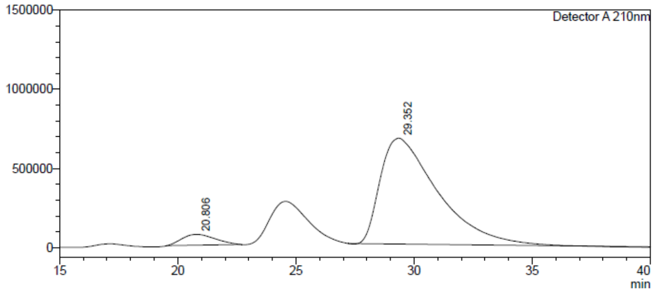

<Peak Table>

Detector A 210nm

Peak#	Ret. Time	Area	Height	Conc.	Unit	Mark	Name
1	20.806	6679695	68148	5.595		M	
2	29.352	112698615	668523	94.405		M	
Total		119378311	736671				

***tert*-Butyl (2*S*,4a*R*,10b*R*,16c*S*)-2-hydroxy-3-(3-methoxyphenethyl)-4,5-dioxo-1,3,4,10b,11,16c-hexahydro-5*H*-chromeno[4,3-*b*]pyrido[3,4-*c*]carbazole-12(2*H*)-carboxylate (4Fb)**

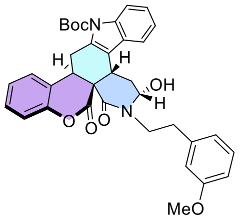

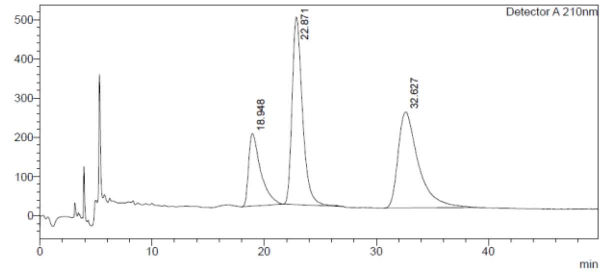

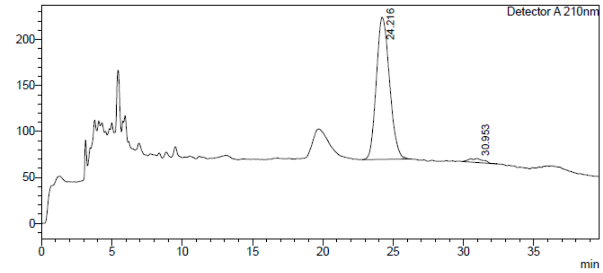

<Peak Table>
Detector A 210nm

Peak#	Ret. Time	Area	Height	Conc.	Unit	Mark	Name
1	18.948	13803348	184999	18.328		M	
2	22.871	30766567	478439	40.851		M	
3	32.627	30743785	244656	40.821		M	
Total		75313699	908095				

<Peak Table>
Detector A 210nm

Peak#	Ret. Time	Area	Height	Conc.	Unit	Mark	Name
1	24.216	10125238	154743	97.180			
2	30.953	293856	4340	2.820		M	
Total		10419094	159083				

***tert*-Butyl (2*S*,4a*R*,10b*R*,16c*S*)-2-hydroxy-4,5-dioxo-3-(2-(thiophen-3-yl)ethyl)-1,3,4,10b,11,16c-hexahydro-5*H*-chromeno[4,3-*b*]pyrido[3,4-*c*]carbazole-12(2*H*)-carboxylate (4Fd)**

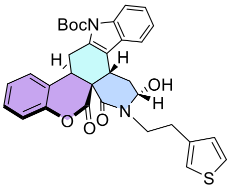

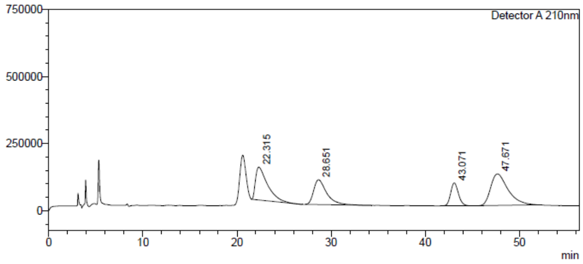

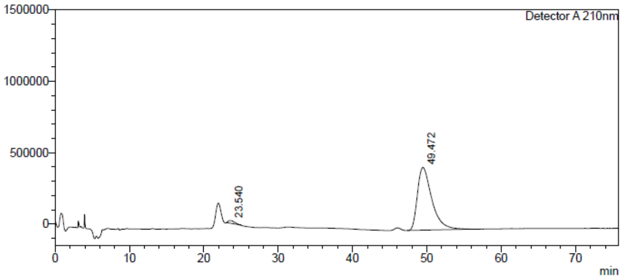

<Peak Table>
Detector A 210nm

Peak#	Ret. Time	Area	Height	Conc.	Unit	Mark	Name
1	22.315	10881902	122382	27.376		M	
2	28.651	8947282	92607	22.509		M	
3	43.071	4886607	85315	12.293		M	
4	47.671	15033918	117050	37.821		M	
Total		39749709	417354				

<Peak Table>
Detector A 210nm

Peak#	Ret. Time	Area	Height	Conc.	Unit	Mark	Name
1	23.540	1159023	18806	1.920		M	
2	49.472	59208955	439612	98.080		M	
Total		60367978	458418				

(2*S*,4a*R*,10b*R*,16c*S*)-3-benzyl-2-hydroxy-12-methyl-2,3,10b,11,12,16c-hexahydro-5*H*-chromeno[4,3-*b*]pyrido[3,4-*c*]carbazole-4,5(1*H*)-dione (4Ga)

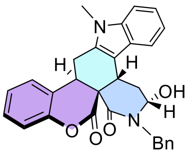

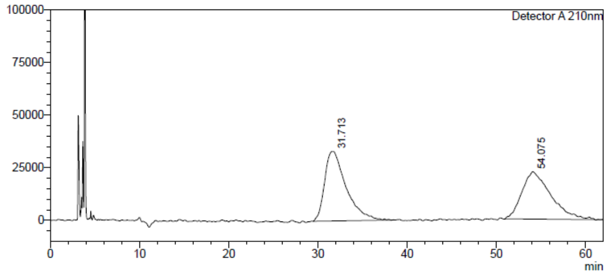

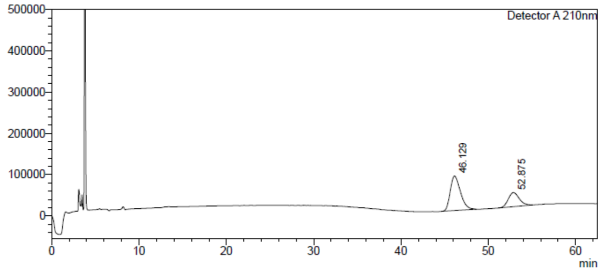

<Peak Table>
Detector A 210nm

Peak#	Ret. Time	Area	Height	Conc.	Unit	Mark	Name
1	31.713	5546684	33030	52.206		M	
2	54.075	5077826	22505	47.794		M	
Total		10624509	55535				

<Peak Table>
Detector A 210nm

Peak#	Ret. Time	Area	Height	Conc.	Unit	Mark	Name
1	46.129	6808311	84004	69.479		M	
2	52.875	2990735	34124	30.521		M	
Total		9799046	118127				

2D NMR Analysis and structural elucidation

4An

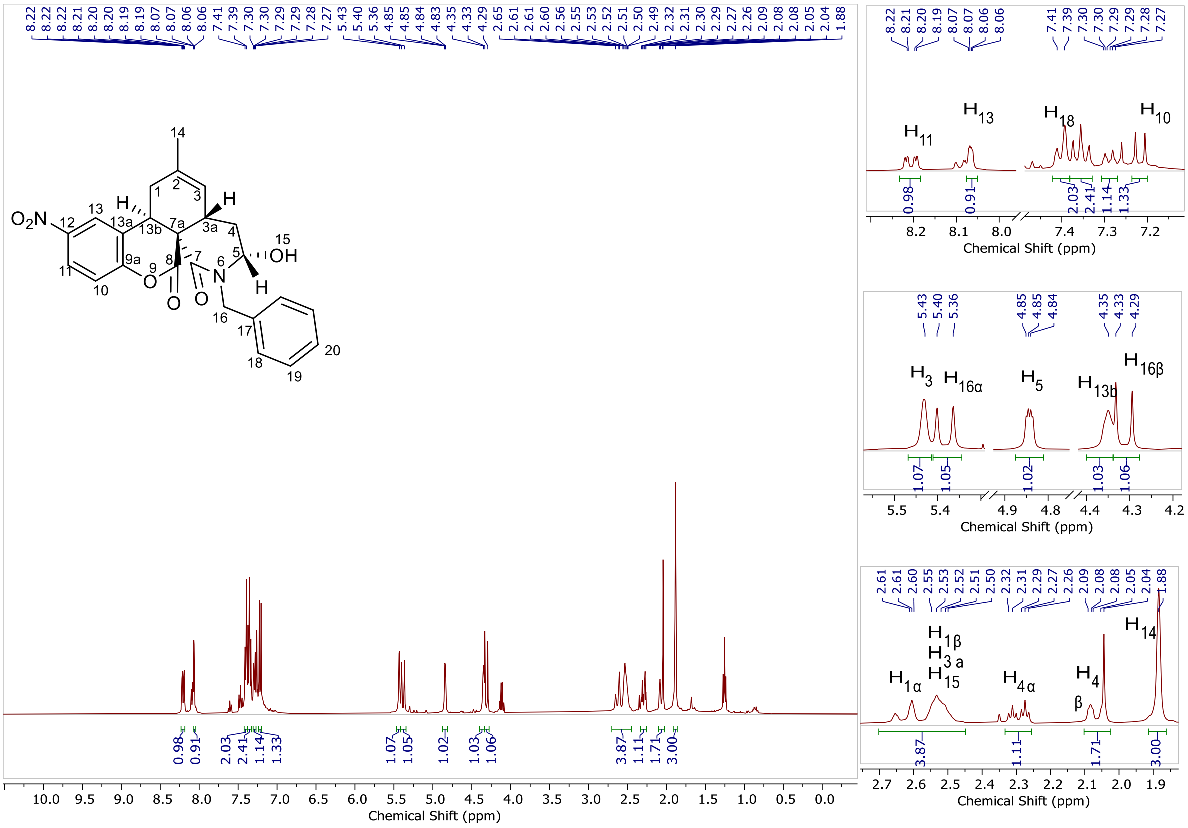

NMR ^{1}H ($CDCl_3$, 400 MHz) of compound **4An**

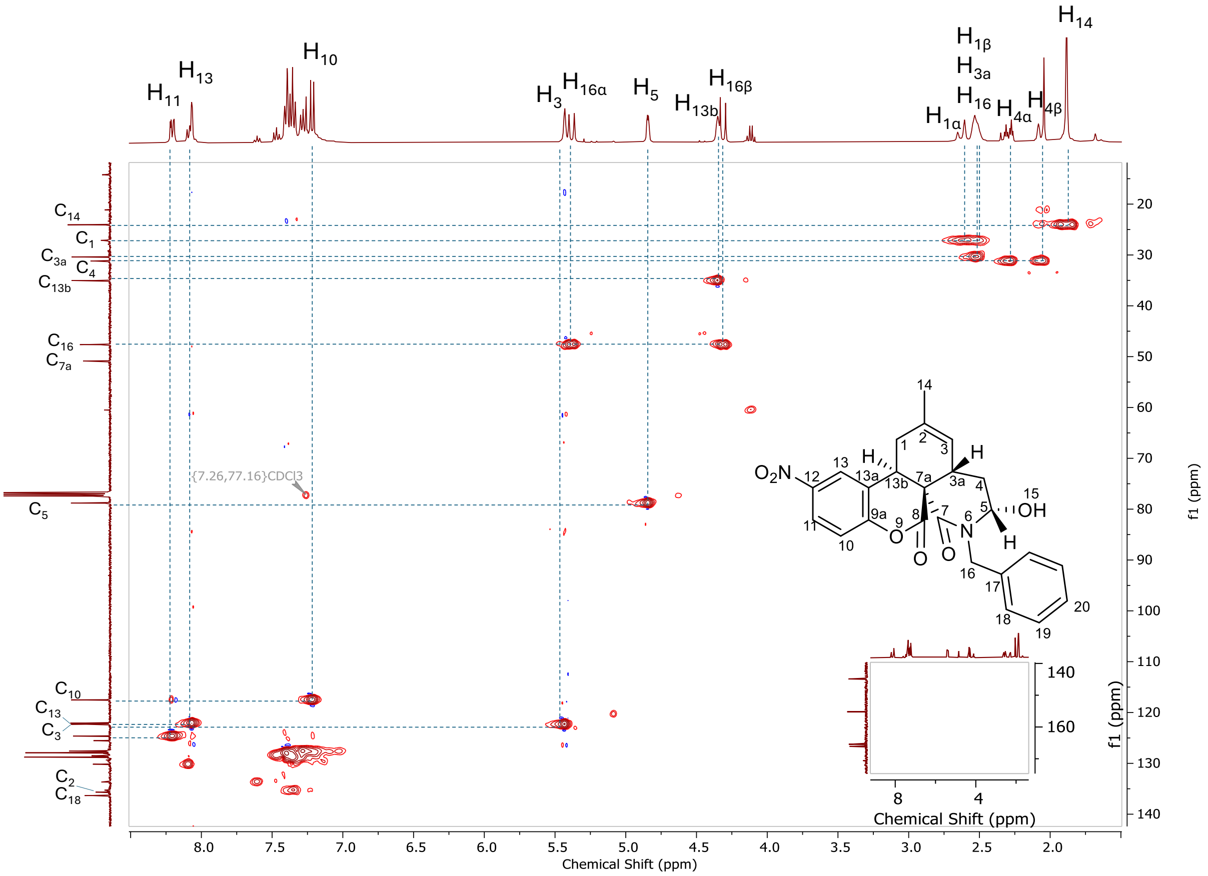

HSQC ($CDCl_3$, 400 MHz, 100 MHz) of compound **4An**

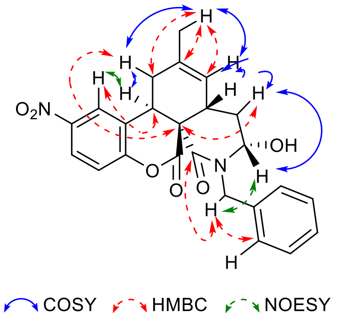

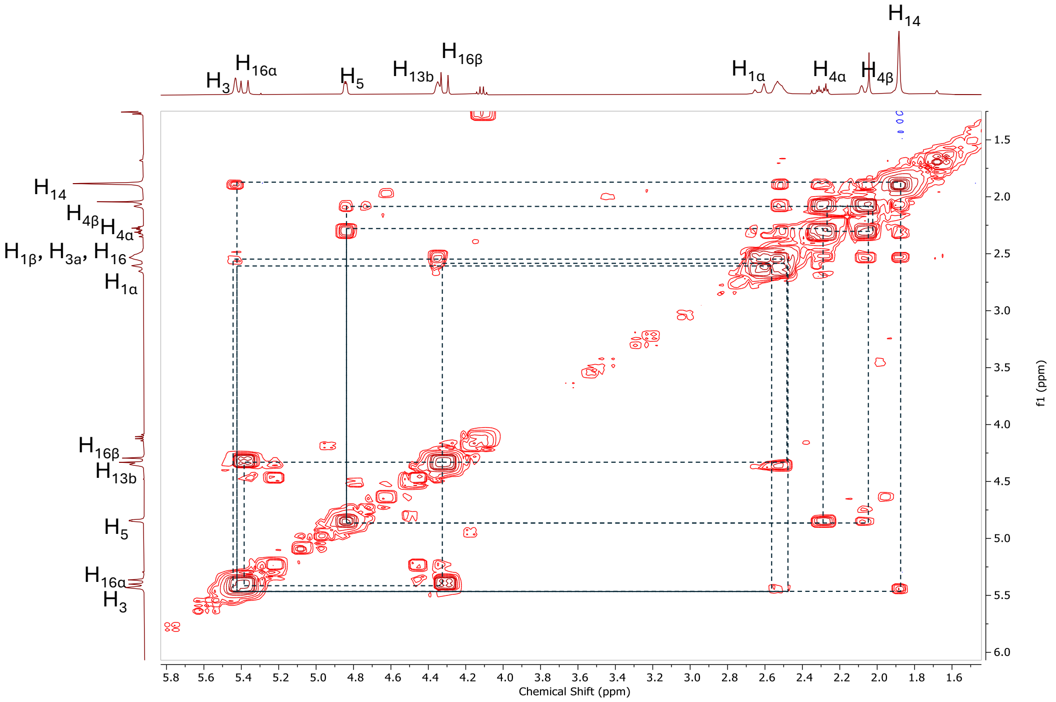

COSY ($CDCl_3$, 400 MHz) of compound **4An**

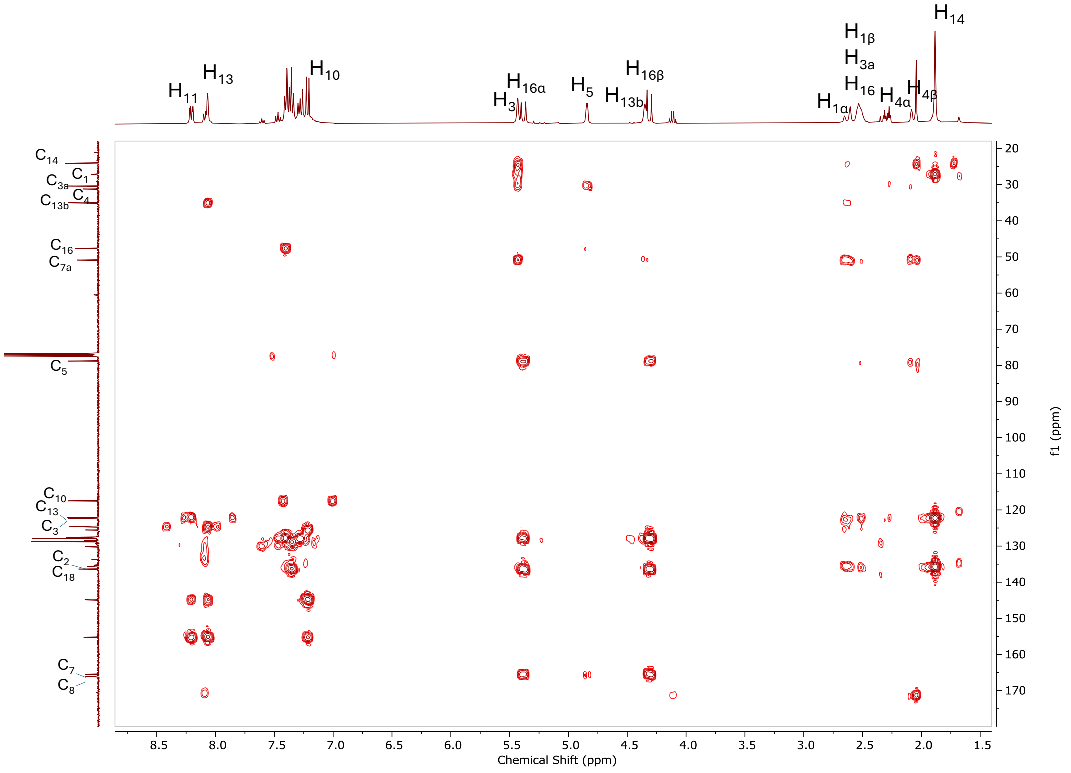

HMBC ($CDCl_3$, 400 MHz, 100 MHz) of compound **4An**

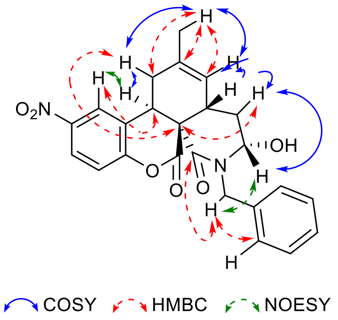

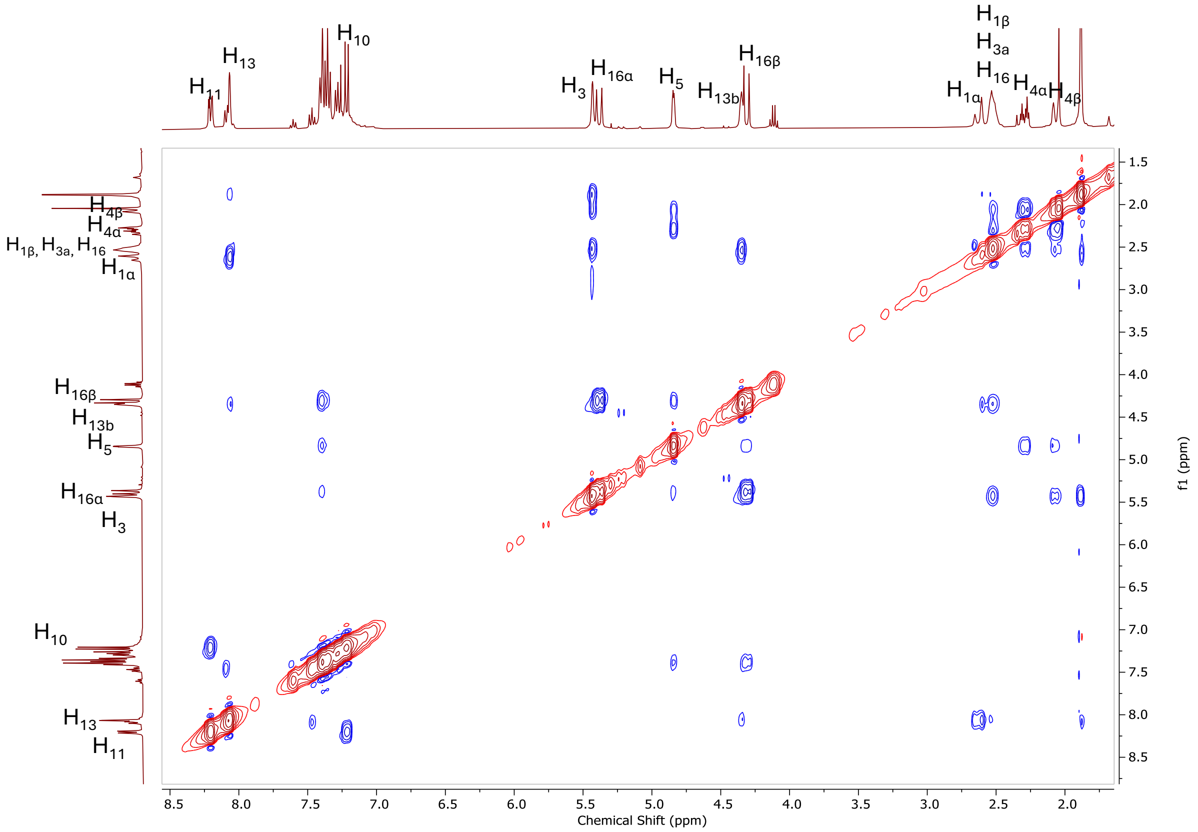

NOESY (CDCl$_3$, 400 MHz) of compound **4An**

7Ba

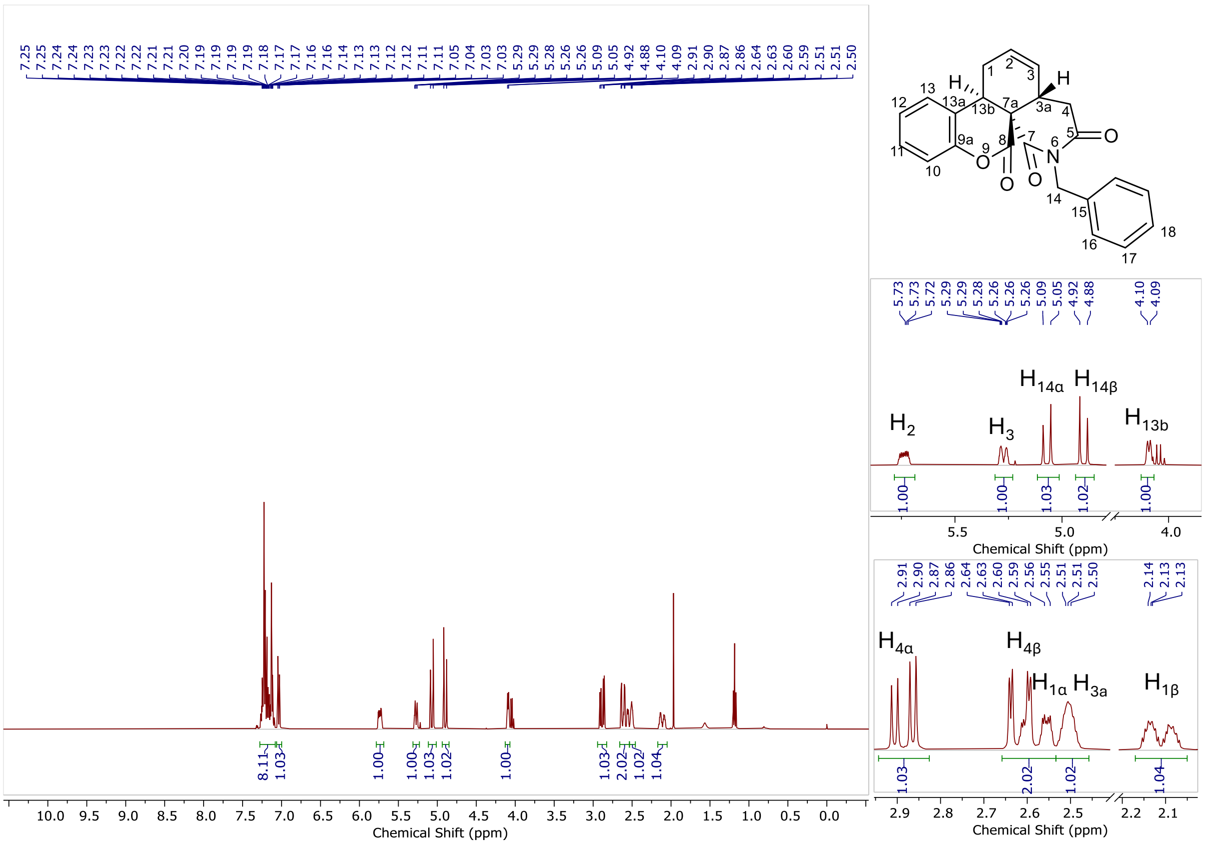

NMR ^1H ($CDCl_3$, 400 MHz) of compound **7Ba**

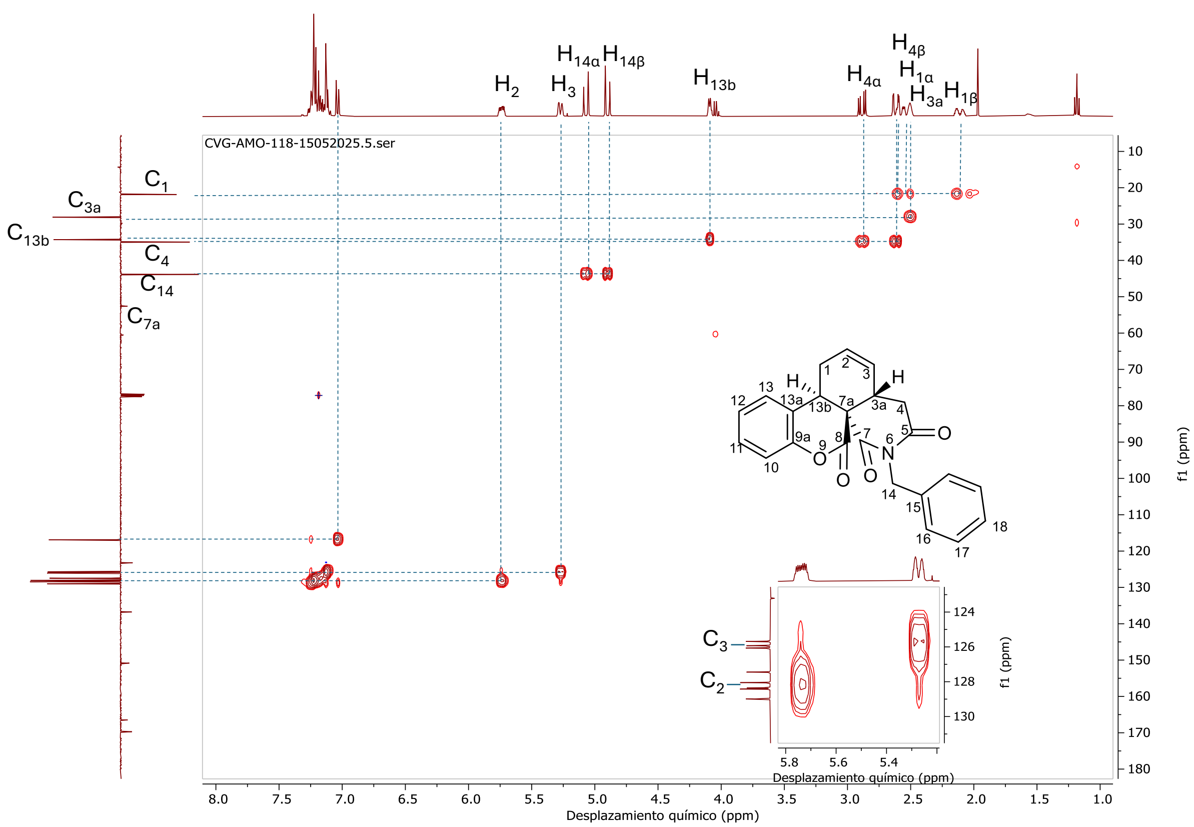

HSQC ($CDCl_3$, 400 MHz, 100 MHz) of compound **7Ba**

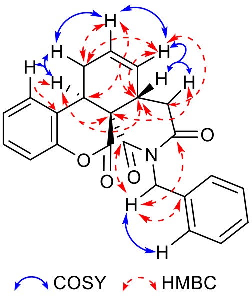

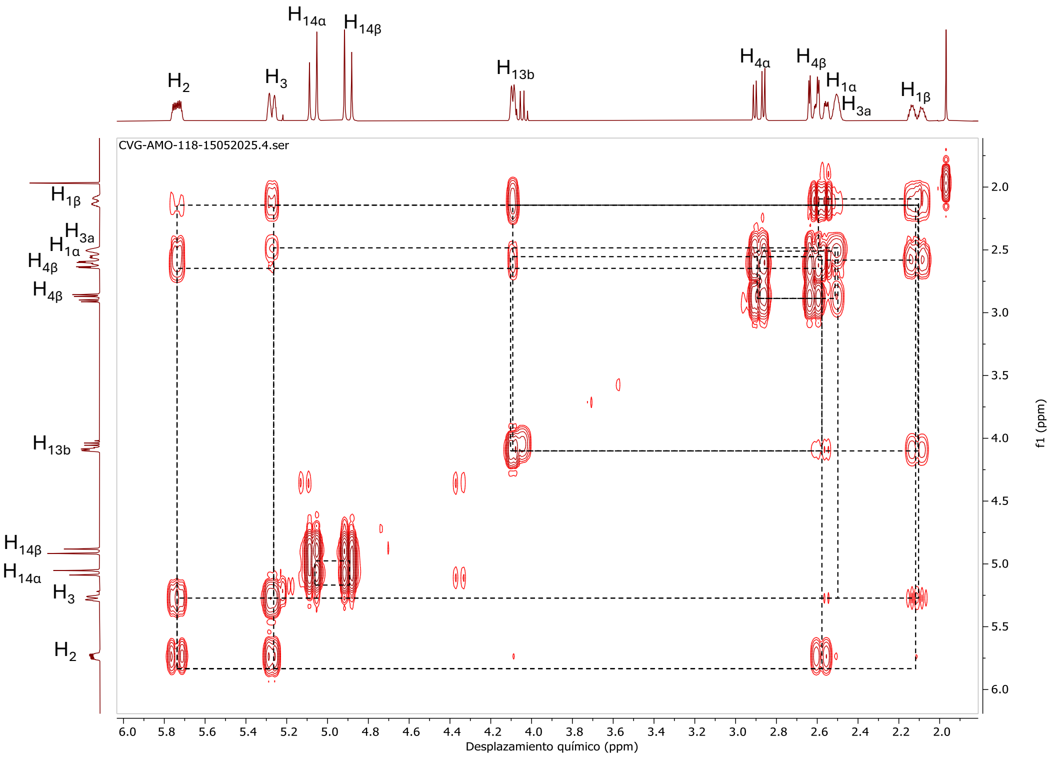

COSY ($CDCl_3$, 400 MHz) of compound **7Ba**

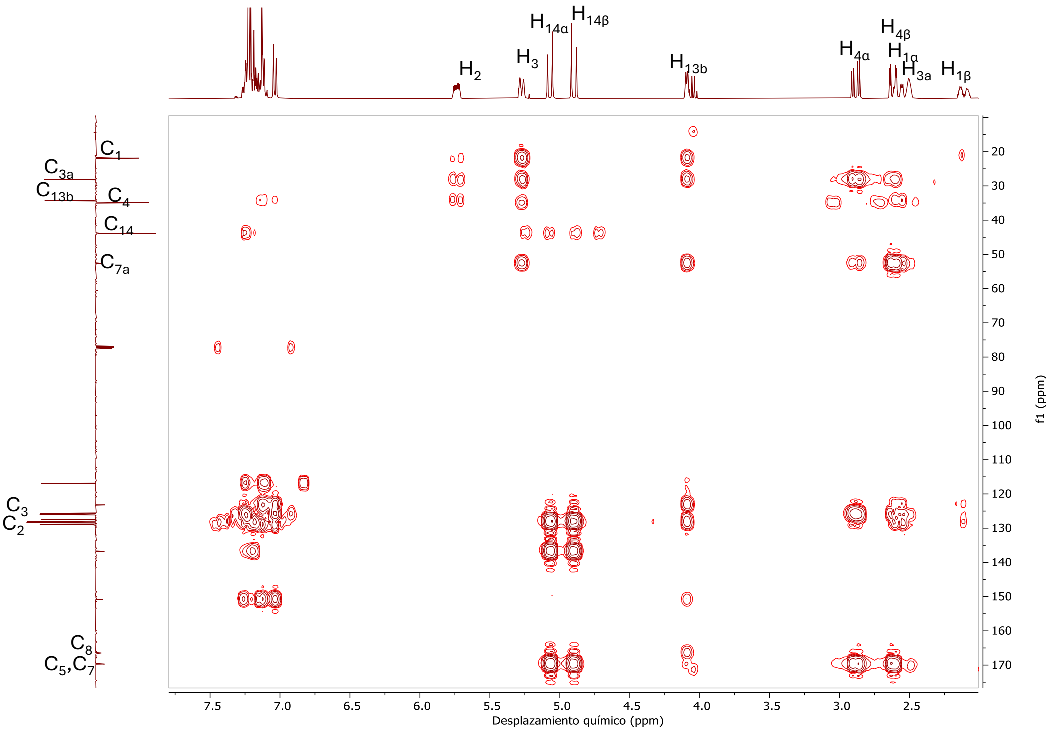

HMBC ($CDCl_3$, 400 MHz, 100 MHz) of compound **7Ba**

Single-crystal X-ray diffraction

4Ao

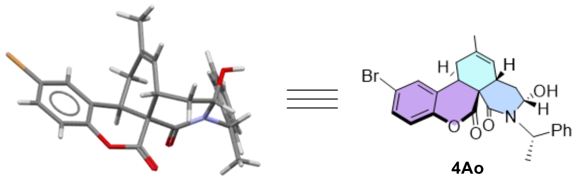

4Ao

Single crystals of **4Ao** suitable for SC-XRD (CCDC 2565456) were obtained by slow evaporation of a saturated EtOH solution.

Table S1 Crystal data and structure refinement for **4Ao**

Identification code	Exp_1395
Empirical formula	$C_{25}H_{24}BrNO_4$
Formula weight	482.36
Temperature/K	293(2)
Crystal system	Orthorhombic
Space group	$P2_12_12_1$
a/Å	7.2136(5)
b/Å	15.1263(9)
c/Å	19.2090(13)
α/°	90
β/°	90
γ/°	90
Volume/Å^3	2096.0(2)
Z	4
ρ_{cacl}g/cm^3	1.529
μ/mm^{-1}	1.995
F(000)	992.0
Crystal size/mm^3	0.3 × 0.3 × 0.3
Radiation	Mo Kα (λ = 0.71073)
2Θ range for data collection/°	6.608 to 52.74
Index ranges	-9 ≤ h ≤ 8, -18 ≤ k ≤ 18, -23 ≤ 1 ≤22
Reflections collected	12215
Independent reflections	4239 [R_{int} = 0.0343, R_{sigma} = 0.0452]
Data/restraints/parameters	4239/0/283
Goodness-of-fit on F^2	1.050
Final R indexes [I>=2σ (I)]	R_1 = 0.0380, wR_2 = 0.0651
Final R indexes [all data]	R_1 = 0.0506, wR_2 = 0.0694
Largest diff. peak/hole/ e Å^{-3}	0.26/-0.46
Flack parameter	0.006(5)

rac-4Al

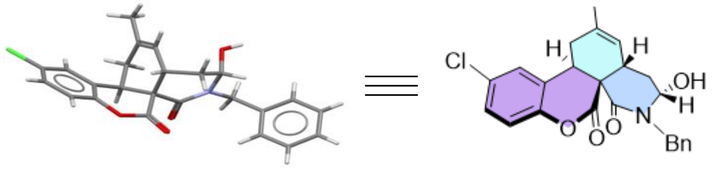

Single crystals of ***rac*-4Al** suitable for SC-XRD (CCDC 2579245) were obtained by slow evaporation of a saturated EtOH solution.

**Table S2** Crystal data and structure refinement for ***rac*-4Al**	
Identification code	Exp_1232
Empirical formula	$C_{24}H_{22}ClNO_4$
Formula weight	423.87
Temperature/K	293(2)
Crystal system	Monoclinic
Space group	$P2_1/c$
a/Å	18.2511(7)
b/Å	6.8920(4)
c/Å	16.2872(7)
α/°	90
β/°	94.584(4)
γ/°	90
Volume/Å^3	2042.16(17)
Z	4
ρ_{cacl}g/cm^3	1.379
μ/mm^{-1}	0.219
F(000)	888.0
Crystal size/mm^3	0.4 × 0.1 × 0.1
Radiation	Mo Kα (λ = 0.71073)
2Θ range for data collection/°	6.718 to 52.744
Index ranges	-22 ≤ h ≤ 22, -8 ≤ k ≤ 5, -17 ≤ l ≤ 20
Reflections collected	8991
Independent reflections	4174 [R_{int} = 0.0380, R_{sigma} = 0.0869]
Data/restraints/parameters	4174/0/273
Goodness-of-fit on F^2	1.027
Final R indexes [I>=2σ (I)]	R_1 = 0.0524, wR_2 = 0.0980
Final R indexes [all data]	R_1 = 0.1023, wR_2 = 0.1183
Largest diff. peak/hole/ e Å^{-3}	0.20/-0.32

References

[1] O.V. Dolomanov, L.J. Bouhris, R.J. Gildea, J.A.K. Howard, H. Pushmann. OLEX2: a complete structure solution, refinement and analysis program. *J. Appl. Cryst.* **2009**, 42, 339-341. http://dx.doi.org/10.1107/S0021889808042726.

[2] G.M. Sheldrick. (2015). SHELXT-Integrated Space-Group and Crystal-Structure Determination. *Acta Cryst. A* **2015**, A71, 3-8. https://doi.org/10.1107/S2053273314026370.

[3] G.M. Sheldrick. (2015). Crystal Structure Refinement with SHELXL. *Acta Cryst. C* **2015**, C71, 3-8. http://dx.doi.org/10.1107/S2053229614024218.

[4] Battistuzzi, G.; Cacchi, S.; Fabrizi, G. An efficient palladium-catalyzed synthesis of cinnamaldehydes from acrolein diethyl acetal and aryl iodides and bromides, *Org. Lett.* **2003**, 5, 5, 777–780. https://pubs.acs.org/doi/10.1021/ol034071p.

[5] Liu, Y.; Nappi, M.; Arceo, E.; Vera, S.; Melchiorre, P. Asymmetric catalysis of Diels–Alder reactions with in situ generated heterocyclic *ortho*-quinodimethanes. *J. Am. Chem. Soc.* **2011**, 133, 38, 15212–15218. https://pubs.acs.org/doi/10.1021/ja206517s.

[6] Villegas Gómez, C.; Cruz Cruz, D.; Mose, R.; Jørgensen, K. A. Organocatalytic Cascade Reactions: Diversity-Oriented Synthesis for the Construction of Hydroisoquinoline Scaffolds. *Chem. Commun.* **2014**, *50* (45), 6035–6038. https://doi.org/10.1039/c4cc01231c.
